# Which combinations of behaviour change techniques work best? Evidence from meta-review of interventions addressing healthy diet and exercise after breast cancer

**DOI:** 10.1186/s12966-026-01961-6

**Published:** 2026-08-04

**Authors:** Monika Boberska, Dominika Wietrzykowska, Ewa Kulis, Julia Kuzminska, Karolina Zalewska-Lunkiewicz, Eva Karaglani, Niki Mourouti, Yannis Manios, Aleksandra Luszczynska, the MELIORA Consortium, Anna Gavrieli, Christos Diou, Christina Pelekanou, Agathi Ntzouvani, Aristotelis Ballas, Ioannis Ioakeimidis, Miguel Cejalvo, Juan Andújar, Juan Antonio Carbonell-Asins, Rosana Pau Landa, Carlos Peña de los Santos, Sheila Romero Chaveli, Maite Martínez Martínez, Marta Tapia Céspedes, Begoña Bermejo de las Heras, Blanca Alabadí, Miguel Civera, Ana Miralles Marco, Gintare Kaliniene, Erika Korobeinikova, Ricardas Radisauskas, Lina Paulauskienė, Ida Liseckiene, Renata Kudukyte-Gaspere, Rugile Ivanauskiene, Anita Wanngren, Britt Sandberg, Roelinde Middelveld,, Linda Engström, Ana Molina Barceló, Mireia Gandia Prat, Oscar García-Sauret, Teresa de Pablo Pardo, Raquel Soler Blasco, Šarūnas Narbutas, Neringa Čiakienė, Asta Tamulėnė, Aikaterini Palascha, Nina McGrath, Laura Bosman, Justine van der Feen, Ana Roca-Umbert, Frans Folkvord, Francisco Lupiáñez-Villanueva, Anna-Maria Krooupa, Nikos Giatsoglou, Vicky Fiska, Antonis Tsanakas, Spiros Nikolopoulos,, Nikos Sarris, Symeon Papadopoulos, Mariangela Panniello, Maria Plakia, Lorenzo Giuliani, Tasos Litsas, Konstantinos Kalampokis, Theodora Karali, Alexios Camarinopoulos, Ilias Maglogiannis, Andreas Menychtas, Christos Panagopoulos, Antonis Pardos, Vasilis Athanasiou, Lisa Becker, Giuseppe Martone, Laurène Mathey, Alexis Strader, Alexandra Olson, Marta Burgos, Gemma Galdón, Odysseas Androutsos, Vaios Svolos, Maria Koutsaki

**Affiliations:** 1 Wroclaw Faculty of Psychology, SWPS University, 30B Ostrowskiego St., Wroclaw, 53-238 Poland; 2 Katowice Faculty of Psychology, SWPS University, 9 Technikow St., Katowice, 40-326 Poland; 3 Department of Nutrition and Dietetics, School of Health Science and Education, Harokopio University, 70 El. Venizelou (Thiseos) Ave, Kallithea, Athens 17671 Greece; 4 European Centre for Obesity, Harokopio University, 70 El. Venizelou Ave, Kallithea-Attica, Athens 17671 Greece; 5 Institute of Agri-Food and Life Sciences, Hellenic Mediterranean University Research Centre, Estavromenos, Heraklion, Crete 71410 Greece; 6 Lyda Hill Institute for Human Resilience, University of Colorado at Colorado Springs, 4863 North Nevada Ave, Colorado Springs, CO 80918 USA

**Keywords:** Breast cancer, Physical activity, Diet, Healthy body weight, Umbrella review, Behaviour change techniques

## Abstract

**Background:**

The primary aim was to identify combinations including ≥ 3 behavior change techniques (BCTs) that are jointly used in behavioral interventions for women with/survivors of breast cancer, and result in a simultaneous improvement of physical activity, healthy nutrition behaviour, and healthy body weight maintenance.

**Methods:**

A meta-review integrated evidence from systematic, scoping, and pragmatic reviews. Ten databases were searched using ASReviews software and through manual searches, resulting in the inclusion of 32 reviews. The ROBIS tool was applied to assess bias risk. All combinations of ≥ 3 BCTs were identified in the original studies, and ratios of studies indicating a significant improvement to those with no improvement were calculated for three outcome domains. BCT combinations used in ≥ 10 original intervention studies (with ≥ 3 studies per each outcome domain) and supported in ≥ 85% of intervention studies were identified as the most promising.

**Results:**

A total of *k* = 17,715 combinations were identified, including 3–12 BCTs. Only 46 combinations met the criteria for the most promising ones (supported by ≥ 85% of studies), with 7 combinations supported in ≥ 90% of studies. The promising combinations contained 3–6 BCTs included the following: goal setting (behaviour), problem solving, demonstration of the behaviour, self-monitoring of behaviour, instruction on how to perform the behaviour, feedback on behaviour, credible source. Interestingly, goal setting (related to behaviour or outcome) was present in 22 of the supported combinations, whereas demonstration of behaviour, problem solving, and self-monitoring were included more frequently (33, 32, and 26 of supported combinations, respectively).

**Conclusions:**

This meta-review offers a novel approach to identifying BCT combinations that are jointly used in interventions resulting in an improvement in physical activity, healthy nutrition behaviour, and healthy body weight maintenance. These evidence-based combinations may serve as examples of good practices for promoting a healthy lifestyle in the context of breast cancer.

**Trial registration:**

PROSPERO database registration: #CRD420251130311.

**Supplementary Information:**

The online version contains supplementary material available at https://doi.org/10.1186/s12966-026-01961-6.

## Background

In Europe, breast cancer causes more than 160,000 deaths, and nearly 4 million disability-adjusted life years are lost annually [1]. Health behaviours, including unhealthy nutrition choices, low levels of physical activity, and high alcohol consumption, account for approximately 22% of the chronic disease burden related to breast cancer [1]. Additionally, adherence to healthy dietary recommendations is associated with a 23% reduction in overall mortality among patients with/survivors of breast cancer [2], while post-diagnosis physical activity may reduce the risk of breast cancer recurrence by up to 35% [3]. Finally, overweight and obesity at the time of breast cancer diagnosis increase the risk of a second cancer diagnosis, second breast cancer, and any obesity-related cancers [4].

Behaviour change interventions that target the adoption and maintenance of a healthy diet and active lifestyle usually include numerous interacting components. The complexity of these interventions and differences in their content resulted in the need to break them down into minimal active components. Using an international consensus approach, Michie and colleagues [5] proposed that these smallest components, called behaviour change techniques (BCTs), may be organized into a 93-technique BCT taxonomy [5]. BCTs are specific procedures used to change people’s beliefs, capabilities, emotions, knowledge, and other psychosocial processes, which, in turn, result in a behaviour change [5–7]. BCTs are replicable, step-by-step processes which can be organized into 16 broad groups: goals and planning, feedback and monitoring, social support, shaping knowledge, natural consequences, comparison of behaviour, associations, repetition and substitution, comparisons of outcomes, reward and threat, regulation, antecedents, identity, scheduled consequences, self-belief, and covert learning [5].

One of the core tasks of behaviour change research is identifying BCTs that are characteristic of interventions targeting the maintenance of physical activity, a healthy diet, and a healthy body weight. A number of original studies and systematic reviews aimed to determine which BCTs are *most commonly used* in interventions addressing nutrition, physical activity, or healthy body weight maintenance among women with/survivors of breast cancer. For example, Cooper et al. [8] found that, among digital physical activity interventions developed for women with breast cancer, self-monitoring and goal setting were the most frequently used techniques. A systematic review of trials aiming at weight management, healthy nutrition, and physical activity suggested that the most commonly used techniques were goal setting (in respect to behaviour), self-monitoring, instructions on the target behaviour, and a credible source [9].

Other research and systematic reviews attempted to identify which BCT(s) is (are) typical of effective interventions. In this case, the research strategy usually relied on identifying whether studies including a specific BCT (considered in isolation from other BCTs) resulted in significant changes in the respective outcomes or investigating the average effect of the studies with versus without a specific BCT. For example, Hailey et al. [10] reviewed interventions designed to increase physical activity among survivors of breast cancer and found that adding objects to the environment was the BCT with the greatest potential to increase in exercise levels, followed by goal setting (in respect to behaviour) and self-monitoring.

Existing approaches, pooling data from experimental studies to draw conclusions about the associations between a single BCT (considered in isolation) and the effects of interventions on the studied outcomes, have several limitations. One of the main drawbacks of these approaches is their focus on the links between a single BCT and the effectiveness of an intervention [11]. Interventions almost always include a combination of several BCTs, but research synthesizing evidence on key BCTs has usually relied on singling out one specific BCT (e.g., Technique A) and checking if it is used (or not) in effective versus ineffective interventions [10, 12]. After identifying the BCTs that individually meet the threshold for being considered supported, a list of respective techniques (e.g., techniques A, B, C) is formed. Each technique is identified in a separate analysis and considered part of a “combination” of effective BCTs. However, such statements are not typically followed by analyses investigating whether the specific BCT combination, viewed as a single entity, is genuinely present in an intervention and linked to the beneficial intervention outcomes. In other words, it is not checked whether interventions that include the actual “A-B-C” BCT combinations are actually effective. Instead, this approach demonstrates that the presence of any single BCT (A, B, or C) characterizes a successful intervention, though it remains unclear whether they must be used jointly. Investigating combinations rather than isolated BCTs is crucial, as certain combinations can interact negatively and worsen outcomes, even if those same techniques are beneficial in other groupings [13].

Although there is plenty of reviews on the effectiveness of BCTs, research evaluating the effects of *BCT combinations* is scarce. This is true in any health context and for any population (not only breast cancer) and for any behavioral outcome. To the best of our knowledge, only a few systematic reviews have aimed to identify specific BCT combinations used in behavioral interventions. For example, Cotie et al. [14] examined physical activity interventions (for any population) using the just-in-time adaptive approach, and elicited 2-component combinations that are most frequently used in this context. This approach provides very limited insight as only 2 components were considered. Another review [15] attempted to elicit associations between theory-based, multi-BCT combinations and physical activity outcomes among adults with overweight or obesity. Unfortunately, the analysis of such associations was not possible due to a large number of different BCT combinations and inconsistent use [15]. Concluding, we know very little about BCT combinations that may be used in interventions that are effective in specific contexts.

This meta-review aims at identifying BCT combinations, defined as sets of ≥ 3 BCTs used together in effective behavioral interventions. We assumed that a BCT combination could include techniques chained in any order; the A-B-C, B-A-C, and C-A-B sequences were considered to represent the same combination. Our meta-review was conducted in the context of breast cancer and included women with breast cancer, those undergoing cancer treatment, and survivors of breast cancer. The main research question was to determine the composition of BCT combinations that are used in behavioral interventions delivered to women with/survivors of breast cancer that result in beneficial changes occurring consistently across three domains of outcomes: physical activity, nutrition behaviour, and healthy weight maintenance.

## Methods

### Design

This systematic review of reviews (a meta-review; [16]) integrates empirical evidence from existing systematic reviews, scoping reviews, and pragmatic reviews. The study was preregistered with the PROSPERO database, no. 420251130311, and it followed Preferred Reporting Items for Systematic Reviews and Meta-Analyses (PRISMA) guidelines [17]. This study utilizes the results of a search reported in an earlier meta-review [18], which singled out six BCTs that were used in effective interventions promoting healthy lifestyles in women with/survivors of breast cancer. In contrast to the previous study ([18] we focused on BCT combinations.

## Procedures

### Search strategy

Nine databases were searched: Academic Research Source eJournals, Academic Search Ultimate, APA PsycArticles, APAPsycInfo, ERIC, Health Source – Consumer Edition, Health Source – Nursing/Academic Edition, MasterFILE Premier, MEDLINE (using the EBSCO platform), and SCOPUS. The systematic searches were followed by reference checks of the identified reviews to double-check for any additional records. No time restrictions were applied, and the searches were completed in 2024.

In line with the Population-Intervention-Comparison-Outcomes (PICO) guidelines [19] combinations of five types of groups of keywords were applied, referring to (1) population (women with breast cancer or survivors); (2) outcomes (e.g., healthy nutrition, physical activity, healthy body weight); (3) applied intervention (e.g., use of BCT, motivational interviewing); and (4 and 5) type of intervention and study design (i.e., systematic reviews of behavioral interventions). The full list of keywords is reported in the Additional file Table S1. The keywords referring to the population, intervention, and outcomes were based on previous reviews [9, 10, 20]. The keywords applied to identify reviews were derived from the list proposed by Hennessy et al. [16].

**Fig. 1:**
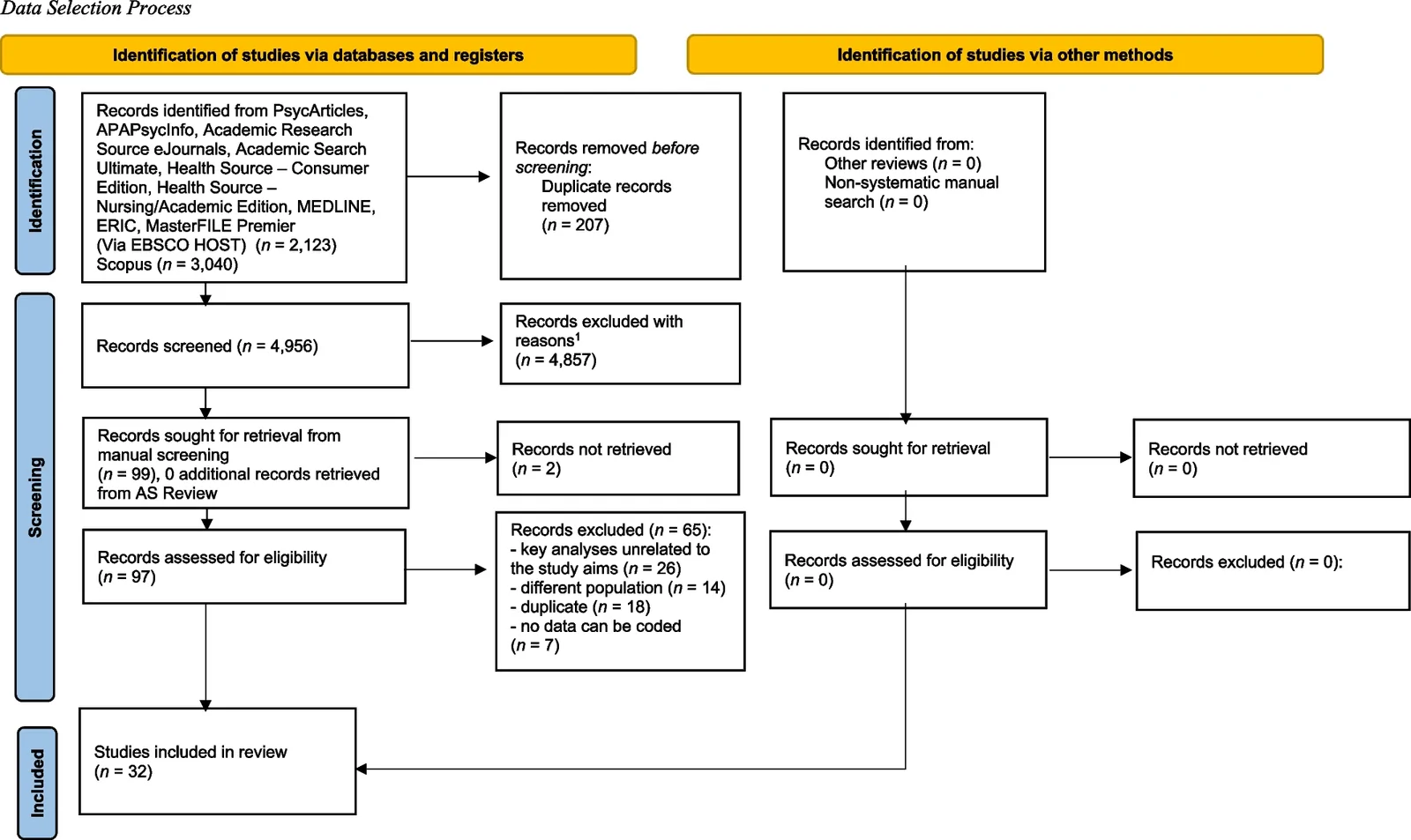
Data selection process. ^1^Records excluded from databases other than SCOPUS: Key analyses unrelated to the study aims (*n* = 300), a different type of study (umbrella review or report of single study *(n* = 1,372), different population (not breast cancer) (*n* = 195), duplicates (*n* = 9). Records excluded from SCOPUS: A different type of a study (umbrella review or report of a single study) (*n* = 2,338), different population (not breast cancer) (*n* = 522), key analyses unrelated to the study aims (*n* = 92), duplicates (*n* = 3), duplicates between EBSCO and SCOPUS (*n* = 24), no full text available (*n* = 1), different language than English (*n* = 1). ASReview was used to facilitate machine-assisted screening alongside manual screening. ASReview-based process identified the same articles as the manual review

The data selection process and flow of studies are reported in Fig. 1.

The initial search yielded *n* = 5,163 records, of which *n* = 4,956 records remained after the removal of duplicates. All abstracts were screened by two researchers (MB, DW), and potential conflicts were resolved through a discussion with a third researcher (JK). This stage resulted in the exclusion of *n* = 4,857 records, followed by an eligibility assessment (by MB and DW) of *n* = 97 full-text. In the final step, 32 articles were included in the systematic review.

To validate the results of the manual screening process further, *n* = 2,123 records identified via the EBSCO platform were screened by one researcher (EKu) using the ASReview software [21]. The respective records (*n* = 2,123) were entered along with eight texts (examples of relevant and irrelevant publications) used to train the AI model in ASReview (see Additional file 1 S1). The search stopped after 100 consecutive irrelevant articles were identified [22]. The number of relevant articles identified was *n* = 33, all of which (100%) overlapped with the *n* = 98 articles identified by manual searches (Fig. 1).

### Inclusion and exclusion criteria

The reviews were included if they met the following criteria: (1) applied methods of systematic reviews [19], scoping reviews [23], or pragmatic reviews [24]; (2) summarized original trials reporting the effectiveness of behavioral interventions using strategies such as BCTs included in the taxonomy proposed by Michie et al. [5]; (3) reviewed results of randomized controlled trials (RCTs), controlled trials (CTs), or pre-post observational studies with an intervention, but no control conditions; (4) applied outcomes such as, physical activity, healthy nutrition behaviour, or body weight indicators; (5) addressed women with breast cancer or breast cancer survivors. In cases where the paper encompassed populations with various cancer types, reviews were included only if ≥ 50% of the analyzed samples consisted of women with/survivors of breast cancer; and (6) were published in peer-reviewed English-language journals.

The following types of publications were excluded: (1) study protocols, conference materials, dissertations, and book chapters; (2) papers reporting original results of intervention trials; (3) reviews that did not provide evidence of associations between BCTs and behavioral or weight outcomes, but focused on outcomes such as quality of life or well-being; (4) reviews of qualitative studies; (5) reviews discussing behavioral interventions but reporting no details that would allow identification of the BCTs (e.g., “psychoeducation,” or “peer support groups”).

### Data extraction and coding

Data extraction and coding were conducted by pairs of researchers (MB, DW, JK, or KZL). Any disagreements were resolved through a consensus [19], involving a discussion with a third researcher (AL).

The extracted data included the following: (1) descriptive information (e.g., he number and design of original studies and country of origin); (2) information about populations studied (e.g., the number of participants, their sociodemographic characteristics, cancer stage, and type of treatment); and (3) type of the indicators and measurements applied to assess behavioral/body weight outcomes (e.g., for physical activity: minutes of total physical activity per week, step count, and energy expenditure; for healthy eating: amount of fruit and vegetable intake, fat and protein intake, and total caloric intake; for body weight: Body Mass Index (BMI) and body weight in kg); (4) the BCTs applied, in particular their names based on the BCT taxonomy by Michie et al. [5], and their content; (5) information about the effectiveness of the intervention (e.g., significance levels for the Time x Group interactions).

Information on the use of behaviour change techniques was extracted from the data provided in each included review. Each original study was coded for the presence or absence of application of each BCT described in the BCT taxonomy [5]. If a description of a BCT was provided but the name of the technique was missing, two coders matched its definitions and names to those in the BCT taxonomy [5]. In cases where only a general description of the applied techniques was provided (e.g., “physical activity education”), the description was not coded as a specific BCT. Each intervention discussed in the original research reported in the included systematic reviews was coded for the presence of any potential BCT combinations (with ≥ 3 BCTs; the order of BCTs did not matter) used in the intervention.

Next, each intervention was coded as ‘effective’ if there was a significant change over time in the intervention group for a respective type of outcome (e.g., physical activity), obtained for at least one indicator of this outcome (e.g., minutes of moderate-to-vigorous physical activity per day/week, metabolic equivalent of task, step count, or energy expenditure). An interventions was also coded as ‘effective’ if there was a significant difference between the intervention and control groups at the follow-up or if there was a significant Time x Group interaction (for CTs and RCTs). If several follow-ups were reported, the intervention was coded as effective if significant results were observed at one or more follow-ups. Importantly, the coding was based on data from the reviews and their supplementary materials (we did not search across publications reporting original results of the trials). In cases where one original intervention study was analyzed across several reviews included in this meta-review, the study was coded and subsequently analyzed only once.

Information regarding effectiveness was coded in one direction. A positive effect was assigned if a significant improvement in outcomes (such as increased physical activity, healthier nutrition behavior, and maintenance of healthy body weight) was reported in the included review. If a significant effect was not reported or if outcomes worsened (e.g., no change/less healthy nutrition), the intervention was coded as ineffective.

### Identifying BCT combinations and the analytical strategy for BCT combinations

We have developed an initial BCTs x Intervention effectiveness dataset (a CSV file) using the data coded in the original studies. This dataset includes all original studies represented as columns and rows, including the following information: (1) for each of the 93 BCTs from the BCT taxonomy [5], an indication of the absence/presence of the respective technique across all original intervention studies; (2) an absence/presence of an analysis reporting the effectiveness of an intervention across each of the three types of outcomes (i.e., physical activity, healthy nutrition behavior, or healthy body weight outcomes) in each intervention study; and (3) if an analysis was present for the respective type of outcome, the information whether the findings indicated a significant beneficial effect of the intervention. All duplicates of original studies were removed from the obtained dataset.

Next, analytical codes were developed in Python (Python 3.13.5; for codes see Additional file 1 S2). The Python codes function as an exploratory (descriptive) tool. The generated outputs provide a list of all potential combinations of ≥ BCTs used across the included original studies. For each identified BCT combination and each outcome domain, the output displays the total number of studies featuring that combination, as well as the percentage of those studies that yielded statistically significant beneficial effect for the respective outcome. However, the codes do not account for potential sources of heterogeneity (e.g., the unequal number of studies in which a respective BCT combination was used).

The codes allow to identify all combinations of  ≥ 3 BCTs that were included in behavioral interventions reported in ≥ 1 original study reporting the effectiveness of this intervention for  ≥ 1 outcomes (physical activity, healthy nutrition behaviour, or healthy body weight). For example, an intervention including 4 specific BCTs for one type of outcome could be included into *k* = 5 combinations (*k* = 4 3-element and *k* = 1 4-element combinations). In turn, an intervention including 5 specific BCTs could be included in *k* = 16 combinations (*k* = 10 3-element, *k* = 5 4-element combinations and *k* = 1 5-element combination), an intervention including 6 specific BCTs could be included in *k* = 42 combinations (k = 20 3-element, *k* = 15 4-element, *k* = 6 5-element and *k* = 1 6-element combination), etc.

The Python codes transformed the initial dataset into the final BCT combinations dataset, including: (1) all BCT combinations identified in the initial dataset, containing a set of ≥ 3 BCTs (the order of the BCTs was irrelevant); (2) the number of intervention studies that included a respective BCT combination;, (3) the number of studies that both addressed a respective BCT combination and used the respective outcome (physical activity, or healthy nutrition behaviour, or healthy body weight); (4) the number of studies that both addressed a respective BCT combination and yielded a beneficial effect for each of these outcomes; (5) the number of studies that both addressed a respective BCT combination and indicated that there were no beneficial effects for each of the three outcomes; and (6) the percentage of effective studies for a given BCT combination, with each outcome type serving as the primary unit of analysis.

Percentages of studies indicating beneficial effects across the three types of outcomes were used as indicators of effectiveness for each BCT combination. For example, if a given BCT combination was included in 44 studies on physical activity, 34 of which were coded as effective and 10 as ineffective, then the effectiveness indicator for this BCT combination in the domain of physical activity would be 77% ([34 × 100]/44). BCT combinations with an effectiveness indicator below 60% were deemed insufficiently supported and were excluded from further analysis. This 60% threshold aligns with the benchmarks established in prior systematic reviews [25, 26].

Finally, BCT combinations were considered supported across the three types of outcomes if they met the following criteria: (1) the combination was included in ≥ 10 intervention studies and (2) there were ≥ 3 intervention studies evaluating the effectiveness for each outcome type.

The Python codes and the dataset used in our analyses are available in an open science repository, ZENODO (see https://zenodo.org/records/21129562).

### Risk of bias, data analysis methods

The assessment was conducted using the Risk of Bias in Systematic Reviews (ROBIS) tool, developed to evaluate the risk of bias in systematic reviews [27]. ROBIS accounts for potential bias in the following domains: (1) eligibility criteria for original studies; (2) the process of identification and selection of original studies; (3) data collection and study appraisal; (4) data synthesis strategy and reporting of results; and (5) the overall risk of bias. Thresholds for categorizing reviews as having low, moderate, or high risk of bias were defined in line with the guidelines proposed by Whiting et al. [27].

## Results

### Descriptive findings

Details of the identified reviews – specifically, the number of original studies, their designs, sample sizes, population characteristics, geographic locations, and outcome types – are reported in Table 1.

**Table 1:** Descriptive results for included reviews

No	Reference	Number of original studies included	Number of persons	Region/Country	Design of research	Indicators of outcomes	Inclusion criteria
1	Amireault et al., 2016 [28]	16	*N* = 3,308	USA, Canada, UK, Australia, Ireland, South Korea	RCTs	Physical activity, Nutrition,	- RCTs - Intervention on healthy eating and physical activity behaviours among cancer survivors - Cancer survivors (an individual from the time of the diagnosis until the end of life) - Age: any
2	Brunet et al., 2020 [29]	5	*N* = 683	USA, Australia	RCTs	Physical activity	- Age ≥ 18 years - Cancer survivors
3	Cooper et al., 2023 [8]	20	*N* = 1,726	USA, South Korea, Australia, Germany, Italy, Netherlands, Japan	RCTs, pre-test-post test, Q-R multicenter, experiment, prospective intervention	Physical activity	- Age ≥ 18 years - Woman diagnosed with BC (in treatment or completed treatment)
4	Finne et al., 2018 [30]	20	*N* = 4,314	No data	RCTs	Physical activity	- Age ≥ 18 years - Cancer diagnosis after completion of the main treatment - Exclusion: current active treatment (except hormonal treatment) or end-of-life patients
5	Gan et al., 2022a [31]	4	*N* = 3,802	USA, Spain, Germany	RCTs	Nutrition	- Age ≥ 18 years - Cancer diagnosis with any type of cancer, had completed the primary treatments - Patients with local cancer recurrence or distant metastasis were excluded
6	Gan et al., 2022b [32]	1	*N* = 492	Spain	RCTs	Nutrition	- Age ≥ 18 years - Cancer diagnosis - Patients with local cancer recurrence or distant metastasis were excluded
7	Goode et al., 2015 [33]	12	*N* = 2,494	USA, Canada	RCTs	Physical activity Nutrition Body weight/composition	- Age ≥ 18 years - Cancer patients (completed or are undergoing treatment)
8	Grimmett et al., 2019 [34]	14	*N* = 3,375	USA, Canada, Netherlands, Australia, Germany	RCTs	Physical activity	- Age ≥ 18 years - Cancer diagnosis
9	Haberlin et al., 2018 [35]	2	*N* = 406	South Korea	pilot RCT, quasi RCT	Physical activity	- Experimental studies (RCTs, pre-post design, quasi-experimental) and observational studies, with or without controls - Evaluated an eHealth-based intervention (Internet and mobile technologies) delivered to cancer survivors and included PA as a primary or secondary outcome measure
10	Hailey et al., 2022 [10]	27	*N* = 3,656	USA, Europe, Australia, Canada, South Korea	RCTs, Quasi-experimental	Physical activity	- Women diagnosed with BC - Completed treatment, except hormonal therapy within the past 5 years
11	Hoedjes et al., 2017 [56]	6	*N* = 1,593	USA	RCTs, wait-list control	Body weight/composition	- Age ≥ 18 years - BMI ≥ 25 - Cancer survivors - Only interventions applied after initial treatment (i.e., surgery, chemotherapy, and radiotherapy) - Hormonal therapy was not considered to be the initial treatment
12	Ibeggazene et al., 2021 [36]	2	*N* = 141	No data	RCTs	Physical activity	- Age ≥ 18 years - Cancer diagnosis - Physically inactive or had a sedentary lifestyle at baseline
13	IJsbrandy et al., 2018 [37]	6	*N* = 725	USA, Canada, New Zealand	RCTs	Physical activity	- Age ≥ 18 years - Cancer diagnosis
14	Kiss et al., 2019 [38]	8	*N* = 1,119	USA, South Korea, Canada, Spain	RCTs	Physical activity Nutrition	- Age ≥ 18 years - Cancer diagnosis (with any type of cancer at any stage prior to, during, or after cancer treatment, including cancer survivors, and received any treatment modality)
15	Knols et al., 2010 [39]	4	*N* = 529	USA, Canada, Switzerland	RCTs	Physical activity	- RCTs, examining the results of a physical activity intervention on daily walking activity in cancer patients and cancer survivors
16	Ning et al., 2022 [40]	6	*N* = 854	USA, Australia, South Korea, Netherlands	RCTs	Physical activity	- RCTs - Age ≥ 18 years - Cancer diagnosis (with any type of cancer at any stage prior to, during, or after cancer treatment, including cancer survivors, and received any treatment modality)
17	Perperidi et al., 2023 [9]	11	*N* = 2,136	USA, Australia, Canada	RCTs	Physical activity	- Age ≥ 18 years - BMI ≥ 25 kg/m2 - Women diagnosed with BC, having completed overall treatment, including surgery, chemotherapy, radiotherapy, and other cancer treatments, except of hormonal or immune therapy
18	Playdon et al., 2013 [41]	6	*N* = 519	USA, Canada	RCTs, single cohort experimental intervention studies, controlled, non-randomized trial	Body weight/composition	- Age ≥ 18 years - Woman diagnosed with BC
19	Pudkasam et al., 2021 [20]	16	*N* = 1,668	USA, Australia, Canada, UK	RCTs	Physical activity	- Age ≥ 18 years - Women diagnosed with BC having completed primary treatments (surgery, chemotherapy and radiotherapy) at least three months prior to recruitment and had no evidence of cancer recurrence
20	Quadflieg et al., 2024 [42]	5	*N* = 328	USA, Europe, Asia,	RCTs	Physical activity	- Age ≥ 18 years - Within 12 months from cancer diagnosis
21	Roberts et al., 2017 [43]	8	*N* = 503	USA, UK, Canada, Australia, Netherlands, South Korea	RCTs, pre-post comparison studies	Physical activity Body weight/composition	- Age ≥ 18 years - Cancer diagnosis (with any type of cancer)
22	Rodrigues et al., 2023 [44]	18	*N* = 2,958	No data	RCTs	Physical activity Nutrition	- Age ≥ 18 years - Cancer diagnosis (with any type of cancer at any point from diagnosis and stage of disease/treatment)
23	Salisbury et al., 2023 [45]	14	*N* = 2,880	No data	RCTs	Physical activity	- Age ≥ 18 years - With a histologically confirmed diagnosis of cancer (including all stages of, and treatments for cancer)
24	Seven et al., 2023 [46]	17	*N* = 3,443	USA, Australia, New Zealand	RCTs, Pre-post-test study	Physical activity Nutrition Body weight/composition	- Aged ≥ 18 years - Cancer diagnosis
25	Short et al., 2013 [47]	9	*N* = 1,231	No data	Individual or clustered RCTs	Physical activity	- Aged ≥ 18 years - Post-treatment (not including hormone therapy) breast cancer survivors
26	Singh et al., 2022 [48]	26	*N* = 3,255	No data	RCTs	Physical activity	- RCTs evaluated PA intervention participants - Age: any - Cancer diagnosis at any stage of treatment (pre-treatment, undergoing treatment, or post-treatment) - Studies that involved samples with multiple cancer types
27	Sremanakova et al., 2021 [12]	13	*N* = 5,102	USA, South Korea, Australia, UK, Netherlands	RCTs	Nutrition	- Age ≥ 18 years - Cancer survivors who completed surgery and all anticancer treatments
28	Stacey et al., 2015 [49]	13	*N* = 1,634	USA, Australia, Taiwan,	RCTs	Physical activity Nutrition Body weight/composition	- Age ≥ 18 years - Cancer diagnosis (with any cancer at any point from diagnosis) - Trials focused on PA only: participants with completed active cancer treatment (excluding hormone treatment)
29	Swartz et al., 2017 [50]	7	*N* = 560	USA, Australia, Norway, South Korea	RCTs	Physical activity	- Age ≥ 18 years - Cancer diagnosis
30	Takemura et al., 2024 [51]	7	*N* = 404	USA, Australia, UK, New Zealand, Luxemburg	RCTs	Physical activity Body weight/composition	- Age ≥ 18 years, - Cancer diagnosis with any type and stage of cancer and at any cancer treatment trajectory (before, during, or after treatment)
31	Teo et al., 2023 [52]	16	*N* = 1,690	USA, Australia, Canada Denmark	RCTs	Physical activity Body weight/composition	- Age ≥ 18 years - RCTs evaluating the effects of wearable-technology-assisted interventions among breast cancer survivors
32	Turner et al., 2018 [53]	6	*N* = 512	No data	RCTs	Physical activity	- Age ≥ 18 years - Sedentary people with a homogenous primary cancer diagnosis

*PA* physical activity, *BC* women with breast cancer or survivors, *RCT* Randomized Controlled Trials, *Q-RCT* Quasi Randomized Controlled Trials

Across the 32 included reviews describing a total of 568 studies (*N* = 89,585 participants), data from 345 original studies (*N* = 58,040 participants) were retained. The original studies that were excluded either did not provide sufficient data to code BCTs or comprised samples in which more than 50% of participants had cancers other than breast cancer. Next, we coded all 345 eligible original studies and checked whether any original study had been included in more than one review, removing all duplicate instances. When the same studies were described in several reviews and differed in the listed techniques, we created a single version of the study description, adopting the most comprehensive one. These steps resulted in *n* = 179 original studies being included in subsequent analyses.

Across these selected original studies, 157 (88%) reported data on physical activity outcomes, 52 (29%) reported on nutrition-related outcomes, and 32 (18%) reported on body weight. Most of the original studies (92%) were randomized controlled trials. The remaining studies comprised pre–post designs (4%), non-randomized controlled trials (2%), prospective intervention studies (1%), and studies employing a wait-list control design (1%).

The behavioral interventions reported in the studies included in the analyzed reviews utilized 69 BCTs out of the 93 proposed by Michie et al. [5]. Details on the coding of individual BCTs and their combinations in each study are reported in Additional file 1 Table S3.

The risk of bias across the 5 domains of the ROBIS tool is presented in the Additional file 1 Table S2. Across 14 out of 32 reviews, the risk of bias was low across five domains of the ROBIS tool.

Among the 179 original studies retained for the analysis, 63 (35.3%) were retrieved from systematic reviews that uniformly presented a high risk of bias, whereas 42 (23.4%) came from reviews that were consistently rated as having a low risk of bias. The remaining 74 studies (41.3%) were retrieved from at least two systematic reviews and featured a mix of both low- and high-risk assessments according to ROBIS. In case the original study was identified in at least two reviews of which at least one was of low risk, the data for coding the original study was retrieved from the low-risk review. For the top seven BCT combinations (confirmed in ≥ 90% of original studies; see Table 2), the proportions of original studies retrieved from low-risk reviews were as follows: 50% (1st combination), 60% (2nd), 57% (3rd), 75% (4th), 58% (5th), 67% (6th), and 75% (7th combination). This indicates that the majority of the data were retrieved from reviews with a low risk of bias.

**Table 2:** The most effective BCT combinations in the studied interventions

No	Total number of studies	BCT #1	BCT # 2	BCT# 3	BCT# 4	BCT #5	BCT #6	Physical activity: effectiveness [%]	Nutrition behaviour: effectiveness [%]	Healthy body weight maintenance: effectiveness [%]
1	16	Goal setting (behaviour)	Problem solving	Demonstration of the behaviour				93	100	100
2	15	Problem solving	Self -monitoring of behaviour	Demonstration of the behaviour				92	100	100
3	14	Goal setting (behaviour)	Problem solving	Self-monitoring of behaviour	Demonstration of the behaviour			92	100	100
4	12	Problem solving	Self -monitoring of behaviour	Instruction on how to perform the behaviour	Demonstration of the behaviour			90	100	100
5	12	Problem solving	Feedback on behaviour	Demonstration of the behaviour				90	100	100
6	12	Self -monitoring of behaviour	Demonstration of the behaviour	Credible source				90	100	100
7	12	Goal setting (behaviour)	Problem solving	Instruction on how to perform the behaviour	Demonstration of the behaviour			90	100	100
8	11	Feedback on behaviour	Demonstration of the behaviour	Credible source				89	100	100
9	11	Problem solving	Feedback on behaviour	Self- monitoring of behaviour	Demonstration of the behaviour			89	100	100
10	11	Goal setting (behaviour)	Problem solving	Feedback on behaviour	Demonstration of the behaviour			89	100	100
11	11	Goal setting (behaviour)	Problem solving	Self-monitoring of behaviour	Instruction on how to perform the behaviour	Demonstration of the behaviour		89	100	100
12	11	Self- monitoring of behaviour	Instruction on how to perform the behaviour	Demonstration of the behaviour	Credible source			89	100	100
13	11	Problem solving	Self- monitoring of behaviour	Instruction on how to perform the behaviour	Demonstration of the behaviour	Graded tasks		89	100	100
14	11	Goal setting (behaviour)	Self-monitoring of behaviour	Demonstration of the behaviour	Credible source			89	100	100
15	11	Problem solving	Demonstration of the behaviour	Graded tasks				89	100	100
16	11	Problem solving	Instruction on how to perform the behaviour	Demonstration of the behaviour	Graded tasks			89	100	100
17	11	Goal setting (behaviour)	Problem solving	Goal setting (outcome)				89	100	100
18	11	Problem solving	Feedback on behaviour	Instruction on how to perform the behaviour	Demonstration of the behaviour			89	100	100
19	11	Problem solving	Self- monitoring of behaviour	Demonstration of the behaviour	Graded tasks			89	100	100
20	10	Goal setting (behaviour)	Social support	Demonstration of the behaviour	Credible source			89	100	100
21	18	Problem solving	Self- monitoring of behaviour	Graded tasks				88	100	100
22	17	Self- monitoring of behaviour	social support	Graded tasks				88	100	100
23	10	Problem solving	Feedback on behaviour	Instruction on how to perform the behaviour	Demonstration of the behaviour	Graded tasks		88	100	100
24	10	Goal setting (behaviour)	Problem solving	Instruction on how to perform the behaviour	Demonstration of the behaviour	Graded tasks		88	100	100
25	10	Goal setting (behaviour)	Problem solving	Feedback on behaviour	Instruction on how to perform the behaviour	Demonstration of the behaviour		88	100	100
26	10	Goal setting (behaviour)	Problem solving	Self-monitoring of behaviour	Demonstration of the behaviour	Graded tasks		88	100	100
27	10	Instruction on how to perform the behaviour	Graded tasks	Credible source				88	100	100
28	10	Self -monitoring of behaviour	Graded tasks	Credible source				88	100	100
29	10	Goal setting (behaviour)	Problem solving	Feedback on behaviour	Self-monitoring of behaviour	Demonstration of the behaviour		88	100	100
30	10	Goal setting (behaviour)	Problem solving	Goal setting (outcome)	Feedback on behaviour			88	100	100
31	10	Goal setting (behaviour)	Problem solving	Goal setting (outcome)	Self-monitoring of behaviour			88	100	100
32	10	Goal setting (behaviour)	Problem solving	Demonstration of the behaviour	Graded tasks			88	100	100
33	10	Goal setting (behaviour)	Problem solving	Self -monitoring of behaviour	Instruction on how to perform the behaviour	Demonstration of the behaviour	Graded tasks	88	100	100
34	10	Feedback on behaviour	Self- monitoring of behaviour	Demonstration of the behaviour	Credible source			88	100	100
35	10	Self- monitoring of behaviour	Instruction on how to perform the behaviour	Graded tasks	Credible source			88	100	100
36	10	Problem solving	Feedback on behaviour	Self- monitoring of behaviour	Instruction on how to perform the behaviour	Demonstration of the behaviour	Graded tasks	88	100	100
37	10	Feedback on behaviour	Instruction on how to perform the behaviour	Demonstration of the behaviour	Credible source			88	100	100
38	10	Problem solving	Feedback on behaviour	Self- monitoring of behaviour	Instruction on how to perform the behaviour	Demonstration of the behaviour		88	100	100
39	10	Problem solving	Feedback on behaviour	Self- monitoring of behaviour	Demonstration of the behaviour	Graded tasks		88	100	100
40	10	Problem solving	Feedback on behaviour	Demonstration of the behaviour	Graded tasks			88	100	100
41	10	Goal setting (behaviour)	Self-monitoring of behaviour	Instruction on how to perform the behaviour	Demonstration of the behaviour	Credible source		88	100	100
42	17	Goal setting (behaviour)	Problem solving	Graded tasks				87	100	100
43	17	Goal setting (behaviour)	Problem solving	Self- monitoring of behaviour	Graded tasks			87	100	100
44	16	Goal setting (behaviour)	Social support	Graded tasks				87	100	100
45	16	Goal setting (behaviour)	Self- monitoring of behaviour	Social support	Graded tasks			87	100	100
46	38	Goal setting (behaviour)	Problem solving	Self- monitoring of behaviour				86	92	100

*BCT* Behavior change technique*, PA* physical activity, BCT 1, 2…− 6 – BCTs included in a given combination; PA effectiveness [%] – % effectiveness of studies for the physical activity outcome (calculated as the proportion of effective to ineffective studies), Nutrition behaviour effectiveness [%] – % effectiveness of studies for the healthy nutrition behavior outcome (calculated as the proportion of effective to ineffective studies), Healthy body weight maintenance effectiveness [%] – % effectiveness of studies for the healthy body weight outcome (calculated as the proportion of effective to ineffective studies)

### BCT combinations identified in effective interventions

The final BCT combination dataset yielded *k* = 17,715 combinations, containing 3–12 BCTs. See Additional file 2 for details. No study reported a combination with ≥ 13 BCTs.

In case of *k* = 8,970 (50.7%) BCT combinations, only sparse evidence was obtained; that is, either *n* ≤ 2 intervention studies reported using these combinations, and/or < 60% of the studies addressed the respective combinations reported beneficial effects for any of the analyzed outcomes. Additional file 2 includes all BCT combinations, along with information on the number of original intervention studies addressing each combination across different outcomes, as well as the percentage of studies supporting each BCT combination for each outcome.

Across the remaining *k* = 8,735 (49.7%) BCT combinations, only *k* = 46 combinations were included in *n* ≥ 10 original studies (with ≥ 3 studies for each outcome). Of these, ≥ 85% of studies reported beneficial effects across all three outcome domains (see Table 2). In other words, we identified 46 BCT combinations for which research addressing physical activity, healthy nutrition behavior, and healthy body weight was conducted. Within each of these three outcome domains, ≥ 85% of the original studies supported the effectiveness of the respective combination. Assuming the same criteria referring to the number of studies needed to demonstrate consistency (i.e., ≥ 10 studies total and ≥ 3 studies per outcome) but with a threshold of ≥ 90% of studies indicating effectiveness in each of the outcome domains, only seven BCT combinations were identified as consistently supported (see Table 2).

The BCT combination with the highest indicators of support included three BCTs: (1) goal setting (behaviour) (BCT#1.1), problem solving (BCT#1.2), and demonstration of the behaviour (BCT#6.1) (combination #1; Table 2). This BCT combination was found in interventions that were beneficial in 93% of studies addressing physical activity outcomes, 100% of studies addressing nutrition outcomes, and 100% of studies addressing body weight-related outcomes. The following two BCT combinations were beneficial in 92% of studies assesing physical activity outcomes, and 100% of studies assesing nutrition behaviour and body weight-related outcomes. These combinations included: (2) problem solving (BCT#1.2), self-monitoring of behaviour (BCT#2.3), and demonstration of the behaviour (BCT#6.1) and (3) goal setting (behaviour) (BCT#1.1), problem solving (BCT#1.2), self-monitoring of behaviour (BCT#2.3), and demonstration of the behaviour (BCT#6.1). Finally, support across ≥ 90% of studies addressing physical activity and 100% of studies addressing nutrition behaviour as well as body weight-related outcomes was found for four types of BCT combinations: (4) problem solving (BCT#1.2), self-monitoring of behaviour (BCT#2.3), instruction on how to perform the behaviour (BCT#4.1), and demonstration of the behaviour (BCT#6.1); (5) problem solving (BCT#1.2), feedback on behaviour (BCT#2.2), and demonstration of the behaviour (BCT#6.1); (6) self-monitoring of behaviour (BCT#2.3), demonstration of the behaviour (BCT#6.1), and a credible source (BCT#9.1); and (7) goal setting (behaviour) (BCT#1.1), problem solving (BCT#1.2), instruction on how to perform the behaviour (BCT#4.1), and demonstration of the behaviour (BCT#6.1). See Table 2 for details. Considering the composition of these top seven combinations, all combinations included demonstration of the behaviour, 6 out of 7 included problem solving, and 4 out of 7 included self-monitoring of behaviour. These were the most consistently used BCTs.

The remaining *k* = 39 supported BCT combinations were supported in 85–89% of studies addressing all three outcome domains (Table 2). The lowest percentage of supported studies was generally found for the physical activity outcome domain. This outcome domain was the most represented, with the highest number of original studies (between 7 and 31) addressing this outcome (see Additional file 1 Table S3), whereas nutrition outcomes were addressed in 3–12 original studies, and weight-related outcomes were addressed in only 3–6 studies. In terms of the composition of the 39 supported BCT combinations, they included the same 7 BCTs that were most consistently represented in BCT combinations supported by ≥ 90% of studies, along with 3 other BCTs.

Overall, the BCTs included in all 46 supported combinations were limited to the following 10 techniques: (1) demonstration of the behaviour (BCT#6.1), included in 33 combinations; (2) problem solving (BCT#1.2), included in 32 combinations; (3) self-monitoring of behaviour (BCT#2.3), included in 26 combinations; (4) goal setting for behaviour (BCT#1.1), included in 22 combinations; (5) graded task (BCT#8.7), included in 21 combinations; (6) instruction on how to perform the behaviour (BCT#4.1), included in 16 combinations; (7) feedback on behaviour (BCT#2.2), included in 15 combinations; (8) credible source (BCT#9.1), included in 11 combinations; (9) social support (BCT#3.1), included in 4 combinations; and (10) goal setting for the outcome (BCT#1.3), included in 3 combinations. More than half (24 out of 46) of the supported BCT combinations did not include either goal setting for behaviour or goal setting for the outcome.

## Discussion

**Fig. 2:**
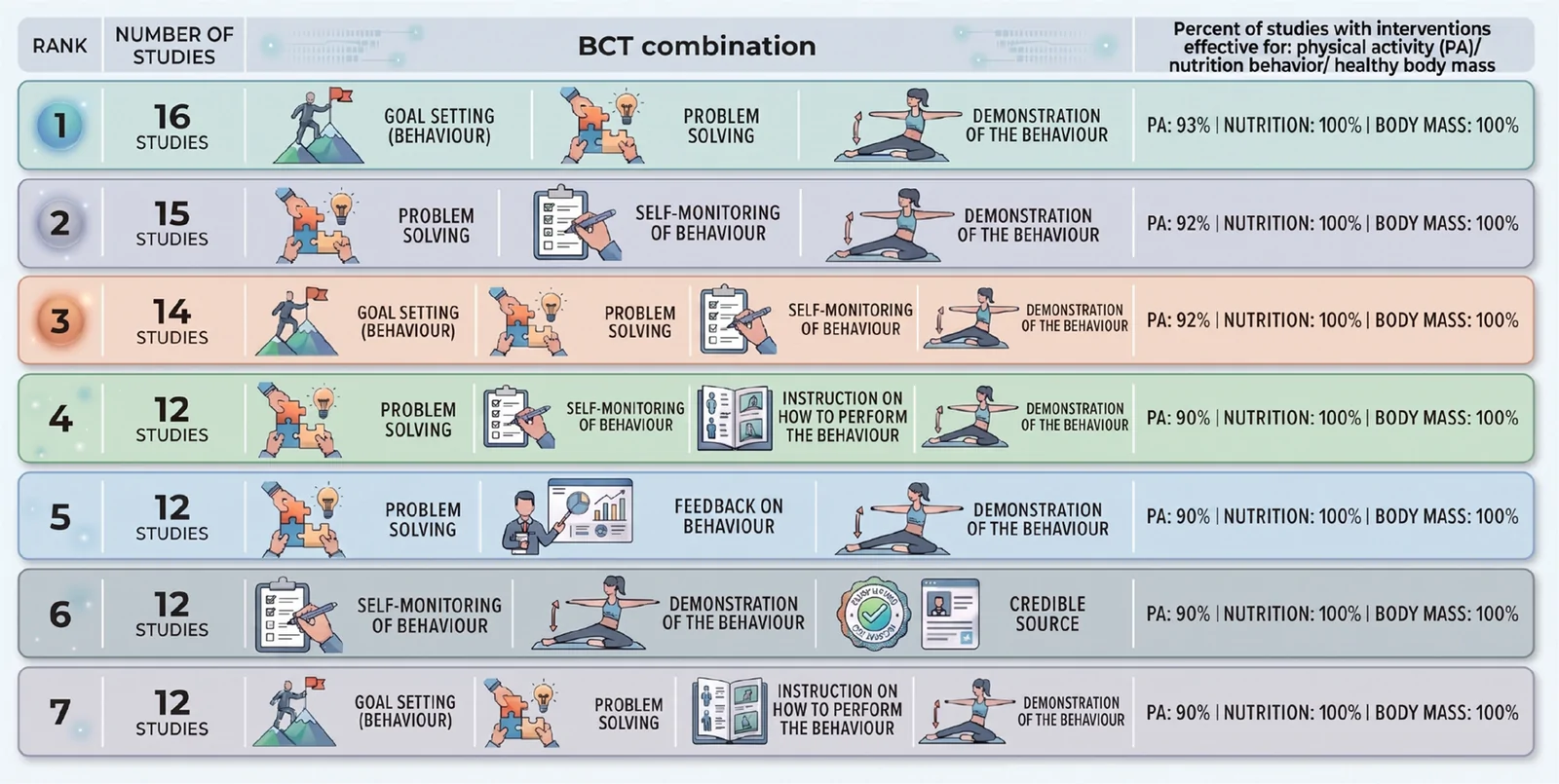
BCT combinations supported across ≥ 90% of lifestyle intervention studies conducted among women with breast cancer or breast cancer survivors

Our meta-review is among the first investigating the use of BCT combinations (defined as a sequence of ≥ 3 techniques) in effective behavioral interventions. We identified 46 combinations of 3–6 BCTs that were used in interventions that benefited physical activity, nutrition behaviour, and healthy body weight maintenance among women with breast cancer and breast cancer survivors. Of these 46 combinations, 7 were supported across ≥ 90% of the original studies, accounting across all three health outcomes (see Fig. 2). The remaining 39 combinations were supported across ≥ 85% of the original studies. However, it should be noted that the results and conclusions of this review are observational rather than based on strong evidence of superiority. For instance, the data do not reflect clinically meaningful and significant differences obtained from trials directly comparing one BCT combination with another.

Identifying BCT combinations that are characteristic of effective interventions is the first step in bridging the gap between taxonomy-guided research and clinical practice. This study identified empirical combinations of up to six BCTs (e.g., instruction on how to perform the behaviour, demonstration of the behaviour, graded tasks, problem solving, feedback on behaviour, self-monitoring of behaviour) that may serve as evidence-based building blocks across three behavioral and weight-related outcomes. While prior reviews evaluated BCTs in isolation—resulting in 'hypothetical' combinations that could have never been actually co-implemented or tested in primary studies—our approach verifies combinations that were genuinely co-delivered. Consequently, these findings offer preliminary guidance for designing complex BCT-based lifestyle interventions for breast cancer patients and survivors.

Most BCTs within the supported combinations derive from major social cognition models, primarily control/self-regulation theory and the health action process approach model. These theories emphasize conscious self-regulatory processes, such as goal setting, self-monitoring of behaviour, problem-solving, and graded tasks, alongside social support [54]. Additionally, the inclusion of instruction and demonstration aligns with social cognitive theory, which underscores the fundamental role of behavioral practice [54, 55].

One interesting finding concerns the content of the supported combinations. First, goal setting (referring to the behaviour or the outcome) was included in only 22 out of 46 supported BCT combinations. Prior reviews of healthy lifestyle interventions for women with breast cancer or survivors of breast cancer consistently identify goal setting (outcome) among the most supported BCTs [9, 10, 34, 52, 56]. Although the present review corroborates the value of goal setting, this technique did not rank among the three most frequently utilized BCTs within the effective interventions. Goal setting, or formulating behavioral intentions, is a cornerstone of many health behaviour change theories, such as the theory of planned behaviour [57], the social cognitive theory [55], the health action process approach [58], and the control/self-regulation theory [59], etc. Grounded in these theories, goal setting may be considered an indispensable BCT. However, specific populations, such as women with breast cancer or survivors of breast cancer—particularly those who actively enroll in lifestyle interventions—may already possess high baseline motivation and self-imposed goals, rendering the explicit inclusion of goal-setting BCTs redundant.

The composition of the supported BCT combinations must be evaluated in the context of the unique challenges faced by women with breast cancer and survivors of breast cancer. Many of these women experience treatment-related or post-treatment fatigue, a decline in physical fitness [60], and need to adopt new exercise routines (e.g., related to rehabilitation). These issues may result in an emphasis on such BCTs as instruction and demonstration of the behaviour. Survivors may experience disruptions to work routines, lower work performance, fear of recurrence, and mood changes [61, 62]. Furthermore, breast cancer treatments can impair cognitive functions, such as attention and memory [63]. These issues contribute to both impairment of the cognitive capacity for self-regulation, as well as an above-average burden of self-regulation due to changes in daily routines, emotional functioning, and the need to integrate new demanding tasks. It seems plausible to assume that under such circumstances, adoption and maintenance of regular exercise, a healthy diet, and maintenance of healthy body weight may require substantial support through an intervention that enhances self-regulatory competence by means of BCTs that prompt self-monitoring, problem-solving, and graded tasks. The impairment of attention and memory [63] may also increase the usefulness of BCTs that focus on instruction and demonstration.

One of the BCTs included in the supported BCT combinations was a credible source. The use of this technique may be of particular relevance for women with/survivors of breast cancer. Patients and survivors may use large language model tools, which provide them with responses that do not follow the most current guidelines, contain incorrect information, provide inconsistent recommendations, and fail to account for regional variations in practice [64]. Qualitative research on patients and survivors’ perspectives suggests that source credibility is a critical priority for women with breast cancer, survivors, and treatment stakeholders when developing healthy lifestyle interventions [65].

## Limitations

There are several limitations to this meta-review and its findings that should be mentioned. First, the analytical strategy in this meta-review used arbitrarily proposed cut-off points for the inclusion of supported BCT combinations, such as a minimum of the total of *n* = 10 studies, ≥ 3 studies per outcome, and 85% of studies indicating beneficial changes. Modifying these cut-offs would result in a different yet overlapping pattern of results. The supported combinations were assumed to encompass as few as 3 BCTs. It remains unclear whether a mix of two 3-component interventions would result in similar or different effects (unless the respective 6 BCTs are also included in a supported 6-component BCT combinations). The applied meta-review design excludes the most recent studies, which were not discussed in the reviews included in this meta-review. The original studies differed in terms of the participants included, who had early to late stages of cancer, had undergone different treatments, and varied in terms of time elapsed since diagnosis. Additionally, 76% of the studies were conducted exclusively in patients with breast cancer, whereas the remaining 24% included individuals with various cancer types, including breast cancer. Using more specific samples (e.g., only young women with early stages of cancer who did not undergo radiation therapy or a radical mastectomy) could result in different findings. We investigated BCT combinations that resulted in consistent findings across 3 outcome domains. However, using only one outcome domain (e.g., physical activity) would yield different numbers and different content of supported combinations. The majority (92%) of the studies used the RCT design, but we did not control for the actual design and the risk of bias in the original studies. Across included reviews, only 14 of 32 had a low risk of bias across all domains included in the risk of bias ROBIS tool. Because the included reviews provided insufficient effect sizes or statistical data required to aggregate quantitative data, a meta-analysis was not feasible. Consequently, our conclusions are based on the presence of statistically significant findings rather than on a pooled estimate of average effects. The included reviews and primary studies vary substantially in terms of intervention format, duration, delivery mode, participant characteristics, and outcome measurement. This underlying heterogeneity limits definitive conclusions and generalizations, as the reported effectiveness may reflect complex interactions between BCT combinations and contextual factors (such as delivery mode or participant demographics). While future studies should employ meta-analytical methods to control for these sources of heterogeneity, meta-regression itself faces significant limitations [66]. To be effective, such analyses require individual participant datasets obtained from a substantial number of RCTs with published or registered protocols.

The implications for practice have some limitations. First, within the primary studies included in our review, certain BCTs and BCT combinations were researched far more frequently than others. Consequently, conclusions regarding effectiveness are constrained to those BCT combinations that were commonly featured in past interventions. While other effective BCT combinations likely exist, they have not yet been evaluated widely enough to draw definitive conclusions.

## Conclusions

Despite the limitations, our review has several strengths, such as the re-analysis of a large set of studies, a relatively robust methodology, and a relatively homogeneous population. This study is among the first to investigate multi-component BCT combinations and their use in effective interventions for improving physical activity and nutrition behaviour, as well as maintaining healthy body weight. We identified 46 combinations of 3–6 BCTs that may be used in health promotion practices targeting women with breast cancer and breast cancer survivors.

## Supplementary Information

Additional file 1: Description of data: Supplementary Table 1 – the list of keywords applied in the review; S1 – The examples of articles used for search strategy in ASReview; S2 – Python code used for BCT combinations; Supplementary Table S2 – details of risk of bias assessment of the included articles in the meta-review; Supplementary Table 3 – most effective BCT combinations.

Additional file 2: Final dataset of BCT combinations (k = 17,715) containing 3–12 BCTs per combination.

Additional file 3: Input data used for Python-based statistical calculations.

Additional file 4: PRISMA checklist.

## Data Availability

All data analyzed during this study are either secondary (retrieved from original studies included in the review) or included in this published article (and its supplementary information files). The dataset supporting the conclusions of this article is included within the article (and its additional file 3).

## Abbreviations

BCTs: Behaviour change techniques
BMI: Body mass index
RCTs: Randomized controlled trials
CT: Controlled trials
PRISMA: Preferred Reporting Items for Systematic Reviews and Meta-Analyses
PICO: Population-Intervention-Comparison-Outcome
ROBIS: Risk of Bias
