# Supplementary material for: Which combinations of behaviour change techniques work best? Evidence from meta-review of interventions addressing healthy diet and exercise after breast cancer
Source: Int J Behav Nutr Phys Act. 2026 Aug 4;23:104. doi: 10.1186/s12966-026-01961-6 (PMC13725973; doi:10.1186/s12966-026-01961-6)
Supplement: Supplementary file 1 [file 12966_2026_1961_MOESM2_ESM.pdf]

Name of Variables	Total number of studies	Number of effective studies for the PA	Number of effective studies for the Nutrition	Number of effective studies for the Body weight	Number of effective studies for the PA and Nutrition	Number of effective studies for the Nutrition and Body weight	Number of effective studies for the PA and Body weight	Number of effective studies for the PA, Nutrition and Body weight	Number of ineffective studies for the PA	Number of ineffective studies for the Nutrition	Number of ineffective studies for the Body weight	Number of ineffective studies for the PA and Nutrition	Number of ineffective studies for the Nutrition and Body weight	Number of ineffective studies for the PA and Weight Loss	Number of ineffective studies for the PA, Nutrition and Weight Loss	Number of studies lacking information on effectiveness for the PA	Number of studies lacking information on effectiveness for the Nutrition	Number of studies lacking information on effectiveness for the Body weight	Number of studies lacking information on effectiveness for the PA and Nutrition	Number of studies lacking information on effectiveness for the Nutrition and Body weight	Number of studies lacking information on effectiveness for the PA and Body weight	Number of studies lacking information on effectiveness for the PA, Nutrition and Body weight	Total number of studies for the Physical Activity outcome	Total number of studies for the Nutrition outcome	Total number of studies for the Body weight outcome	Total number of studies for the PA and Nutrition outcome	Total number of studies for the PA and Body weight outcome	Total number of studies for the Nutrition and Body weight outcome	Total number of studies for the PA, Nutrition and Body weight outcome	Technique 1	Technique 2	Technique 3	Technique 4
Phyton Code	Liczba badań	PA Effective	Dieta Effective	Weight Loss Effective	PA & Dieta Effective	Dieta & Weight Loss Effective	PA & Weight Loss Effective	PA & Dieta & Weight Loss Effective	PA Ineffective	Dieta Ineffective	Weight Loss Ineffective	PA & Dieta Ineffective	Dieta & Weight Loss Ineffective	PA & Weight Loss Ineffective	PA & Dieta & Weight Loss Ineffective	PA No Data	Dieta No Data	Weight Loss No Data	PA & Dieta No Data	Dieta & Weight Loss No Data	PA & Weight Loss No Data	PA & Dieta & Weight Loss No Data	Total PA	Total Dieta	Total Weight Loss	Total PA & Dieta	Total PA & Weight Loss	Total Dieta & Weight Loss	Total PA & Dieta & Weight Loss	Technika 1	Technika 2	Technika 3	Technika 4
	48	34	17	9	0	0	0	0	10	2	0	0	0	0	0	3	3	0	0	0	0	0	47	22	9	21	9	9	9	Goal setting (behaviour)	Feedback on behaviour	self monitoring of behaviour	
	40	23	10	6	0	0	0	0	13	2	0	0	0	0	0	3	3	0	0	0	0	0	39	15	6	14	6	6	6	Goal setting (behaviour)	social support	Instruction on how to perform the behaviour	
	39	25	9	9	0	0	0	0	9	1	0	0	0	0	0	4	4	0	0	0	0	0	38	14	9	14	8	7	7	Goal setting (behaviour)	self monitoring of behaviour	Instruction on how to perform the behaviour	
	38	31	12	6	0	0	0	0	5	1	0	0	0	0	0	2	2	0	0	0	0	0	38	15	6	15	6	6	6	Goal setting (behaviour)	Problem solving	self monitoring of behaviour	
	38	29	9	5	0	0	0	0	7	2	0	0	0	0	0	2	2	0	0	0	0	0	38	13	5	13	5	5	5	Goal setting (behaviour)	self monitoring of behaviour	social support	
	36	27	15	7	0	0	0	0	6	2	0	0	0	0	0	3	2	0	0	0	0	0	36	19	7	19	7	7	7	Goal setting (behaviour)	Problem solving	Feedback on behaviour	
	33	19	4	5	0	0	0	0	11	1	0	0	0	0	0	3	3	0	0	0	0	0	33	8	5	8	5	5	5	Goal setting (behaviour)	Action planning	social support	
	33	22	8	5	0	0	0	0	7	3	2	0	0	0	0	2	2	0	0	0	0	0	31	13	7	11	5	7	5	self monitoring of behaviour	social support	Instruction on how to perform the behaviour	
	33	22	10	4	0	0	0	0	9	3	0	0	0	0	0	2	1	0	0	0	0	0	33	14	4	14	4	4	4	Goal setting (behaviour)	Feedback on behaviour	social support	
	33	20	8	7	0	0	0	0	9	2	1	0	0	0	0	3	4	0	0	0	0	0	32	14	8	13	7	8	7	Feedback on behaviour	self monitoring of behaviour	Instruction on how to perform the behaviour	
	32	21	8	5	0	0	0	0	8	3	1	0	0	0	0	1	1	0	0	0	0	0	30	12	6	11	4	5	4	Feedback on behaviour	self monitoring of behaviour	social support	
	32	19	9	6	0	0	0	0	10	2	0	0	0	0	0	3	3	0	0	0	0	0	32	14	6	14	6	6	6	Goal setting (behaviour)	Feedback on behaviour	Instruction on how to perform the behaviour	
	32	25	11	7	0	0	0	0	4	2	1	0	0	0	0	2	3	0	0	0	0	0	31	16	8	15	7	8	7	Problem solving	Feedback on behaviour	self monitoring of behaviour	
	30	22	10	3	0	0	0	0	6	2	0	0	0	0	0	1	0	0	0	0	0	0	29	12	3	11	3	3	3	Goal setting (behaviour)	Problem solving	social support	
	30	14	3	6	0	0	0	0	12	1	0	0	0	0	0	4	4	0	0	0	0	0	30	8	6	8	6	6	6	Goal setting (behaviour)	Action planning	Instruction on how to perform the behaviour	
	30	20	10	5	0	0	0	0	7	2	0	0	0	0	0	2	2	0	0	0	0	0	29	14	5	13	5	5	5	Goal setting (behaviour)	Problem solving	Instruction on how to perform the behaviour	
	29	19	4	5	0	0	0	0	7	1	0	0	0	0	0	3	3	0	0	0	0	0	29	8	5	8	5	5	5	Goal setting (behaviour)	Action planning	self monitoring of behaviour	
	29	18	11	3	0	0	0	0	6	5	3	0	0	0	0	0	0	0	0	0	0	0	24	16	6	11	3	6	3	Problem solving	social support	Instruction on how to perform the behaviour	
	29	18	8	4	0	0	0	0	9	3	1	0	0	0	0	1	1	0	0	0	0	0	28	12	5	11	4	5	4	Feedback on behaviour	social support	Instruction on how to perform the behaviour	
	29	18	8	6	0	0	0	0	8	1	0	0	0	0	0	3	3	0	0	0	0	0	29	12	6	12	6	6	6	Goal setting (behaviour)	Feedback on behaviour	self monitoring of behaviour	Instruction on how to perform the behaviour
	29	23	11	6	0	0	0	0	4	1	0	0	0	0	0	2	2	0	0	0	0	0	29	14	6	14	6	6	6	Goal setting (behaviour)	Problem solving	Feedback on behaviour	self monitoring of behaviour
	28	14	2	5	0	0	0	0	10	2	1	0	0	0	0	3	3	0	0	0	0	0	27	7	6	6	5	6	5	Action planning	social support	Instruction on how to perform the behaviour	
	28	20	8	4	0	0	0	0	7	2	0	0	0	0	0	1	1	0	0	0	0	0	28	11	4	11	4	4	4	Goal setting (behaviour)	Feedback on behaviour	self monitoring of behaviour	social support
	28	19	8	6	0	0	0	0	5	3	2	0	0	0	0	2	3	0	0	0	0	0	26	14	8	12	6	8	6	Problem solving	self monitoring of behaviour	Instruction on how to perform the behaviour	
	28	20	8	5	0	0	0	0	6	1	0	0	0	0	0	2	2	0	0	0	0	0	28	11	5	11	5	5	5	Goal setting (behaviour)	self monitoring of behaviour	social support	Instruction on how to perform the behaviour
	27	14	8	7	0	0	0	0	8	2	0	0	0	0	0	4	4	0	0	0	0	0	26	14	7	13	7	7	7	Goal setting (behaviour)	Instruction on how to perform the behaviour	Credible source	

2	2	Problem solving	social support	Graded tasks	
2	2	Action planning	Feedback on behaviour	Prompts/cues	
2	2	Goal setting (behaviour)	Problem solving	social support	Graded tasks
2	2	Goal setting (behaviour)	Action planning	self monitoring of behaviour	Instruction on how to perform the behaviour
2	2	Goal setting (behaviour)	Feedback on behaviour	social support	Instruction on how to perform the behaviour
4	4	Goal setting (behaviour)	Action planning	self monitoring of behaviour	Instruction on how to perform the behaviour
5	5	self monitoring of behaviour	Demonstration of the behaviour	Credible source	
1	1	Goal setting (behaviour)	Problem solving	Feedback on behaviour	self monitoring of behaviour
5	5	Feedback on behaviour	self monitoring of behaviour	Instruction on how to perform the behaviour	Demonstration of the behaviour
1	1	Goal setting (behaviour)	self monitoring of behaviour	social support	Behavioural practice/rehearsal
3	3	Problem solving	Review of behaviour goals	Feedback on behaviour	self monitoring of behaviour
2	2	Goal setting (behaviour)	Action planning	social support	Instruction on how to perform the behaviour
2	2	Goal setting (behaviour)	Problem solving	Action planning	Feedback on behaviour
2	1	social support	Instruction on how to perform the behaviour	Behavioural practice/rehearsal	Credible source
3	3	Action planning	self monitoring of behaviour	social support	Graded tasks
2	2	Goal setting (behaviour)	Action planning	self monitoring of behaviour	Behavioural practice/rehearsal
2	2	Goal setting (behaviour)	Action planning	Feedback on behaviour	Prompts/cues
2	1	self monitoring of behaviour	social support	Instruction on how to perform the behaviour	Behavioural practice/rehearsal
2	2	Goal setting (behaviour)	Feedback on behaviour	Instruction on how to perform the behaviour	Information about health consequences
5	5	Goal setting (behaviour)	Problem solving	Feedback on behaviour	self monitoring of behaviour
2	2	Goal setting (behaviour)	Problem solving	Feedback on behaviour	Prompts/cues
4	4	Goal setting (behaviour)	Action planning	self monitoring of behaviour	Credible source
1	1	Goal setting (behaviour)	Problem solving	Action planning	Information about health consequences
4	4	Goal setting (behaviour)	Problem solving	Instruction on how to perform the behaviour	Demonstration of the behaviour
5	5	Feedback on behaviour	self monitoring of behaviour	Demonstration of the behaviour	Graded tasks
1	1	self monitoring of behaviour	social support	Instruction on how to perform the behaviour	Information about health consequences
4	4	Goal setting (behaviour)	self monitoring of behaviour	Instruction on how to perform the behaviour	Demonstration of the behaviour
2	2	Problem solving	Feedback on behaviour	Prompts/cues	
4	4	Action planning	Feedback on behaviour	Demonstration of the behaviour	
2	2	Goal setting (behaviour)	Feedback on behaviour	Instruction on how to perform the behaviour	Behavioural practice/rehearsal
3	3	Review of behaviour goals	self monitoring of behaviour	Graded tasks	
5	5	Goal setting (behaviour)	Problem solving	Feedback on behaviour	self monitoring of behaviour

2	2	Goal setting (behaviour)	Problem solving	Feedback on behaviour	social support	Instruction on how to perform the behaviour	Graded tasks		78	100	100	0	0	0	0
2	2	self monitoring of behaviour	social support	Instruction on how to perform the behaviour	Demonstration of the behaviour	Graded tasks			89	100	100	0	0	0	0
2	2	Goal setting (behaviour)	Problem solving	Action planning	self monitoring of behaviour	social support	Graded tasks		78	100	100	0	0	0	0
4	4	Problem solving	Action planning	Feedback on behaviour	Graded tasks				75	100	100	0	0	0	0
4	4	Problem solving	Action planning	Feedback on behaviour	Credible source				88	75	100	0	0	0	0
2	2	Problem solving	Action planning	Review of behaviour goals	self monitoring of behaviour				75	0	100	0	0	0	0
2	2	Problem solving	Action planning	Demonstration of the behaviour	Behavioural practice/rehearsal				86	100	100	0	0	0	0
1	1	Problem solving	Action planning	Feedback on behaviour	Information about health consequences				63	50	100	0	0	0	0
2	2	Problem solving	Feedback on behaviour	self monitoring of behaviour	Behavioural practice/rehearsal				75	0	100	0	0	0	0
2	2	Problem solving	Review of behaviour goals	self monitoring of behaviour	Graded tasks				75	100	100	0	0	0	0
5	5	Problem solving	Graded tasks	Credible source					86	100	100	0	0	0	0
2	2	Problem solving	self monitoring of behaviour	Instruction on how to perform the behaviour	Prompts/cues				88	75	100	0	0	0	0
2	2	Problem solving	Feedback on behaviour	Prompts/cues	Graded tasks				88	100	100	0	0	0	0
2	2	Problem solving	self monitoring of behaviour	Demonstration of the behaviour	Behavioural practice/rehearsal				88	0	100	0	0	0	0
1	0	Problem solving	self monitoring of behaviour	social support	Behavioural practice/rehearsal				75	0	0	0	0	0	0
3	2	Problem solving	Instruction on how to perform the behaviour	Behavioural practice/rehearsal	Credible source				83	50	67	0	0	0	0
1	1	Problem solving	Instruction on how to perform the behaviour	Information about health consequences	Behavioural practice/rehearsal				67	100	100	0	0	0	0
3	3	Action planning	Feedback on behaviour	Instruction on how to perform the behaviour	Behavioural practice/rehearsal				57	0	100	0	0	0	0
3	3	Action planning	Review of behaviour goals	Feedback on behaviour	Instruction on how to perform the behaviour				71	0	100	0	0	0	0
3	3	Action planning	Review of behaviour goals	self monitoring of behaviour	Instruction on how to perform the behaviour				71	0	100	0	0	0	0
3	3	Action planning	Review of behaviour goals	Feedback on behaviour	self monitoring of behaviour				71	0	100	0	0	0	0
2	2	Action planning	self monitoring of behaviour	Demonstration of the behaviour	Behavioural practice/rehearsal				75	0	100	0	0	0	0
2	2	Goal setting (behaviour)	Problem solving	Feedback on behaviour	self monitoring of behaviour	social support	Instruction on how to perform the behaviour	Graded tasks	78	100	100	0	0	0	0
5	5	Feedback on behaviour	Instruction on how to perform the behaviour	Graded tasks	Credible source				86	100	100	0	0	0	0
5	5	Feedback on behaviour	self monitoring of behaviour	Graded tasks	Credible source				86	100	100	0	0	0	0
5	5	Instruction on how to perform the behaviour	Demonstration of the behaviour	Graded tasks	Credible source				100	100	100	0	0	0	0
5	5	Goal setting (behaviour)	Problem solving	Goal setting (outcome)	Feedback on behaviour	self monitoring of behaviour			86	100	100	0	0	0	0
5	5	self monitoring of behaviour	Demonstration of the behaviour	Graded tasks	Credible source				100	100	100	0	0	0	0
2	2	Goal setting (behaviour)	self monitoring of behaviour	social support	Instruction on how to perform the behaviour	Demonstration of the behaviour	Graded tasks		89	100	100	0	0	0	0
2	2	Problem solving	Feedback on behaviour	self monitoring of behaviour	social support	Instruction on how to perform the behaviour	Graded tasks		78	100	100	0	0	0	0
2	1	Action planning	Instruction on how to perform the behaviour	Information about health consequences	Credible source				71	33	50	0	0	0	0
4	4	Goal setting (behaviour)	Graded tasks	Credible source					86	100	100	0	0	0	0

1	0	social support	Information about health consequences	Social comparison					
1	0	Instruction on how to perform the behaviour	Information about health consequences	Social comparison					
4	4	Problem solving	Action planning	Feedback on behaviour	Demonstration of the behaviour				
2	2	Problem solving	Action planning	Feedback on behaviour	Prompts/cues				
2	2	Problem solving	Action planning	Feedback on behaviour	Behavioural practice/rehearsal				
5	5	Problem solving	self monitoring of behaviour	Instruction on how to perform the behaviour	Demonstration of the behaviour	Graded tasks	Credible source		
2	2	Problem solving	Action planning	Instruction on how to perform the behaviour	Prompts/cues				
1	1	Problem solving	Action planning	Information about health consequences	Behavioural practice/rehearsal				
2	1	Problem solving	Action planning	Information about health consequences	Credible source				
2	2	Problem solving	Review of behaviour goals	Feedback on behaviour	Graded tasks				
3	3	Problem solving	Review of behaviour goals	Feedback on behaviour	Instruction on how to perform the behaviour				
5	5	Problem solving	Feedback on behaviour	self monitoring of behaviour	Instruction on how to perform the behaviour	Graded tasks	Credible source		
3	3	Problem solving	Review of behaviour goals	self monitoring of behaviour	Instruction on how to perform the behaviour				
1	1	Feedback on behaviour	self monitoring of behaviour	Social comparison					
2	2	Problem solving	Discrepancy between current behaviour and goal	Feedback on behaviour	self monitoring of behaviour				
2	2	Problem solving	Feedback on behaviour	Instruction on how to perform the behaviour	Prompts/cues				
5	3	Problem solving	Goal setting (outcome)	social support	Instruction on how to perform the behaviour				
5	3	Problem solving	Goal setting (outcome)	self monitoring of behaviour	social support				
0	0	Goal setting (behaviour)	self monitoring of behaviour	Information about health consequences	Adding object to the environment				
4	4	Goal setting (behaviour)	Feedback on behaviour	self monitoring of behaviour	Instruction on how to perform the behaviour	Demonstration of the behaviour	Credible source		
4	4	Goal setting (behaviour)	Feedback on behaviour	self monitoring of behaviour	Instruction on how to perform the behaviour	Graded tasks	Credible source		
2	1	Problem solving	Instruction on how to perform the behaviour	Information about health consequences	Demonstration of the behaviour				
2	2	Problem solving	Feedback on behaviour	Instruction on how to perform the behaviour	Behavioural practice/rehearsal				
1	1	Problem solving	self monitoring of behaviour	Information about health consequences	Credible source				
4	4	Goal setting (behaviour)	self monitoring of behaviour	Instruction on how to perform the behaviour	Demonstration of the behaviour	Graded tasks	Credible source		
3	3	Action planning	Review of behaviour goals	self monitoring of behaviour	Graded tasks				
2	2	Goal setting (behaviour)	Problem solving	self monitoring of behaviour	social support	Instruction on how to perform the behaviour	Demonstration of the behaviour	Graded tasks	
2	2	Goal setting (behaviour)	Feedback on behaviour	self monitoring of behaviour	social support	Instruction on how to perform the behaviour	Demonstration of the behaviour	Graded tasks	
4	4	Action planning	self monitoring of behaviour	Demonstration of the behaviour	Graded tasks				
1	1	Goal setting (behaviour)	Problem solving	Review of behaviour goals	self monitoring of behaviour	social support			
1	1	Action planning	Review of behaviour goals	self monitoring of behaviour	social support				
1	1	Goal setting (behaviour)	Action planning	Review of behaviour goals	self monitoring of behaviour	social support			
1	0	Action planning	Information about health consequences	Social comparison					
3	3	Goal setting (behaviour)	Problem solving	Action planning	Feedback on behaviour	self monitoring of behaviour	Instruction on how to perform the behaviour	Graded tasks	
3	3	Goal setting (behaviour)	Problem solving	Action planning	Feedback on behaviour	self monitoring of behaviour	Instruction on how to perform the behaviour	Credible source	
1	1	Problem solving	Review of behaviour goals	self monitoring of behaviour	social support				
0	0	self monitoring of behaviour	Information about health consequences	Adding object to the environment					
0	0	Goal setting (behaviour)	Problem solving	Action planning	self monitoring of behaviour	social support	Instruction on how to perform the behaviour	Information about health consequences	
0	0	Goal setting (behaviour)	Problem solving	Information about health consequences	Adding object to the environment				
1	0	social support	Information about health consequences	Demonstration of the behaviour					
2	2	Problem solving	Feedback on behaviour	social support	Adding object to the environment				
2	2	Problem solving	Feedback on behaviour	Instruction on how to perform the behaviour	Adding object to the environment				
2	2	Problem solving	self monitoring of behaviour	social support	Instruction on how to perform the behaviour	Demonstration of the behaviour	Graded tasks		
2	2	Goal setting (behaviour)	Problem solving	Action planning	self monitoring of behaviour	social support	Instruction on how to perform the behaviour	Graded tasks	
2	2	Feedback on behaviour	self monitoring of behaviour	social support	Instruction on how to perform the behaviour	Demonstration of the behaviour	Graded tasks		
3	3	Action planning	Feedback on behaviour	self monitoring of behaviour	social support	Instruction on how to perform the behaviour	Graded tasks		
2	2	Goal setting (behaviour)	Feedback on behaviour	self monitoring of behaviour	social support	Demonstration of the behaviour	Graded tasks		
2	2	Problem solving	Action planning	self monitoring of behaviour	social support	Instruction on how to perform the behaviour	Graded tasks		
2	2	Action planning	self monitoring of behaviour	Prompts/cues	Graded tasks				
4	4	Action planning	Instruction on how to perform the behaviour	Demonstration of the behaviour	Graded tasks				
4	4	Action planning	Feedback on behaviour	Demonstration of the behaviour	Graded tasks				
2	2	Feedback on behaviour	self monitoring of behaviour	Demonstration of the behaviour	Prompts/cues				
1	1	Feedback on behaviour	self monitoring of behaviour	Information about health consequences	Prompts/cues				
2	2	Review of behaviour goals	Feedback on behaviour	self monitoring of behaviour	Information about health consequences				
1	1	Goal setting (behaviour)	Problem solving	Instruction on how to perform the behaviour	Information about health consequences	Behavioural practice/rehearsal			
1	1	Goal setting (behaviour)	Problem solving	Feedback on behaviour	self monitoring of behaviour	Behavioural practice/rehearsal			
2	2	Goal setting (behaviour)	Problem solving	Feedback on behaviour	Instruction on how to perform the behaviour	Prompts/cues			
1	1	Goal setting (behaviour)	Problem solving	Review of behaviour goals	self monitoring of behaviour	Graded tasks			
2	2	Goal setting (behaviour)	Problem solving	Discrepancy between current behaviour and goal	Feedback on behaviour	self monitoring of behaviour			
2	2	Goal setting (behaviour)	Action planning	Review of behaviour goals	self monitoring of behaviour	Instruction on how to perform the behaviour			

2	2	Goal setting (behaviour)	Discrepancy between current behaviour and goal	Feedback on behaviour	Instruction on how to perform the behaviour			67	75	100	0	0	0	0	4
1	0	social support	Instruction on how to perform the behaviour	Information about health consequences	Demonstration of the behaviour			80	60	0	0	0	0	0	4
1	0	self monitoring of behaviour	social support	Behavioural practice/rehearsal	Credible source			83	50	0	0	0	0	0	4
1	0	Problem solving	Action planning	social support	Information about health consequences	Social comparison		67	0	0	0	0	0	0	5
1	0	Problem solving	Action planning	Instruction on how to perform the behaviour	Information about health consequences	Social comparison		67	0	0	0	0	0	0	5
1	1	Problem solving	Review of behaviour goals	Feedback on behaviour	self monitoring of behaviour	social support		57	50	100	0	0	0	0	5
4	4	Goal setting (behaviour)	Feedback on behaviour	Demonstration of the behaviour	Graded tasks	Credible source		100	100	100	0	0	0	0	5
1	1	Action planning	Review of behaviour goals	Feedback on behaviour	self monitoring of behaviour	social support		67	0	100	0	0	0	0	5
1	1	Action planning	Review of behaviour goals	Feedback on behaviour	social support	Instruction on how to perform the behaviour		67	0	100	0	0	0	0	5
3	3	Review of behaviour goals	Feedback on behaviour	self monitoring of behaviour	Instruction on how to perform the behaviour	Graded tasks		60	0	100	0	0	0	0	5
2	1	Discrepancy between current behaviour and goal	social support	Instruction on how to perform the behaviour				50	50	50	0	0	0	0	3
0	0	Action planning	self monitoring of behaviour	Instruction on how to perform the behaviour	Social comparison			57	0	0	0	0	0	0	4
0	0	self monitoring of behaviour	social support	Instruction on how to perform the behaviour	Social comparison			57	0	0	0	0	0	0	4
1	1	Goal setting (behaviour)	Problem solving	Review of behaviour goals	Feedback on behaviour	social support		57	50	100	0	0	0	0	5
3	3	Action planning	Review of behaviour goals	Feedback on behaviour	self monitoring of behaviour	Instruction on how to perform the behaviour	Graded tasks	60	0	100	0	0	0	0	6
1	1	Goal setting (behaviour)	Action planning	Review of behaviour goals	Feedback on behaviour	social support		67	0	100	0	0	0	0	5
0	0	Goal setting (behaviour)	Action planning	social support	Instruction on how to perform the behaviour	Social comparison		57	0	0	0	0	0	0	5
0	0	Problem solving	Action planning	Feedback on behaviour	social support	Information about health consequences		57	0	0	0	0	0	0	5
1	0	Problem solving	Action planning	social support	Instruction on how to perform the behaviour	Social comparison		67	0	0	0	0	0	0	5
0	0	Goal setting (behaviour)	self monitoring of behaviour	Verbal persuasion about capability				71	0	0	0	0	0	0	3
1	1	Feedback on behaviour	self monitoring of behaviour	Instruction on how to perform the behaviour	Social comparison			50	0	100	0	0	0	0	4
1	1	Problem solving	self monitoring of behaviour	social support	Prompts/cues	Graded tasks		86	100	100	0	0	0	0	5
2	2	Action planning	Feedback on behaviour	social support	Instruction on how to perform the behaviour	Demonstration of the behaviour		57	100	100	0	0	0	0	5
1	1	Action planning	Feedback on behaviour	social support	Instruction on how to perform the behaviour	Prompts/cues		57	50	100	0	0	0	0	5
2	2	Feedback on behaviour	self monitoring of behaviour	Instruction on how to perform the behaviour	Prompts/cues	Graded tasks		67	100	100	0	0	0	0	5
1	1	Action planning	Review of behaviour goals	Feedback on behaviour	social support			67	0	100	0	0	0	0	4
0	0	Action planning	self monitoring of behaviour	social support	Social comparison			57	0	0	0	0	0	0	4
1	1	Goal setting (behaviour)	self monitoring of behaviour	Prompts/cues	Adding object to the environment			86	100	100	0	0	0	0	4
0	0	Feedback on behaviour	self monitoring of behaviour	Information about health consequences	Adding object to the environment			86	100	0	0	0	0	0	4
2	2	Feedback on behaviour	social support	Demonstration of the behaviour	Credible source			86	100	100	0	0	0	0	4
0	0	Goal setting (behaviour)	Feedback on behaviour	self monitoring of behaviour	Information about health consequences	Adding object to the environment		86	100	0	0	0	0	0	5
2	2	Goal setting (behaviour)	Feedback on behaviour	social support	Demonstration of the behaviour	Credible source		86	100	100	0	0	0	0	5

[illegible]

3	Problem solving	Review of behaviour goals	Feedback on behaviour	Instruction on how to perform the behaviour	Credible source			60	50	100	0	0	0	0	5
1	Goal setting (behaviour)	Problem solving	Review of behaviour goals	Feedback on behaviour	social support	Instruction on how to perform the behaviour		50	50	100	0	0	0	0	6
0	Goal setting (behaviour)	Problem solving	Action planning	social support	Instruction on how to perform the behaviour	Social comparison		67	0	0	0	0	0	0	6
0	Goal setting (behaviour)	Problem solving	Action planning	social support	Information about health consequences	Social comparison		67	0	0	0	0	0	0	6
0	self monitoring of behaviour	social support	Instruction on how to perform the behaviour	Information about health consequences	Social comparison			67	0	0	0	0	0	0	5
1	self monitoring of behaviour	social support	Instruction on how to perform the behaviour	Information about health consequences	Graded tasks			60	100	100	0	0	0	0	5
2	Problem solving	Action planning	Review of behaviour goals	Feedback on behaviour	Instruction on how to perform the behaviour	Graded tasks		60	0	100	0	0	0	0	6
1	Goal setting (behaviour)	self monitoring of behaviour	Instruction on how to perform the behaviour	Demonstration of the behaviour	Behavioural practice/rehearsal	Credible source		80	100	100	0	0	0	0	6
2	Problem solving	Action planning	Review of behaviour goals	Feedback on behaviour	self monitoring of behaviour	Graded tasks		60	0	100	0	0	0	0	6
1	Problem solving	Review of behaviour goals	self monitoring of behaviour	Prompts/cues	Graded tasks			80	100	100	0	0	0	0	5
1	Problem solving	Action planning	self monitoring of behaviour	Information about health consequences	Behavioural practice/rehearsal			60	0	100	0	0	0	0	5
2	Problem solving	Review of behaviour goals	self monitoring of behaviour	Instruction on how to perform the behaviour	Graded tasks			60	0	100	0	0	0	0	5
3	Problem solving	Review of behaviour goals	self monitoring of behaviour	Instruction on how to perform the behaviour	Credible source			60	50	100	0	0	0	0	5
4	Problem solving	Action planning	self monitoring of behaviour	Graded tasks	Credible source			80	100	100	0	0	0	0	5
4	Problem solving	Action planning	self monitoring of behaviour	Demonstration of the behaviour	Credible source			100	100	100	0	0	0	0	5
1	Goal setting (behaviour)	Action planning	self monitoring of behaviour	social support	Information about health consequences	Behavioural practice/rehearsal		60	0	100	0	0	0	0	6
1	Goal setting (behaviour)	social support	Instruction on how to perform the behaviour	Demonstration of the behaviour	Behavioural practice/rehearsal	Credible source		80	100	100	0	0	0	0	6
3	Goal setting (behaviour)	Action planning	social support	Instruction on how to perform the behaviour	Demonstration of the behaviour	Credible source		80	100	100	0	0	0	0	6
0	Goal setting (behaviour)	self monitoring of behaviour	social support	Information about health consequences	Adding object to the environment			83	100	0	0	0	0	0	5
0	Goal setting (behaviour)	self monitoring of behaviour	social support	Instruction on how to perform the behaviour	Information abour social & environmental consequences			67	100	0	0	0	0	0	5
1	Feedback on behaviour	Information about health consequences	Credible source					80	50	100	0	0	0	0	3
2	Review of behaviour goals	Information about health consequences	Graded tasks					50	100	100	0	0	0	0	3
2	Feedback on behaviour	Information about health consequences	Graded tasks					50	100	100	0	0	0	0	3
4	Goal setting (behaviour)	Problem solving	Feedback on behaviour	self monitoring of behaviour	Demonstration of the behaviour	Graded tasks	Credible source	100	100	100	0	0	0	0	7
1	Review of behaviour goals	Prompts/cues	Graded tasks					80	100	100	0	0	0	0	3
2	Goal setting (behaviour)	Review of behaviour goals	social support	Instruction on how to perform the behaviour	Credible source			50	67	100	0	0	0	0	5
1	Goal setting (behaviour)	self monitoring of behaviour	social support	Information about health consequences	Behavioural practice/rehearsal			60	0	100	0	0	0	0	5
0	Goal setting (behaviour)	self monitoring of behaviour	social support	Information about health consequences	Credible source			83	50	0	0	0	0	0	5
2	Problem solving	Action planning	Instruction on how to perform the behaviour	Behavioural practice/rehearsal	Credible source			80	0	100	0	0	0	0	5
4	Problem solving	Action planning	Instruction on how to perform the behaviour	Graded tasks	Credible source			80	100	100	0	0	0	0	5
2	Problem solving	Review of behaviour goals	Feedback on behaviour	Instruction on how to perform the behaviour	Graded tasks			60	0	100	0	0	0	0	5
2	Problem solving	Action planning	Instruction on how to perform the behaviour	Demonstration of the behaviour	Behavioural practice/rehearsal			75	100	100	0	0	0	0	5
2	Problem solving	Action planning	Instruction on how to perform the behaviour	Prompts/cues	Graded tasks			80	100	100	0	0	0	0	5
2	Problem solving	Action planning	Review of behaviour goals	self monitoring of behaviour	Instruction on how to perform the behaviour	Graded tasks		60	0	100	0	0	0	0	6
0	Action planning	social support	Instruction on how to perform the behaviour	Social comparison	Behavioural practice/rehearsal			50	0	0	0	0	0	0	5
0	Feedback on behaviour	self monitoring of behaviour	social support	Instruction on how to perform the behaviour	Social comparison			50	0	0	0	0	0	0	5
2	Goal setting (behaviour)	self monitoring of behaviour	social support	Graded tasks	Adding object to the environment			100	100	100	0	0	0	0	5
2	Goal setting (behaviour)	self monitoring of behaviour	Instruction on how to perform the behaviour	Graded tasks	Adding object to the environment			100	100	100	0	0	0	0	5
0	Action planning	self monitoring of behaviour	social support	Information about health consequences	Social comparison			67	0	0	0	0	0	0	5
0	Action planning	self monitoring of behaviour	Instruction on how to perform the behaviour	Information about health consequences	Social comparison			67	0	0	0	0	0	0	5
0	Action planning	Feedback on behaviour	social support	Instruction on how to perform the behaviour	Social comparison			50	0	0	0	0	0	0	5
1	Problem solving	Action planning	self monitoring of behaviour	Instruction on how to perform the behaviour	Information about health consequences	Behavioural practice/rehearsal		60	0	100	0	0	0	0	6

3	3	self monitoring of behaviour	Self monitoring of outcome(s) of behaviour	Instruction on how to perform the behaviour				
0	0	Goal setting (behaviour)	Problem solving	Instruction on how to perform the behaviour	Information abour social & environmental consequences			
0	0	self monitoring of behaviour	social support	Information abour social & environmental consequences				
1	1	Goal setting (behaviour)	Problem solving	Review of behaviour goals	Prompts/cues	Graded tasks		
2	2	self monitoring of behaviour	social support	Graded tasks	Adding object to the environment			
2	2	Goal setting (behaviour)	Problem solving	Discrepancy between current behaviour and goal	self monitoring of behaviour	Instruction on how to perform the behaviour		
2	2	Goal setting (behaviour)	Problem solving	Discrepancy between current behaviour and goal	self monitoring of behaviour	Graded tasks		
2	2	Goal setting (behaviour)	Problem solving	Discrepancy between current behaviour and goal	Feedback on behaviour	Instruction on how to perform the behaviour		
2	2	Goal setting (behaviour)	Problem solving	Review of behaviour goals	Instruction on how to perform the behaviour	Credible source		
1	1	Goal setting (behaviour)	Problem solving	Review of behaviour goals	Discrepancy between current behaviour and goal	self monitoring of behaviour		
2	2	Goal setting (behaviour)	Action planning	Review of behaviour goals	Feedback on behaviour	self monitoring of behaviour	Instruction on how to perform the behaviour	Graded tasks
4	4	Goal setting (behaviour)	Problem solving	Goal setting (outcome)	Instruction on how to perform the behaviour	Demonstration of the behaviour		
1	1	Goal setting (behaviour)	Problem solving	Feedback on behaviour	self monitoring of behaviour	social support	Instruction on how to perform the behaviour	Prompts/cues
1	1	Goal setting (behaviour)	Problem solving	Feedback on behaviour	self monitoring of behaviour	Social comparison		
5	5	Goal setting (behaviour)	Problem solving	Goal setting (outcome)	Instruction on how to perform the behaviour	Credible source		
0	0	Action planning	social support	Information abour social & environmental consequences				
0	0	Action planning	Instruction on how to perform the behaviour	Information abour social & environmental consequences				
2	2	Goal setting (behaviour)	Problem solving	Action planning	self monitoring of behaviour	social support	Instruction on how to perform the behaviour	Demonstration of the behaviour
0	0	Goal setting (behaviour)	Problem solving	Action planning	self monitoring of behaviour	social support	Instruction on how to perform the behaviour	Behavioural practice/rehearsal
0	0	self monitoring of behaviour	Instruction on how to perform the behaviour	Information abour social & environmental consequences				
2	2	Goal setting (outcome)	self monitoring of behaviour	Adding object to the environment				
0	0	social support	Information abour social & environmental consequences	Behavioural practice/rehearsal				
0	0	Instruction on how to perform the behaviour	Information abour social & environmental consequences	Behavioural practice/rehearsal				
3	3	self monitoring of behaviour	Credible source	Adding object to the environment				
3	2	Problem solving	Action planning	Discrepancy between current behaviour and goal	Credible source			
1	1	Goal setting (behaviour)	Problem solving	Action planning	self monitoring of behaviour	social support	Instruction on how to perform the behaviour	Prompts/cues
2	2	Goal setting (behaviour)	social support	Graded tasks	Adding object to the environment			
1	1	Goal setting (behaviour)	Problem solving	self monitoring of behaviour	social support	Instruction on how to perform the behaviour	Prompts/cues	Graded tasks
1	1	Goal setting (behaviour)	Problem solving	Action planning	self monitoring of behaviour	social support	Prompts/cues	Graded tasks
2	2	Problem solving	Action planning	Feedback on behaviour	Adding object to the environment			
2	2	Goal setting (behaviour)	Feedback on behaviour	Graded tasks	Adding object to the environment			
2	2	Feedback on behaviour	Graded tasks	Adding object to the environment				
2	2	Feedback on behaviour	self monitoring of behaviour	Graded tasks	Adding object to the environment			
1	1	Goal setting (behaviour)	Action planning	Feedback on behaviour	self monitoring of behaviour	social support	Instruction on how to perform the behaviour	Prompts/cues
2	1	Instruction on how to perform the behaviour	Information about health consequences	Demonstration of the behaviour	Credible source			
1	1	Instruction on how to perform the behaviour	Information about health consequences	Demonstration of the behaviour	Behavioural practice/rehearsal			
2	2	Problem solving	Feedback on behaviour	Graded tasks	Adding object to the environment			
1	1	Goal setting (behaviour)	Problem solving	Action planning	Review of behaviour goals	Instruction on how to perform the behaviour		
1	1	Goal setting (behaviour)	Problem solving	Action planning	Review of behaviour goals	Graded tasks		
2	2	Goal setting (behaviour)	Problem solving	Action planning	Discrepancy between current behaviour and goal	self monitoring of behaviour		
1	1	Problem solving	self monitoring of behaviour	social support	Instruction on how to perform the behaviour	Prompts/cues	Graded tasks	
1	1	Action planning	Feedback on behaviour	self monitoring of behaviour	social support	Instruction on how to perform the behaviour	Prompts/cues	
2	2	Goal setting (behaviour)	Action planning	Discrepancy between current behaviour and goal	Feedback on behaviour	self monitoring of behaviour	Instruction on how to perform the behaviour	Credible source
2	2	Goal setting (behaviour)	Action planning	Feedback on behaviour	self monitoring of behaviour	Instruction on how to perform the behaviour	Prompts/cues	Graded tasks
1	1	Goal setting (behaviour)	Problem solving	Action planning	Review of behaviour goals	Feedback on behaviour		
1	1	Problem solving	Action planning	self monitoring of behaviour	social support	Instruction on how to perform the behaviour	Prompts/cues	
2	2	Goal setting (behaviour)	Problem solving	Action planning	Feedback on behaviour	social support	Credible source	
0	0	Goal setting (behaviour)	Problem solving	Action planning	social support	Instruction on how to perform the behaviour	Information about health consequences	Behavioural practice/rehearsal
1	1	Problem solving	Review of behaviour goals	Prompts/cues	Graded tasks			
2	2	Problem solving	Review of behaviour goals	Instruction on how to perform the behaviour	Graded tasks			
0	0	Goal setting (behaviour)	Information about health consequences	Information abour social & environmental consequences				
0	0	Goal setting (behaviour)	Information abour social & environmental consequences	Behavioural practice/rehearsal				
5	5	Goal setting (behaviour)	Problem solving	Goal setting (outcome)	Credible source			
0	0	Problem solving	social support	Information abour social & environmental consequences				
0	0	Problem solving	Information about health consequences	Information abour social & environmental consequences				
1	1	Goal setting (behaviour)	Problem solving	self monitoring of behaviour	Social comparison			
1	1	Goal setting (behaviour)	Problem solving	Feedback on behaviour	Social comparison			
1	1	Problem solving	Action planning	self monitoring of behaviour	social support	Prompts/cues	Graded tasks	
1	1	Goal setting (behaviour)	self monitoring of behaviour	social support	Instruction on how to perform the behaviour	Prompts/cues	Graded tasks	
2	2	Problem solving	Action planning	Feedback on behaviour	social support	Adding object to the environment		
1	1	Problem solving	Feedback on behaviour	self monitoring of behaviour	social support	Instruction on how to perform the behaviour	Prompts/cues	
1	1	Goal setting (behaviour)	Problem solving	Feedback on behaviour	social support	Instruction on how to perform the behaviour	Prompts/cues	
2	2	Instruction on how to perform the behaviour	Demonstration of the behaviour	Prompts/cues	Graded tasks			
1	1	Goal setting (behaviour)	Action planning	self monitoring of behaviour	social support	Prompts/cues	Graded tasks	
1	1	Goal setting (behaviour)	Problem solving	social support	Instruction on how to perform the behaviour	Prompts/cues	Graded tasks	
2	2	Goal setting (behaviour)	Feedback on behaviour	self monitoring of behaviour	Graded tasks	Adding object to the environment		
1	1	Goal setting (behaviour)	Problem solving	Action planning	social support	Instruction on how to perform the behaviour	Prompts/cues	
1	1	Goal setting (behaviour)	Problem solving	Action planning	social support	Prompts/cues	Graded tasks	
2	2	Goal setting (behaviour)	Action planning	Feedback on behaviour	self monitoring of behaviour	Instruction on how to perform the behaviour	Information about health consequences	Behavioural practice/rehearsal
2	2	Goal setting (behaviour)	Problem solving	Feedback on behaviour	Graded tasks	Adding object to the environment		
2	2	Goal setting (behaviour)	Problem solving	Action planning	Feedback on behaviour	Adding object to the environment		
2	2	Problem solving	Action planning	Feedback on behaviour	Instruction on how to perform the behaviour	Adding object to the environment		
0	0	Goal setting (behaviour)	Problem solving	Feedback on behaviour	self monitoring of behaviour	Information about health consequences	Adding object to the environment	
0	0	Problem solving	Feedback on behaviour	self monitoring of behaviour	Information about health consequences	Adding object to the environment		
5	5	Goal setting (outcome)	Instruction on how to perform the behaviour	Demonstration of the behaviour	Credible source			
2	2	self monitoring of behaviour	Demonstration of the behaviour	Prompts/cues	Graded tasks			
1	1	self monitoring of behaviour	social support	Instruction on how to perform the behaviour	Prompts/cues	Graded tasks		
1	1	Action planning	self monitoring of behaviour	social support	Prompts/cues	Graded tasks		
5	5	Goal setting (behaviour)	Goal setting (outcome)	Feedback on behaviour	Credible source			
2	2	Problem solving	Action planning	Discrepancy between current behaviour and goal	self monitoring of behaviour			
2	3	Problem solving	Action planning	Discrepancy between current behaviour and goal	Instruction on how to perform the behaviour			
4	4	Problem solving	Action planning	Graded tasks	Credible source			
1	1	Problem solving	Review of behaviour goals	Discrepancy between current behaviour and goal	self monitoring of behaviour			
2	2	Problem solving	Action planning	Behavioural practice/rehearsal	Credible source			
4	4	Goal setting (behaviour)	Problem solving	Goal setting (outcome)	Demonstration of the behaviour			
2	2	Goal setting (behaviour)	Problem solving	Action planning	Feedback on behaviour	self monitoring of behaviour	social support	Credible source
3	3	Problem solving	Review of behaviour goals	self monitoring of behaviour	Credible source			
1	1	Problem solving	Review of behaviour goals	Instruction on how to perform the behaviour	Information about health consequences			
1	1	Problem solving	Review of behaviour goals	Feedback on behaviour	Prompts/cues			
0	0	Goal setting (behaviour)	Problem solving	Information abour social & environmental consequences				
2	2	Goal setting (behaviour)	Goal setting (outcome)	Adding object to the environment				
0	0	Goal setting (behaviour)	Action planning	Information abour social & environmental consequences				
0	0	Problem solving	Instruction on how to perform the behaviour	Information abour social & environmental consequences				
0	0	Goal setting (behaviour)	self monitoring of behaviour	Information abour social & environmental consequences				
2	2	Discrepancy between current behaviour and goal	self monitoring of behaviour	Instruction on how to perform the behaviour	Credible source			
1	1	Problem solving	Action planning	social support	Prompts/cues	Graded tasks		
2	2	Problem solving	Feedback on behaviour	self monitoring of behaviour	Graded tasks	Adding object to the environment		
2	2	Feedback on behaviour	self monitoring of behaviour	Information about health consequences	Behavioural practice/rehearsal			
2	2	Feedback on behaviour	self monitoring of behaviour	Information about health consequences	Graded tasks			
1	1	Feedback on behaviour	self monitoring of behaviour	Information about health consequences	Credible source			
2	2	Problem solving	Action planning	Feedback on behaviour	self monitoring of behaviour	Adding object to the environment		
1	1	Problem solving	social support	Instruction on how to perform the behaviour	Prompts/cues	Graded tasks		
1	1	Problem solving	Feedback on behaviour	social support	Instruction on how to perform the behaviour	Prompts/cues		
1	1	Problem solving	Action planning	social support	Instruction on how to perform the behaviour	Prompts/cues		
1	1	Goal setting (behaviour)	social support	Instruction on how to perform the behaviour	Prompts/cues	Graded tasks		
1	1	Goal setting (behaviour)	Action planning	social support	Prompts/cues	Graded tasks		
5	5	Goal setting (outcome)	self monitoring of behaviour	Demonstration of the behaviour	Credible source			
5	5	Goal setting (outcome)	self monitoring of behaviour	Instruction on how to perform the behaviour	Demonstration of the behaviour			
2	2	Goal setting (behaviour)	Problem solving	Action planning	Feedback on behaviour	Instruction on how to perform the behaviour	Adding object to the environment	
2	2	Feedback on behaviour	Instruction on how to perform the behaviour	Information about health consequences	Behavioural practice/rehearsal			
2	2	Goal setting (behaviour)	Problem solving	Action planning	Feedback on behaviour	self monitoring of behaviour	Adding object to the environment	
2	2	Goal setting (behaviour)	Problem solving	Action planning	Feedback on behaviour	social support	Adding object to the environment	
2	2	Feedback on behaviour	Instruction on how to perform the behaviour	Demonstration of the behaviour	Prompts/cues			
4	4	Goal setting (behaviour)	Problem solving	Feedback on behaviour	Demonstration of the behaviour	Graded tasks	Credible source	
2	2	social support	Graded tasks	Adding object to the environment				
1	1	social support	Instruction on how to perform the behaviour	Prompts/cues	Graded tasks			
5	5	Goal setting (outcome)	Feedback on behaviour	Instruction on how to perform the behaviour	Demonstration of the behaviour			
2	2	Feedback on behaviour	Demonstration of the behaviour	Behavioural practice/rehearsal	Graded tasks			
2	2	Feedback on behaviour	Demonstration of the behaviour	Behavioural practice/rehearsal	Credible source			
2	2	Feedback on behaviour	Instruction on how to perform the behaviour	Prompts/cues	Credible source			
1	1	Action planning	social support	Prompts/cues	Graded tasks			
2	2	Problem solving	Action planning	Review of behaviour goals	Feedback on behaviour	self monitoring of behaviour	Instruction on how to perform the behaviour	Graded tasks
1	1	Goal setting (behaviour)	Feedback on behaviour	Prompts/cues	Adding object to the environment			
1	1	social support	Demonstration of the behaviour	Prompts/cues				
2	2	Goal setting (behaviour)	Feedback on behaviour	Demonstration of the behaviour	Adding object to the environment			
2	2	Feedback on behaviour	Demonstration of the behaviour	Adding object to the environment				
2	2	Goal setting (behaviour)	Problem solving	Action planning	Feedback on behaviour	social support	Instruction on how to perform the behaviour	Credible source
2	2	Problem solving	Goal setting (outcome)	self monitoring of behaviour	Instruction on how to perform the behaviour	Demonstration of the behaviour	Credible source	

5	3	2	5	0	0	0	0	0	0	0	0	0	0	0	2	3	0	0	0	0	0	5	5	5	5	5	5	5	Problem solving	Goal setting (outcome)	Feedback on behaviour	self monitoring of behaviour	Instruction on how to perform the behaviour	Graded tasks			100	100	100	0	0	0	0	6
5	3	2	5	0	0	0	0	0	0	0	0	0	0	0	2	3	0	0	0	0	0	5	5	5	5	5	5	5	Problem solving	Goal setting (outcome)	Feedback on behaviour	self monitoring of behaviour	Demonstration of the behaviour	Credible source			100	100	100	0	0	0	0	6
5	3	2	5	0	0	0	0	0	0	0	0	0	0	0	2	3	0	0	0	0	0	5	5	5	5	5	5	5	Problem solving	Goal setting (outcome)	Feedback on behaviour	self monitoring of behaviour	Graded tasks	Credible source			100	100	100	0	0	0	0	6
5	4	2	4	0	0	0	0	0	0	0	0	0	0	0	1	2	0	0	0	0	0	5	4	4	4	4	4	4	Goal setting (outcome)	Action planning	self monitoring of behaviour	Demonstration of the behaviour					100	100	100	0	0	0	0	4
5	3	0	0	0	0	0	0	2	0	0	0	0	0	0	0	0	0	0	0	0	0	5	0	0	0	0	0	0	Problem solving	Action planning	Information about emotinal consequences						60	0	0	0	0	0	0	3
5	2	0	3	0	0	0	0	1	0	0	0	0	0	0	2	3	0	0	0	0	0	5	3	3	3	3	3	3	Action planning	Review of behaviour goals	Feedback on behaviour	Behavioural practice/rehearsal					67	0	100	0	0	0	0	4
5	4	0	0	0	0	0	0	1	2	0	0	0	0	0	0	0	0	0	0	0	0	5	2	0	2	0	0	0	Goal setting (behaviour)	Feedback on behaviour	Verbal persuasion about capability						80	0	0	0	0	0	0	3
5	4	1	0	0	0	0	0	1	0	0	0	0	0	0	0	0	0	0	0	0	0	5	1	0	1	0	0	0	Review of behaviour goals	self monitoring of behaviour	Adding object to the environment						80	100	0	0	0	0	0	3
5	3	1	1	0	0	0	0	2	2	0	0	0	0	0	0	0	0	0	0	0	0	5	3	1	3	1	1	1	Goal setting (behaviour)	Feedback on behaviour	self monitoring of behaviour	social support	Prompts/cues	Credible source			60	33	100	0	0	0	0	6
5	4	2	1	0	0	0	0	1	0	0	0	0	0	0	0	0	0	0	0	0	0	5	2	1	2	1	1	1	Goal setting (behaviour)	Feedback on behaviour	self monitoring of behaviour	social support	Prompts/cues	Adding object to the environment			80	100	100	0	0	0	0	6
5	4	0	0	0	0	0	0	1	1	0	0	0	0	0	0	0	0	0	0	0	0	5	1	0	1	0	0	0	Goal setting (behaviour)	Problem solving	Verbal persuasion about capability						80	0	0	0	0	0	0	3
5	3	0	0	0	0	0	0	2	0	0	0	0	0	0	0	0	0	0	0	0	0	5	0	0	0	0	0	0	Goal setting (behaviour)	Action planning	Information about emotinal consequences						60	0	0	0	0	0	0	3
5	3	0	0	0	0	0	0	2	0	0	0	0	0	0	0	0	0	0	0	0	0	5	0	0	0	0	0	0	self monitoring of behaviour	Information about health consequences	Information about emotinal consequences						60	0	0	0	0	0	0	3
5	3	0	0	0	0	0	0	2	0	0	0	0	0	0	0	0	0	0	0	0	0	5	0	0	0	0	0	0	Goal setting (behaviour)	Problem solving	Action planning	Instruction on how to perform the behaviour	Information about emotinal consequences				60	0	0	0	0	0	0	5
5	4	0	0	0	0	0	0	1	2	0	0	0	0	0	0	0	0	0	0	0	0	5	2	0	2	0	0	0	Goal setting (behaviour)	Prompts/cues	Verbal persuasion about capability						80	0	0	0	0	0	0	3
5	3	1	1	0	0	0	0	1	1	0	0	0	0	0	1	1	0	0	0	0	0	5	3	1	3	1	1	1	Goal setting (behaviour)	Problem solving	Action planning	Feedback on behaviour	self monitoring of behaviour	Instruction on how to perform the behaviour	Information about health consequences	Credible source	75	50	100	0	0	0	0	8
5	2	1	1	0	0	0	0	1	2	1	0	0	0	0	1	1	0	0	0	0	0	4	4	2	3	1	2	1	Action planning	Discrepancy between current behaviour and goal	Instruction on how to perform the behaviour	Information about health consequences					67	33	50	0	0	0	0	4
5	1	1	2	0	0	0	0	1	1	0	0	0	0	0	1	1	0	0	0	0	0	4	3	3	2	2	3	2	Action planning	Discrepancy between current behaviour and goal	Instruction on how to perform the behaviour	Demonstration of the behaviour					33	50	67	0	0	0	0	4
5	3	0	2	0	0	0	0	1	1	0	0	0	0	0	1	2	0	0	0	0	0	5	3	2	3	2	2	2	Action planning	Review of behaviour goals	Feedback on behaviour	Credible source					75	0	100	0	0	0	0	4
5	3	0	2	0	0	0	0	1	0	0	0	0	0	0	1	2	0	0	0	0	0	5	2	2	2	2	2	2	Action planning	Review of behaviour goals	self monitoring of behaviour	Demonstration of the behaviour					75	0	100	0	0	0	0	4
5	3	0	1	0	0	0	0	1	1	0	0	0	0	0	1	1	0	0	0	0	0	5	2	1	2	1	1	1	Action planning	Review of behaviour goals	self monitoring of behaviour	Prompts/cues					75	0	100	0	0	0	0	4
5	4	1	1	0	0	0	0	1	0	0	0	0	0	0	0	0	0	0	0	0	0	5	1	1	1	1	1	1	Action planning	Prompts/cues	Social reward						80	100	100	0	0	0	0	3
5	4	0	0	0	0	0	0	1	2	0	0	0	0	0	0	0	0	0	0	0	0	5	2	0	2	0	0	0	Feedback on behaviour	self monitoring of behaviour	Verbal persuasion about capability						80	0	0	0	0	0	0	3
5	5	2	1	0	0	0	0	0	0	0	0	0	0	0	0	0	0	0	0	0	0	5	2	1	2	1	1	1	social support	Prompts/cues	Social reward						100	100	100	0	0	0	0	3
5	4	0	0	0	0	0	0	1	2	0	0	0	0	0	0	0	0	0	0	0	0	5	2	0	2	0	0	0	social support	Prompts/cues	Verbal persuasion about capability						80	0	0	0	0	0	0	3
5	2	0	1	0	0	0	0	2	1	0	0	0	0	0	1	1	0	0	0	0	0	5	2	1	2	1	1	1	Goal setting (behaviour)	Problem solving	Action planning	Review of behaviour goals	Feedback on behaviour	self monitoring of behaviour	Instruction on how to perform the behaviour	Information about health consequences	50	0	100	0	0	0	0	8
5	2	0	1	0	0	0	0	2	0	0	0	0	0	0	1	1	0	0	0	0	0	5	2	1	1	1	1	1	Goal setting (behaviour)	Problem solving	Action planning	Review of behaviour goals	Feedback on behaviour	self monitoring of behaviour	Instruction on how to perform the behaviour	Graded tasks	50	0	100	0	0	0	0	8
5	5	0	1	0	0	0	0	0	1	0	0	0	0	0	0	0	0	0	0	0	0	5	1	0	1	0	0	0	Action planning	social support	Verbal persuasion about capability						100	0	0	0	0	0	0	3

5	3	0	2	0	0	0	0	0	1	1	0	0	0	0	1	2	0	0	0	0	0	4	3	3	2	2	3	2	Problem solving	Goal setting (outcome)	self monitoring of behaviour	Behavioural practice/rehearsal				100	0	67	0	0	0	0	4
5	3	0	2	0	0	0	0	0	1	1	0	0	0	0	1	2	0	0	0	0	0	4	3	3	2	2	3	2	Problem solving	Goal setting (outcome)	Instruction on how to perform the behaviour	Behavioural practice/rehearsal				100	0	67	0	0	0	0	4
5	4	2	4	0	0	0	0	0	0	0	0	0	0	0	1	2	0	0	0	0	0	4	4	4	4	4	4	4	Problem solving	Goal setting (outcome)	Action planning	Demonstration of the behaviour				100	100	100	0	0	0	0	4
5	2	0	1	0	0	0	0	2	1	0	0	0	0	0	1	1	0	0	0	0	0	5	2	1	2	1	1	1	self monitoring of behaviour	Social comparison	Credible source					50	0	100	0	0	0	0	3
5	4	1	0	0	0	0	0	1	0	0	0	0	0	0	0	0	0	0	0	0	0	5	1	0	1	0	0	0	self monitoring of behaviour	Information about health consequences	Information abour social & environmental consequences					80	100	0	0	0	0	0	3
5	2	0	1	0	0	0	0	1	2	1	0	0	0	0	1	1	0	0	0	0	0	4	3	2	2	1	2	1	Instruction on how to perform the behaviour	Social comparison	Credible source					67	0	50	0	0	0	0	3
5	2	0	2	0	0	0	0	1	0	0	0	0	0	0	2	2	0	0	0	0	0	5	2	2	2	2	2	2	Instruction on how to perform the behaviour	Generalisation of target behaviour	Credible source					67	0	100	0	0	0	0	3
5	4	2	2	0	0	0	0	1	0	0	0	0	0	0	0	0	0	0	0	0	0	5	2	2	2	2	2	2	Goal setting (outcome)	Instruction on how to perform the behaviour	Adding object to the environment					80	100	100	0	0	0	0	3
5	4	2	2	0	0	0	0	1	0	0	0	0	0	0	0	0	0	0	0	0	0	5	2	2	2	2	2	2	Action planning	Demonstration of the behaviour	Adding object to the environment					80	100	100	0	0	0	0	3
5	4	3	3	0	0	0	0	1	0	0	0	0	0	0	0	0	0	0	0	0	0	5	3	3	3	3	3	3	Goal setting (behaviour)	Problem solving	Goal setting (outcome)	Feedback on behaviour	self monitoring of behaviour	social support		80	100	100	0	0	0	0	6
5	3	0	0	0	0	0	0	2	0	0	0	0	0	0	0	0	0	0	0	0	0	5	0	0	0	0	0	0	self monitoring of behaviour	Behavioural practice/rehearsal	Adding object to the environment					60	0	0	0	0	0	0	3
5	3	0	2	0	0	0	0	1	0	0	0	0	0	0	1	2	0	0	0	0	0	5	2	2	2	2	2	2	Problem solving	Action planning	Review of behaviour goals	Demonstration of the behaviour				75	0	100	0	0	0	0	4
5	3	0	1	0	0	0	0	1	1	0	0	0	0	0	1	1	0	0	0	0	0	5	2	1	2	1	1	1	Problem solving	Action planning	Review of behaviour goals	Prompts/cues				75	0	100	0	0	0	0	4
5	3	0	2	0	0	0	0	1	0	0	0	0	0	0	1	2	0	0	0	0	0	5	2	2	2	2	2	2	Problem solving	Action planning	Review of behaviour goals	Behavioural practice/rehearsal				75	0	100	0	0	0	0	4
5	2	0	1	0	0	0	0	2	1	0	0	0	0	0	1	1	0	0	0	0	0	5	2	1	2	1	1	1	Problem solving	Action planning	Review of behaviour goals	Information about health consequences				50	0	100	0	0	0	0	4
5	2	1	1	0	0	0	0	1	2	1	0	0	0	0	1	1	0	0	0	0	0	4	4	2	3	1	2	1	Problem solving	Action planning	Discrepancy between current behaviour and goal	Information about health consequences				67	33	50	0	0	0	0	4
5	3	2	2	0	0	0	0	1	1	0	0	0	0	0	1	1	0	0	0	0	0	5	4	2	4	2	2	2	Goal setting (behaviour)	Problem solving	Action planning	Discrepancy between current behaviour and goal	Feedback on behaviour			75	67	100	0	0	0	0	5
5	1	0	2	0	0	0	0	2	0	0	0	0	0	0	2	2	0	0	0	0	0	5	2	2	2	2	2	2	Goal setting (behaviour)	Action planning	Review of behaviour goals	self monitoring of behaviour	Instruction on how to perform the behaviour	Information about health consequences	Graded tasks	33	0	100	0	0	0	0	7
5	1	0	2	0	0	0	0	2	0	0	0	0	0	0	2	2	0	0	0	0	0	5	2	2	2	2	2	2	Goal setting (behaviour)	Action planning	Review of behaviour goals	Feedback on behaviour	Instruction on how to perform the behaviour	Information about health consequences	Graded tasks	33	0	100	0	0	0	0	7
5	4	2	2	0	0	0	0	1	0	0	0	0	0	0	0	0	0	0	0	0	0	5	2	2	2	2	2	2	Goal setting (behaviour)	Action planning	Feedback on behaviour	self monitoring of behaviour	social support	Demonstration of the behaviour	Graded tasks	80	100	100	0	0	0	0	7
5	4	2	2	0	0	0	0	1	0	0	0	0	0	0	0	0	0	0	0	0	0	5	2	2	2	2	2	2	Goal setting (behaviour)	Problem solving	Action planning	Feedback on behaviour	social support	Demonstration of the behaviour	Graded tasks	80	100	100	0	0	0	0	7
5	4	1	1	0	0	0	0	1	0	0	0	0	0	0	0	0	0	0	0	0	0	5	1	1	1	1	1	1	Action planning	self monitoring of behaviour	social support	Instruction on how to perform the behaviour	Prompts/cues	Graded tasks		80	100	100	0	0	0	0	6
5	4	2	1	0	0	0	0	1	0	0	0	0	0	0	0	0	0	0	0	0	0	5	2	1	2	1	1	1	Feedback on behaviour	self monitoring of behaviour	social support	Instruction on how to perform the behaviour	Prompts/cues	Graded tasks		80	100	100	0	0	0	0	6
5	4	1	0	0	0	0	0	0	2	1	0	0	0	0	0	0	0	0	0	0	0	4	3	1	2	0	1	0	social support	Information about health consequences	Pros and cons					100	33	0	0	0	0	0	3
5	4	0	0	0	0	0	0	1	0	0	0	0	0	0	0	0	0	0	0	0	0	5	0	0	0	0	0	0	Information about health consequences	Information abour social & environmental consequences	Behavioural practice/rehearsal					80	0	0	0	0	0	0	3
5	3	0	0	0	0	0	0	2	0	0	0	0	0	0	0	0	0	0	0	0	0	5	0	0	0	0	0	0	self monitoring of behaviour	Information abour social & environmental consequences	Behavioural practice/rehearsal					60	0	0	0	0	0	0	3
5	3	1	3	0	0	0	0	1	0	0	0	0	0	0	1	2	0	0	0	0	0	5	3	3	3	3	3	3	Problem solving	Goal setting (outcome)	Review of behaviour goals	self monitoring of behaviour				75	100	100	0	0	0	0	4
5	4	2	4	0	0	0	0	1	0	0	0	0	0	0	1	2	0	0	0	0	0	5	4	4	4	4	4	4	Problem solving	Goal setting (outcome)	Action planning	Feedback on behaviour				100	100	100	0	0	0	0	4
5	4	2	4	0	0	0	0	0	0	0	0	0	0	0	1	2	0	0	0	0	0	5	4	4	4	4	4	4	Problem solving	Goal setting (outcome)	Action planning	Credible source				100	100	100	0	0	0	0	4
5	3	1	3	0	0	0	0	1	0	0	0	0	0	0	1	2	0	0	0	0	0	5	3	3	3	3	3	3	Problem solving	Goal setting (outcome)	Review of behaviour goals	Feedback on behaviour				75	100	100	0	0	0	0	4
5	3	1	1	0	0	0	0	1	1	0	0	0	0	0	1	1	0	0	0	0	0	5	3	1	3	1	1	1	Goal setting (behaviour)	Instruction on how to perform the behaviour	Information about health consequences	Prompts/cues				75	50	100	0	0	0	0	4
5	2	0	1	0	0	0	0	2	0	0	0	0	0	0	1	1	0	0	0	0	0	5	1	1	1	1	1	1	Goal setting (behaviour)	Instruction on how to perform the behaviour	Behavioural practice/rehearsal	Generalisation of target behaviour				50	0	100	0	0	0	0	4

0	0	self monitoring of behaviour	Instruction on how to perform the behaviour	Information abour social & environmental consequences	Behavioural practice/rehearsal			
0	0	Problem solving	Action planning	Feedback on behaviour	Social comparison			
0	0	Problem solving	Action planning	self monitoring of behaviour	Social comparison			
2	2	Action planning	social support	Demonstration of the behaviour	Adding object to the environment			
1	1	Feedback on behaviour	self monitoring of behaviour	Prompts/cues	Graded tasks	Social reward		
4	4	Goal setting (behaviour)	Problem solving	Goal setting (outcome)	self monitoring of behaviour	Instruction on how to perform the behaviour	Demonstration of the behaviour	Credible source
1	1	Goal setting (behaviour)	Feedback on behaviour	self monitoring of behaviour	Prompts/cues	Graded tasks	Social reward	
1	1	Goal setting (behaviour)	Instruction on how to perform the behaviour	Prompts/cues	Graded tasks	Social reward		
1	1	Goal setting (behaviour)	Feedback on behaviour	Prompts/cues	Graded tasks	Social reward		
1	1	Goal setting (behaviour)	self monitoring of behaviour	Instruction on how to perform the behaviour	Prompts/cues	Social reward		
1	1	Goal setting (behaviour)	self monitoring of behaviour	Instruction on how to perform the behaviour	Graded tasks	Social reward		
0	0	Goal setting (behaviour)	Information about health consequences	Social comparison	Behavioural practice/rehearsal			
2	2	Goal setting (behaviour)	Problem solving	Goal setting (outcome)	self monitoring of behaviour	Adding object to the environment		
3	3	Action planning	social support	Credible source	Adding object to the environment			
0	0	Action planning	Instruction on how to perform the behaviour	Information about health consequences	Information abour social & environmental consequences			
3	3	Action planning	self monitoring of behaviour	Credible source	Adding object to the environment			
0	0	Action planning	social support	Information about health consequences	Information abour social & environmental consequences			
0	0	self monitoring of behaviour	Instruction on how to perform the behaviour	Information about health consequences	Information abour social & environmental consequences			
0	0	Action planning	Information about health consequences	Information abour social & environmental consequences	Behavioural practice/rehearsal			
4	4	Goal setting (behaviour)	Goal setting (outcome)	Feedback on behaviour	Instruction on how to perform the behaviour	Demonstration of the behaviour		
2	2	Action planning	Feedback on behaviour	self monitoring of behaviour	Instruction on how to perform the behaviour	Behavioural practice/rehearsal	Credible source	
1	1	Action planning	Feedback on behaviour	self monitoring of behaviour	Instruction on how to perform the behaviour	Information about health consequences	Prompts/cues	
2	2	Action planning	Feedback on behaviour	self monitoring of behaviour	Instruction on how to perform the behaviour	Information about health consequences	Graded tasks	
2	2	Action planning	self monitoring of behaviour	Instruction on how to perform the behaviour	Demonstration of the behaviour	Behavioural practice/rehearsal	Credible source	
2	2	Action planning	Feedback on behaviour	self monitoring of behaviour	Instruction on how to perform the behaviour	Prompts/cues	Credible source	
3	3	Action planning	Feedback on behaviour	self monitoring of behaviour	Instruction on how to perform the behaviour	Behavioural practice/rehearsal	Graded tasks	
4	4	Action planning	Feedback on behaviour	Instruction on how to perform the behaviour	Demonstration of the behaviour	Graded tasks	Credible source	
2	2	Goal setting (behaviour)	self monitoring of behaviour	social support	Demonstration of the behaviour	Credible source	Adding object to the environment	
0	0	social support	Instruction on how to perform the behaviour	Behavioural practice/rehearsal	Adding object to the environment			
0	0	self monitoring of behaviour	Instruction on how to perform the behaviour	Behavioural practice/rehearsal	Adding object to the environment			
3	3	self monitoring of behaviour	Instruction on how to perform the behaviour	Credible source	Adding object to the environment			
0	0	social support	Information about health consequences	Behavioural practice/rehearsal	Credible source			
2	2	Goal setting (behaviour)	Action planning	Discrepancy between current behaviour and goal	Demonstration of the behaviour			
1	1	Goal setting (behaviour)	Action planning	Review of behaviour goals	Prompts/cues			
3	3	Goal setting (behaviour)	Problem solving	Goal setting (outcome)	Action planning			
5	5	Goal setting (outcome)	self monitoring of behaviour	Demonstration of the behaviour	Graded tasks	Credible source		
5	5	Goal setting (outcome)	self monitoring of behaviour	Instruction on how to perform the behaviour	Demonstration of the behaviour	Graded tasks		
1	1	Goal setting (behaviour)	Problem solving	Discrepancy between current behaviour and goal	Information about health consequences			
5	5	Problem solving	Goal setting (outcome)	Graded tasks				
2	2	Goal setting (behaviour)	Generalisation of target behaviour	Credible source				
3	3	Goal setting (behaviour)	Social support (emotional)	Demonstration of the behaviour				
0	0	Goal setting (behaviour)	social support	Instruction on how to perform the behaviour	Information about health consequences	Information abour social & environmental consequences	Behavioural practice/rehearsal	
0	0	Goal setting (behaviour)	self monitoring of behaviour	social support	Demonstration of the behaviour	Behavioural practice/rehearsal	Credible source	
0	0	Problem solving	Information about health consequences	Social comparison	Behavioural practice/rehearsal			
0	0	Goal setting (behaviour)	self monitoring of behaviour	Information about health consequences	Social comparison			
0	0	Action planning	Feedback on behaviour	Information about health consequences	Social comparison			
0	0	Problem solving	social support	Social comparison	Behavioural practice/rehearsal			
1	1	Goal setting (behaviour)	Discrepancy between current behaviour and goal	self monitoring of behaviour	Information about health consequences	Prompts/cues		
1	1	Goal setting (behaviour)	Discrepancy between current behaviour and goal	Feedback on behaviour	Information about health consequences	Prompts/cues		
5	5	Goal setting (outcome)	Instruction on how to perform the behaviour	Demonstration of the behaviour	Graded tasks	Credible source		
5	5	Goal setting (outcome)	self monitoring of behaviour	Instruction on how to perform the behaviour	Graded tasks	Credible source		
0	0	Problem solving	Action planning	Social comparison	Behavioural practice/rehearsal			
1	1	Action planning	Feedback on behaviour	self monitoring of behaviour	Instruction on how to perform the behaviour	Information about health consequences	Credible source	
4	4	Action planning	self monitoring of behaviour	Instruction on how to perform the behaviour	Demonstration of the behaviour	Graded tasks	Credible source	
0	0	Goal setting (behaviour)	self monitoring of behaviour	Social comparison	Behavioural practice/rehearsal			
1	1	Goal setting (behaviour)	social support	Information about health consequences	Graded tasks			
0	0	Action planning	Feedback on behaviour	Social comparison	Behavioural practice/rehearsal			
0	0	Action planning	self monitoring of behaviour	Social comparison	Behavioural practice/rehearsal			
1	1	Action planning	social support	Information about health consequences	Graded tasks			
4	4	Goal setting (behaviour)	Goal setting (outcome)	self monitoring of behaviour	Demonstration of the behaviour	Credible source		

2	2	Goal setting (behaviour)	Action planning	self monitoring of behaviour	social support	Demonstration of the behaviour	Credible source	
2	2	Goal setting (behaviour)	Action planning	self monitoring of behaviour	social support	Demonstration of the behaviour	Adding object to the environment	
3	3	Goal setting (behaviour)	Action planning	self monitoring of behaviour	social support	Credible source	Adding object to the environment	
2	2	Goal setting (behaviour)	Action planning	Instruction on how to perform the behaviour	Demonstration of the behaviour	Behavioural practice/rehearsal	Credible source	
0	0	Goal setting (behaviour)	Action planning	self monitoring of behaviour	social support	Information about social & environmental consequences	Behavioural practice/rehearsal	
0	0	Goal setting (behaviour)	Action planning	self monitoring of behaviour	social support	Demonstration of the behaviour	Behavioural practice/rehearsal	
0	0	Goal setting (behaviour)	self monitoring of behaviour	social support	Information about social & environmental consequences	Behavioural practice/rehearsal		
5	5	Problem solving	Goal setting (outcome)	Feedback on behaviour	self monitoring of behaviour	Instruction on how to perform the behaviour	Demonstration of the behaviour	Credible source
1	1	Instruction on how to perform the behaviour	Information about health consequences	Prompts/cues				
1	1	self monitoring of behaviour	Information about health consequences	Demonstration of the behaviour				
1	1	Review of behaviour goals	Information about health consequences	Prompts/cues				
3	3	Review of behaviour goals	Behavioural practice/rehearsal	Graded tasks				
1	1	Goal setting (behaviour)	Action planning	social support	Instruction on how to perform the behaviour	Demonstration of the behaviour	Behavioural practice/rehearsal	
2	2	Goal setting (behaviour)	Action planning	Instruction on how to perform the behaviour	Demonstration of the behaviour	Adding object to the environment		
1	1	Review of behaviour goals	self monitoring of behaviour	social support	Instruction on how to perform the behaviour	Graded tasks		
1	1	Feedback on behaviour	social support	Instruction on how to perform the behaviour	Information about health consequences	Behavioural practice/rehearsal		
4	4	Problem solving	Action planning	Demonstration of the behaviour	Graded tasks	Credible source		
1	1	Goal setting (behaviour)	Discrepancy between current behaviour and goal	Feedback on behaviour	self monitoring of behaviour	social support	Instruction on how to perform the behaviour	
1	1	Problem solving	Review of behaviour goals	self monitoring of behaviour	Instruction on how to perform the behaviour	Information about health consequences		
2	2	Goal setting (behaviour)	self monitoring of behaviour	Demonstration of the behaviour	Credible source	Adding object to the environment		
2	2	Goal setting (behaviour)	Action planning	Demonstration of the behaviour	Behavioural practice/rehearsal	Credible source		
2	2	Goal setting (behaviour)	Review of behaviour goals	social support	Instruction on how to perform the behaviour	Behavioural practice/rehearsal		
1	1	Problem solving	Action planning	Review of behaviour goals	self monitoring of behaviour	Instruction on how to perform the behaviour	Information about health consequences	
0	0	Feedback on behaviour	social support	Instruction on how to perform the behaviour	Social comparison	Behavioural practice/rehearsal		
0	0	Feedback on behaviour	self monitoring of behaviour	social support	Information about health consequences	Social comparison		
1	1	Feedback on behaviour	self monitoring of behaviour	social support	Information about health consequences	Behavioural practice/rehearsal		
1	1	Review of behaviour goals	Feedback on behaviour	social support	Instruction on how to perform the behaviour	Graded tasks		
0	0	Action planning	self monitoring of behaviour	Instruction on how to perform the behaviour	Social comparison	Behavioural practice/rehearsal		
0	0	Feedback on behaviour	self monitoring of behaviour	Instruction on how to perform the behaviour	Information about health consequences	Social comparison		
1	1	Review of behaviour goals	self monitoring of behaviour	social support	Instruction on how to perform the behaviour	Information about health consequences		
4	4	Problem solving	Goal setting (outcome)	Action planning	Instruction on how to perform the behaviour	Demonstration of the behaviour	Credible source	
4	4	Problem solving	Goal setting (outcome)	Action planning	Feedback on behaviour	self monitoring of behaviour	Instruction on how to perform the behaviour	
4	4	Problem solving	Goal setting (outcome)	Action planning	self monitoring of behaviour	Instruction on how to perform the behaviour	Credible source	
0	0	self monitoring of behaviour	social support	Instruction on how to perform the behaviour	Social comparison	Behavioural practice/rehearsal		
0	0	social support	Instruction on how to perform the behaviour	Information about health consequences	Social comparison	Behavioural practice/rehearsal		
0	0	Goal setting (behaviour)	Problem solving	Action planning	Review of behaviour goals	Feedback on behaviour	social support	
0	0	Goal setting (behaviour)	Problem solving	Action planning	Review of behaviour goals	social support	Instruction on how to perform the behaviour	
0	0	Feedback on behaviour	self monitoring of behaviour	Instruction on how to perform the behaviour	Social comparison	Behavioural practice/rehearsal		
0	0	Feedback on behaviour	self monitoring of behaviour	social support	Social comparison	Behavioural practice/rehearsal		
1	1	Problem solving	Action planning	Review of behaviour goals	Feedback on behaviour	Instruction on how to perform the behaviour	Information about health consequences	
1	1	Problem solving	Action planning	Review of behaviour goals	Feedback on behaviour	self monitoring of behaviour	Information about health consequences	
2	2	Problem solving	Action planning	Review of behaviour goals	Feedback on behaviour	Instruction on how to perform the behaviour	Credible source	
2	2	Goal setting (behaviour)	self monitoring of behaviour	Instruction on how to perform the behaviour	Demonstration of the behaviour	Prompts/cues	Graded tasks	
1	1	Goal setting (behaviour)	self monitoring of behaviour	Instruction on how to perform the behaviour	Demonstration of the behaviour	Behavioural practice/rehearsal	Graded tasks	
2	2	Problem solving	Action planning	Review of behaviour goals	Feedback on behaviour	self monitoring of behaviour	Credible source	
4	4	Problem solving	Goal setting (outcome)	Action planning	self monitoring of behaviour	Demonstration of the behaviour	Credible source	
1	1	Problem solving	Review of behaviour goals	self monitoring of behaviour	Information about health consequences	Prompts/cues		
0	0	Goal setting (behaviour)	Action planning	social support	Instruction on how to perform the behaviour	Information about social & environmental consequences	Social comparison	
0	0	Goal setting (behaviour)	Action planning	social support	Information about social & environmental consequences	Social comparison	Behavioural practice/rehearsal	
0	0	Goal setting (behaviour)	Action planning	Instruction on how to perform the behaviour	Information about social & environmental consequences	Social comparison	Behavioural practice/rehearsal	
1	1	Problem solving	Review of behaviour goals	Discrepancy between current behaviour and goal	self monitoring of behaviour	Prompts/cues		
1	1	Problem solving	Review of behaviour goals	Discrepancy between current behaviour and goal	Feedback on behaviour	self monitoring of behaviour		
1	1	Problem solving	Review of behaviour goals	Discrepancy between current behaviour and goal	Feedback on behaviour	Prompts/cues		
0	0	Goal setting (behaviour)	Feedback on behaviour	self monitoring of behaviour	social support	Instruction on how to perform the behaviour	Information about social & environmental consequences	
2	2	Problem solving	Action planning	Instruction on how to perform the behaviour	Prompts/cues	Credible source		
2	2	Problem solving	Action planning	self monitoring of behaviour	Behavioural practice/rehearsal	Credible source		
1	1	Problem solving	Review of behaviour goals	Feedback on behaviour	Prompts/cues	Graded tasks		
2	2	Problem solving	Action planning	self monitoring of behaviour	Demonstration of the behaviour	Prompts/cues		
2	2	Problem solving	Action planning	self monitoring of behaviour	Prompts/cues	Credible source		
1	1	Problem solving	Action planning	self monitoring of behaviour	Information about health consequences	Prompts/cues		
1	1	Problem solving	Action planning	Feedback on behaviour	Information about health consequences	Behavioural practice/rehearsal		
1	1	Problem solving	Review of behaviour goals	Feedback on behaviour	Instruction on how to perform the behaviour	Information about health consequences		
0	0	Goal setting (behaviour)	Problem solving	self monitoring of behaviour	social support	Information about health consequences	Social comparison	
2	2	Problem solving	Feedback on behaviour	Instruction on how to perform the behaviour	Prompts/cues	Credible source		
2	2	Problem solving	Feedback on behaviour	Instruction on how to perform the behaviour	Behavioural practice/rehearsal	Graded tasks		

5	3	0	0	0	0	0	0	2	0	0	0	0	0	0	0	0	0	0	0	0	0	5	0	0	0	0	0	0	Action planning	Information abour social & environmental consequences	Social comparison	Behavioural practice/rehearsal			60	0	0	0	0	0	0	4
5	5	3	2	0	0	0	0	0	0	0	0	0	0	0	0	0	0	0	0	0	0	5	3	2	3	2	2	2	Goal setting (behaviour)	Problem solving	self monitoring of behaviour	social support	Demonstration of the behaviour	Adding object to the environment	100	100	100	0	0	0	0	6
5	3	2	1	0	0	0	0	1	2	1	0	0	0	0	0	0	0	0	0	0	0	4	4	2	3	1	2	1	Problem solving	Discrepancy between current behaviour and goal	social support				75	50	50	0	0	0	0	3
5	3	2	1	0	0	0	0	1	2	1	0	0	0	0	0	0	0	0	0	0	0	4	4	2	3	1	2	1	Problem solving	Discrepancy between current behaviour and goal	social support	Instruction on how to perform the behaviour			75	50	50	0	0	0	0	4
5	4	4	3	0	0	0	0	1	0	0	0	0	0	0	0	0	0	0	0	0	0	5	4	3	4	3	3	3	Goal setting (behaviour)	Problem solving	Goal setting (outcome)	Feedback on behaviour	social support	Instruction on how to perform the behaviour	80	100	100	0	0	0	0	6
5	3	0	2	0	0	0	0	1	0	0	0	0	0	0	1	2	0	0	0	0	0	5	2	2	2	2	2	2	Review of behaviour goals	self monitoring of behaviour	Demonstration of the behaviour				75	0	100	0	0	0	0	3
5	2	0	3	0	0	0	0	1	0	0	0	0	0	0	2	3	0	0	0	0	0	5	3	3	3	3	3	3	Review of behaviour goals	Feedback on behaviour	Behavioural practice/rehearsal				67	0	100	0	0	0	0	3
5	4	1	2	0	0	0	0	0	0	0	0	0	0	0	1	1	0	0	0	0	0	5	2	2	2	2	2	2	Action planning	self monitoring of behaviour	Social support (emotional)				100	100	100	0	0	0	0	3
5	2	1	1	0	0	0	0	1	2	1	0	0	0	0	1	1	0	0	0	0	0	4	4	2	3	1	2	1	Action planning	Discrepancy between current behaviour and goal	Information about health consequences				67	33	50	0	0	0	0	3
5	3	0	0	0	0	0	0	2	0	0	0	0	0	0	0	0	0	0	0	0	0	5	0	0	0	0	0	0	Problem solving	self monitoring of behaviour	social support	Information about health consequences	Behavioural practice/rehearsal		60	0	0	0	0	0	0	5
5	2	0	3	0	0	0	0	1	0	0	0	0	0	0	2	3	0	0	0	0	0	5	3	3	3	3	3	3	Action planning	Review of behaviour goals	Instruction on how to perform the behaviour	Demonstration of the behaviour	Behavioural practice/rehearsal		67	0	100	0	0	0	0	5
5	4	1	0	0	0	0	0	1	0	0	0	0	0	0	0	0	0	0	0	0	0	5	1	0	1	0	0	0	Goal setting (behaviour)	Problem solving	self monitoring of behaviour	social support	Information about health consequences	Information abour social & environmental consequences	80	100	0	0	0	0	0	6
5	3	0	0	0	0	0	0	2	0	0	0	0	0	0	0	0	0	0	0	0	0	5	0	0	0	0	0	0	Goal setting (behaviour)	Problem solving	self monitoring of behaviour	social support	Information about health consequences	Behavioural practice/rehearsal	60	0	0	0	0	0	0	6
5	4	1	0	0	0	0	0	1	1	0	0	0	0	0	0	0	0	0	0	0	0	5	2	0	2	0	0	0	Goal setting (behaviour)	Problem solving	self monitoring of behaviour	social support	Information about health consequences	Credible source	80	50	0	0	0	0	0	6
5	4	0	0	0	0	0	0	1	0	0	0	0	0	0	0	0	0	0	0	0	0	5	0	0	0	0	0	0	Problem solving	self monitoring of behaviour	social support	Demonstration of the behaviour	Behavioural practice/rehearsal		80	0	0	0	0	0	0	5
5	4	0	0	0	0	0	0	1	0	0	0	0	0	0	0	0	0	0	0	0	0	5	0	0	0	0	0	0	Problem solving	Action planning	Instruction on how to perform the behaviour	Information about health consequences	Information abour social & environmental consequences		80	0	0	0	0	0	0	5
5	4	0	0	0	0	0	0	1	0	0	0	0	0	0	0	0	0	0	0	0	0	5	0	0	0	0	0	0	Problem solving	Action planning	Instruction on how to perform the behaviour	Information abour social & environmental consequences	Behavioural practice/rehearsal		80	0	0	0	0	0	0	5
5	2	0	0	0	0	0	0	3	0	0	0	0	0	0	0	0	0	0	0	0	0	5	0	0	0	0	0	0	Action planning	Feedback on behaviour	social support	Instruction on how to perform the behaviour	Social comparison	Behavioural practice/rehearsal	40	0	0	0	0	0	0	6
5	2	0	1	0	0	0	0	2	0	0	0	0	0	0	1	1	0	0	0	0	0	5	1	1	1	1	1	1	Review of behaviour goals	Feedback on behaviour	self monitoring of behaviour	social support	Instruction on how to perform the behaviour	Graded tasks	50	0	100	0	0	0	0	6
5	3	0	0	0	0	0	0	2	0	0	0	0	0	0	0	0	0	0	0	0	0	5	0	0	0	0	0	0	Action planning	social support	Instruction on how to perform the behaviour	Information about health consequences	Social comparison	Behavioural practice/rehearsal	60	0	0	0	0	0	0	6
5	2	0	1	0	0	0	0	2	0	0	0	0	0	0	1	1	0	0	0	0	0	5	1	1	1	1	1	1	Action planning	social support	Instruction on how to perform the behaviour	Demonstration of the behaviour	Behavioural practice/rehearsal		50	0	100	0	0	0	0	5
5	2	0	3	0	0	0	0	1	0	0	0	0	0	0	2	3	0	0	0	0	0	5	3	3	3	3	3	3	Review of behaviour goals	Instruction on how to perform the behaviour	Demonstration of the behaviour	Behavioural practice/rehearsal			67	0	100	0	0	0	0	4
5	5	3	2	0	0	0	0	0	0	0	0	0	0	0	0	0	0	0	0	0	0	5	3	2	3	2	2	2	Problem solving	self monitoring of behaviour	social support	Demonstration of the behaviour	Adding object to the environment		100	100	100	0	0	0	0	5
5	4	0	0	0	0	0	0	0	1	1	0	0	0	0	0	0	0	0	0	0	0	4	1	1	0	0	1	0	Problem solving	self monitoring of behaviour	social support	Behavioural practice/rehearsal	Credible source		100	0	0	0	0	0	0	5
5	4	1	0	0	0	0	0	1	0	0	0	0	0	0	0	0	0	0	0	0	0	5	1	0	1	0	0	0	Problem solving	self monitoring of behaviour	social support	Information about health consequences	Information abour social & environmental consequences		80	100	0	0	0	0	0	5
5	3	0	0	0	0	0	0	2	0	0	0	0	0	0	0	0	0	0	0	0	0	5	0	0	0	0	0	0	Action planning	Instruction on how to perform the behaviour	Information abour social & environmental consequences	Social comparison			60	0	0	0	0	0	0	4
5	2	1	1	0	0	0	0	2	2	1	0	0	0	0	0	0	0	0	0	0	0	4	3	2	2	1	2	1	Action planning	Discrepancy between current behaviour and goal	social support	Credible source			50	33	50	0	0	0	0	4
5	1	0	2	0	0	0	0	2	0	0	0	0	0	0	2	2	0	0	0	0	0	5	2	2	2	2	2	2	Action planning	Feedback on behaviour	self monitoring of behaviour	Information about health consequences	Graded tasks		33	0	100	0	0	0	0	5
5	3	1	1	0	0	0	0	1	1	0	0	0	0	0	1	1	0	0	0	0	0	5	3	1	3	1	1	1	Action planning	Feedback on behaviour	self monitoring of behaviour	Information about health consequences	Credible source		75	50	100	0	0	0	0	5
5	3	2	1	0	0	0	0	2	1	0	0	0	0	0	0	0	0	0	0	0	0	5	3	1	3	1	1	1	Discrepancy between current behaviour and goal	self monitoring of behaviour	social support	Instruction on how to perform the behaviour			60	67	100	0	0	0	0	4

1	1	Action planning	Review of behaviour goals	self monitoring of behaviour	social support	Instruction on how to perform the behaviour	Information about health consequences	Graded tasks	
1	1	Goal setting (behaviour)	Problem solving	Action planning	Review of behaviour goals	Instruction on how to perform the behaviour	Credible source		
0	0	Feedback on behaviour	self monitoring of behaviour	social support	Instruction on how to perform the behaviour	Information about health consequences	Social comparison	Behavioural practice/rehearsal	
0	0	Goal setting (behaviour)	Problem solving	Action planning	Review of behaviour goals	Feedback on behaviour	self monitoring of behaviour	social support	Information about health consequences
0	0	Goal setting (behaviour)	Problem solving	Action planning	Review of behaviour goals	Feedback on behaviour	self monitoring of behaviour	social support	Graded tasks
0	0	Goal setting (behaviour)	Problem solving	Action planning	Review of behaviour goals	Feedback on behaviour	social support	Instruction on how to perform the behaviour	Information about health consequences
0	0	Goal setting (behaviour)	Problem solving	Action planning	Review of behaviour goals	Feedback on behaviour	social support	Instruction on how to perform the behaviour	Graded tasks
0	0	Goal setting (behaviour)	Problem solving	Action planning	Feedback on behaviour	self monitoring of behaviour	Instruction on how to perform the behaviour	Social comparison	Behavioural practice/rehearsal
0	0	Goal setting (behaviour)	Problem solving	Action planning	Feedback on behaviour	self monitoring of behaviour	Information about health consequences	Social comparison	Behavioural practice/rehearsal
0	0	Goal setting (behaviour)	Problem solving	Action planning	Feedback on behaviour	social support	Instruction on how to perform the behaviour	Information about health consequences	Behavioural practice/rehearsal
1	1	Goal setting (behaviour)	Problem solving	Action planning	Review of behaviour goals	Feedback on behaviour	Credible source		
1	1	Goal setting (behaviour)	Problem solving	Action planning	Review of behaviour goals	self monitoring of behaviour	Demonstration of the behaviour		
0	0	Goal setting (behaviour)	Problem solving	Action planning	Review of behaviour goals	self monitoring of behaviour	social support	Instruction on how to perform the behaviour	Information about health consequences
1	1	Goal setting (behaviour)	Problem solving	Action planning	Review of behaviour goals	self monitoring of behaviour	Behavioural practice/rehearsal		
1	1	Goal setting (behaviour)	Problem solving	Action planning	Review of behaviour goals	self monitoring of behaviour	Credible source		
1	1	Goal setting (behaviour)	Problem solving	Action planning	Review of behaviour goals	Instruction on how to perform the behaviour	Prompts/cues		
1	1	Goal setting (behaviour)	Problem solving	Action planning	Review of behaviour goals	Feedback on behaviour	Prompts/cues		
0	0	Goal setting (behaviour)	Problem solving	Action planning	Feedback on behaviour	self monitoring of behaviour	social support	Information about health consequences	Behavioural practice/rehearsal
1	1	Goal setting (behaviour)	Problem solving	Action planning	Discrepancy between current behaviour and goal	Information about health consequences	Prompts/cues		
1	1	Goal setting (behaviour)	Problem solving	Action planning	Discrepancy between current behaviour and goal	Information about health consequences	Credible source		
1	1	Goal setting (behaviour)	Problem solving	Action planning	Discrepancy between current behaviour and goal	Prompts/cues	Credible source		
1	1	Goal setting (behaviour)	Problem solving	Action planning	Discrepancy between current behaviour and goal	self monitoring of behaviour	Information about health consequences		
1	1	Goal setting (behaviour)	Problem solving	Action planning	Feedback on behaviour	Information about health consequences	Graded tasks		
1	1	Goal setting (behaviour)	Problem solving	Action planning	Instruction on how to perform the behaviour	Information about health consequences	Graded tasks		
1	1	Goal setting (behaviour)	Problem solving	Action planning	Instruction on how to perform the behaviour	Demonstration of the behaviour	Behavioural practice/rehearsal		
1	1	Goal setting (behaviour)	Problem solving	Action planning	self monitoring of behaviour	Behavioural practice/rehearsal	Credible source		
3	3	Goal setting (behaviour)	Problem solving	Goal setting (outcome)	Action planning	Feedback on behaviour	Instruction on how to perform the behaviour		
1	1	Instruction on how to perform the behaviour	Information about health consequences	Demonstration of the behaviour	Behavioural practice/rehearsal	Credible source			
0	0	Goal setting (behaviour)	Problem solving	Review of behaviour goals	Feedback on behaviour	self monitoring of behaviour	social support	Instruction on how to perform the behaviour	Graded tasks
1	1	Goal setting (behaviour)	Problem solving	Action planning	Discrepancy between current behaviour and goal	Instruction on how to perform the behaviour	Information about health consequences		
1	1	Goal setting (behaviour)	Problem solving	Action planning	Discrepancy between current behaviour and goal	Instruction on how to perform the behaviour	Prompts/cues		
1	1	Goal setting (behaviour)	Problem solving	Action planning	Discrepancy between current behaviour and goal	Feedback on behaviour	Information about health consequences		

2	2	Action planning	Review of behaviour goals	Instruction on how to perform the behaviour	Graded tasks	Credible source	
2	2	Action planning	Review of behaviour goals	Demonstration of the behaviour	Behavioural practice/rehearsal	Graded tasks	
1	0	social support	Instruction on how to perform the behaviour	Social comparison	Credible source		
0	0	self monitoring of behaviour	social support	Information abour social & environmental consequences	Social comparison		
0	0	self monitoring of behaviour	Instruction on how to perform the behaviour	Information abour social & environmental consequences	Social comparison		
1	1	Action planning	Discrepancy between current behaviour and goal	Feedback on behaviour	Instruction on how to perform the behaviour	Information about health consequences	
1	1	Action planning	Discrepancy between current behaviour and goal	Feedback on behaviour	Instruction on how to perform the behaviour	Prompts/cues	
1	1	Action planning	Discrepancy between current behaviour and goal	self monitoring of behaviour	Instruction on how to perform the behaviour	Prompts/cues	
1	1	Action planning	Review of behaviour goals	Feedback on behaviour	social support	Information about health consequences	Graded tasks
0	0	Problem solving	self monitoring of behaviour	social support	Instruction on how to perform the behaviour	Information about health consequences	Graded tasks
0	0	Problem solving	self monitoring of behaviour	social support	Instruction on how to perform the behaviour	Social comparison	Behavioural practice/rehearsal
0	0	Problem solving	self monitoring of behaviour	social support	Information about health consequences	Social comparison	Behavioural practice/rehearsal
0	0	Problem solving	self monitoring of behaviour	Instruction on how to perform the behaviour	Information about health consequences	Social comparison	Behavioural practice/rehearsal
1	1	Action planning	Discrepancy between current behaviour and goal	Feedback on behaviour	Information about health consequences	Credible source	
1	1	Action planning	Discrepancy between current behaviour and goal	Feedback on behaviour	self monitoring of behaviour	Information about health consequences	
1	1	Action planning	Discrepancy between current behaviour and goal	Feedback on behaviour	self monitoring of behaviour	Prompts/cues	
1	1	Goal setting (behaviour)	Problem solving	self monitoring of behaviour	social support	Graded tasks	Social reward
1	1	Goal setting (behaviour)	Problem solving	self monitoring of behaviour	social support	Social reward	Adding object to the environment
1	1	Goal setting (behaviour)	Problem solving	self monitoring of behaviour	Instruction on how to perform the behaviour	Prompts/cues	Social reward
1	1	Goal setting (behaviour)	Problem solving	self monitoring of behaviour	Instruction on how to perform the behaviour	Prompts/cues	Adding object to the environment
1	1	Goal setting (behaviour)	Problem solving	social support	Prompts/cues	Social reward	Adding object to the environment
1	1	Goal setting (behaviour)	Problem solving	social support	Graded tasks	Social reward	Adding object to the environment
1	1	Goal setting (behaviour)	Problem solving	self monitoring of behaviour	Instruction on how to perform the behaviour	Social reward	Adding object to the environment
2	2	Goal setting (behaviour)	Action planning	Feedback on behaviour	self monitoring of behaviour	Behavioural practice/rehearsal	Graded tasks
0	0	Feedback on behaviour	self monitoring of behaviour	Information abour social & environmental consequences	Social comparison		
2	2	Action planning	Review of behaviour goals	Instruction on how to perform the behaviour	Demonstration of the behaviour	Graded tasks	
0	0	Feedback on behaviour	self monitoring of behaviour	Information abour social & environmental consequences	Behavioural practice/rehearsal		
0	0	Feedback on behaviour	social support	Information abour social & environmental consequences	Social comparison		
0	0	Feedback on behaviour	social support	Information abour social & environmental consequences	Behavioural practice/rehearsal		
0	0	Feedback on behaviour	Instruction on how to perform the behaviour	Information abour social & environmental consequences	Social comparison		
1	1	Action planning	Discrepancy between current behaviour and goal	Feedback on behaviour	Information about health consequences	Prompts/cues	
1	1	Goal setting (behaviour)	Problem solving	self monitoring of behaviour	social support	Prompts/cues	Adding object to the environment
1	1	Action planning	Review of behaviour goals	self monitoring of behaviour	Instruction on how to perform the behaviour	Prompts/cues	
1	1	Action planning	Review of behaviour goals	self monitoring of behaviour	Information about health consequences	Prompts/cues	
2	2	Action planning	Review of behaviour goals	Feedback on behaviour	self monitoring of behaviour	Demonstration of the behaviour	
1	1	Action planning	Review of behaviour goals	Feedback on behaviour	self monitoring of behaviour	Prompts/cues	
2	2	Action planning	Review of behaviour goals	self monitoring of behaviour	Graded tasks	Credible source	
1	1	Action planning	Review of behaviour goals	Instruction on how to perform the behaviour	Information about health consequences	Prompts/cues	
0	0	Feedback on behaviour	Instruction on how to perform the behaviour	Information abour social & environmental consequences	Behavioural practice/rehearsal		
0	0	Feedback on behaviour	Information abour social & environmental consequences	Social comparison	Behavioural practice/rehearsal		
1	1	Goal setting (behaviour)	Action planning	Discrepancy between current behaviour and goal	Information about health consequences	Prompts/cues	Credible source
1	1	Goal setting (behaviour)	Action planning	Discrepancy between current behaviour and goal	self monitoring of behaviour	Information about health consequences	Credible source
1	1	Goal setting (behaviour)	Problem solving	self monitoring of behaviour	Instruction on how to perform the behaviour	Graded tasks	Social reward
1	1	Goal setting (behaviour)	Problem solving	self monitoring of behaviour	social support	Instruction on how to perform the behaviour	Social reward
4	2	Problem solving	Goal setting (outcome)	Feedback on outcomes of behaviour	Instruction on how to perform the behaviour	Credible source	
1	1	Goal setting (behaviour)	Problem solving	social support	Instruction on how to perform the behaviour	Prompts/cues	Social reward
1	1	Goal setting (behaviour)	Problem solving	social support	Instruction on how to perform the behaviour	Prompts/cues	Adding object to the environment
2	2	Action planning	Review of behaviour goals	self monitoring of behaviour	Instruction on how to perform the behaviour	Demonstration of the behaviour	
0	0	Goal setting (behaviour)	Action planning	self monitoring of behaviour	Information abour social & environmental consequences	Social comparison	
1	1	Goal setting (behaviour)	Action planning	Discrepancy between current behaviour and goal	Feedback on behaviour	social support	
2	2	Action planning	Review of behaviour goals	self monitoring of behaviour	Demonstration of the behaviour	Graded tasks	
1	1	Action planning	Review of behaviour goals	self monitoring of behaviour	Prompts/cues	Graded tasks	
1	1	Goal setting (behaviour)	Action planning	Feedback on behaviour	Instruction on how to perform the behaviour	Demonstration of the behaviour	Behavioural practice/rehearsal
2	2	Goal setting (behaviour)	Action planning	Feedback on behaviour	Instruction on how to perform the behaviour	Behavioural practice/rehearsal	Graded tasks
1	1	Goal setting (behaviour)	Action planning	Discrepancy between current behaviour and goal	self monitoring of behaviour	Prompts/cues	Credible source
1	1	Goal setting (behaviour)	Action planning	Discrepancy between current behaviour and goal	Instruction on how to perform the behaviour	Information about health consequences	Prompts/cues
0	0	Goal setting (behaviour)	Action planning	Feedback on behaviour	self monitoring of behaviour	Information abour social & environmental consequences	
0	0	Goal setting (behaviour)	Action planning	Feedback on behaviour	social support	Information abour social & environmental consequences	
0	0	self monitoring of behaviour	Information abour social & environmental consequences	Social comparison	Behavioural practice/rehearsal		
1	0	social support	social support (practical	Instruction on how to perform the behaviour	Credible source		
2	2	Action planning	Review of behaviour goals	Feedback on behaviour	Graded tasks	Credible source	
0	0	Goal setting (behaviour)	Action planning	Feedback on behaviour	Instruction on how to perform the behaviour	Information abour social & environmental consequences	
0	0	Goal setting (behaviour)	Action planning	Feedback on behaviour	Information abour social & environmental consequences	Social comparison	
0	0	Goal setting (behaviour)	Action planning	Feedback on behaviour	Information abour social & environmental consequences	Behavioural practice/rehearsal	
0	0	Goal setting (behaviour)	Feedback on behaviour	self monitoring of behaviour	Information abour social & environmental consequences	Social comparison	
0	0	Goal setting (behaviour)	Feedback on behaviour	self monitoring of behaviour	Information abour social & environmental consequences	Behavioural practice/rehearsal	
0	0	Goal setting (behaviour)	Feedback on behaviour	social support	Information abour social & environmental consequences	Social comparison	
0	0	Goal setting (behaviour)	Feedback on behaviour	social support	Information abour social & environmental consequences	Behavioural practice/rehearsal	
0	0	Goal setting (behaviour)	Feedback on behaviour	Instruction on how to perform the behaviour	Information abour social & environmental consequences	Social comparison	
0	0	Goal setting (behaviour)	Feedback on behaviour	Instruction on how to perform the behaviour	Information abour social & environmental consequences	Behavioural practice/rehearsal	
1	1	Goal setting (behaviour)	Action planning	Discrepancy between current behaviour and goal	self monitoring of behaviour	social support	
1	1	Goal setting (behaviour)	Action planning	Discrepancy between current behaviour and goal	social support	Credible source	
1	1	Goal setting (behaviour)	Action planning	Feedback on behaviour	self monitoring of behaviour	Behavioural practice/rehearsal	Credible source
0	0	Problem solving	Feedback on behaviour	self monitoring of behaviour	Information about health consequences	Social comparison	Behavioural practice/rehearsal
0	0	Problem solving	Feedback on behaviour	social support	Instruction on how to perform the behaviour	Information about health consequences	Behavioural practice/rehearsal
1	1	Goal setting (behaviour)	Action planning	Feedback on behaviour	Instruction on how to perform the behaviour	Behavioural practice/rehearsal	Credible source
1	1	Goal setting (behaviour)	Action planning	Feedback on behaviour	Information about health consequences	Prompts/cues	Credible source
1	1	Goal setting (behaviour)	Discrepancy between current behaviour and goal	self monitoring of behaviour	social support	Credible source	
1	1	Goal setting (behaviour)	Discrepancy between current behaviour and goal	social support	Instruction on how to perform the behaviour	Demonstration of the behaviour	
1	1	Goal setting (behaviour)	Discrepancy between current behaviour and goal	social support	Instruction on how to perform the behaviour	Credible source	
0	0	Goal setting (behaviour)	self monitoring of behaviour	Instruction on how to perform the behaviour	Information about health consequences	Social comparison	Behavioural practice/rehearsal
0	0	Problem solving	Action planning	self monitoring of behaviour	social support	Social comparison	Behavioural practice/rehearsal
0	0	Problem solving	Action planning	self monitoring of behaviour	Instruction on how to perform the behaviour	Social comparison	Behavioural practice/rehearsal
0	0	Problem solving	Action planning	Feedback on behaviour	social support	Information about health consequences	Behavioural practice/rehearsal
0	0	Problem solving	Action planning	Feedback on behaviour	social support	Social comparison	Behavioural practice/rehearsal
1	1	Goal setting (behaviour)	Action planning	Instruction on how to perform the behaviour	Information about health consequences	Behavioural practice/rehearsal	Credible source
2	2	Goal setting (behaviour)	Action planning	self monitoring of behaviour	Demonstration of the behaviour	Prompts/cues	Graded tasks
1	1	Action planning	Discrepancy between current behaviour and goal	Feedback on behaviour	self monitoring of behaviour	social support	
0	0	Problem solving	Feedback on behaviour	social support	Instruction on how to perform the behaviour	Social comparison	Behavioural practice/rehearsal
0	0	Problem solving	Feedback on behaviour	social support	Information about health consequences	Social comparison	Behavioural practice/rehearsal
0	0	Problem solving	Feedback on behaviour	Instruction on how to perform the behaviour	Information about health consequences	Social comparison	Behavioural practice/rehearsal
0	0	Problem solving	Feedback on behaviour	self monitoring of behaviour	social support	Information about health consequences	Behavioural practice/rehearsal
0	0	Problem solving	Feedback on behaviour	self monitoring of behaviour	social support	Social comparison	Behavioural practice/rehearsal
0	0	Problem solving	Action planning	Review of behaviour goals	social support	Instruction on how to perform the behaviour	Information about health consequences
0	0	Problem solving	Action planning	Review of behaviour goals	social support	Instruction on how to perform the behaviour	Graded tasks
0	0	Problem solving	Action planning	Feedback on behaviour	self monitoring of behaviour	Social comparison	Behavioural practice/rehearsal
1	1	Goal setting (behaviour)	Action planning	self monitoring of behaviour	Information about health consequences	Prompts/cues	Credible source
0	0	Problem solving	Feedback on behaviour	self monitoring of behaviour	Instruction on how to perform the behaviour	Social comparison	Behavioural practice/rehearsal
0	0	Problem solving	Review of behaviour goals	Feedback on behaviour	self monitoring of behaviour	social support	Graded tasks
0	0	Problem solving	Review of behaviour goals	Feedback on behaviour	social support	Instruction on how to perform the behaviour	Information about health consequences
0	0	Problem solving	Review of behaviour goals	Feedback on behaviour	social support	Instruction on how to perform the behaviour	Graded tasks
0	0	Problem solving	Review of behaviour goals	self monitoring of behaviour	social support	Instruction on how to perform the behaviour	Information about health consequences
0	0	Problem solving	Review of behaviour goals	self monitoring of behaviour	social support	Instruction on how to perform the behaviour	Graded tasks
0	0	Problem solving	Action planning	self monitoring of behaviour	Information about health consequences	Social comparison	Behavioural practice/rehearsal
1	1	Goal setting (behaviour)	Action planning	Discrepancy between current behaviour and goal	Instruction on how to perform the behaviour	Information about health consequences	Credible source
1	1	Goal setting (behaviour)	Action planning	Discrepancy between current behaviour and goal	Feedback on behaviour	Prompts/cues	Credible source
1	1	Goal setting (behaviour)	Action planning	Discrepancy between current behaviour and goal	self monitoring of behaviour	Instruction on how to perform the behaviour	Information about health consequences
1	1	Goal setting (behaviour)	Action planning	Discrepancy between current behaviour and goal	self monitoring of behaviour	Instruction on how to perform the behaviour	Prompts/cues
1	1	Goal setting (behaviour)	Action planning	Discrepancy between current behaviour and goal	self monitoring of behaviour	Information about health consequences	Prompts/cues
2	2	Goal setting (behaviour)	Action planning	self monitoring of behaviour	Instruction on how to perform the behaviour	Behavioural practice/rehearsal	Graded tasks
2	2	Goal setting (behaviour)	Action planning	Feedback on behaviour	Demonstration of the behaviour	Prompts/cues	Graded tasks
0	0	Problem solving	Review of behaviour goals	Feedback on behaviour	self monitoring of behaviour	social support	Information about health consequences
3	3	Goal setting (behaviour)	Action planning	Feedback on behaviour	Demonstration of the behaviour	Graded tasks	Credible source
1	1	Goal setting (behaviour)	Review of behaviour goals	Discrepancy between current behaviour and goal	Feedback on behaviour	self monitoring of behaviour	Information about health consequences
1	1	Goal setting (behaviour)	Review of behaviour goals	Discrepancy between current behaviour and goal	Feedback on behaviour	self monitoring of behaviour	Graded tasks
2	2	Goal setting (behaviour)	Action planning	Instruction on how to perform the behaviour	Demonstration of the behaviour	Prompts/cues	Graded tasks
3	3	Goal setting (behaviour)	Action planning	Instruction on how to perform the behaviour	Demonstration of the behaviour	Graded tasks	Credible source
1	1	Goal setting (behaviour)	Action planning	Instruction on how to perform the behaviour	Behavioural practice/rehearsal	Generalisation of target behaviour	Credible source
1	1	Goal setting (behaviour)	Action planning	Instruction on how to perform the behaviour	Information about health consequences	Prompts/cues	Credible source
0	0	Goal setting (behaviour)	self monitoring of behaviour	social support	Information about health consequences	Social comparison	Behavioural practice/rehearsal
1	1	Goal setting (behaviour)	Discrepancy between current behaviour and goal	Feedback on behaviour	social support	Credible source	
1	1	Action planning	Review of behaviour goals	Feedback on behaviour	Information about health consequences	Prompts/cues	
2	2	Action planning	Review of behaviour goals	Feedback on behaviour	Demonstration of the behaviour	Behavioural practice/rehearsal	
0	0	Goal setting (behaviour)	Feedback on behaviour	Information abour social & environmental consequences	Social comparison	Behavioural practice/rehearsal	
1	1	Goal setting (behaviour)	Action planning	self monitoring of behaviour	Demonstration of the behaviour	Behavioural practice/rehearsal	Credible source
3	3	Goal setting (behaviour)	Action planning	self monitoring of behaviour	Demonstration of the behaviour	Graded tasks	Credible source
0	0	Problem solving	Action planning	Feedback on behaviour	Instruction on how to perform the behaviour	Social comparison	Behavioural practice/rehearsal
0	0	Problem solving	Action planning	Feedback on behaviour	Information about health consequences	Social comparison	Behavioural practice/rehearsal
0	0	Action planning	Feedback on behaviour	social support	Information abour social & environmental consequences	Social comparison	
0	0	Action planning	Feedback on behaviour	social support	Information abour social & environmental consequences	Behavioural practice/rehearsal	
2	2	Action planning	Feedback on behaviour	social support	Demonstration of the behaviour	Credible source	
0	0	Action planning	Feedback on behaviour	self monitoring of behaviour	social support	Information abour social & environmental consequences	
0	0	Action planning	Feedback on behaviour	self monitoring of behaviour	Instruction on how to perform the behaviour	Information abour social & environmental consequences	
2	2	Action planning	Review of behaviour goals	Feedback on behaviour	Demonstration of the behaviour	Graded tasks	
2	2	Action planning	Review of behaviour goals	Feedback on behaviour	Instruction on how to perform the behaviour	Demonstration of the behaviour	
1	1	Action planning	Review of behaviour goals	Feedback on behaviour	Instruction on how to perform the behaviour	Prompts/cues	
1	1	Action planning	Discrepancy between current behaviour and goal	Feedback on behaviour	social support	Instruction on how to perform the behaviour	
1	1	Action planning	Discrepancy between current behaviour and goal	Feedback on behaviour	social support	Credible source	
1	1	Action planning	Discrepancy between current behaviour and goal	self monitoring of behaviour	social support	Instruction on how to perform the behaviour	
1	1	Action planning	Discrepancy between current behaviour and goal	self monitoring of behaviour	social support	Credible source	
2	1	Action planning	Discrepancy between current behaviour and goal	social support	Instruction on how to perform the behaviour	Demonstration of the behaviour	
0	0	Action planning	self monitoring of behaviour	social support	Information abour social & environmental consequences	Social comparison	
0	0	Action planning	self monitoring of behaviour	Instruction on how to perform the behaviour	Information abour social & environmental consequences	Social comparison	
0	0	Action planning	Feedback on behaviour	social support	Instruction on how to perform the behaviour	Information abour social & environmental consequences	
4	4	Goal setting (outcome)	Action planning	Instruction on how to perform the behaviour	Demonstration of the behaviour	Graded tasks	
4	4	Goal setting (outcome)	Action planning	Instruction on how to perform the behaviour	Graded tasks	Credible source	
4	4	Goal setting (outcome)	Action planning	Demonstration of the behaviour	Graded tasks	Credible source	
4	4	Goal setting (outcome)	Action planning	self monitoring of behaviour	Instruction on how to perform the behaviour	Graded tasks	
0	0	Action planning	Feedback on behaviour	self monitoring of behaviour	Information abour social & environmental consequences	Social comparison	
0	0	Action planning	Feedback on behaviour	self monitoring of behaviour	Information abour social & environmental consequences	Behavioural practice/rehearsal	

2	2	Problem solving	Review of behaviour goals	Instruction on how to perform the behaviour	Graded tasks	Credible source	
1	1	Problem solving	Review of behaviour goals	Information about health consequences	Prompts/cues	Graded tasks	
1	1	Problem solving	Action planning	Review of behaviour goals	Feedback on behaviour	self monitoring of behaviour	Prompts/cues
2	2	Problem solving	Action planning	Review of behaviour goals	Feedback on behaviour	self monitoring of behaviour	Behavioural practice/rehearsal
1	1	Feedback on behaviour	self monitoring of behaviour	social support	Information about health consequences	Graded tasks	
1	1	Problem solving	Review of behaviour goals	Discrepancy between current behaviour and goal	self monitoring of behaviour	Information about health consequences	
1	1	Problem solving	Review of behaviour goals	Discrepancy between current behaviour and goal	self monitoring of behaviour	Graded tasks	
1	1	Problem solving	Review of behaviour goals	Discrepancy between current behaviour and goal	Feedback on behaviour	Information about health consequences	
1	1	Goal setting (behaviour)	Discrepancy between current behaviour and goal	self monitoring of behaviour	social support	Instruction on how to perform the behaviour	Credible source
0	0	Goal setting (behaviour)	Feedback on behaviour	self monitoring of behaviour	social support	Information about social & environmental consequences	Social comparison
1	1	Problem solving	Action planning	Review of behaviour goals	self monitoring of behaviour	Prompts/cues	Graded tasks
2	2	Problem solving	Action planning	Review of behaviour goals	self monitoring of behaviour	Behavioural practice/rehearsal	Graded tasks
2	2	Problem solving	Action planning	Review of behaviour goals	self monitoring of behaviour	Graded tasks	Credible source
1	1	Problem solving	Action planning	Review of behaviour goals	Instruction on how to perform the behaviour	Information about health consequences	Prompts/cues
1	1	Problem solving	Action planning	Review of behaviour goals	Instruction on how to perform the behaviour	Information about health consequences	Graded tasks
1	1	Problem solving	Action planning	Discrepancy between current behaviour and goal	Feedback on behaviour	Instruction on how to perform the behaviour	Information about health consequences
1	1	Problem solving	Action planning	Discrepancy between current behaviour and goal	Feedback on behaviour	Instruction on how to perform the behaviour	Prompts/cues
2	2	Problem solving	Action planning	Review of behaviour goals	Instruction on how to perform the behaviour	Graded tasks	Credible source
2	2	Problem solving	Action planning	Review of behaviour goals	self monitoring of behaviour	Instruction on how to perform the behaviour	Behavioural practice/rehearsal
1	1	Problem solving	Action planning	Review of behaviour goals	self monitoring of behaviour	Information about health consequences	Prompts/cues
1	1	Problem solving	Action planning	Review of behaviour goals	self monitoring of behaviour	Information about health consequences	Graded tasks
2	2	Problem solving	Action planning	Review of behaviour goals	self monitoring of behaviour	Demonstration of the behaviour	Graded tasks
2	2	Problem solving	Action planning	Review of behaviour goals	Feedback on behaviour	Behavioural practice/rehearsal	Graded tasks
2	2	Problem solving	Action planning	Review of behaviour goals	Instruction on how to perform the behaviour	Demonstration of the behaviour	Behavioural practice/rehearsal
2	2	Problem solving	Action planning	Review of behaviour goals	Instruction on how to perform the behaviour	Demonstration of the behaviour	Graded tasks
2	2	Problem solving	Action planning	Review of behaviour goals	Instruction on how to perform the behaviour	Behavioural practice/rehearsal	Graded tasks
1	1	Feedback on behaviour	social support	Instruction on how to perform the behaviour	Information about health consequences	Graded tasks	
0	0	Feedback on behaviour	social support	Information about health consequences	Social comparison	Behavioural practice/rehearsal	
0	0	Feedback on behaviour	Instruction on how to perform the behaviour	Information about health consequences	Social comparison	Behavioural practice/rehearsal	
0	0	Action planning	self monitoring of behaviour	Information about health consequences	Social comparison	Behavioural practice/rehearsal	
0	0	Action planning	Feedback on behaviour	Information about health consequences	Social comparison	Behavioural practice/rehearsal	
2	2	Problem solving	Action planning	Review of behaviour goals	Feedback on behaviour	Graded tasks	Credible source
2	2	Problem solving	Action planning	Review of behaviour goals	self monitoring of behaviour	Instruction on how to perform the behaviour	Demonstration of the behaviour
1	1	Problem solving	Action planning	Review of behaviour goals	self monitoring of behaviour	Instruction on how to perform the behaviour	Prompts/cues
1	1	Problem solving	Action planning	Information about health consequences	Prompts/cues	Credible source	
2	2	Problem solving	Action planning	Demonstration of the behaviour	Behavioural practice/rehearsal	Graded tasks	
2	2	Problem solving	Action planning	Demonstration of the behaviour	Behavioural practice/rehearsal	Credible source	
2	2	Problem solving	Action planning	Instruction on how to perform the behaviour	Behavioural practice/rehearsal	Graded tasks	
1	1	Goal setting (behaviour)	Discrepancy between current behaviour and goal	Feedback on behaviour	self monitoring of behaviour	social support	Credible source
1	1	Review of behaviour goals	Feedback on behaviour	social support	Information about health consequences	Graded tasks	
1	1	Review of behaviour goals	self monitoring of behaviour	social support	Information about health consequences	Graded tasks	
1	1	Review of behaviour goals	social support	Instruction on how to perform the behaviour	Information about health consequences	Graded tasks	
1	1	Problem solving	Action planning	self monitoring of behaviour	Information about health consequences	Graded tasks	
0	0	Goal setting (behaviour)	self monitoring of behaviour	social support	Instruction on how to perform the behaviour	Information about social & environmental consequences	Social comparison
0	0	Goal setting (behaviour)	self monitoring of behaviour	social support	Information about social & environmental consequences	Social comparison	Behavioural practice/rehearsal
0	0	Goal setting (behaviour)	self monitoring of behaviour	Instruction on how to perform the behaviour	Information about social & environmental consequences	Social comparison	Behavioural practice/rehearsal
1	1	Action planning	Discrepancy between current behaviour and goal	Feedback on behaviour	self monitoring of behaviour	social support	Instruction on how to perform the behaviour
1	1	Action planning	Discrepancy between current behaviour and goal	Feedback on behaviour	self monitoring of behaviour	social support	Credible source
1	1	Action planning	Discrepancy between current behaviour and goal	Feedback on behaviour	social support	Instruction on how to perform the behaviour	Credible source
1	1	Problem solving	Action planning	Discrepancy between current behaviour and goal	self monitoring of behaviour	Information about health consequences	Prompts/cues
0	0	Goal setting (behaviour)	Feedback on behaviour	self monitoring of behaviour	Instruction on how to perform the behaviour	Information about social & environmental consequences	Social comparison
0	0	Goal setting (behaviour)	Feedback on behaviour	self monitoring of behaviour	Instruction on how to perform the behaviour	Information about social & environmental consequences	Behavioural practice/rehearsal
0	0	Goal setting (behaviour)	Feedback on behaviour	self monitoring of behaviour	Information about social & environmental consequences	Social comparison	Behavioural practice/rehearsal
0	0	Goal setting (behaviour)	Feedback on behaviour	social support	Instruction on how to perform the behaviour	Information about social & environmental consequences	Social comparison
0	0	Goal setting (behaviour)	Feedback on behaviour	social support	Instruction on how to perform the behaviour	Information about social & environmental consequences	Behavioural practice/rehearsal
0	0	Goal setting (behaviour)	Feedback on behaviour	social support	Information about social & environmental consequences	Social comparison	Behavioural practice/rehearsal
0	0	Goal setting (behaviour)	Feedback on behaviour	Instruction on how to perform the behaviour	Information about social & environmental consequences	Social comparison	Behavioural practice/rehearsal
1	1	Goal setting (behaviour)	Discrepancy between current behaviour and goal	Feedback on behaviour	social support	Instruction on how to perform the behaviour	Credible source
1	1	Problem solving	Action planning	Discrepancy between current behaviour and goal	Feedback on behaviour	self monitoring of behaviour	Prompts/cues
1	1	Goal setting (behaviour)	self monitoring of behaviour	social support	Graded tasks	Social reward	
1	1	Goal setting (behaviour)	self monitoring of behaviour	social support	Social reward	Adding object to the environment	
0	0	Goal setting (behaviour)	Action planning	social support	Instruction on how to perform the behaviour	Verbal persuasion about capability	
1	1	Goal setting (behaviour)	Instruction on how to perform the behaviour	Prompts/cues	Social reward	Adding object to the environment	
1	1	Goal setting (behaviour)	self monitoring of behaviour	Instruction on how to perform the behaviour	Social reward	Adding object to the environment	
1	1	Goal setting (behaviour)	social support	Instruction on how to perform the behaviour	Prompts/cues	Social reward	
1	1	Goal setting (behaviour)	social support	Instruction on how to perform the behaviour	Graded tasks	Social reward	
2	2	Problem solving	Action planning	Review of behaviour goals	Demonstration of the behaviour	Behavioural practice/rehearsal	Graded tasks
1	1	Problem solving	Action planning	Feedback on behaviour	Instruction on how to perform the behaviour	Information about health consequences	Graded tasks
1	1	Problem solving	Action planning	Discrepancy between current behaviour and goal	Feedback on behaviour	Information about health consequences	Prompts/cues
1	1	Problem solving	Action planning	Discrepancy between current behaviour and goal	Feedback on behaviour	Information about health consequences	Credible source
1	1	Problem solving	Action planning	Discrepancy between current behaviour and goal	Feedback on behaviour	Prompts/cues	Credible source
1	1	Problem solving	Action planning	Discrepancy between current behaviour and goal	self monitoring of behaviour	Instruction on how to perform the behaviour	Information about health consequences
1	1	Problem solving	Action planning	Discrepancy between current behaviour and goal	self monitoring of behaviour	Instruction on how to perform the behaviour	Prompts/cues
1	1	Problem solving	Action planning	Discrepancy between current behaviour and goal	Feedback on behaviour	self monitoring of behaviour	Information about health consequences
1	1	Goal setting (behaviour)	Problem solving	Instruction on how to perform the behaviour	Social reward	Adding object to the environment	
1	1	Goal setting (behaviour)	Problem solving	social support	Graded tasks	Social reward	
1	1	Goal setting (behaviour)	self monitoring of behaviour	social support	Instruction on how to perform the behaviour	Social reward	
0	0	Goal setting (behaviour)	self monitoring of behaviour	social support	Instruction on how to perform the behaviour	Verbal persuasion about capability	
1	1	Problem solving	Action planning	Feedback on behaviour	self monitoring of behaviour	Information about health consequences	Graded tasks
2	2	Problem solving	Action planning	Feedback on behaviour	Instruction on how to perform the behaviour	Demonstration of the behaviour	Behavioural practice/rehearsal
0	0	Problem solving	self monitoring of behaviour	social support	Social comparison	Behavioural practice/rehearsal	
1	1	Problem solving	social support	Instruction on how to perform the behaviour	Prompts/cues	Graded tasks	Adding object to the environment
1	1	Problem solving	Action planning	Discrepancy between current behaviour and goal	Information about health consequences	Prompts/cues	
1	1	Problem solving	Action planning	Discrepancy between current behaviour and goal	Prompts/cues	Credible source	
1	1	Problem solving	Action planning	Discrepancy between current behaviour and goal	self monitoring of behaviour	Prompts/cues	
1	1	Goal setting (behaviour)	Problem solving	social support	Social reward	Adding object to the environment	
1	1	Goal setting (behaviour)	Problem solving	Instruction on how to perform the behaviour	Prompts/cues	Social reward	
1	1	Goal setting (behaviour)	Problem solving	Instruction on how to perform the behaviour	Prompts/cues	Adding object to the environment	
1	1	Goal setting (behaviour)	Problem solving	Instruction on how to perform the behaviour	Graded tasks	Social reward	
2	2	Problem solving	Action planning	Feedback on behaviour	Behavioural practice/rehearsal	Graded tasks	
1	1	Problem solving	Action planning	Discrepancy between current behaviour and goal	Information about health consequences	Prompts/cues	Credible source
2	2	Problem solving	Action planning	self monitoring of behaviour	Instruction on how to perform the behaviour	Behavioural practice/rehearsal	Graded tasks
2	2	Problem solving	Action planning	Feedback on behaviour	self monitoring of behaviour	Behavioural practice/rehearsal	Graded tasks
2	2	Problem solving	Action planning	Feedback on behaviour	self monitoring of behaviour	Behavioural practice/rehearsal	Credible source
1	1	Problem solving	Action planning	Discrepancy between current behaviour and goal	Feedback on behaviour	Information about health consequences	
1	1	Problem solving	Action planning	Discrepancy between current behaviour and goal	Feedback on behaviour	Prompts/cues	
1	1	Problem solving	Action planning	Discrepancy between current behaviour and goal	self monitoring of behaviour	Information about health consequences	
2	2	Action planning	Feedback on behaviour	self monitoring of behaviour	social support	Demonstration of the behaviour	Credible source
1	1	Problem solving	Action planning	Discrepancy between current behaviour and goal	self monitoring of behaviour	Information about health consequences	Credible source
1	1	Problem solving	Action planning	Discrepancy between current behaviour and goal	self monitoring of behaviour	Prompts/cues	Credible source
1	1	Problem solving	Action planning	Discrepancy between current behaviour and goal	Instruction on how to perform the behaviour	Information about health consequences	Prompts/cues
1	1	Problem solving	Action planning	Discrepancy between current behaviour and goal	Instruction on how to perform the behaviour	Prompts/cues	Credible source
1	1	Action planning	Feedback on behaviour	social support	Information about health consequences	Graded tasks	
1	1	Problem solving	Action planning	self monitoring of behaviour	Information about health consequences	Prompts/cues	Credible source
1	1	Problem solving	Action planning	self monitoring of behaviour	Instruction on how to perform the behaviour	Information about health consequences	Graded tasks
2	2	Problem solving	Action planning	Feedback on behaviour	self monitoring of behaviour	Demonstration of the behaviour	Prompts/cues
2	2	Problem solving	Action planning	Feedback on behaviour	Behavioural practice/rehearsal	Credible source	
2	2	Problem solving	Action planning	self monitoring of behaviour	Social support (emotional)	Demonstration of the behaviour	
1	1	Problem solving	Action planning	Discrepancy between current behaviour and goal	Instruction on how to perform the behaviour	Prompts/cues	
0	0	Problem solving	self monitoring of behaviour	Information about health consequences	Social comparison	Behavioural practice/rehearsal	
2	2	Problem solving	Action planning	Feedback on behaviour	Instruction on how to perform the behaviour	Behavioural practice/rehearsal	Graded tasks
2	2	Problem solving	Action planning	Feedback on behaviour	Instruction on how to perform the behaviour	Behavioural practice/rehearsal	Credible source
1	1	Problem solving	Action planning	Feedback on behaviour	Information about health consequences	Prompts/cues	Credible source
2	2	Problem solving	Action planning	Feedback on behaviour	Demonstration of the behaviour	Behavioural practice/rehearsal	Graded tasks
1	1	Goal setting (behaviour)	social support	Prompts/cues	Graded tasks	Adding object to the environment	
1	1	Goal setting (behaviour)	social support	Prompts/cues	Social reward	Adding object to the environment	
1	1	Goal setting (behaviour)	social support	Graded tasks	Social reward	Adding object to the environment	
1	1	Goal setting (behaviour)	Instruction on how to perform the behaviour	Prompts/cues	Graded tasks	Adding object to the environment	
2	2	Problem solving	Action planning	Review of behaviour goals	Demonstration of the behaviour	Graded tasks	
1	1	Problem solving	Action planning	Review of behaviour goals	Prompts/cues	Graded tasks	
2	2	Problem solving	Action planning	Review of behaviour goals	Behavioural practice/rehearsal	Graded tasks	
2	2	Problem solving	Action planning	Review of behaviour goals	Graded tasks	Credible source	

2	2	Action planning	Review of behaviour goals	self monitoring of behaviour	Instruction on how to perform the behaviour	Demonstration of the behaviour	Behavioural practice/rehearsal		
2	2	Action planning	Review of behaviour goals	Feedback on behaviour	Instruction on how to perform the behaviour	Demonstration of the behaviour	Behavioural practice/rehearsal		
2	2	Action planning	Review of behaviour goals	Feedback on behaviour	Instruction on how to perform the behaviour	Demonstration of the behaviour	Graded tasks		
2	2	Goal setting (behaviour)	Action planning	Feedback on behaviour	self monitoring of behaviour	social support	Instruction on how to perform the behaviour	Demonstration of the behaviour	Credible source
1	1	Action planning	Discrepancy between current behaviour and goal	Feedback on behaviour	self monitoring of behaviour	social support	Instruction on how to perform the behaviour	Credible source	
0	0	Action planning	Feedback on behaviour	self monitoring of behaviour	social support	Instruction on how to perform the behaviour	Information abour social & environmental consequences	Social comparison	
0	0	Goal setting (behaviour)	Action planning	Feedback on behaviour	self monitoring of behaviour	social support	Information abour social & environmental consequences	Social comparison	Behavioural practice/rehearsal
0	0	Goal setting (behaviour)	Action planning	Feedback on behaviour	self monitoring of behaviour	Instruction on how to perform the behaviour	Information abour social & environmental consequences	Social comparison	Behavioural practice/rehearsal
2	2	Action planning	Review of behaviour goals	Feedback on behaviour	self monitoring of behaviour	Instruction on how to perform the behaviour	Demonstration of the behaviour		
1	1	Action planning	Review of behaviour goals	Feedback on behaviour	self monitoring of behaviour	Instruction on how to perform the behaviour	Prompts/cues		
1	1	Goal setting (behaviour)	Action planning	social support	Information about health consequences	Graded tasks			
0	0	Goal setting (behaviour)	Feedback on behaviour	self monitoring of behaviour	social support	Instruction on how to perform the behaviour	Information abour social & environmental consequences	Social comparison	
0	0	Goal setting (behaviour)	Feedback on behaviour	self monitoring of behaviour	social support	Instruction on how to perform the behaviour	Information abour social & environmental consequences	Behavioural practice/rehearsal	
0	0	Goal setting (behaviour)	Feedback on behaviour	self monitoring of behaviour	social support	Information abour social & environmental consequences	Social comparison	Behavioural practice/rehearsal	
0	0	Action planning	self monitoring of behaviour	social support	Instruction on how to perform the behaviour	Information abour social & environmental consequences	Social comparison	Behavioural practice/rehearsal	
0	0	Feedback on behaviour	self monitoring of behaviour	social support	Instruction on how to perform the behaviour	Information abour social & environmental consequences	Social comparison	Behavioural practice/rehearsal	
1	1	Goal setting (behaviour)	Action planning	Discrepancy between current behaviour and goal	Feedback on behaviour	self monitoring of behaviour	social support	Instruction on how to perform the behaviour	Credible source
0	0	Goal setting (behaviour)	Action planning	Feedback on behaviour	self monitoring of behaviour	social support	Instruction on how to perform the behaviour	Information abour social & environmental consequences	Social comparison
0	0	Goal setting (behaviour)	Action planning	Feedback on behaviour	self monitoring of behaviour	social support	Instruction on how to perform the behaviour	Information abour social & environmental consequences	Behavioural practice/rehearsal
0	0	Problem solving	Information about emotinal consequences	Social comparison					
1	1	Goal setting (behaviour)	Discrepancy between current behaviour and goal	Instruction on how to perform the behaviour	Information about health consequences	Credible source			
1	1	Goal setting (behaviour)	Discrepancy between current behaviour and goal	Instruction on how to perform the behaviour	Prompts/cues	Credible source			
0	0	Action planning	Feedback on behaviour	self monitoring of behaviour	social support	Instruction on how to perform the behaviour	Information abour social & environmental consequences	Behavioural practice/rehearsal	
2	2	Action planning	Feedback on behaviour	self monitoring of behaviour	social support	Instruction on how to perform the behaviour	Demonstration of the behaviour	Credible source	
0	0	Action planning	Feedback on behaviour	self monitoring of behaviour	social support	Information abour social & environmental consequences	Social comparison	Behavioural practice/rehearsal	
0	0	Action planning	Feedback on behaviour	self monitoring of behaviour	Instruction on how to perform the behaviour	Information abour social & environmental consequences	Social comparison	Behavioural practice/rehearsal	
1	1	Goal setting (behaviour)	Discrepancy between current behaviour and goal	Feedback on behaviour	self monitoring of behaviour	social support	Instruction on how to perform the behaviour	Credible source	
1	1	Action planning	Discrepancy between current behaviour and goal	Feedback on behaviour	self monitoring of behaviour	Information about health consequences	Prompts/cues		
1	1	Action planning	Discrepancy between current behaviour and goal	Feedback on behaviour	self monitoring of behaviour	Information about health consequences	Credible source		
1	1	Action planning	Discrepancy between current behaviour and goal	Feedback on behaviour	self monitoring of behaviour	Prompts/cues	Credible source		
1	1	Action planning	Discrepancy between current behaviour and goal	Feedback on behaviour	Instruction on how to perform the behaviour	Information about health consequences	Prompts/cues		
1	1	Action planning	Discrepancy between current behaviour and goal	Feedback on behaviour	Instruction on how to perform the behaviour	Prompts/cues	Credible source		
1	1	Action planning	Discrepancy between current behaviour and goal	Feedback on behaviour	Information about health consequences	Prompts/cues	Credible source		
1	1	Action planning	Discrepancy between current behaviour and goal	self monitoring of behaviour	Instruction on how to perform the behaviour	Information about health consequences	Prompts/cues		
1	1	Review of behaviour goals	social support	Information about health consequences	Graded tasks				
1	1	Goal setting (behaviour)	self monitoring of behaviour	Behavioural practice/rehearsal	Graded tasks	Credible source			
1	1	Goal setting (behaviour)	Feedback on behaviour	Information about health consequences	Prompts/cues	Graded tasks			
1	1	Goal setting (behaviour)	Discrepancy between current behaviour and goal	Information about health consequences	Prompts/cues	Credible source			
1	1	Goal setting (behaviour)	Discrepancy between current behaviour and goal	self monitoring of behaviour	Information about health consequences	Credible source			
0	0	self monitoring of behaviour	Information about health consequences	Social comparison	Behavioural practice/rehearsal				
2	2	Action planning	Review of behaviour goals	Feedback on behaviour	Instruction on how to perform the behaviour	Graded tasks	Credible source		
1	1	Action planning	Discrepancy between current behaviour and goal	self monitoring of behaviour	Instruction on how to perform the behaviour	Information about health consequences	Credible source		
1	1	Action planning	Discrepancy between current behaviour and goal	self monitoring of behaviour	Instruction on how to perform the behaviour	Prompts/cues	Credible source		
0	0	Goal setting (behaviour)	Problem solving	Information abour social & environmental consequences	Information about emotinal consequences				
0	0	Goal setting (behaviour)	Problem solving	Information about emotinal consequences	Social comparison				
0	0	Goal setting (behaviour)	Problem solving	Information about emotinal consequences	Behavioural practice/rehearsal				
0	0	Goal setting (behaviour)	Action planning	social support	Information about emotinal consequences				
0	0	Goal setting (behaviour)	Action planning	Information abour social & environmental consequences	Information about emotinal consequences				
0	0	Goal setting (behaviour)	Action planning	Information about emotinal consequences	Social comparison				
0	0	Goal setting (behaviour)	Action planning	Information about emotinal consequences	Behavioural practice/rehearsal				
0	0	Goal setting (behaviour)	social support	Instruction on how to perform the behaviour	Information about emotinal consequences				
2	2	Action planning	Review of behaviour goals	Feedback on behaviour	self monitoring of behaviour	Graded tasks	Credible source		
1	1	Action planning	Review of behaviour goals	Feedback on behaviour	Instruction on how to perform the behaviour	Information about health consequences	Prompts/cues		
1	1	Action planning	Review of behaviour goals	Feedback on behaviour	self monitoring of behaviour	Information about health consequences	Prompts/cues		
0	0	Information about health consequences	Information about emotinal consequences	Behavioural practice/rehearsal					
0	0	Information abour social & environmental consequences	Information about emotinal consequences	Social comparison					
0	0	Information abour social & environmental consequences	Information about emotinal consequences	Behavioural practice/rehearsal					
0	0	Information about emotinal consequences	Social comparison	Behavioural practice/rehearsal					
0	0	Goal setting (behaviour)	Problem solving	social support	Information about emotinal consequences				
2	2	Action planning	Review of behaviour goals	Feedback on behaviour	Demonstration of the behaviour	Behavioural practice/rehearsal	Graded tasks		

0	0	Goal setting (behaviour)	Problem solving	Information abour social & environmental consequences	Information about emotional consequences	Social comparison		
0	0	Goal setting (behaviour)	Problem solving	Information abour social & environmental consequences	Information about emotional consequences	Behavioural practice/rehearsal		
0	0	Goal setting (behaviour)	Problem solving	Information abour social & environmental consequences	Social comparison	Behavioural practice/rehearsal		
0	0	Goal setting (behaviour)	Problem solving	Information about emotional consequences	Social comparison	Behavioural practice/rehearsal		
0	0	Goal setting (behaviour)	Action planning	social support	Instruction on how to perform the behaviour	Information about emotional consequences		
0	0	Goal setting (behaviour)	Action planning	social support	Information about health consequences	Information about emotional consequences		
0	0	Goal setting (behaviour)	Problem solving	social support	Information about emotional consequences	Social comparison		
0	0	Goal setting (behaviour)	Problem solving	social support	Information about emotional consequences	Behavioural practice/rehearsal		
0	0	Goal setting (behaviour)	Problem solving	Instruction on how to perform the behaviour	Information abour social & environmental consequences	Information about emotional consequences		
0	0	Goal setting (behaviour)	Problem solving	Instruction on how to perform the behaviour	Information abour social & environmental consequences	Social comparison		
0	0	Goal setting (behaviour)	Problem solving	Instruction on how to perform the behaviour	Information about emotional consequences	Social comparison		
0	0	Goal setting (behaviour)	Problem solving	Instruction on how to perform the behaviour	Information about emotional consequences	Behavioural practice/rehearsal		
0	0	Goal setting (behaviour)	Problem solving	Information about health consequences	Information abour social & environmental consequences	Information about emotional consequences		
0	0	Goal setting (behaviour)	Problem solving	Information about health consequences	Information abour social & environmental consequences	Social comparison		
0	0	Goal setting (behaviour)	social support	Information abour social & environmental consequences	Information about emotional consequences	Social comparison		
0	0	Goal setting (behaviour)	Action planning	Information about health consequences	Information abour social & environmental consequences	Information about emotional consequences		
0	0	Goal setting (behaviour)	Action planning	Information about health consequences	Information abour social & environmental consequences	Social comparison		
0	0	Goal setting (behaviour)	Action planning	Information about health consequences	Information about emotional consequences	Social comparison		
0	0	Goal setting (behaviour)	Action planning	Information about health consequences	Information about emotional consequences	Behavioural practice/rehearsal		
0	0	Goal setting (behaviour)	Action planning	Information abour social & environmental consequences	Information about emotional consequences	Social comparison		
0	0	Goal setting (behaviour)	Problem solving	social support	Information abour social & environmental consequences	Information about emotional consequences		
0	0	Goal setting (behaviour)	Problem solving	social support	Information abour social & environmental consequences	Social comparison		
1	1	Goal setting (behaviour)	Problem solving	Action planning	Discrepancy between current behaviour and goal	self monitoring of behaviour	Instruction on how to perform the behaviour	Information about health consequences
1	1	Goal setting (behaviour)	Problem solving	Action planning	Discrepancy between current behaviour and goal	self monitoring of behaviour	Instruction on how to perform the behaviour	Prompts/cues
0	0	Goal setting (behaviour)	social support	Instruction on how to perform the behaviour	Information about health consequences	Information about emotional consequences		
1	1	Goal setting (behaviour)	Action planning	Review of behaviour goals	Feedback on behaviour	Prompts/cues		
2	2	Goal setting (behaviour)	Action planning	Review of behaviour goals	Feedback on behaviour	Behavioural practice/rehearsal		
1	1	Goal setting (behaviour)	Action planning	Review of behaviour goals	Feedback on behaviour	Credible source		
0	0	Goal setting (behaviour)	social support	Information about health consequences	Information about emotional consequences	Social comparison		
0	0	Goal setting (behaviour)	social support	Information about health consequences	Information about emotional consequences	Behavioural practice/rehearsal		
0	0	Goal setting (behaviour)	Feedback on behaviour	social support	Demonstration of the behaviour	Behavioural practice/rehearsal	Credible source	
2	2	Goal setting (behaviour)	Problem solving	Demonstration of the behaviour	Prompts/cues	Graded tasks		
[illegible]	[illegible]	[illegible]	[illegible]	[illegible]	[illegible]	[illegible]	[illegible]	[illegible]

1	1	Goal setting (behaviour)	Feedback on behaviour	social support	Instruction on how to perform the behaviour	Demonstration of the behaviour	Prompts/cues	
2	2	Goal setting (behaviour)	Action planning	Feedback on behaviour	Instruction on how to perform the behaviour	Demonstration of the behaviour	Prompts/cues	Graded tasks
3	3	Goal setting (behaviour)	Action planning	Feedback on behaviour	Instruction on how to perform the behaviour	Demonstration of the behaviour	Graded tasks	Credible source
2	2	Goal setting (behaviour)	Action planning	Feedback on behaviour	self monitoring of behaviour	Demonstration of the behaviour	Prompts/cues	Graded tasks
3	3	Goal setting (behaviour)	Action planning	Feedback on behaviour	self monitoring of behaviour	Demonstration of the behaviour	Graded tasks	Credible source
1	1	Goal setting (behaviour)	Action planning	Discrepancy between current behaviour and goal	Feedback on behaviour	Instruction on how to perform the behaviour	Prompts/cues	Credible source
1	1	Goal setting (behaviour)	Action planning	Discrepancy between current behaviour and goal	Feedback on behaviour	Information about health consequences	Prompts/cues	Credible source
1	1	Goal setting (behaviour)	Action planning	Discrepancy between current behaviour and goal	self monitoring of behaviour	Instruction on how to perform the behaviour	Information about health consequences	Prompts/cues
1	1	Goal setting (behaviour)	Action planning	Discrepancy between current behaviour and goal	self monitoring of behaviour	Instruction on how to perform the behaviour	Information about health consequences	Credible source
0	0	social support	Instruction on how to perform the behaviour	Information about health consequences	Information abour social & environmental consequences	Information about emotinal consequences		
0	0	social support	Instruction on how to perform the behaviour	Information about health consequences	Information abour social & environmental consequences	Social comparison		
0	0	social support	Instruction on how to perform the behaviour	Information about health consequences	Information about emotinal consequences	Behavioural practice/rehearsal		
0	0	Action planning	social support	Information abour social & environmental consequences	Information about emotinal consequences	Behavioural practice/rehearsal		
0	0	Action planning	social support	Information about emotinal consequences	Social comparison	Behavioural practice/rehearsal		
1	1	Instruction on how to perform the behaviour	Behavioural practice/rehearsal	Generalisation of target behaviour	Credible source			
3	3	Goal setting (behaviour)	Action planning	self monitoring of behaviour	Instruction on how to perform the behaviour	Demonstration of the behaviour	Graded tasks	Credible source
1	1	Goal setting (behaviour)	Action planning	self monitoring of behaviour	Instruction on how to perform the behaviour	Information about health consequences	Prompts/cues	Credible source
0	0	Action planning	Instruction on how to perform the behaviour	Information abour social & environmental consequences	Information about emotinal consequences	Social comparison		
0	0	Action planning	Instruction on how to perform the behaviour	Information abour social & environmental consequences	Information about emotinal consequences	Behavioural practice/rehearsal		
0	0	Action planning	Instruction on how to perform the behaviour	Information about emotinal consequences	Social comparison	Behavioural practice/rehearsal		
0	0	Problem solving	Information about health consequences	Information about emotinal consequences	Social comparison	Behavioural practice/rehearsal		
0	0	Problem solving	Information abour social & environmental consequences	Information about emotinal consequences	Social comparison	Behavioural practice/rehearsal		
0	0	Action planning	Information about health consequences	Information abour social & environmental consequences	Social comparison	Behavioural practice/rehearsal		
0	0	Action planning	Information about health consequences	Information about emotinal consequences	Social comparison	Behavioural practice/rehearsal		
0	0	Action planning	Information abour social & environmental consequences	Information about emotinal consequences	Social comparison	Behavioural practice/rehearsal		
0	0	Goal setting (behaviour)	Problem solving	Action planning	social support	Information abour social & environmental consequences	Information about emotinal consequences	
0	0	Goal setting (behaviour)	Problem solving	Action planning	social support	Information abour social & environmental consequences	Social comparison	
0	0	Goal setting (behaviour)	Problem solving	Action planning	social support	Information about emotinal consequences	Social comparison	
0	0	Goal setting (behaviour)	Problem solving	Action planning	social support	Information about emotinal consequences	Behavioural practice/rehearsal	
0	0	Goal setting (behaviour)	Problem solving	Action planning	Instruction on how to perform the behaviour	Information abour social & environmental consequences	Information about emotinal consequences	
0	0	Action planning	Instruction on how to perform the behaviour	Information about health consequences	Information abour social & environmental consequences	Information about emotinal consequences		
0	0	Action planning	Instruction on how to perform the behaviour	Information about health consequences	Information abour social & environmental consequences	Social comparison		
0	0	Action planning	Instruction on how to perform the behaviour	Information about health consequences	Information about emotinal consequences	Behavioural practice/rehearsal		
1	1	Problem solving	Feedback on behaviour	Prompts/cues	Graded tasks	Social reward		
1	1	Problem solving	Feedback on behaviour	Prompts/cues	Graded tasks	Adding object to the environment		
1	1	Problem solving	Feedback on behaviour	Prompts/cues	Social reward	Adding object to the environment		
1	1	Problem solving	Feedback on behaviour	Graded tasks	Social reward	Adding object to the environment		
0	0	Problem solving	Feedback on behaviour	social support	Information about health consequences	Adding object to the environment		
0	0	Problem solving	Feedback on behaviour	Instruction on how to perform the behaviour	Information about health consequences	Adding object to the environment		
3	3	Feedback on behaviour	self monitoring of behaviour	Social support (emotional)	Demonstration of the behaviour			
1	1	Goal setting (behaviour)	Discrepancy between current behaviour and goal	Feedback on behaviour	self monitoring of behaviour	Instruction on how to perform the behaviour	Information about health consequences	Prompts/cues
2	2	Problem solving	Action planning	Feedback on behaviour	Graded tasks	Adding object to the environment		
0	0	Goal setting (behaviour)	Feedback on behaviour	Instruction on how to perform the behaviour	Information about health consequences	Adding object to the environment		
1	1	Goal setting (behaviour)	Feedback on behaviour	Prompts/cues	Graded tasks	Adding object to the environment		
1	1	Goal setting (behaviour)	Feedback on behaviour	Prompts/cues	Social reward	Adding object to the environment		
1	1	Goal setting (behaviour)	Feedback on behaviour	Graded tasks	Social reward	Adding object to the environment		
3	2	Feedback on behaviour	self monitoring of behaviour	Feedback on outcomes of behaviour	Instruction on how to perform the behaviour			
4	2	Goal setting (outcome)	Feedback on outcomes of behaviour	Instruction on how to perform the behaviour	Credible source			
1	1	Problem solving	Feedback on behaviour	self monitoring of behaviour	Prompts/cues	Adding object to the environment		
1	1	Feedback on behaviour	Graded tasks	Social reward	Adding object to the environment			
2	2	self monitoring of behaviour	Self monitoring of outcome(s) of behaviour	Instruction on how to perform the behaviour	Demonstration of the behaviour			
2	2	self monitoring of behaviour	Self monitoring of outcome(s) of behaviour	Instruction on how to perform the behaviour	Graded tasks			
1	1	Goal setting (behaviour)	Feedback on behaviour	self monitoring of behaviour	Social reward	Adding object to the environment		
2	2	Goal setting (behaviour)	Action planning	Feedback on behaviour	Graded tasks	Adding object to the environment		
1	1	Goal setting (behaviour)	Problem solving	Feedback on behaviour	Graded tasks	Social reward		
1	1	Goal setting (behaviour)	Problem solving	Feedback on behaviour	Social reward	Adding object to the environment		
1	1	Goal setting (behaviour)	Problem solving	Feedback on behaviour	Prompts/cues	Adding object to the environment		
1	1	Goal setting (behaviour)	Action planning	Discrepancy between current behaviour and goal	self monitoring of behaviour	Instruction on how to perform the behaviour	Prompts/cues	Credible source
2	2	self monitoring of behaviour	Self monitoring of outcome(s) of behaviour	Demonstration of the behaviour	Graded tasks			
1	1	Feedback on behaviour	Instruction on how to perform the behaviour	Social comparison	Credible source			
1	1	Feedback on behaviour	social support	Instruction on how to perform the behaviour	Demonstration of the behaviour	Prompts/cues		
1	1	self monitoring of behaviour	social support	Instruction on how to perform the behaviour	Demonstration of the behaviour	Prompts/cues		
0	0	Feedback on behaviour	Instruction on how to perform the behaviour	Information about health consequences	Adding object to the environment			
1	1	Feedback on behaviour	Prompts/cues	Graded tasks	Adding object to the environment			
1	1	Feedback on behaviour	Prompts/cues	Social reward	Adding object to the environment			
2	2	Action planning	Feedback on behaviour	Instruction on how to perform the behaviour	Graded tasks	Adding object to the environment		
1	1	Action planning	Feedback on behaviour	social support	Prompts/cues	Graded tasks		
1	1	Goal setting (behaviour)	Feedback on behaviour	self monitoring of behaviour	Instruction on how to perform the behaviour	Behavioural practice/rehearsal	Graded tasks	Credible source
1	1	Goal setting (behaviour)	Discrepancy between current behaviour and goal	Feedback on behaviour	self monitoring of behaviour	Instruction on how to perform the behaviour	Prompts/cues	Credible source
1	1	Goal setting (behaviour)	Discrepancy between current behaviour and goal	Feedback on behaviour	self monitoring of behaviour	Information about health consequences	Prompts/cues	Credible source
1	1	Goal setting (behaviour)	Discrepancy between current behaviour and goal	Feedback on behaviour	Instruction on how to perform the behaviour	Information about health consequences	Prompts/cues	Credible source
1	1	Goal setting (behaviour)	Discrepancy between current behaviour and goal	self monitoring of behaviour	Instruction on how to perform the behaviour	Information about health consequences	Prompts/cues	Credible source
4	4	Problem solving	Goal setting (outcome)	Action planning	Feedback on behaviour	self monitoring of behaviour	Demonstration of the behaviour	Credible source
0	0	social support	Instruction on how to perform the behaviour	Information about emotinal consequences	Social comparison	Behavioural practice/rehearsal		
0	0	social support	Information about health consequences	Information abour social & environmental consequences	Information about emotinal consequences	Social comparison		
0	0	social support	Information about health consequences	Information abour social & environmental consequences	Information about emotinal consequences	Behavioural practice/rehearsal		
0	0	Goal setting (behaviour)	Problem solving	Action planning	Instruction on how to perform the behaviour	Information abour social & environmental consequences	Social comparison	
0	0	Goal setting (behaviour)	Problem solving	Action planning	Instruction on how to perform the behaviour	Information about emotinal consequences	Social comparison	
1	1	Goal setting (behaviour)	Review of behaviour goals	Feedback on behaviour	self monitoring of behaviour	Information about health consequences	Prompts/cues	Graded tasks
2	2	Action planning	Feedback on behaviour	self monitoring of behaviour	Graded tasks	Adding object to the environment		
2	2	Action planning	Feedback on behaviour	social support	Graded tasks	Adding object to the environment		
1	1	self monitoring of behaviour	Information about health consequences	Demonstration of the behaviour	Credible source			
1	1	self monitoring of behaviour	Information about health consequences	Prompts/cues	Graded tasks			
2	2	self monitoring of behaviour	Instruction on how to perform the behaviour	Information about health consequences	Reduce negative emotions			
0	0	Information about health consequences	Information abour social & environmental consequences	Information about emotinal consequences	Social comparison	Behavioural practice/rehearsal		
0	0	Goal setting (behaviour)	Problem solving	Action planning	social support	Instruction on how to perform the behaviour	Information about emotinal consequences	
0	0	Goal setting (behaviour)	Problem solving	Action planning	social support	Information about health consequences	Information about emotinal consequences	
0	0	social support	Instruction on how to perform the behaviour	Information abour social & environmental consequences	Information about emotinal consequences	Social comparison		
0	0	social support	Instruction on how to perform the behaviour	Information abour social & environmental consequences	Information about emotinal consequences	Behavioural practice/rehearsal		
1	1	Goal setting (behaviour)	Discrepancy between current behaviour and goal	Feedback on behaviour	self monitoring of behaviour	Instruction on how to perform the behaviour	Information about health consequences	Credible source
1	1	Problem solving	Instruction on how to perform the behaviour	Social reward				
0	0	Problem solving	Review of behaviour goals	Feedback on behaviour	self monitoring of behaviour	Adding object to the environment		
1	1	social support	Social reward	Adding object to the environment				
1	1	Instruction on how to perform the behaviour	Social reward	Adding object to the environment				
0	0	social support	Information about health consequences	Information abour social & environmental consequences	Social comparison	Behavioural practice/rehearsal		
0	0	social support	Information about health consequences	Information about emotinal consequences	Social comparison	Behavioural practice/rehearsal		
0	0	social support	Information abour social & environmental consequences	Information about emotinal consequences	Social comparison	Behavioural practice/rehearsal		
4	4	Goal setting (behaviour)	Goal setting (outcome)	self monitoring of behaviour	Demonstration of the behaviour	Graded tasks	Credible source	
4	4	Goal setting (behaviour)	Goal setting (outcome)	self monitoring of behaviour	Instruction on how to perform the behaviour	Demonstration of the behaviour	Graded tasks	
4	4	Goal setting (behaviour)	Goal setting (outcome)	Feedback on behaviour	Instruction on how to perform the behaviour	Demonstration of the behaviour	Graded tasks	
4	4	Goal setting (behaviour)	Goal setting (outcome)	Feedback on behaviour	Instruction on how to perform the behaviour	Demonstration of the behaviour	Credible source	
1	1	social support	Instruction on how to perform the behaviour	Social reward				
0	0	social support	Instruction on how to perform the behaviour	Verbal persuasion about capability				
1	1	Goal setting (behaviour)	social support	Instruction on how to perform the behaviour	Demonstration of the behaviour	Prompts/cues		
4	4	Problem solving	Goal setting (outcome)	Action planning	Feedback on behaviour	self monitoring of behaviour	Instruction on how to perform the behaviour	Graded tasks
0	0	Goal setting (behaviour)	Problem solving	Action planning	Information about health consequences	Information abour social & environmental consequences	Information about emotinal consequences	
0	0	Goal setting (behaviour)	Problem solving	Action planning	Information about health consequences	Information abour social & environmental consequences	Social comparison	

4	3	0	0	0	0	0	0	1	0	0	0	0	0	0	0	0	0	0	0	0	0	4	0	0	0	0	0	0	Goal setting (behaviour)	Action planning	social support	Information about health consequences	Information about emotinal consequences	Social comparison			75	0	0	0	0	0	0	6
4	3	0	0	0	0	0	0	1	0	0	0	0	0	0	0	0	0	0	0	0	0	4	0	0	0	0	0	0	Goal setting (behaviour)	Action planning	social support	Information about health consequences	Information about emotinal consequences	Behavioural practice/rehearsal			75	0	0	0	0	0	0	6
4	3	0	0	0	0	0	0	1	0	0	0	0	0	0	0	0	0	0	0	0	0	4	0	0	0	0	0	0	Goal setting (behaviour)	Problem solving	Instruction on how to perform the behaviour	Information about health consequences	Information about emotinal consequences	Behavioural practice/rehearsal			75	0	0	0	0	0	0	6
4	3	0	0	0	0	0	0	1	0	0	0	0	0	0	0	0	0	0	0	0	0	4	0	0	0	0	0	0	Goal setting (behaviour)	Problem solving	Instruction on how to perform the behaviour	Information abour social & environmental consequences	Information about emotinal consequences	Social comparison			75	0	0	0	0	0	0	6
4	4	1	1	0	0	0	0	0	0	0	0	0	0	0	0	0	0	0	0	0	0	4	1	1	1	1	1	1	Goal setting (behaviour)	Problem solving	Action planning	Prompts/cues	Social reward				100	100	100	0	0	0	0	5
4	4	0	0	0	0	0	0	0	1	0	0	0	0	0	0	0	0	0	0	0	0	4	1	0	1	0	0	0	Goal setting (behaviour)	Problem solving	Action planning	Prompts/cues	Verbal persuasion about capability				100	0	0	0	0	0	0	5
4	3	2	1	0	0	0	0	1	0	0	0	0	0	0	0	0	0	0	0	0	0	4	2	1	2	1	1	1	self monitoring of behaviour	Demonstration of the behaviour	Prompts/cues	Social reward					75	100	100	0	0	0	0	4
4	3	0	0	0	0	0	0	1	2	0	0	0	0	0	0	0	0	0	0	0	0	4	2	0	2	0	0	0	Feedback on behaviour	self monitoring of behaviour	Prompts/cues	Verbal persuasion about capability					75	0	0	0	0	0	0	4
4	4	2	1	0	0	0	0	0	0	0	0	0	0	0	0	0	0	0	0	0	0	4	2	1	2	1	1	1	Feedback on behaviour	social support	Prompts/cues	Social reward					100	100	100	0	0	0	0	4
4	2	1	1	0	0	0	0	1	1	0	0	0	0	0	1	1	0	0	0	0	0	4	3	1	3	1	1	1	Action planning	Discrepancy between current behaviour and goal	Feedback on behaviour	self monitoring of behaviour	Instruction on how to perform the behaviour	Information about health consequences	Credible source		67	50	100	0	0	0	0	7
4	2	1	1	0	0	0	0	1	1	0	0	0	0	0	1	1	0	0	0	0	0	4	3	1	3	1	1	1	Action planning	Discrepancy between current behaviour and goal	Feedback on behaviour	self monitoring of behaviour	Instruction on how to perform the behaviour	Prompts/cues	Credible source		67	50	100	0	0	0	0	7
4	2	1	1	0	0	0	0	1	1	0	0	0	0	0	1	1	0	0	0	0	0	4	3	1	3	1	1	1	Action planning	Discrepancy between current behaviour and goal	Feedback on behaviour	self monitoring of behaviour	Information about health consequences	Prompts/cues	Credible source		67	50	100	0	0	0	0	7
4	3	0	0	0	0	0	0	1	2	0	0	0	0	0	0	0	0	0	0	0	0	4	2	0	2	0	0	0	Goal setting (behaviour)	Feedback on behaviour	social support	Prompts/cues	Verbal persuasion about capability				75	0	0	0	0	0	0	5
4	3	2	1	0	0	0	0	1	0	0	0	0	0	0	0	0	0	0	0	0	0	4	2	1	2	1	1	1	Goal setting (behaviour)	Feedback on behaviour	Demonstration of the behaviour	Prompts/cues	Social reward				75	100	100	0	0	0	0	5
4	4	0	0	0	0	0	0	0	1	0	0	0	0	0	0	0	0	0	0	0	0	4	1	0	1	0	0	0	Problem solving	Action planning	social support	Prompts/cues	Verbal persuasion about capability				100	0	0	0	0	0	0	5
4	3	2	1	0	0	0	0	1	0	0	0	0	0	0	0	0	0	0	0	0	0	4	2	1	2	1	1	1	Goal setting (behaviour)	self monitoring of behaviour	Demonstration of the behaviour	Prompts/cues	Social reward				75	100	100	0	0	0	0	5
4	2	0	2	0	0	0	0	1	0	0	0	0	0	0	1	2	0	0	0	0	0	4	2	2	2	2	2	2	Action planning	Review of behaviour goals	Feedback on behaviour	Instruction on how to perform the behaviour	Demonstration of the behaviour	Behavioural practice/rehearsal	Graded tasks		67	0	100	0	0	0	0	7
4	2	0	2	0	0	0	0	1	0	0	0	0	0	0	1	2	0	0	0	0	0	4	2	2	2	2	2	2	Action planning	Review of behaviour goals	Feedback on behaviour	self monitoring of behaviour	Demonstration of the behaviour	Behavioural practice/rehearsal	Graded tasks		67	0	100	0	0	0	0	7
4	4	1	1	0	0	0	0	0	0	0	0	0	0	0	0	0	0	0	0	0	0	4	1	1	1	1	1	1	Goal setting (behaviour)	Problem solving	Action planning	social support	Social reward				100	100	100	0	0	0	0	5
4	4	0	0	0	0	0	0	0	1	0	0	0	0	0	0	0	0	0	0	0	0	4	1	0	1	0	0	0	Goal setting (behaviour)	Problem solving	Action planning	social support	Verbal persuasion about capability				100	0	0	0	0	0	0	5
4	3	2	1	0	0	0	0	1	0	0	0	0	0	0	0	0	0	0	0	0	0	4	2	1	2	1	1	1	Goal setting (behaviour)	Feedback on behaviour	self monitoring of behaviour	Demonstration of the behaviour	Social reward				75	100	100	0	0	0	0	5
4	3	0	0	0	0	0	0	1	2	0	0	0	0	0	0	0	0	0	0	0	0	4	2	0	2	0	0	0	Goal setting (behaviour)	Feedback on behaviour	self monitoring of behaviour	Prompts/cues	Verbal persuasion about capability				75	0	0	0	0	0	0	5
4	4	0	0	0	0	0	0	0	1	0	0	0	0	0	0	0	0	0	0	0	0	4	1	0	1	0	0	0	Goal setting (behaviour)	Action planning	self monitoring of behaviour	Prompts/cues	Verbal persuasion about capability				100	0	0	0	0	0	0	5
4	3	1	1	0	0	0	0	1	0	0	0	0	0	0	0	0	0	0	0	0	0	4	1	1	1	1	1	1	Goal setting (behaviour)	Action planning	social support	Demonstration of the behaviour	Prompts/cues				75	100	100	0	0	0	0	5
4	4	1	1	0	0	0	0	0	0	0	0	0	0	0	0	0	0	0	0	0	0	4	1	1	1	1	1	1	Goal setting (behaviour)	Action planning	social support	Prompts/cues	Social reward				100	100	100	0	0	0	0	5
4	4	0	0	0	0	0	0	0	1	0	0	0	0	0	0	0	0	0	0	0	0	4	1	0	1	0	0	0	Goal setting (behaviour)	Action planning	social support	Prompts/cues	Verbal persuasion about capability				100	0	0	0	0	0	0	5
4	2	0	2	0	0	0	0	1	0	0	0	0	0	0	1	2	0	0	0	0	0	4	2	2	2	2	2	2	Action planning	Review of behaviour goals	Feedback on behaviour	self monitoring of behaviour	Instruction on how to perform the behaviour	Graded tasks	Credible source		67	0	100	0	0	0	0	7
4	4	2	1	0	0	0	0	0	0	0	0	0	0	0	0	0	0	0	0	0	0	4	2	1	2	1	1	1	Goal setting (behaviour)	Feedback on behaviour	social support	Prompts/cues	Social reward				100	100	100	0	0	0	0	5
4	3	1	0	0	0	0	0	1	0	0	0	0	0	0	0	0	0	0	0	0	0	4	1	0	1	0	0	0	Problem solving	Feedback on behaviour	self monitoring of behaviour	social support	Information about health consequences	Adding object to the environment			75	100	0	0	0	0	0	6
4	4	0	0	0	0	0	0	0	1	0	0	0	0	0	0	0	0	0	0	0	0	4	1	0	1	0	0	0	Goal setting (behaviour)	Action planning	Feedback on behaviour	social support	Verbal persuasion about capability				100	0	0	0	0	0	0	5
4	3	1	1	0	0	0	0	1	0	0	0	0	0	0	0	0	0	0	0	0	0	4	1	1	1	1	1	1	Goal setting (behaviour)	Action planning	Feedback on behaviour	Prompts/cues	Social reward				75	100	100	0	0	0	0	5
4	4	1	1	0	0	0	0	0	0	0	0	0	0	0	0	0	0	0	0	0	0	4	1	1	1	1	1	1	Goal setting (behaviour)	Action planning	self monitoring of behaviour	social support	Social reward				100	100	100	0	0	0	0	5

1	1	Goal setting (behaviour)	Action planning	Discrepancy between current behaviour and goal	Feedback on behaviour	self monitoring of behaviour	Instruction on how to perform the behaviour	Information about health consequences	Credible source
1	1	Goal setting (behaviour)	Feedback on behaviour	self monitoring of behaviour	Demonstration of the behaviour	Prompts/cues	Adding object to the environment		
2	2	Goal setting (behaviour)	Feedback on behaviour	self monitoring of behaviour	Demonstration of the behaviour	Credible source	Adding object to the environment		
2	2	Problem solving	Action planning	Review of behaviour goals	Feedback on behaviour	self monitoring of behaviour	Instruction on how to perform the behaviour	Demonstration of the behaviour	Behavioural practice/rehearsal
2	2	Problem solving	Action planning	Review of behaviour goals	Feedback on behaviour	self monitoring of behaviour	Instruction on how to perform the behaviour	Demonstration of the behaviour	Graded tasks
2	2	Problem solving	Action planning	Review of behaviour goals	Feedback on behaviour	self monitoring of behaviour	Instruction on how to perform the behaviour	Behavioural practice/rehearsal	Graded tasks
2	2	Problem solving	Action planning	Review of behaviour goals	Feedback on behaviour	self monitoring of behaviour	Instruction on how to perform the behaviour	Graded tasks	Credible source
0	0	Problem solving	social support	Information about social & environmental consequences	Information about emotinal consequences	Social comparison	Behavioural practice/rehearsal		
0	0	Action planning	social support	Information about social & environmental consequences	Information about emotinal consequences	Social comparison	Behavioural practice/rehearsal		
0	0	Action planning	Instruction on how to perform the behaviour	Information about health consequences	Information about social & environmental consequences	Information about emotinal consequences	Social comparison		
2	2	Feedback on behaviour	self monitoring of behaviour	social support	Credible source	Adding object to the environment			
1	1	Feedback on behaviour	self monitoring of behaviour	Demonstration of the behaviour	Prompts/cues	Adding object to the environment			
2	2	Feedback on behaviour	self monitoring of behaviour	Demonstration of the behaviour	Credible source	Adding object to the environment			
4	4	Problem solving	Goal setting (outcome)	Action planning	Feedback on behaviour	self monitoring of behaviour	Instruction on how to perform the behaviour	Demonstration of the behaviour	Credible source
4	4	Problem solving	Goal setting (outcome)	Action planning	Feedback on behaviour	self monitoring of behaviour	Instruction on how to perform the behaviour	Graded tasks	Credible source
4	4	Goal setting (behaviour)	Goal setting (outcome)	Instruction on how to perform the behaviour	Demonstration of the behaviour	Graded tasks	Credible source		
0	0	Action planning	social support	Instruction on how to perform the behaviour	Information about health consequences	Information about emotinal consequences	Behavioural practice/rehearsal		
0	0	Action planning	social support	Instruction on how to perform the behaviour	Information about social & environmental consequences	Information about emotinal consequences	Social comparison		
0	0	Action planning	social support	Instruction on how to perform the behaviour	Information about social & environmental consequences	Information about emotinal consequences	Behavioural practice/rehearsal		
0	0	Action planning	social support	Instruction on how to perform the behaviour	Information about emotinal consequences	Social comparison	Behavioural practice/rehearsal		
0	0	Action planning	social support	Information about health consequences	Information about social & environmental consequences	Information about emotinal consequences	Social comparison		
0	0	Action planning	social support	Information about health consequences	Information about social & environmental consequences	Information about emotinal consequences	Behavioural practice/rehearsal		
0	0	Problem solving	social support	Information about health consequences	Information about emotinal consequences	Social comparison	Behavioural practice/rehearsal		
2	2	Problem solving	Behavioural practice/rehearsal	Graded tasks	Credible source				
0	0	Problem solving	Instruction on how to perform the behaviour	Information about health consequences	Information about social & environmental consequences	Information about emotinal consequences	Social comparison		
0	0	Problem solving	Instruction on how to perform the behaviour	Information about health consequences	Information about social & environmental consequences	Information about emotinal consequences	Behavioural practice/rehearsal		
0	0	Problem solving	Instruction on how to perform the behaviour	Information about health consequences	Information about social & environmental consequences	Social comparison	Behavioural practice/rehearsal		
0	0	Problem solving	Instruction on how to perform the behaviour	Information about health consequences	Information about emotinal consequences	Social comparison	Behavioural practice/rehearsal		
0	0	Problem solving	Instruction on how to perform the behaviour	Information about social & environmental consequences	Information about emotinal consequences	Social comparison	Behavioural practice/rehearsal		
0	0	Problem solving	Information about health consequences	Information about social & environmental consequences	Information about emotinal consequences	Social comparison	Behavioural practice/rehearsal		
1	1	Goal setting (behaviour)	Feedback on behaviour	self monitoring of behaviour	Instruction on how to perform the behaviour	Social comparison	Credible source		
0	0	Feedback on behaviour	self monitoring of behaviour	social support	Information about health consequences	Credible source			
0	0	Goal setting (behaviour)	Feedback on behaviour	self monitoring of behaviour	social support	Information about health consequences	Credible source		
2	1	Problem solving	Information about health consequences	Demonstration of the behaviour	Credible source				
1	1	Problem solving	Information about health consequences	Prompts/cues	Graded tasks				
1	1	Problem solving	Information about health consequences	Prompts/cues	Credible source				
1	1	Problem solving	Information about health consequences	Behavioural practice/rehearsal	Credible source				
2	2	Problem solving	Demonstration of the behaviour	Prompts/cues	Graded tasks				
2	2	Problem solving	Instruction on how to perform the behaviour	Demonstration of the behaviour	Prompts/cues				
2	2	Goal setting (outcome)	Action planning	Instruction on how to perform the behaviour	Behavioural practice/rehearsal				
4	4	Goal setting (outcome)	Action planning	Instruction on how to perform the behaviour	Graded tasks				
1	1	Problem solving	Information about health consequences	Demonstration of the behaviour	Behavioural practice/rehearsal				
2	2	Goal setting (outcome)	Feedback on behaviour	self monitoring of behaviour	Behavioural practice/rehearsal				
2	2	Goal setting (outcome)	Feedback on behaviour	Instruction on how to perform the behaviour	Behavioural practice/rehearsal				
4	4	Goal setting (outcome)	Action planning	Demonstration of the behaviour	Graded tasks				
4	4	Goal setting (outcome)	Action planning	Feedback on behaviour	Demonstration of the behaviour				
2	2	Problem solving	Action planning	Feedback on behaviour	self monitoring of behaviour	social support	Graded tasks	Adding object to the environment	
1	1	Problem solving	Feedback on behaviour	self monitoring of behaviour	Prompts/cues	Graded tasks	Social reward	Adding object to the environment	
2	2	Problem solving	Discrepancy between current behaviour and goal	self monitoring of behaviour	Demonstration of the behaviour				
3	2	Problem solving	Discrepancy between current behaviour and goal	Instruction on how to perform the behaviour	Demonstration of the behaviour				
2	2	Problem solving	Review of behaviour goals	Graded tasks	Credible source				
0	0	Instruction on how to perform the behaviour	Information about health consequences	Information about social & environmental consequences	Information about emotinal consequences	Social comparison	Behavioural practice/rehearsal		
1	1	Problem solving	Discrepancy between current behaviour and goal	Prompts/cues	Credible source				
2	2	Problem solving	Discrepancy between current behaviour and goal	Instruction on how to perform the behaviour	Graded tasks				
4	4	Goal setting (outcome)	Action planning	Graded tasks	Credible source				
2	2	Problem solving	Action planning	Review of behaviour goals	self monitoring of behaviour	Instruction on how to perform the behaviour	Demonstration of the behaviour	Behavioural practice/rehearsal	Graded tasks
2	2	Problem solving	Action planning	Review of behaviour goals	Feedback on behaviour	self monitoring of behaviour	Demonstration of the behaviour	Behavioural practice/rehearsal	Graded tasks
2	2	Goal setting (behaviour)	Action planning	Feedback on behaviour	self monitoring of behaviour	Instruction on how to perform the behaviour	Graded tasks	Adding object to the environment	
2	2	Goal setting (behaviour)	Action planning	Feedback on behaviour	social support	Instruction on how to perform the behaviour	Graded tasks	Adding object to the environment	
0	0	Goal setting (behaviour)	Feedback on behaviour	self monitoring of behaviour	social support	Instruction on how to perform the behaviour	Information about health consequences	Adding object to the environment	
1	1	Goal setting (behaviour)	self monitoring of behaviour	Social comparison	Credible source				
1	3	Goal setting (behaviour)	self monitoring of behaviour	Social support (emotional)	Graded tasks				
1	1	Goal setting (behaviour)	Feedback on behaviour	Social comparison	Credible source				
4	4	Problem solving	Goal setting (outcome)	Action planning	Feedback on behaviour	self monitoring of behaviour	Demonstration of the behaviour	Graded tasks	Credible source
4	4	Problem solving	Goal setting (outcome)	Action planning	Feedback on behaviour	Instruction on how to perform the behaviour	Demonstration of the behaviour	Graded tasks	Credible source

2	2	Goal setting (outcome)	Action planning	Adding object to the environment					
2	2	Goal setting (outcome)	social support	Demonstration of the behaviour					
0	0	Goal setting (behaviour)	Problem solving	Action planning	self monitoring of behaviour	social support	Information about health consequences	Information abour social & environmental consequences	
0	0	Goal setting (behaviour)	Problem solving	Action planning	self monitoring of behaviour	Instruction on how to perform the behaviour	Information about health consequences	Information abour social & environmental consequences	
0	0	Goal setting (behaviour)	Problem solving	Action planning	self monitoring of behaviour	Instruction on how to perform the behaviour	Information about health consequences	Adding object to the environment	
0	0	Goal setting (behaviour)	Problem solving	Action planning	self monitoring of behaviour	Instruction on how to perform the behaviour	Information abour social & environmental consequences	Behavioural practice/rehearsal	
2	2	Goal setting (behaviour)	Problem solving	Action planning	self monitoring of behaviour	Instruction on how to perform the behaviour	Demonstration of the behaviour	Adding object to the environment	
0	0	Goal setting (behaviour)	Problem solving	Action planning	self monitoring of behaviour	Instruction on how to perform the behaviour	Behavioural practice/rehearsal	Adding object to the environment	
0	0	Goal setting (behaviour)	Problem solving	Action planning	self monitoring of behaviour	social support	Information about health consequences	Credible source	
0	0	Goal setting (behaviour)	Problem solving	Action planning	self monitoring of behaviour	social support	Information about health consequences	Adding object to the environment	
2	2	Instruction on how to perform the behaviour	Demonstration of the behaviour	Generalisation of target behaviour					
0	0	self monitoring of behaviour	Information abour social & environmental consequences	Adding object to the environment					
0	0	social support	Instruction on how to perform the behaviour	Generalisation of target behaviour					
3	3	Feedback on behaviour	Instruction on how to perform the behaviour	Avoidance/reducing exposure to the behaviour					
0	0	Goal setting (behaviour)	Feedback on behaviour	self monitoring of behaviour	social support	Information about health consequences	Information abour social & environmental consequences		
3	3	Goal setting (behaviour)	Goal setting (outcome)	Feedback on behaviour	self monitoring of behaviour	social support	Instruction on how to perform the behaviour		
2	2	Goal setting (behaviour)	Goal setting (outcome)	Feedback on behaviour	self monitoring of behaviour	Instruction on how to perform the behaviour	Adding object to the environment		
3	3	Goal setting (behaviour)	Problem solving	Goal setting (outcome)	self monitoring of behaviour	social support	Instruction on how to perform the behaviour	Credible source	
1	1	Problem solving	Review of behaviour goals	Feedback on behaviour	social support	Credible source			
1	1	Problem solving	Review of behaviour goals	self monitoring of behaviour	social support	Credible source			
1	1	Goal setting (behaviour)	Problem solving	Review of behaviour goals	Feedback on behaviour	social support	Credible source		
1	1	Goal setting (behaviour)	Problem solving	Review of behaviour goals	self monitoring of behaviour	social support	Credible source		
0	0	Goal setting (behaviour)	Problem solving	Feedback on behaviour	social support	Information about health consequences	Information abour social & environmental consequences		
0	0	Goal setting (behaviour)	Problem solving	Feedback on behaviour	self monitoring of behaviour	Instruction on how to perform the behaviour	Information abour social & environmental consequences		
0	0	Goal setting (behaviour)	Problem solving	Feedback on behaviour	self monitoring of behaviour	Information about health consequences	Information abour social & environmental consequences		
0	0	Goal setting (behaviour)	Problem solving	Feedback on behaviour	self monitoring of behaviour	social support	Information abour social & environmental consequences		
3	2	Problem solving	Action planning	Discrepancy between current behaviour and goal	Demonstration of the behaviour				
1	1	Problem solving	Action planning	Discrepancy between current behaviour and goal	Prompts/cues				
1	1	Review of behaviour goals	Feedback on behaviour	self monitoring of behaviour	social support	Credible source			
1	1	Review of behaviour goals	Feedback on behaviour	social support	Instruction on how to perform the behaviour	Credible source			
1	1	Review of behaviour goals	self monitoring of behaviour	social support	Instruction on how to perform the behaviour	Credible source			
2	2	Action planning	Feedback on behaviour	social support	Graded tasks	Credible source			
2	2	Action planning	self monitoring of behaviour	social support	Graded tasks	Credible source			
1	1	Problem solving	Discrepancy between current behaviour and goal	self monitoring of behaviour	social support	Instruction on how to perform the behaviour			
0	0	Goal setting (behaviour)	Problem solving	social support	Instruction on how to perform the behaviour	Information about health consequences	Information abour social & environmental consequences	Social comparison	
0	0	Goal setting (behaviour)	Problem solving	social support	Instruction on how to perform the behaviour	Information about health consequences	Information about emotinal consequences	Social comparison	
0	0	Goal setting (behaviour)	Problem solving	social support	Instruction on how to perform the behaviour	Information about health consequences	Information about emotinal consequences	Behavioural practice/rehearsal	
0	0	Goal setting (behaviour)	Problem solving	social support	Instruction on how to perform the behaviour	Information abour social & environmental consequences	Information about emotinal consequences	Social comparison	
0	0	Goal setting (behaviour)	Problem solving	social support	Instruction on how to perform the behaviour	Information abour social & environmental consequences	Information about emotinal consequences	Behavioural practice/rehearsal	
3	2	Problem solving	Goal setting (outcome)	Behavioural practice/rehearsal	Credible source				
1	1	Problem solving	Action planning	Review of behaviour goals	Discrepancy between current behaviour and goal				
2	2	Problem solving	Action planning	self monitoring of behaviour	Social support (emotional)				
1	1	Goal setting (behaviour)	Review of behaviour goals	self monitoring of behaviour	social support	Instruction on how to perform the behaviour	Credible source		
1	1	Goal setting (behaviour)	Behavioural practice/rehearsal	Generalisation of target behaviour	Credible source				
1	1	Goal setting (behaviour)	Behavioural practice/rehearsal	Graded tasks	Credible source				
1	1	Problem solving	Goal setting (outcome)	Feedback on behaviour	Information about health consequences				
1	1	Problem solving	Goal setting (outcome)	self monitoring of behaviour	Information about health consequences				
0	0	Information about health consequences	Information abour social & environmental consequences	Adding object to the environment					
0	0	Information about health consequences	Behavioural practice/rehearsal	Adding object to the environment					
0	0	self monitoring of behaviour	Information about health consequences	Pros and cons					
0	0	Goal setting (behaviour)	Problem solving	Instruction on how to perform the behaviour	Information about health consequences	Information about emotinal consequences	Social comparison	Behavioural practice/rehearsal	
0	0	Goal setting (behaviour)	Action planning	social support	Instruction on how to perform the behaviour	Information about health consequences	Information about emotinal consequences	Behavioural practice/rehearsal	
0	0	Goal setting (behaviour)	Action planning	social support	Instruction on how to perform the behaviour	Information abour social & environmental consequences	Information about emotinal consequences	Social comparison	
0	0	Goal setting (behaviour)	Action planning	social support	Instruction on how to perform the behaviour	Information abour social & environmental consequences	Information about emotinal consequences	Behavioural practice/rehearsal	
2	2	Problem solving	Action planning	Social support (emotional)	Demonstration of the behaviour				
2	2	Problem solving	Action planning	self monitoring of behaviour	social support	Graded tasks	Credible source		
1	1	Goal setting (behaviour)	Review of behaviour goals	Feedback on behaviour	self monitoring of behaviour	social support	Credible source		
1	1	Goal setting (behaviour)	Review of behaviour goals	Feedback on behaviour	social support	Instruction on how to perform the behaviour	Credible source		
0	0	social support	Information abour social & environmental consequences	Adding object to the environment					
0	0	social support	Behavioural practice/rehearsal	Generalisation of target behaviour					
0	0	Action planning	Behavioural practice/rehearsal	Adding object to the environment					
0	0	Goal setting (behaviour)	Action planning	social support	Information about health consequences	Information abour social & environmental consequences	Information about emotinal consequences	Behavioural practice/rehearsal	
0	0	Goal setting (behaviour)	Action planning	social support	Information about health consequences	Information abour social & environmental consequences	Social comparison	Behavioural practice/rehearsal	
0	0	Goal setting (behaviour)	Action planning	social support	Information about health consequences	Information about emotinal consequences	Social comparison	Behavioural practice/rehearsal	
0	0	Goal setting (behaviour)	Problem solving	Instruction on how to perform the behaviour	Information about health consequences	Information abour social & environmental consequences	Information about emotinal consequences	Behavioural practice/rehearsal	
0	0	Goal setting (behaviour)	Problem solving	Instruction on how to perform the behaviour	Information about health consequences	Information abour social & environmental consequences	Social comparison	Behavioural practice/rehearsal	
2	2	Goal setting (behaviour)	Problem solving	Goal setting (outcome)	self monitoring of behaviour	social support	Instruction on how to perform the behaviour	Adding object to the environment	
2	2	Goal setting (behaviour)	Problem solving	Goal setting (outcome)	Action planning	social support	Instruction on how to perform the behaviour	Adding object to the environment	
2	2	Goal setting (behaviour)	Problem solving	Goal setting (outcome)	Action planning	self monitoring of behaviour	social support	Instruction on how to perform the behaviour	
2	2	Goal setting (behaviour)	Problem solving	Goal setting (outcome)	Action planning	self monitoring of behaviour	social support	Adding object to the environment	
2	2	Goal setting (behaviour)	Problem solving	Goal setting (outcome)	Action planning	self monitoring of behaviour	Instruction on how to perform the behaviour	Adding object to the environment	
0	0	self monitoring of behaviour	social support	Instruction on how to perform the behaviour	Information about health consequences	Information abour social & environmental consequences	Behavioural practice/rehearsal		
0	0	self monitoring of behaviour	social support	Instruction on how to perform the behaviour	Information about health consequences	Information abour social & environmental consequences	Adding object to the environment		
0	0	Instruction on how to perform the behaviour	Information abour social & environmental consequences	Adding object to the environment					
0	0	Action planning	self monitoring of behaviour	Instruction on how to perform the behaviour	Information about health consequences	Information abour social & environmental consequences	Behavioural practice/rehearsal		
0	0	Action planning	self monitoring of behaviour	social support	Instruction on how to perform the behaviour	Demonstration of the behaviour	Behavioural practice/rehearsal		
0	0	Action planning	self monitoring of behaviour	social support	Instruction on how to perform the behaviour	Behavioural practice/rehearsal	Credible source		
0	0	Action planning	self monitoring of behaviour	social support	Instruction on how to perform the behaviour	Behavioural practice/rehearsal	Adding object to the environment		
2	2	Goal setting (behaviour)	Goal setting (outcome)	Action planning	social support				
2	2	Goal setting (behaviour)	Goal setting (outcome)	Action planning	Adding object to the environment				
2	2	Goal setting (behaviour)	Goal setting (outcome)	social support	Demonstration of the behaviour				
2	2	Action planning	social support	Instruction on how to perform the behaviour	Demonstration of the behaviour	Credible source	Adding object to the environment		
0	0	Goal setting (behaviour)	Problem solving	Feedback on behaviour	self monitoring of behaviour	social support	Information about health consequences	Information abour social & environmental consequences	
3	3	Goal setting (behaviour)	Problem solving	Goal setting (outcome)	Feedback on behaviour	self monitoring of behaviour	social support	Instruction on how to perform the behaviour	
0	0	Feedback on behaviour	self monitoring of behaviour	social support	Instruction on how to perform the behaviour	Information about health consequences	Information abour social & environmental consequences		
0	0	Problem solving	Feedback on behaviour	social support	Instruction on how to perform the behaviour	Information about health consequences	Information abour social & environmental consequences		
0	0	Problem solving	Feedback on behaviour	self monitoring of behaviour	Instruction on how to perform the behaviour	Information about health consequences	Information abour social & environmental consequences		
2	2	self monitoring of behaviour	social support	Instruction on how to perform the behaviour	Demonstration of the behaviour	Credible source	Adding object to the environment		
0	0	Goal setting (behaviour)	Problem solving	Information abour social & environmental consequences	Adding object to the environment				
2	2	Action planning	self monitoring of behaviour	social support	Demonstration of the behaviour	Credible source	Adding object to the environment		
0	0	Goal setting (behaviour)	Feedback on behaviour	social support	Instruction on how to perform the behaviour	Information about health consequences	Information abour social & environmental consequences		
0	0	Goal setting (behaviour)	Feedback on behaviour	self monitoring of behaviour	Instruction on how to perform the behaviour	Information about health consequences	Information abour social & environmental consequences		
0	0	Goal setting (behaviour)	Feedback on behaviour	self monitoring of behaviour	Instruction on how to perform the behaviour	Behavioural practice/rehearsal	Adding object to the environment		
3	3	self monitoring of behaviour	Social support (emotional)	Graded tasks					
2	2	self monitoring of behaviour	Self monitoring of outcome(s) of behaviour	Graded tasks					
0	0	Goal setting (behaviour)	Problem solving	Feedback on behaviour	social support	Instruction on how to perform the behaviour	Information about health consequences	Information abour social & environmental consequences	
0	0	Goal setting (behaviour)	Problem solving	Feedback on behaviour	self monitoring of behaviour	Instruction on how to perform the behaviour	Information about health consequences	Information abour social & environmental consequences	
0	0	Goal setting (behaviour)	Problem solving	Feedback on behaviour	self monitoring of behaviour	social support	Instruction on how to perform the behaviour	Information abour social & environmental consequences	
0	0	self monitoring of behaviour	social support	Pros and cons					
0	0	Action planning	Information about health consequences	Adding object to the environment					
3	3	self monitoring of behaviour	Self monitoring of outcome(s) of behaviour	Credible source					
4	2	self monitoring of behaviour	Feedback on outcomes of behaviour	Credible source					
2	2	self monitoring of behaviour	Self monitoring of outcome(s) of behaviour	Demonstration of the behaviour					
3	3	Feedback on behaviour	Social support (emotional)	Demonstration of the behaviour					
0	0	Problem solving	Feedback on behaviour	self monitoring of behaviour	social support	Information about health consequences	Information abour social & environmental consequences		
0	0	Problem solving	Feedback on behaviour	self monitoring of behaviour	social support	Instruction on how to perform the behaviour	Information abour social & environmental consequences		
2	2	Goal setting (behaviour)	Goal setting (outcome)	social support	Adding object to the environment				
0	0	Goal setting (behaviour)	Action planning	Information about health consequences	Adding object to the environment				
0	0	Goal setting (behaviour)	Problem solving	Feedback on behaviour	self monitoring of behaviour	social support	Instruction on how to perform the behaviour	Information about health consequences	Information abour social & environmental consequence
0	0	Problem solving	Feedback on behaviour	self monitoring of behaviour	social support	Instruction on how to perform the behaviour	Information about health consequences	Information abour social & environmental consequences	
0	0	Goal setting (behaviour)	Feedback on behaviour	self monitoring of behaviour	social support	Instruction on how to perform the behaviour	Information about health consequences	Information abour social & environmental consequences	
0	0	Goal setting (behaviour)	self monitoring of behaviour	Information about health consequences	Pros and cons				
0	0	Goal setting (behaviour)	self monitoring of behaviour	Information abour social & environmental consequences	Adding object to the environment				
3	2	Feedback on behaviour	Feedback on outcomes of behaviour	Instruction on how to perform the behaviour					
0	0	Goal setting (behaviour)	Action planning	social support	Instruction on how to perform the behaviour	Information about health consequences	Information abour social & environmental consequences	Information about emotinal consequences	
0	0	Goal setting (behaviour)	Action planning	social support	Instruction on how to perform the behaviour	Information about health consequences	Information abour social & environmental consequences	Social comparison	
0	0	Goal setting (behaviour)	Action planning	social support	Instruction on how to perform the behaviour	Information about health consequences	Information about emotinal consequences	Social comparison	
0	0	Goal setting (behaviour)	Problem solving	social support	Instruction on how to perform the behaviour	Information abour social & environmental consequences	Social comparison	Behavioural practice/rehearsal	
0	0	Goal setting (behaviour)	Problem solving	social support	Instruction on how to perform the behaviour	Information about emotinal consequences	Social comparison	Behavioural practice/rehearsal	
0	0	Goal setting (behaviour)	Problem solving	social support	Information about health consequences	Information abour social & environmental consequences	Information about emotinal consequences	Social comparison	
0	0	Goal setting (behaviour)	Action planning	social support	Instruction on how to perform the behaviour	Information about emotinal consequences	Social comparison	Behavioural practice/rehearsal	
0	0	Goal setting (behaviour)	Action planning	social support	Information about health consequences	Information abour social & environmental consequences	Information about emotinal consequences	Social comparison	
0	0	Goal setting (behaviour)	Problem solving	social support	Information about health consequences	Information about emotinal consequences	Social comparison	Behavioural practice/rehearsal	
0	0	Goal setting (behaviour)	Problem solving	social support	Information abour social & environmental consequences	Information about emotinal consequences	Social comparison	Behavioural practice/rehearsal	
0	0	Goal setting (behaviour)	Problem solving	Instruction on how to perform the behaviour	Information about health consequences	Information abour social & environmental consequences	Information about emotinal consequences	Social comparison	
0	0	Goal setting (behaviour)	Problem solving	Action planning	Information about health consequences	Information about emotinal consequences	Social comparison	Behavioural practice/rehearsal	
0	0	Goal setting (behaviour)	Problem solving	Action planning	Information abour social & environmental consequences	Information about emotinal consequences	Social comparison	Behavioural practice/rehearsal	
0	0	Goal setting (behaviour)	Problem solving	social support	Instruction on how to perform the behaviour	Information about health consequences	Information abour social & environmental consequences	Information about emotinal consequences	
0	0	Goal setting (behaviour)	Problem solving	Instruction on how to perform the behaviour	Information abour social & environmental consequences	Information about emotinal consequences	Social comparison	Behavioural practice/rehearsal	
0	0	Goal setting (behaviour)	Problem solving	Information about health consequences	Information abour social & environmental consequences	Information about emotinal consequences	Social comparison	Behavioural practice/rehearsal	
2	2	Action planning	self monitoring of behaviour	Instruction on how to perform the behaviour	Demonstration of the behaviour	Credible source	Adding object to the environment		
0	0	Goal setting (behaviour)	Action planning	Behavioural practice/rehearsal	Adding object to the environment				
1	1	Goal setting (behaviour)	Discrepancy between current behaviour and goal	Information about health consequences	Credible source				
1	1	Goal setting (behaviour)	Feedback on behaviour	Instruction on how to perform the behaviour	Prompts/cues	Graded tasks	Social reward		
1	1	Goal setting (behaviour)	Feedback on behaviour	Information about health consequences	Demonstration of the behaviour				
1	1	Goal setting (behaviour)	Discrepancy between current behaviour and goal	Prompts/cues	Credible source				
0	0	Goal setting (behaviour)	Problem solving	social support	Information about health consequences	Information abour social & environmental consequences	Information about emotinal consequences	Behavioural practice/rehearsal	
0	0	Goal setting (behaviour)	Problem solving	social support	Information about health consequences	Information abour social & environmental consequences	Social comparison	Behavioural practice/rehearsal	
2	2	Goal setting (outcome)	Feedback on behaviour	Self monitoring of outcome(s) of behaviour					
2	2	Problem solving	Instruction on how to perform the behaviour	Generalisation of target behaviour					

0	0	Goal setting (behaviour)	Problem solving	Action planning	social support	Instruction on how to perform the behaviour	Information about emotinal consequences	Social comparison	Behavioural practice/rehearsal
0	0	Action planning	social support	Instruction on how to perform the behaviour	Information about health consequences	Information abour social & environmental consequences	Information about emotinal consequences	Social comparison	
0	0	Action planning	social support	Instruction on how to perform the behaviour	Information about health consequences	Information abour social & environmental consequences	Information about emotinal consequences	Behavioural practice/rehearsal	
0	0	Action planning	social support	Instruction on how to perform the behaviour	Information about health consequences	Information abour social & environmental consequences	Social comparison	Behavioural practice/rehearsal	
0	0	Action planning	social support	Instruction on how to perform the behaviour	Information about health consequences	Information about emotinal consequences	Social comparison	Behavioural practice/rehearsal	
0	0	self monitoring of behaviour	social support	Information abour social & environmental consequences	Adding object to the environment				
0	0	Action planning	self monitoring of behaviour	Behavioural practice/rehearsal	Adding object to the environment				
0	0	Action planning	Instruction on how to perform the behaviour	Information about health consequences	Information abour social & environmental consequences	Information about emotinal consequences	Social comparison	Behavioural practice/rehearsal	
0	0	social support	Instruction on how to perform the behaviour	Information about health consequences	Information abour social & environmental consequences	Information about emotinal consequences	Social comparison	Behavioural practice/rehearsal	
0	0	Problem solving	Action planning	Information about health consequences	Information abour social & environmental consequences	Information about emotinal consequences	Social comparison	Behavioural practice/rehearsal	
0	0	Problem solving	social support	Instruction on how to perform the behaviour	Information about health consequences	Information abour social & environmental consequences	Information about emotinal consequences	Social comparison	
0	0	Problem solving	social support	Instruction on how to perform the behaviour	Information about health consequences	Information abour social & environmental consequences	Information about emotinal consequences	Behavioural practice/rehearsal	
0	0	Problem solving	social support	Instruction on how to perform the behaviour	Information about health consequences	Information abour social & environmental consequences	Social comparison	Behavioural practice/rehearsal	
0	0	Problem solving	social support	Information about health consequences	Information abour social & environmental consequences	Information about emotinal consequences	Social comparison	Behavioural practice/rehearsal	
0	0	Problem solving	Instruction on how to perform the behaviour	Information about health consequences	Information abour social & environmental consequences	Information about emotinal consequences	Social comparison	Behavioural practice/rehearsal	
1	1	Goal setting (behaviour)	Action planning	Discrepancy between current behaviour and goal	Information about health consequences				
1	1	Goal setting (behaviour)	Action planning	Discrepancy between current behaviour and goal	Prompts/cues				
0	0	Action planning	social support	Instruction on how to perform the behaviour	Information abour social & environmental consequences	Information about emotinal consequences	Social comparison	Behavioural practice/rehearsal	
0	0	Action planning	social support	Information about health consequences	Information abour social & environmental consequences	Information about emotinal consequences	Social comparison	Behavioural practice/rehearsal	
1	0	social support	Instruction on how to perform the behaviour	Information about health consequences	Pros and cons				
0	0	self monitoring of behaviour	Instruction on how to perform the behaviour	Information abour social & environmental consequences	Adding object to the environment				
1	1	Goal setting (behaviour)	Action planning	Review of behaviour goals	Discrepancy between current behaviour and goal				
1	1	Goal setting (behaviour)	Goal setting (outcome)	self monitoring of behaviour	Information about health consequences				
1	1	Goal setting (behaviour)	Goal setting (outcome)	self monitoring of behaviour	Behavioural practice/rehearsal				
1	1	Goal setting (behaviour)	Goal setting (outcome)	Instruction on how to perform the behaviour	Information about health consequences				
1	1	Goal setting (behaviour)	Goal setting (outcome)	Instruction on how to perform the behaviour	Behavioural practice/rehearsal				
3	3	Goal setting (behaviour)	Goal setting (outcome)	Action planning	Demonstration of the behaviour				
0	0	Problem solving	social support	Instruction on how to perform the behaviour	Information about health consequences	Information about emotinal consequences	Social comparison	Behavioural practice/rehearsal	
0	0	Problem solving	social support	Instruction on how to perform the behaviour	Information abour social & environmental consequences	Information about emotinal consequences	Social comparison	Behavioural practice/rehearsal	
2	2	Problem solving	Goal setting (outcome)	Action planning	self monitoring of behaviour	Instruction on how to perform the behaviour	Adding object to the environment		
2	2	Goal setting (behaviour)	social support	Instruction on how to perform the behaviour	Demonstration of the behaviour	Credible source	Adding object to the environment		
2	2	Problem solving	Goal setting (outcome)	Action planning	self monitoring of behaviour	social support	Instruction on how to perform the behaviour		

2	2	Problem solving	self monitoring of behaviour	social support	Credible source	Adding object to the environment			
1	1	Action planning	Review of behaviour goals	social support	Instruction on how to perform the behaviour	Credible source			
3	3	Action planning	Review of behaviour goals	Instruction on how to perform the behaviour	Demonstration of the behaviour	Credible source			
3	3	Action planning	Review of behaviour goals	Instruction on how to perform the behaviour	Behavioural practice/rehearsal	Credible source			
2	2	Goal setting (behaviour)	Review of behaviour goals	Instruction on how to perform the behaviour	Demonstration of the behaviour	Behavioural practice/rehearsal			
2	2	Goal setting (behaviour)	Review of behaviour goals	Instruction on how to perform the behaviour	Behavioural practice/rehearsal	Credible source			
0	0	Problem solving	self monitoring of behaviour	social support	Information abour social & environmental consequences	Adding object to the environment			
2	2	Goal setting (behaviour)	Problem solving	social support	Instruction on how to perform the behaviour	Demonstration of the behaviour	Graded tasks	Adding object to the environment	
0	0	Problem solving	Action planning	social support	Behavioural practice/rehearsal	Credible source			
0	0	Problem solving	Action planning	social support	Behavioural practice/rehearsal	Adding object to the environment			
2	2	Problem solving	Action planning	social support	Credible source	Adding object to the environment			
0	0	Problem solving	Action planning	Instruction on how to perform the behaviour	Information about health consequences	Adding object to the environment			
0	0	Goal setting (behaviour)	Problem solving	self monitoring of behaviour	social support	Behavioural practice/rehearsal	Credible source		
0	0	Goal setting (behaviour)	Problem solving	self monitoring of behaviour	social support	Behavioural practice/rehearsal	Adding object to the environment		
2	2	Goal setting (behaviour)	Problem solving	Action planning	Demonstration of the behaviour	Credible source	Adding object to the environment		
0	0	Problem solving	self monitoring of behaviour	social support	Behavioural practice/rehearsal	Adding object to the environment			
2	2	Problem solving	Action planning	Demonstration of the behaviour	Credible source	Adding object to the environment			
2	2	Problem solving	Action planning	Instruction on how to perform the behaviour	Demonstration of the behaviour	Adding object to the environment			
0	0	Problem solving	Action planning	Instruction on how to perform the behaviour	Behavioural practice/rehearsal	Adding object to the environment			
2	2	Problem solving	Action planning	Instruction on how to perform the behaviour	Credible source	Adding object to the environment			
3	3	Review of behaviour goals	Demonstration of the behaviour	Behavioural practice/rehearsal	Credible source				
0	0	Goal setting (behaviour)	Problem solving	Action planning	social support	Demonstration of the behaviour	Behavioural practice/rehearsal		
2	2	Goal setting (behaviour)	Problem solving	Action planning	social support	Demonstration of the behaviour	Credible source		
2	2	Goal setting (behaviour)	Problem solving	Action planning	social support	Demonstration of the behaviour	Adding object to the environment		
1	1	Goal setting (behaviour)	Feedback on behaviour	Instruction on how to perform the behaviour	Prompts/cues	Adding object to the environment			
2	2	Action planning	Feedback on behaviour	self monitoring of behaviour	Demonstration of the behaviour	Adding object to the environment			
4	4	Goal setting (behaviour)	Goal setting (outcome)	Feedback on behaviour	self monitoring of behaviour	Instruction on how to perform the behaviour	Demonstration of the behaviour	Graded tasks	Credible source
4	4	Goal setting (behaviour)	Problem solving	Goal setting (outcome)	self monitoring of behaviour	Instruction on how to perform the behaviour	Demonstration of the behaviour	Graded tasks	Credible source
1	1	Action planning	social support	Instruction on how to perform the behaviour	Prompts/cues	Adding object to the environment			
0	0	Problem solving	social support	Information about health consequences	Information abour social & environmental consequences	Adding object to the environment			
1	0	Problem solving	social support	Instruction on how to perform the behaviour	Information about health consequences	Pros and cons			
0	0	Problem solving	social support	Instruction on how to perform the behaviour	Information abour social & environmental consequences	Adding object to the environment			
1	1	Goal setting (outcome)	Feedback on behaviour	Information about health consequences					
2	2	Goal setting (outcome)	Feedback on behaviour	Behavioural practice/rehearsal					
1	1	Goal setting (outcome)	self monitoring of behaviour	Information about health consequences					
4	4	Goal setting (behaviour)	Problem solving	Goal setting (outcome)	Feedback on behaviour	Instruction on how to perform the behaviour	Demonstration of the behaviour	Graded tasks	Credible source
1	1	Goal setting (behaviour)	Action planning	self monitoring of behaviour	Prompts/cues	Adding object to the environment			
1	1	Goal setting (behaviour)	Action planning	social support	Prompts/cues	Adding object to the environment			
1	1	Goal setting (behaviour)	Action planning	Instruction on how to perform the behaviour	Prompts/cues	Adding object to the environment			
1	1	Action planning	self monitoring of behaviour	Instruction on how to perform the behaviour	Prompts/cues	Adding object to the environment			
1	0	Problem solving	social support	Instruction on how to perform the behaviour	Demonstration of the behaviour	Behavioural practice/rehearsal			
0	0	Problem solving	self monitoring of behaviour	Information about health consequences	Information abour social & environmental consequences	Behavioural practice/rehearsal			
0	0	Problem solving	self monitoring of behaviour	Information about health consequences	Information abour social & environmental consequences	Adding object to the environment			
2	2	Problem solving	self monitoring of behaviour	Demonstration of the behaviour	Credible source	Adding object to the environment			
1	1	Action planning	Instruction on how to perform the behaviour	Prompts/cues	Adding object to the environment				
1	1	Feedback on behaviour	Instruction on how to perform the behaviour	Prompts/cues	Adding object to the environment				
2	2	Action planning	Feedback on behaviour	Demonstration of the behaviour	Adding object to the environment				
1	1	Action planning	self monitoring of behaviour	Prompts/cues	Adding object to the environment				
1	1	Action planning	Review of behaviour goals	social support	Credible source				
2	2	Goal setting (outcome)	Action planning	social support	Instruction on how to perform the behaviour	Adding object to the environment			
0	0	Problem solving	Instruction on how to perform the behaviour	Information about health consequences	Behavioural practice/rehearsal	Adding object to the environment			

2	2	Goal setting (behaviour)	Goal setting (outcome)	self monitoring of behaviour	social support	Instruction on how to perform the behaviour	Demonstration of the behaviour
2	2	Goal setting (behaviour)	Goal setting (outcome)	self monitoring of behaviour	social support	Demonstration of the behaviour	Credible source
2	2	Goal setting (behaviour)	Goal setting (outcome)	self monitoring of behaviour	social support	Demonstration of the behaviour	Adding object to the environment
2	2	Goal setting (behaviour)	Goal setting (outcome)	self monitoring of behaviour	social support	Credible source	Adding object to the environment
2	2	Goal setting (behaviour)	Goal setting (outcome)	self monitoring of behaviour	Instruction on how to perform the behaviour	Demonstration of the behaviour	Adding object to the environment
0	0	Goal setting (behaviour)	Goal setting (outcome)	self monitoring of behaviour	Instruction on how to perform the behaviour	Behavioural practice/rehearsal	Adding object to the environment
2	2	Goal setting (behaviour)	Goal setting (outcome)	self monitoring of behaviour	Instruction on how to perform the behaviour	Credible source	Adding object to the environment
2	2	Goal setting (behaviour)	Goal setting (outcome)	self monitoring of behaviour	Demonstration of the behaviour	Credible source	Adding object to the environment
0	0	Goal setting (behaviour)	Action planning	social support	Behavioural practice/rehearsal	Generalisation of target behaviour	Credible source
0	0	Goal setting (behaviour)	Action planning	Instruction on how to perform the behaviour	Information about health consequences	Information abour social & environmental consequences	Adding object to the environment
0	0	Goal setting (behaviour)	Action planning	Instruction on how to perform the behaviour	Information about health consequences	Behavioural practice/rehearsal	Adding object to the environment
0	0	Goal setting (behaviour)	Action planning	Instruction on how to perform the behaviour	Information abour social & environmental consequences	Behavioural practice/rehearsal	Credible source
0	0	Goal setting (behaviour)	Action planning	Instruction on how to perform the behaviour	Information abour social & environmental consequences	Behavioural practice/rehearsal	Adding object to the environment
0	0	Goal setting (behaviour)	Action planning	Information about health consequences	Information abour social & environmental consequences	Behavioural practice/rehearsal	Adding object to the environment
0	0	Goal setting (behaviour)	self monitoring of behaviour	social support	Instruction on how to perform the behaviour	Information about health consequences	Demonstration of the behaviour
0	0	Goal setting (behaviour)	Problem solving	Information abour social & environmental consequences	Behavioural practice/rehearsal	Adding object to the environment	
0	0	Goal setting (behaviour)	Action planning	social support	Instruction on how to perform the behaviour	Information abour social & environmental consequences	Adding object to the environment
0	0	Goal setting (behaviour)	Action planning	social support	Instruction on how to perform the behaviour	Behavioural practice/rehearsal	Generalisation of target behaviour
0	0	Goal setting (behaviour)	Action planning	social support	Instruction on how to perform the behaviour	Generalisation of target behaviour	Credible source
0	0	Goal setting (behaviour)	Action planning	social support	Information about health consequences	Information abour social & environmental consequences	Adding object to the environment
0	0	Goal setting (behaviour)	Action planning	social support	Information about health consequences	Behavioural practice/rehearsal	Credible source
0	0	Goal setting (behaviour)	Action planning	social support	Information about health consequences	Behavioural practice/rehearsal	Adding object to the environment
0	0	Goal setting (behaviour)	Action planning	social support	Information abour social & environmental consequences	Behavioural practice/rehearsal	Credible source
0	0	Goal setting (behaviour)	Action planning	social support	Information abour social & environmental consequences	Behavioural practice/rehearsal	Adding object to the environment
0	0	Goal setting (behaviour)	Action planning	self monitoring of behaviour	social support	Information abour social & environmental consequences	Adding object to the environment
0	0	Goal setting (behaviour)	Action planning	self monitoring of behaviour	Instruction on how to perform the behaviour	Information abour social & environmental consequences	Credible source
0	0	Goal setting (behaviour)	Action planning	self monitoring of behaviour	Instruction on how to perform the behaviour	Information abour social & environmental consequences	Adding object to the environment
0	0	Goal setting (behaviour)	Action planning	self monitoring of behaviour	Information about health consequences	Information abour social & environmental consequences	Adding object to the environment
0	0	Goal setting (behaviour)	Action planning	self monitoring of behaviour	Information about health consequences	Behavioural practice/rehearsal	Adding object to the environment
0	0	Goal setting (behaviour)	Action planning	self monitoring of behaviour	Information abour social & environmental consequences	Behavioural practice/rehearsal	Credible source
0	0	Goal setting (behaviour)	Action planning	self monitoring of behaviour	Information abour social & environmental consequences	Behavioural practice/rehearsal	Adding object to the environment
0	0	Goal setting (behaviour)	Action planning	social support	Instruction on how to perform the behaviour	Information abour social & environmental consequences	Credible source
0	0	Goal setting (behaviour)	Action planning	self monitoring of behaviour	Information abour social & environmental consequences	Credible source	
0	0	Goal setting (behaviour)	Action planning	self monitoring of behaviour	Information abour social & environmental consequences	Adding object to the environment	
0	0	Goal setting (behaviour)	Action planning	social support	Instruction on how to perform the behaviour	Generalisation of target behaviour	
0	0	Goal setting (behaviour)	Action planning	social support	Information abour social & environmental consequences	Credible source	
0	0	Goal setting (behaviour)	Action planning	social support	Information abour social & environmental consequences	Adding object to the environment	
2	2	Goal setting (behaviour)	Goal setting (outcome)	social support	Demonstration of the behaviour	Credible source	Adding object to the environment
2	2	Goal setting (behaviour)	Goal setting (outcome)	Instruction on how to perform the behaviour	Demonstration of the behaviour	Credible source	Adding object to the environment
0	0	Goal setting (behaviour)	Action planning	self monitoring of behaviour	social support	Information abour social & environmental consequences	Credible source
0	0	Goal setting (behaviour)	self monitoring of behaviour	social support	Information about health consequences	Behavioural practice/rehearsal	Adding object to the environment
0	0	Goal setting (behaviour)	self monitoring of behaviour	social support	Information abour social & environmental consequences	Behavioural practice/rehearsal	Credible source
0	0	Goal setting (behaviour)	self monitoring of behaviour	social support	Information abour social & environmental consequences	Behavioural practice/rehearsal	Adding object to the environment
0	0	Goal setting (behaviour)	self monitoring of behaviour	Instruction on how to perform the behaviour	Information about health consequences	Behavioural practice/rehearsal	Adding object to the environment
0	0	Goal setting (behaviour)	self monitoring of behaviour	Instruction on how to perform the behaviour	Information abour social & environmental consequences	Behavioural practice/rehearsal	Credible source
0	0	Goal setting (behaviour)	self monitoring of behaviour	Instruction on how to perform the behaviour	Information abour social & environmental consequences	Behavioural practice/rehearsal	Adding object to the environment
0	0	Goal setting (behaviour)	self monitoring of behaviour	Information about health consequences	Information abour social & environmental consequences	Behavioural practice/rehearsal	Adding object to the environment
1	0	social support	Information about health consequences	Credible source	Pros and cons		
0	0	Goal setting (behaviour)	Problem solving	Instruction on how to perform the behaviour	Information about health consequences	Pros and cons	
0	0	Goal setting (behaviour)	Problem solving	Action planning	Information abour social & environmental consequences	Adding object to the environment	
0	0	Goal setting (behaviour)	Problem solving	self monitoring of behaviour	social support	Pros and cons	
0	0	Goal setting (behaviour)	Problem solving	self monitoring of behaviour	Instruction on how to perform the behaviour	Pros and cons	
0	0	Goal setting (behaviour)	Problem solving	self monitoring of behaviour	Information about health consequences	Pros and cons	
0	0	Goal setting (behaviour)	self monitoring of behaviour	social support	Instruction on how to perform the behaviour	Information about health consequences	Pros and cons
0	0	Goal setting (behaviour)	self monitoring of behaviour	social support	Instruction on how to perform the behaviour	Information abour social & environmental consequences	Credible source
0	0	Goal setting (behaviour)	self monitoring of behaviour	social support	Information about health consequences	Demonstration of the behaviour	Credible source
0	0	Goal setting (behaviour)	Goal setting (outcome)	self monitoring of behaviour	Behavioural practice/rehearsal	Adding object to the environment	
2	2	Goal setting (behaviour)	Goal setting (outcome)	self monitoring of behaviour	Credible source	Adding object to the environment	
0	0	Goal setting (behaviour)	Goal setting (outcome)	social support	Instruction on how to perform the behaviour	Information about health consequences	
2	2	Goal setting (behaviour)	Goal setting (outcome)	social support	Demonstration of the behaviour	Credible source	
2	2	Goal setting (behaviour)	Goal setting (outcome)	social support	Demonstration of the behaviour	Adding object to the environment	
2	2	Goal setting (behaviour)	Goal setting (outcome)	social support	Credible source	Adding object to the environment	
0	0	Goal setting (behaviour)	Problem solving	social support	Instruction on how to perform the behaviour	Pros and cons	
0	0	Goal setting (behaviour)	Problem solving	social support	Information about health consequences	Pros and cons	
0	0	Goal setting (behaviour)	Problem solving	Behavioural practice/rehearsal	Credible source	Adding object to the environment	
2	2	Goal setting (behaviour)	Goal setting (outcome)	Action planning	social support	Demonstration of the behaviour	
2	2	Goal setting (behaviour)	Goal setting (outcome)	Action planning	social support	Credible source	
2	2	Goal setting (behaviour)	Goal setting (outcome)	Action planning	Demonstration of the behaviour	Adding object to the environment	
2	2	Goal setting (behaviour)	Goal setting (outcome)	Action planning	Credible source	Adding object to the environment	
2	2	Goal setting (behaviour)	Goal setting (outcome)	self monitoring of behaviour	social support	Demonstration of the behaviour	
0	0	Goal setting (behaviour)	Goal setting (outcome)	self monitoring of behaviour	Information about health consequences	Adding object to the environment	
2	2	Goal setting (behaviour)	Goal setting (outcome)	self monitoring of behaviour	Demonstration of the behaviour	Adding object to the environment	
2	2	Problem solving	Goal setting (outcome)	Action planning	self monitoring of behaviour	Credible source	Adding object to the environment
2	2	Problem solving	Goal setting (outcome)	Action planning	social support	Instruction on how to perform the behaviour	Demonstration of the behaviour
2	2	Problem solving	Goal setting (outcome)	Action planning	social support	Instruction on how to perform the behaviour	Credible source
2	2	Problem solving	Goal setting (outcome)	Action planning	social support	Demonstration of the behaviour	Credible source
2	2	Problem solving	Goal setting (outcome)	Action planning	social support	Demonstration of the behaviour	Adding object to the environment
2	2	Problem solving	Goal setting (outcome)	Action planning	social support	Credible source	Adding object to the environment
2	2	Problem solving	Goal setting (outcome)	Action planning	Instruction on how to perform the behaviour	Demonstration of the behaviour	Adding object to the environment
2	2	Problem solving	Goal setting (outcome)	Action planning	Instruction on how to perform the behaviour	Credible source	Adding object to the environment
0	0	Goal setting (behaviour)	social support	Instruction on how to perform the behaviour	Information abour social & environmental consequences	Behavioural practice/rehearsal	Adding object to the environment
0	0	Goal setting (behaviour)	social support	Instruction on how to perform the behaviour	Behavioural practice/rehearsal	Generalisation of target behaviour	Credible source
0	0	Goal setting (behaviour)	social support	Instruction on how to perform the behaviour	Behavioural practice/rehearsal	Credible source	Adding object to the environment
0	0	Goal setting (behaviour)	social support	Information about health consequences	Information abour social & environmental consequences	Behavioural practice/rehearsal	Adding object to the environment
0	0	Goal setting (behaviour)	Instruction on how to perform the behaviour	Information about health consequences	Information abour social & environmental consequences	Behavioural practice/rehearsal	Adding object to the environment
2	2	Problem solving	Goal setting (outcome)	Action planning	self monitoring of behaviour	social support	Demonstration of the behaviour
2	2	Problem solving	Goal setting (outcome)	Action planning	self monitoring of behaviour	social support	Credible source
2	2	Problem solving	Goal setting (outcome)	Action planning	self monitoring of behaviour	Demonstration of the behaviour	Adding object to the environment
0	0	Instruction on how to perform the behaviour	Demonstration of the behaviour	Behavioural practice/rehearsal	Adding object to the environment		
0	0	Instruction on how to perform the behaviour	Behavioural practice/rehearsal	Credible source	Adding object to the environment		
0	0	Information about health consequences	Information abour social & environmental consequences	Behavioural practice/rehearsal	Adding object to the environment		
0	0	Goal setting (behaviour)	Problem solving	Goal setting (outcome)	social support	Information about health consequences	
0	0	Goal setting (behaviour)	Problem solving	Goal setting (outcome)	Information about health consequences	Adding object to the environment	
2	2	Goal setting (behaviour)	Problem solving	Goal setting (outcome)	Demonstration of the behaviour	Adding object to the environment	
2	2	Goal setting (behaviour)	Problem solving	Goal setting (outcome)	Credible source	Adding object to the environment	
0	0	Goal setting (behaviour)	social support	Instruction on how to perform the behaviour	Information abour social & environmental consequences	Behavioural practice/rehearsal	Credible source
0	0	social support	Information about health consequences	Credible source	Adding object to the environment		
0	0	social support	Information abour social & environmental consequences	Behavioural practice/rehearsal	Credible source		
0	0	social support	Information abour social & environmental consequences	Behavioural practice/rehearsal	Adding object to the environment		
0	0	social support	Behavioural practice/rehearsal	Generalisation of target behaviour	Credible source		
0	0	social support	Behavioural practice/rehearsal	Credible source	Adding object to the environment		
1	0	Instruction on how to perform the behaviour	Information about health consequences	Credible source	Pros and cons		
0	0	Instruction on how to perform the behaviour	Information abour social & environmental consequences	Behavioural practice/rehearsal	Credible source		
0	0	Instruction on how to perform the behaviour	Information abour social & environmental consequences	Behavioural practice/rehearsal	Adding object to the environment		
0	0	Action planning	social support	Instruction on how to perform the behaviour	Generalisation of target behaviour		
1	0	Action planning	social support	Instruction on how to perform the behaviour	Pros and cons		
1	0	Action planning	social support	Information about health consequences	Demonstration of the behaviour		
1	0	Action planning	social support	Information about health consequences	Pros and cons		
0	0	Action planning	social support	Information abour social & environmental consequences	Credible source		
0	0	Action planning	social support	Information abour social & environmental consequences	Adding object to the environment		
0	0	Action planning	social support	Behavioural practice/rehearsal	Generalisation of target behaviour		
0	0	Action planning	social support	Generalisation of target behaviour	Credible source		

0	0	Goal setting (behaviour)	social support (practical	Instruction on how to perform the behaviour	Behavioural practice/rehearsal							33	0	0	0	0	0	0	4
0	0	Goal setting (behaviour)	social support (practical	Instruction on how to perform the behaviour	Credible source							33	0	0	0	0	0	0	4
0	0	Goal setting (behaviour)	social support (practical	Information about health consequences	Credible source							67	0	0	0	0	0	0	4
0	0	Goal setting (behaviour)	Problem solving	Action planning	social support	Instruction on how to perform the behaviour	Demonstration of the behaviour	Behavioural practice/rehearsal				67	0	0	0	0	0	0	7
0	0	Goal setting (behaviour)	Problem solving	Action planning	social support	Information about health consequences	Information abour social & environmental consequences	Adding object to the environment				67	0	0	0	0	0	0	7
0	0	Goal setting (behaviour)	Problem solving	Action planning	social support	Information about health consequences	Behavioural practice/rehearsal	Adding object to the environment				67	0	0	0	0	0	0	7
0	0	Goal setting (behaviour)	Problem solving	Action planning	social support	Information abour social & environmental consequences	Behavioural practice/rehearsal	Adding object to the environment				67	0	0	0	0	0	0	7
0	0	Goal setting (behaviour)	Problem solving	Action planning	Instruction on how to perform the behaviour	Information about health consequences	Information abour social & environmental consequences	Adding object to the environment				67	0	0	0	0	0	0	7
0	0	Goal setting (behaviour)	Problem solving	Action planning	Instruction on how to perform the behaviour	Information about health consequences	Behavioural practice/rehearsal	Adding object to the environment				67	0	0	0	0	0	0	7
0	0	Goal setting (behaviour)	Problem solving	Action planning	Instruction on how to perform the behaviour	Information abour social & environmental consequences	Behavioural practice/rehearsal	Adding object to the environment				67	0	0	0	0	0	0	7
0	0	Goal setting (behaviour)	Problem solving	Action planning	Information about health consequences	Information abour social & environmental consequences	Behavioural practice/rehearsal	Adding object to the environment				67	0	0	0	0	0	0	7
2	2	Action planning	Review of behaviour goals	Feedback on behaviour	self monitoring of behaviour	Instruction on how to perform the behaviour	Information about health consequences	Behavioural practice/rehearsal	Graded tasks	Reduce negative emotions		0	0	100	0	0	0	0	9
2	2	Action planning	Review of behaviour goals	Feedback on behaviour	self monitoring of behaviour	Instruction on how to perform the behaviour	Demonstration of the behaviour	Behavioural practice/rehearsal	Graded tasks	Credible source		100	0	100	0	0	0	0	9
3	3	Goal setting (behaviour)	Problem solving	Goal setting (outcome)	Action planning	Feedback on behaviour	self monitoring of behaviour	Instruction on how to perform the behaviour	Demonstration of the behaviour	Graded tasks	Credible source	100	100	100	0	0	0	0	10
1	1	Goal setting (behaviour)	Problem solving	Action planning	Review of behaviour goals	Discrepancy between current behaviour and goal	Feedback on behaviour	self monitoring of behaviour	Instruction on how to perform the behaviour	Information about health consequences	Prompts/cues	50	0	100	0	0	0	0	10
1	1	Goal setting (behaviour)	Problem solving	Action planning	Review of behaviour goals	Discrepancy between current behaviour and goal	Feedback on behaviour	self monitoring of behaviour	Instruction on how to perform the behaviour	Information about health consequences	Credible source	50	0	100	0	0	0	0	10
1	1	Goal setting (behaviour)	Problem solving	Action planning	Review of behaviour goals	Discrepancy between current behaviour and goal	Feedback on behaviour	self monitoring of behaviour	Instruction on how to perform the behaviour	Prompts/cues	Credible source	50	0	100	0	0	0	0	10
1	1	Goal setting (behaviour)	Problem solving	Action planning	Review of behaviour goals	Discrepancy between current behaviour and goal	Feedback on behaviour	self monitoring of behaviour	Information about health consequences	Prompts/cues	Credible source	50	0	100	0	0	0	0	10
1	1	Goal setting (behaviour)	Problem solving	Action planning	Review of behaviour goals	Discrepancy between current behaviour and goal	Feedback on behaviour	Instruction on how to perform the behaviour	Information about health consequences	Prompts/cues	Credible source	50	0	100	0	0	0	0	10
2	2	Goal setting (behaviour)	Goal setting (outcome)	self monitoring of behaviour	social support	Instruction on how to perform the behaviour	Demonstration of the behaviour	Credible source				100	100	100	0	0	0	0	7
2	2	Goal setting (behaviour)	Goal setting (outcome)	self monitoring of behaviour	social support	Instruction on how to perform the behaviour	Demonstration of the behaviour	Adding object to the environment				100	100	100	0	0	0	0	7
2	2	Goal setting (behaviour)	Goal setting (outcome)	self monitoring of behaviour	social support	Instruction on how to perform the behaviour	Credible source	Adding object to the environment				100	100	100	0	0	0	0	7
2	2	Goal setting (behaviour)	Goal setting (outcome)	self monitoring of behaviour	social support	Demonstration of the behaviour	Credible source	Adding object to the environment				100	100	100	0	0	0	0	7
2	2	Goal setting (behaviour)	Goal setting (outcome)	self monitoring of behaviour	Instruction on how to perform the behaviour	Demonstration of the behaviour	Credible source	Adding object to the environment				100	100	100	0	0	0	0	7
2	2	Goal setting (behaviour)	Goal setting (outcome)	social support	Instruction on how to perform the behaviour	Demonstration of the behaviour	Credible source	Adding object to the environment				100	100	100	0	0	0	0	7
0	0	Goal setting (behaviour)	Action planning	self monitoring of behaviour	social support	Instruction on how to perform the behaviour	Information abour social & environmental consequences	Credible source				67	0	0	0	0	0	0	7
0	0	Goal setting (behaviour)	Action planning	self monitoring of behaviour	social support	Instruction on how to perform the behaviour	Information abour social & environmental consequences	Adding object to the environment				67	0	0	0	0	0	0	7
2	2	Goal setting (behaviour)	Goal setting (outcome)	Action planning	self monitoring of behaviour	Instruction on how to perform the behaviour	Demonstration of the behaviour	Adding object to the environment				100	100	100	0	0	0	0	7
2	2	Goal setting (behaviour)	Goal setting (outcome)	Action planning	self monitoring of behaviour	Instruction on how to perform the behaviour	Credible source	Adding object to the environment				100	100	100	0	0	0	0	7
2	2	Goal setting (behaviour)	Goal setting (outcome)	Action planning	self monitoring of behaviour	Demonstration of the behaviour	Credible source	Adding object to the environment				100	100	100	0	0	0	0	7
2	2	Goal setting (behaviour)	Goal setting (outcome)	Action planning	social support	Instruction on how to perform the behaviour	Demonstration of the behaviour	Credible source				100	100	100	0	0	0	0	7
2	2	Goal setting (behaviour)	Goal setting (outcome)	Action planning	social support	Instruction on how to perform the behaviour	Demonstration of the behaviour	Adding object to the environment				100	100	100	0	0	0	0	7

1	1	Problem solving	Review of behaviour goals	Discrepancy between current behaviour and goal	Feedback on behaviour	self monitoring of behaviour	Instruction on how to perform the behaviour	Prompts/cues	Credible source
1	1	Problem solving	Review of behaviour goals	Discrepancy between current behaviour and goal	Feedback on behaviour	self monitoring of behaviour	Information about health consequences	Prompts/cues	Graded tasks
1	1	Problem solving	Review of behaviour goals	Discrepancy between current behaviour and goal	Feedback on behaviour	self monitoring of behaviour	Information about health consequences	Prompts/cues	Credible source
1	1	Problem solving	Review of behaviour goals	Discrepancy between current behaviour and goal	Feedback on behaviour	Instruction on how to perform the behaviour	Information about health consequences	Prompts/cues	Credible source
1	1	Problem solving	Review of behaviour goals	Discrepancy between current behaviour and goal	self monitoring of behaviour	Instruction on how to perform the behaviour	Information about health consequences	Prompts/cues	Credible source
1	1	Problem solving	Review of behaviour goals	Feedback on behaviour	self monitoring of behaviour	Instruction on how to perform the behaviour	Information about health consequences	Prompts/cues	Graded tasks
0	0	Feedback on behaviour	social support	Information about health consequences	Demonstration of the behaviour				
0	0	Feedback on behaviour	Information about health consequences	Prompts/cues	Adding object to the environment				
2	2	Problem solving	Action planning	Feedback on behaviour	self monitoring of behaviour	Instruction on how to perform the behaviour	Demonstration of the behaviour	Behavioural practice/rehearsal	Credible source
2	2	Problem solving	Action planning	Feedback on behaviour	self monitoring of behaviour	Instruction on how to perform the behaviour	Prompts/cues	Graded tasks	Credible source
2	2	Problem solving	Action planning	Feedback on behaviour	self monitoring of behaviour	Instruction on how to perform the behaviour	Behavioural practice/rehearsal	Graded tasks	Credible source
2	2	Problem solving	Action planning	Feedback on behaviour	self monitoring of behaviour	Demonstration of the behaviour	Behavioural practice/rehearsal	Graded tasks	Credible source
2	2	Problem solving	Action planning	Feedback on behaviour	Instruction on how to perform the behaviour	Demonstration of the behaviour	Behavioural practice/rehearsal	Graded tasks	Credible source
2	2	Problem solving	Action planning	self monitoring of behaviour	Instruction on how to perform the behaviour	Demonstration of the behaviour	Behavioural practice/rehearsal	Graded tasks	Credible source
1	1	Problem solving	Action planning	Feedback on behaviour	self monitoring of behaviour	Instruction on how to perform the behaviour	Information about health consequences	Prompts/cues	Graded tasks
2	2	Problem solving	Action planning	Feedback on behaviour	self monitoring of behaviour	Instruction on how to perform the behaviour	Demonstration of the behaviour	Prompts/cues	Graded tasks
0	0	Goal setting (behaviour)	self monitoring of behaviour	social support	social support (practical	Credible source			
0	0	Goal setting (behaviour)	self monitoring of behaviour	Information about health consequences	Prompts/cues	Adding object to the environment			
0	0	Goal setting (behaviour)	social support	Information about health consequences	Prompts/cues	Credible source			
0	0	Feedback on behaviour	self monitoring of behaviour	social support	social support (practical	Credible source			
0	0	Feedback on behaviour	self monitoring of behaviour	Information about health consequences	Prompts/cues	Adding object to the environment			
0	0	Feedback on behaviour	social support	Information about health consequences	Prompts/cues	Credible source			
1	1	Problem solving	Action planning	Review of behaviour goals	self monitoring of behaviour	Instruction on how to perform the behaviour	Information about health consequences	Prompts/cues	Graded tasks
1	1	Problem solving	Action planning	Review of behaviour goals	self monitoring of behaviour	Instruction on how to perform the behaviour	Information about health consequences	Prompts/cues	Credible source
2	2	Problem solving	Action planning	Review of behaviour goals	self monitoring of behaviour	Instruction on how to perform the behaviour	Demonstration of the behaviour	Behavioural practice/rehearsal	Credible source
2	2	Problem solving	Action planning	Review of behaviour goals	self monitoring of behaviour	Instruction on how to perform the behaviour	Demonstration of the behaviour	Graded tasks	Credible source
2	2	Problem solving	Action planning	Review of behaviour goals	self monitoring of behaviour	Instruction on how to perform the behaviour	Behavioural practice/rehearsal	Graded tasks	Credible source
2	2	Problem solving	Action planning	Review of behaviour goals	self monitoring of behaviour	Demonstration of the behaviour	Behavioural practice/rehearsal	Graded tasks	Credible source
2	2	Problem solving	Action planning	Review of behaviour goals	Instruction on how to perform the behaviour	Demonstration of the behaviour	Behavioural practice/rehearsal	Graded tasks	Credible source
2	2	Problem solving	Action planning	Discrepancy between current behaviour and goal	Feedback on behaviour	self monitoring of behaviour	Instruction on how to perform the behaviour	Graded tasks	Credible source
2	2	Problem solving	Action planning	Review of behaviour goals	Feedback on behaviour	self monitoring of behaviour	Demonstration of the behaviour	Graded tasks	Credible source
2	2	Problem solving	Action planning	Review of behaviour goals	Feedback on behaviour	self monitoring of behaviour	Behavioural practice/rehearsal	Graded tasks	Credible source
1	1	Problem solving	Action planning	Review of behaviour goals	Feedback on behaviour	Instruction on how to perform the behaviour	Information about health consequences	Prompts/cues	Graded tasks
1	1	Problem solving	Action planning	Review of behaviour goals	Feedback on behaviour	Instruction on how to perform the behaviour	Information about health consequences	Prompts/cues	Credible source
2	2	Problem solving	Action planning	Review of behaviour goals	Feedback on behaviour	Instruction on how to perform the behaviour	Demonstration of the behaviour	Behavioural practice/rehearsal	Credible source
2	2	Problem solving	Action planning	Review of behaviour goals	Feedback on behaviour	Instruction on how to perform the behaviour	Demonstration of the behaviour	Graded tasks	Credible source
2	2	Problem solving	Action planning	Review of behaviour goals	Feedback on behaviour	Instruction on how to perform the behaviour	Behavioural practice/rehearsal	Graded tasks	Credible source
2	2	Problem solving	Action planning	Review of behaviour goals	Feedback on behaviour	Demonstration of the behaviour	Behavioural practice/rehearsal	Graded tasks	Credible source
0	0	Goal setting (behaviour)	Feedback on behaviour	self monitoring of behaviour	social support (practical	Information about health consequences			
0	0	Goal setting (behaviour)	Feedback on behaviour	self monitoring of behaviour	social support (practical	Credible source			
0	0	Goal setting (behaviour)	Feedback on behaviour	social support	social support (practical	Credible source			
0	0	Goal setting (behaviour)	Feedback on behaviour	social support	Information about health consequences	Demonstration of the behaviour			
0	0	Goal setting (behaviour)	Feedback on behaviour	Information about health consequences	Prompts/cues	Adding object to the environment			
1	1	Problem solving	Action planning	Review of behaviour goals	Feedback on behaviour	self monitoring of behaviour	Information about health consequences	Prompts/cues	Graded tasks
1	1	Problem solving	Action planning	Review of behaviour goals	Feedback on behaviour	self monitoring of behaviour	Information about health consequences	Prompts/cues	Credible source
2	2	Problem solving	Action planning	Review of behaviour goals	Feedback on behaviour	self monitoring of behaviour	Demonstration of the behaviour	Behavioural practice/rehearsal	Credible source
0	0	Goal setting (behaviour)	Feedback on behaviour	social support	Information about health consequences	Prompts/cues	Credible source		
0	0	Goal setting (behaviour)	self monitoring of behaviour	social support	Information about health consequences	Prompts/cues	Credible source		
0	0	Feedback on behaviour	self monitoring of behaviour	social support	Information about health consequences	Prompts/cues	Credible source		
0	0	Goal setting (behaviour)	Feedback on behaviour	self monitoring of behaviour	social support	Information about health consequences	Prompts/cues	Credible source	
2	2	Goal setting (behaviour)	Action planning	Feedback on behaviour	self monitoring of behaviour	Instruction on how to perform the behaviour	Information about health consequences	Behavioural practice/rehearsal	Reduce negative emotions
2	2	Goal setting (behaviour)	Action planning	Feedback on behaviour	self monitoring of behaviour	Instruction on how to perform the behaviour	Information about health consequences	Graded tasks	Reduce negative emotions
1	1	Goal setting (behaviour)	Action planning	Feedback on behaviour	self monitoring of behaviour	Instruction on how to perform the behaviour	Demonstration of the behaviour	Behavioural practice/rehearsal	Graded tasks
1	1	Goal setting (behaviour)	Action planning	Feedback on behaviour	self monitoring of behaviour	Instruction on how to perform the behaviour	Demonstration of the behaviour	Behavioural practice/rehearsal	Credible source
1	1	Problem solving	Action planning	Review of behaviour goals	Discrepancy between current behaviour and goal	self monitoring of behaviour	Information about health consequences	Prompts/cues	Credible source
1	1	Problem solving	Action planning	Review of behaviour goals	Discrepancy between current behaviour and goal	Instruction on how to perform the behaviour	Information about health consequences	Prompts/cues	Credible source
1	1	Problem solving	Action planning	Review of behaviour goals	Feedback on behaviour	self monitoring of behaviour	Instruction on how to perform the behaviour	Information about health consequences	Credible source
2	2	Problem solving	Action planning	Review of behaviour goals	Feedback on behaviour	self monitoring of behaviour	Instruction on how to perform the behaviour	Demonstration of the behaviour	Credible source
1	1	Problem solving	Action planning	Review of behaviour goals	Feedback on behaviour	self monitoring of behaviour	Instruction on how to perform the behaviour	Prompts/cues	Graded tasks
1	1	Problem solving	Action planning	Review of behaviour goals	Feedback on behaviour	self monitoring of behaviour	Instruction on how to perform the behaviour	Prompts/cues	Credible source
2	2	Problem solving	Action planning	Review of behaviour goals	Feedback on behaviour	self monitoring of behaviour	Instruction on how to perform the behaviour	Behavioural practice/rehearsal	Credible source
0	0	Goal setting (behaviour)	Feedback on behaviour	self monitoring of behaviour	Information about health consequences	Prompts/cues	Adding object to the environment		
1	1	Problem solving	Action planning	Review of behaviour goals	Discrepancy between current behaviour and goal	Feedback on behaviour	self monitoring of behaviour	Prompts/cues	Credible source
1	1	Problem solving	Action planning	Review of behaviour goals	Discrepancy between current behaviour and goal	Feedback on behaviour	Instruction on how to perform the behaviour	Information about health consequences	Prompts/cues
1	1	Problem solving	Action planning	Review of behaviour goals	Discrepancy between current behaviour and goal	Feedback on behaviour	Instruction on how to perform the behaviour	Information about health consequences	Credible source
1	1	Problem solving	Action planning	Review of behaviour goals	Discrepancy between current behaviour and goal	Feedback on behaviour	Instruction on how to perform the behaviour	Prompts/cues	Credible source
1	1	Problem solving	Action planning	Review of behaviour goals	Discrepancy between current behaviour and goal	Feedback on behaviour	Information about health consequences	Prompts/cues	Credible source
1	1	Problem solving	Action planning	Review of behaviour goals	Discrepancy between current behaviour and goal	self monitoring of behaviour	Instruction on how to perform the behaviour	Information about health consequences	Prompts/cues
1	1	Problem solving	Action planning	Review of behaviour goals	Discrepancy between current behaviour and goal	self monitoring of behaviour	Instruction on how to perform the behaviour	Information about health consequences	Credible source
1	1	Problem solving	Action planning	Review of behaviour goals	Discrepancy between current behaviour and goal	self monitoring of behaviour	Instruction on how to perform the behaviour	Prompts/cues	Credible source
0	0	self monitoring of behaviour	social support	Information about health consequences	Prompts/cues	Credible source			
0	0	Goal setting (behaviour)	Feedback on behaviour	self monitoring of behaviour	social support	social support (practical	Credible source		
2	2	Problem solving	Goal setting (outcome)	Action planning	self monitoring of behaviour	Instruction on how to perform the behaviour	Demonstration of the behaviour	Behavioural practice/rehearsal	Credible source
1	1	Problem solving	Action planning	Review of behaviour goals	Discrepancy between current behaviour and goal	Feedback on behaviour	self monitoring of behaviour	Instruction on how to perform the behaviour	Information about health consequences

1	1	Review of behaviour goals	Discrepancy between current behaviour and goal	Feedback on behaviour	self monitoring of behaviour	Instruction on how to perform the behaviour	Prompts/cues	Credible source	
1	1	Feedback on behaviour	social support	Demonstration of the behaviour	Social reward				
1	1	self monitoring of behaviour	social support	Demonstration of the behaviour	Social reward				
0	0	self monitoring of behaviour	social support	Social reward	Verbal persuasion about capability				
0	0	self monitoring of behaviour	Prompts/cues	Social reward	Verbal persuasion about capability				
1	1	social support	Demonstration of the behaviour	Prompts/cues	Social reward				
0	0	social support	Prompts/cues	Social reward	Verbal persuasion about capability				
1	1	Goal setting (behaviour)	Problem solving	Action planning	Feedback on behaviour	Social reward			
0	0	Goal setting (behaviour)	Problem solving	Action planning	Feedback on behaviour	Verbal persuasion about capability			
1	1	Action planning	Feedback on behaviour	Demonstration of the behaviour	Social reward				
0	0	Action planning	Feedback on behaviour	Prompts/cues	Verbal persuasion about capability				
1	1	Action planning	self monitoring of behaviour	social support	Social support (emotional)				
1	1	Action planning	self monitoring of behaviour	Demonstration of the behaviour	Social reward				
0	0	Action planning	self monitoring of behaviour	Social reward	Verbal persuasion about capability				
0	0	Action planning	social support	Social reward	Verbal persuasion about capability				
1	1	Action planning	Demonstration of the behaviour	Prompts/cues	Social reward				
0	0	Action planning	Prompts/cues	Social reward	Verbal persuasion about capability				
2	2	Action planning	Discrepancy between current behaviour and goal	Feedback on behaviour	self monitoring of behaviour	Instruction on how to perform the behaviour	Graded tasks	Credible source	
1	1	Action planning	Feedback on behaviour	self monitoring of behaviour	Instruction on how to perform the behaviour	Information about health consequences	Prompts/cues	Graded tasks	
2	2	Action planning	Feedback on behaviour	self monitoring of behaviour	Instruction on how to perform the behaviour	Information about health consequences	Behavioural practice/rehearsal	Graded tasks	
2	2	Action planning	Feedback on behaviour	self monitoring of behaviour	Instruction on how to perform the behaviour	Information about health consequences	Behavioural practice/rehearsal	Reduce negative emotions	
2	2	Action planning	Feedback on behaviour	self monitoring of behaviour	Instruction on how to perform the behaviour	Information about health consequences	Graded tasks	Reduce negative emotions	
2	2	Action planning	Feedback on behaviour	self monitoring of behaviour	Instruction on how to perform the behaviour	Prompts/cues	Graded tasks	Credible source	
2	2	Action planning	Feedback on behaviour	self monitoring of behaviour	Instruction on how to perform the behaviour	Behavioural practice/rehearsal	Graded tasks	Credible source	
2	2	Action planning	Feedback on behaviour	self monitoring of behaviour	Instruction on how to perform the behaviour	Behavioural practice/rehearsal	Graded tasks	Reduce negative emotions	
2	2	Action planning	Review of behaviour goals	self monitoring of behaviour	Instruction on how to perform the behaviour	Demonstration of the behaviour	Graded tasks	Credible source	
2	2	Action planning	Review of behaviour goals	self monitoring of behaviour	Instruction on how to perform the behaviour	Behavioural practice/rehearsal	Graded tasks	Credible source	
2	2	Action planning	Review of behaviour goals	self monitoring of behaviour	Instruction on how to perform the behaviour	Behavioural practice/rehearsal	Graded tasks	Reduce negative emotions	
2	2	Action planning	Review of behaviour goals	self monitoring of behaviour	Information about health consequences	Behavioural practice/rehearsal	Graded tasks	Reduce negative emotions	
2	2	Action planning	Review of behaviour goals	self monitoring of behaviour	Demonstration of the behaviour	Behavioural practice/rehearsal	Graded tasks	Credible source	
2	2	Action planning	Review of behaviour goals	Instruction on how to perform the behaviour	Information about health consequences	Behavioural practice/rehearsal	Graded tasks	Reduce negative emotions	
2	2	Action planning	Review of behaviour goals	Instruction on how to perform the behaviour	Demonstration of the behaviour	Behavioural practice/rehearsal	Graded tasks	Credible source	
2	2	Action planning	Discrepancy between current behaviour and goal	Feedback on behaviour	self monitoring of behaviour	Instruction on how to perform the behaviour	Demonstration of the behaviour	Credible source	
2	2	Review of behaviour goals	Feedback on behaviour	Instruction on how to perform the behaviour	Demonstration of the behaviour	Behavioural practice/rehearsal	Graded tasks	Credible source	
2	2	Review of behaviour goals	self monitoring of behaviour	Instruction on how to perform the behaviour	Information about health consequences	Behavioural practice/rehearsal	Graded tasks	Reduce negative emotions	
2	2	Review of behaviour goals	self monitoring of behaviour	Instruction on how to perform the behaviour	Demonstration of the behaviour	Behavioural practice/rehearsal	Graded tasks	Credible source	
2	2	Feedback on behaviour	self monitoring of behaviour	Instruction on how to perform the behaviour	Information about health consequences	Behavioural practice/rehearsal	Graded tasks	Reduce negative emotions	
3	3	Goal setting (behaviour)	Problem solving	Goal setting (outcome)	Action planning	Feedback on behaviour	self monitoring of behaviour	Instruction on how to perform the behaviour	Demonstration of the behaviour
3	3	Goal setting (behaviour)	Problem solving	Goal setting (outcome)	Action planning	Feedback on behaviour	self monitoring of behaviour	Instruction on how to perform the behaviour	Graded tasks
2	2	Action planning	Review of behaviour goals	self monitoring of behaviour	Instruction on how to perform the behaviour	Information about health consequences	Graded tasks	Reduce negative emotions	
2	2	Action planning	Review of behaviour goals	self monitoring of behaviour	Instruction on how to perform the behaviour	Demonstration of the behaviour	Behavioural practice/rehearsal	Credible source	
2	2	Review of behaviour goals	Feedback on behaviour	self monitoring of behaviour	Instruction on how to perform the behaviour	Information about health consequences	Graded tasks	Reduce negative emotions	
2	2	Review of behaviour goals	Feedback on behaviour	self monitoring of behaviour	Instruction on how to perform the behaviour	Demonstration of the behaviour	Behavioural practice/rehearsal	Credible source	
2	2	Review of behaviour goals	Feedback on behaviour	self monitoring of behaviour	Instruction on how to perform the behaviour	Demonstration of the behaviour	Graded tasks	Credible source	
2	2	Review of behaviour goals	Feedback on behaviour	self monitoring of behaviour	Instruction on how to perform the behaviour	Behavioural practice/rehearsal	Graded tasks	Credible source	
2	2	Review of behaviour goals	Feedback on behaviour	self monitoring of behaviour	Instruction on how to perform the behaviour	Behavioural practice/rehearsal	Graded tasks	Reduce negative emotions	
2	2	Review of behaviour goals	Feedback on behaviour	self monitoring of behaviour	Information about health consequences	Behavioural practice/rehearsal	Graded tasks	Reduce negative emotions	
2	2	Review of behaviour goals	Feedback on behaviour	self monitoring of behaviour	Demonstration of the behaviour	Behavioural practice/rehearsal	Graded tasks	Credible source	
2	2	Review of behaviour goals	Feedback on behaviour	Instruction on how to perform the behaviour	Information about health consequences	Behavioural practice/rehearsal	Graded tasks	Reduce negative emotions	
2	2	Action planning	Review of behaviour goals	Feedback on behaviour	self monitoring of behaviour	Instruction on how to perform the behaviour	Behavioural practice/rehearsal	Credible source	
2	2	Action planning	Review of behaviour goals	Feedback on behaviour	self monitoring of behaviour	Instruction on how to perform the behaviour	Behavioural practice/rehearsal	Reduce negative emotions	
2	2	Action planning	Review of behaviour goals	Feedback on behaviour	self monitoring of behaviour	Instruction on how to perform the behaviour	Graded tasks	Reduce negative emotions	
1	1	Action planning	Review of behaviour goals	Feedback on behaviour	self monitoring of behaviour	Information about health consequences	Prompts/cues	Graded tasks	
1	1	Action planning	Review of behaviour goals	Feedback on behaviour	self monitoring of behaviour	Information about health consequences	Prompts/cues	Credible source	
2	2	Action planning	Review of behaviour goals	Feedback on behaviour	self monitoring of behaviour	Information about health consequences	Behavioural practice/rehearsal	Graded tasks	
2	2	Action planning	Review of behaviour goals	Feedback on behaviour	self monitoring of behaviour	Information about health consequences	Behavioural practice/rehearsal	Reduce negative emotions	
2	2	Action planning	Review of behaviour goals	Feedback on behaviour	self monitoring of behaviour	Information about health consequences	Graded tasks	Reduce negative emotions	
1	1	Goal setting (behaviour)	Action planning	self monitoring of behaviour	Demonstration of the behaviour	Social reward			
0	0	Goal setting (behaviour)	Action planning	self monitoring of behaviour	Social reward	Verbal persuasion about capability			
0	0	Goal setting (behaviour)	Action planning	social support	Social reward	Verbal persuasion about capability			
1	1	Goal setting (behaviour)	Action planning	Demonstration of the behaviour	Prompts/cues	Social reward			
0	0	Goal setting (behaviour)	Action planning	Prompts/cues	Social reward	Verbal persuasion about capability			
1	1	Goal setting (behaviour)	Feedback on behaviour	social support	Demonstration of the behaviour	Social reward			
1	1	Goal setting (behaviour)	self monitoring of behaviour	social support	Demonstration of the behaviour	Social reward			
1	1	Action planning	Review of behaviour goals	Feedback on behaviour	self monitoring of behaviour	Instruction on how to perform the behaviour	Prompts/cues	Credible source	

2	2	Goal setting (behaviour)	Feedback on behaviour	self monitoring of behaviour	Instruction on how to perform the behaviour	Information about health consequences	Graded tasks	Reduce negative emotions
2	2	Goal setting (behaviour)	Feedback on behaviour	self monitoring of behaviour	Instruction on how to perform the behaviour	Prompts/cues	Graded tasks	Credible source
2	2	Goal setting (behaviour)	Feedback on behaviour	self monitoring of behaviour	Instruction on how to perform the behaviour	Behavioural practice/rehearsal	Graded tasks	Reduce negative emotions
2	2	Goal setting (behaviour)	Feedback on behaviour	self monitoring of behaviour	Information about health consequences	Behavioural practice/rehearsal	Graded tasks	Reduce negative emotions
1	1	Problem solving	Action planning	Review of behaviour goals	Discrepancy between current behaviour and goal	self monitoring of behaviour	Instruction on how to perform the behaviour	Prompts/cues
1	1	Problem solving	Action planning	Review of behaviour goals	Discrepancy between current behaviour and goal	self monitoring of behaviour	Instruction on how to perform the behaviour	Credible source
1	1	Problem solving	Action planning	Review of behaviour goals	Discrepancy between current behaviour and goal	self monitoring of behaviour	Information about health consequences	Prompts/cues
1	1	Problem solving	Action planning	Review of behaviour goals	Discrepancy between current behaviour and goal	self monitoring of behaviour	Information about health consequences	Credible source
1	1	Problem solving	Action planning	Review of behaviour goals	Discrepancy between current behaviour and goal	self monitoring of behaviour	Prompts/cues	Credible source
1	1	Problem solving	Action planning	Review of behaviour goals	Discrepancy between current behaviour and goal	Instruction on how to perform the behaviour	Information about health consequences	Prompts/cues
1	1	Problem solving	Action planning	Review of behaviour goals	Discrepancy between current behaviour and goal	Instruction on how to perform the behaviour	Information about health consequences	Credible source
2	2	Goal setting (behaviour)	Discrepancy between current behaviour and goal	Feedback on behaviour	self monitoring of behaviour	Instruction on how to perform the behaviour	Graded tasks	Credible source
1	1	Problem solving	Action planning	Review of behaviour goals	Discrepancy between current behaviour and goal	Feedback on behaviour	self monitoring of behaviour	Credible source
1	1	Problem solving	Action planning	Review of behaviour goals	Discrepancy between current behaviour and goal	Feedback on behaviour	Instruction on how to perform the behaviour	Information about health consequences
1	1	Problem solving	Action planning	Review of behaviour goals	Discrepancy between current behaviour and goal	Feedback on behaviour	Instruction on how to perform the behaviour	Prompts/cues
1	1	Problem solving	Action planning	Review of behaviour goals	Discrepancy between current behaviour and goal	Feedback on behaviour	Instruction on how to perform the behaviour	Credible source
1	1	Problem solving	Action planning	Review of behaviour goals	Discrepancy between current behaviour and goal	Feedback on behaviour	Information about health consequences	Prompts/cues
1	1	Problem solving	Action planning	Review of behaviour goals	Discrepancy between current behaviour and goal	Feedback on behaviour	Information about health consequences	Credible source
1	1	Problem solving	Action planning	Review of behaviour goals	Discrepancy between current behaviour and goal	Feedback on behaviour	Prompts/cues	Credible source
1	1	Problem solving	Action planning	Review of behaviour goals	Discrepancy between current behaviour and goal	self monitoring of behaviour	Instruction on how to perform the behaviour	Information about health consequences
2	2	Goal setting (behaviour)	Discrepancy between current behaviour and goal	Feedback on behaviour	self monitoring of behaviour	Instruction on how to perform the behaviour	Demonstration of the behaviour	Graded tasks
2	2	Goal setting (behaviour)	Discrepancy between current behaviour and goal	Feedback on behaviour	self monitoring of behaviour	Instruction on how to perform the behaviour	Demonstration of the behaviour	Credible source
1	1	Goal setting (behaviour)	Discrepancy between current behaviour and goal	Feedback on behaviour	self monitoring of behaviour	Instruction on how to perform the behaviour	Prompts/cues	Graded tasks
1	1	Action planning	social support	Instruction on how to perform the behaviour	Demonstration of the behaviour	Prompts/cues		
1	1	Feedback on behaviour	social support	Demonstration of the behaviour	Prompts/cues	Graded tasks		
1	1	self monitoring of behaviour	social support	Demonstration of the behaviour	Prompts/cues	Graded tasks		
1	1	social support	Instruction on how to perform the behaviour	Demonstration of the behaviour	Prompts/cues	Graded tasks		
2	2	Goal setting (behaviour)	Action planning	self monitoring of behaviour	Information about health consequences	Behavioural practice/rehearsal	Graded tasks	Reduce negative emotions
1	1	Goal setting (behaviour)	Review of behaviour goals	self monitoring of behaviour	Instruction on how to perform the behaviour	Information about health consequences	Prompts/cues	Credible source
2	2	Goal setting (behaviour)	Review of behaviour goals	self monitoring of behaviour	Instruction on how to perform the behaviour	Information about health consequences	Behavioural practice/rehearsal	Graded tasks
2	2	Goal setting (behaviour)	Review of behaviour goals	self monitoring of behaviour	Instruction on how to perform the behaviour	Information about health consequences	Behavioural practice/rehearsal	Reduce negative emotions
2	2	Goal setting (behaviour)	Review of behaviour goals	self monitoring of behaviour	Instruction on how to perform the behaviour	Information about health consequences	Graded tasks	Reduce negative emotions
1	1	Goal setting (behaviour)	Review of behaviour goals	self monitoring of behaviour	Instruction on how to perform the behaviour	Demonstration of the behaviour	Behavioural practice/rehearsal	Graded tasks
2	2	Goal setting (behaviour)	Review of behaviour goals	self monitoring of behaviour	Instruction on how to perform the behaviour	Behavioural practice/rehearsal	Graded tasks	Reduce negative emotions
2	2	Goal setting (behaviour)	Review of behaviour goals	self monitoring of behaviour	Information about health consequences	Behavioural practice/rehearsal	Graded tasks	Reduce negative emotions
2	2	Goal setting (behaviour)	Review of behaviour goals	Instruction on how to perform the behaviour	Information about health consequences	Behavioural practice/rehearsal	Graded tasks	Reduce negative emotions
1	1	Goal setting (behaviour)	Review of behaviour goals	Feedback on behaviour	Instruction on how to perform the behaviour	Information about health consequences	Prompts/cues	Credible source
2	2	Goal setting (behaviour)	Review of behaviour goals	Feedback on behaviour	Instruction on how to perform the behaviour	Information about health consequences	Behavioural practice/rehearsal	Graded tasks
2	2	Goal setting (behaviour)	Review of behaviour goals	Feedback on behaviour	Instruction on how to perform the behaviour	Information about health consequences	Behavioural practice/rehearsal	Reduce negative emotions
2	2	Goal setting (behaviour)	Review of behaviour goals	Feedback on behaviour	Instruction on how to perform the behaviour	Information about health consequences	Graded tasks	Reduce negative emotions
1	1	Goal setting (behaviour)	Review of behaviour goals	Feedback on behaviour	Instruction on how to perform the behaviour	Demonstration of the behaviour	Behavioural practice/rehearsal	Graded tasks
2	2	Goal setting (behaviour)	Review of behaviour goals	Feedback on behaviour	Instruction on how to perform the behaviour	Behavioural practice/rehearsal	Graded tasks	Reduce negative emotions
2	2	Goal setting (behaviour)	Review of behaviour goals	Feedback on behaviour	Information about health consequences	Behavioural practice/rehearsal	Graded tasks	Reduce negative emotions
1	1	Goal setting (behaviour)	Review of behaviour goals	self monitoring of behaviour	Instruction on how to perform the behaviour	Information about health consequences	Prompts/cues	Graded tasks
2	2	Goal setting (behaviour)	Review of behaviour goals	Feedback on behaviour	self monitoring of behaviour	Instruction on how to perform the behaviour	Graded tasks	Reduce negative emotions
1	1	Goal setting (behaviour)	Review of behaviour goals	Feedback on behaviour	self monitoring of behaviour	Information about health consequences	Prompts/cues	Credible source
2	2	Goal setting (behaviour)	Review of behaviour goals	Feedback on behaviour	self monitoring of behaviour	Information about health consequences	Behavioural practice/rehearsal	Graded tasks
2	2	Goal setting (behaviour)	Review of behaviour goals	Feedback on behaviour	self monitoring of behaviour	Information about health consequences	Behavioural practice/rehearsal	Reduce negative emotions
2	2	Goal setting (behaviour)	Review of behaviour goals	Feedback on behaviour	self monitoring of behaviour	Information about health consequences	Graded tasks	Reduce negative emotions
1	1	Goal setting (behaviour)	Review of behaviour goals	Feedback on behaviour	self monitoring of behaviour	Demonstration of the behaviour	Behavioural practice/rehearsal	Graded tasks
2	2	Goal setting (behaviour)	Review of behaviour goals	Feedback on behaviour	self monitoring of behaviour	Behavioural practice/rehearsal	Graded tasks	Reduce negative emotions
1	1	Goal setting (behaviour)	Review of behaviour goals	Feedback on behaviour	Instruction on how to perform the behaviour	Information about health consequences	Prompts/cues	Graded tasks
1	1	Goal setting (behaviour)	Review of behaviour goals	Feedback on behaviour	self monitoring of behaviour	Instruction on how to perform the behaviour	Prompts/cues	Graded tasks
1	1	Goal setting (behaviour)	Review of behaviour goals	Feedback on behaviour	self monitoring of behaviour	Instruction on how to perform the behaviour	Prompts/cues	Credible source
1	1	Goal setting (behaviour)	Problem solving	social support	Instruction on how to perform the behaviour	Demonstration of the behaviour	Prompts/cues	
1	1	Goal setting (behaviour)	Problem solving	social support	Demonstration of the behaviour	Prompts/cues	Graded tasks	
1	1	Goal setting (behaviour)	Action planning	social support	Instruction on how to perform the behaviour	Demonstration of the behaviour	Prompts/cues	
1	1	Goal setting (behaviour)	Action planning	Feedback on behaviour	self monitoring of behaviour	Demonstration of the behaviour	Behavioural practice/rehearsal	Graded tasks
1	1	Goal setting (behaviour)	Action planning	Feedback on behaviour	self monitoring of behaviour	Demonstration of the behaviour	Behavioural practice/rehearsal	Credible source
2	2	Goal setting (behaviour)	Action planning	Feedback on behaviour	self monitoring of behaviour	Prompts/cues	Graded tasks	Credible source

1	1	Goal setting (behaviour)	Problem solving	Action planning	Feedback on behaviour	Demonstration of the behaviour	Behavioural practice/rehearsal	Graded tasks
2	2	Goal setting (behaviour)	Problem solving	Action planning	Feedback on behaviour	Prompts/cues	Graded tasks	Credible source
1	1	Goal setting (behaviour)	Problem solving	Action planning	self monitoring of behaviour	Social support (emotional)	Demonstration of the behaviour	Prompts/cues
1	1	Goal setting (behaviour)	Problem solving	Action planning	self monitoring of behaviour	Social support (emotional)	Demonstration of the behaviour	Behavioural practice/rehearsal
1	1	Goal setting (behaviour)	Problem solving	Action planning	self monitoring of behaviour	Social support (emotional)	Prompts/cues	Behavioural practice/rehearsal
2	2	Goal setting (behaviour)	Problem solving	Action planning	Discrepancy between current behaviour and goal	Feedback on behaviour	Instruction on how to perform the behaviour	Graded tasks
2	2	Goal setting (behaviour)	Problem solving	Action planning	Discrepancy between current behaviour and goal	Feedback on behaviour	Graded tasks	Credible source
0	0	social support	Instruction on how to perform the behaviour	Demonstration of the behaviour	Behavioural practice/rehearsal	Graded tasks	Credible source	
1	1	Goal setting (behaviour)	Problem solving	Action planning	Feedback on behaviour	self monitoring of behaviour	Behavioural practice/rehearsal	Graded tasks
1	1	Goal setting (behaviour)	Problem solving	Action planning	Feedback on behaviour	self monitoring of behaviour	Behavioural practice/rehearsal	Credible source
2	2	Goal setting (behaviour)	Problem solving	Action planning	Feedback on behaviour	Instruction on how to perform the behaviour	Demonstration of the behaviour	Prompts/cues
1	1	Goal setting (behaviour)	Problem solving	Action planning	Feedback on behaviour	Instruction on how to perform the behaviour	Demonstration of the behaviour	Behavioural practice/rehearsal
1	1	Goal setting (behaviour)	Problem solving	Action planning	Feedback on behaviour	Instruction on how to perform the behaviour	Behavioural practice/rehearsal	Graded tasks
1	1	Goal setting (behaviour)	Problem solving	Action planning	Feedback on behaviour	Instruction on how to perform the behaviour	Behavioural practice/rehearsal	Credible source
1	1	Goal setting (behaviour)	Problem solving	Action planning	Feedback on behaviour	Information about health consequences	Prompts/cues	Graded tasks
0	0	Problem solving	self monitoring of behaviour	social support	Instruction on how to perform the behaviour	Behavioural practice/rehearsal	Graded tasks	
0	0	Problem solving	self monitoring of behaviour	social support	Demonstration of the behaviour	Behavioural practice/rehearsal	Graded tasks	
0	0	Problem solving	social support	Instruction on how to perform the behaviour	Demonstration of the behaviour	Behavioural practice/rehearsal	Graded tasks	
0	0	Feedback on behaviour	self monitoring of behaviour	social support	Behavioural practice/rehearsal	Graded tasks	Credible source	
0	0	Feedback on behaviour	social support	Instruction on how to perform the behaviour	Behavioural practice/rehearsal	Graded tasks	Credible source	
0	0	Feedback on behaviour	social support	Demonstration of the behaviour	Behavioural practice/rehearsal	Graded tasks	Credible source	
0	0	self monitoring of behaviour	social support	Instruction on how to perform the behaviour	Behavioural practice/rehearsal	Graded tasks	Credible source	
0	0	self monitoring of behaviour	social support	Demonstration of the behaviour	Behavioural practice/rehearsal	Graded tasks	Credible source	
1	1	Goal setting (behaviour)	Problem solving	Action planning	Review of behaviour goals	Information about health consequences	Prompts/cues	Credible source
1	1	Goal setting (behaviour)	Problem solving	Action planning	Review of behaviour goals	Demonstration of the behaviour	Behavioural practice/rehearsal	Graded tasks
2	2	Goal setting (behaviour)	Problem solving	Action planning	Discrepancy between current behaviour and goal	Feedback on behaviour	self monitoring of behaviour	Graded tasks
0	0	Goal setting (behaviour)	self monitoring of behaviour	social support	Instruction on how to perform the behaviour	Behavioural practice/rehearsal	Graded tasks	Credible source
0	0	Goal setting (behaviour)	self monitoring of behaviour	social support	Demonstration of the behaviour	Behavioural practice/rehearsal	Graded tasks	Credible source
0	0	Goal setting (behaviour)	social support	Instruction on how to perform the behaviour	Demonstration of the behaviour	Behavioural practice/rehearsal	Graded tasks	Credible source
0	0	Problem solving	Feedback on behaviour	self monitoring of behaviour	social support	Instruction on how to perform the behaviour	Demonstration of the behaviour	Behavioural practice/rehearsal
0	0	Problem solving	Feedback on behaviour	self monitoring of behaviour	social support	Instruction on how to perform the behaviour	Behavioural practice/rehearsal	Graded tasks
1	1	Goal setting (behaviour)	Problem solving	Action planning	Review of behaviour goals	Instruction on how to perform the behaviour	Information about health consequences	Credible source
1	1	Goal setting (behaviour)	Problem solving	Action planning	Review of behaviour goals	Instruction on how to perform the behaviour	Demonstration of the behaviour	Behavioural practice/rehearsal
1	1	Goal setting (behaviour)	Problem solving	Action planning	Review of behaviour goals	Instruction on how to perform the behaviour	Demonstration of the behaviour	Graded tasks
1	1	Goal setting (behaviour)	Problem solving	Action planning	Review of behaviour goals	Instruction on how to perform the behaviour	Prompts/cues	Graded tasks
1	1	Goal setting (behaviour)	Problem solving	Action planning	Review of behaviour goals	Instruction on how to perform the behaviour	Prompts/cues	Credible source
1	1	Goal setting (behaviour)	Problem solving	Action planning	Review of behaviour goals	Instruction on how to perform the behaviour	Behavioural practice/rehearsal	Graded tasks
1	1	Goal setting (behaviour)	Problem solving	Action planning	Review of behaviour goals	Instruction on how to perform the behaviour	Graded tasks	Credible source
1	1	Goal setting (behaviour)	Problem solving	Action planning	Review of behaviour goals	Information about health consequences	Prompts/cues	Graded tasks
1	1	Goal setting (behaviour)	Problem solving	Action planning	Review of behaviour goals	Feedback on behaviour	Graded tasks	Credible source
1	1	Goal setting (behaviour)	Problem solving	Action planning	Review of behaviour goals	self monitoring of behaviour	Instruction on how to perform the behaviour	Demonstration of the behaviour
1	1	Goal setting (behaviour)	Problem solving	Action planning	Review of behaviour goals	self monitoring of behaviour	Instruction on how to perform the behaviour	Behavioural practice/rehearsal
1	1	Goal setting (behaviour)	Problem solving	Action planning	Review of behaviour goals	self monitoring of behaviour	Information about health consequences	Credible source
1	1	Goal setting (behaviour)	Problem solving	Action planning	Review of behaviour goals	self monitoring of behaviour	Demonstration of the behaviour	Graded tasks
1	1	Goal setting (behaviour)	Problem solving	Action planning	Review of behaviour goals	self monitoring of behaviour	Prompts/cues	Credible source
1	1	Goal setting (behaviour)	Problem solving	Action planning	Review of behaviour goals	self monitoring of behaviour	Behavioural practice/rehearsal	Graded tasks
1	1	Goal setting (behaviour)	Problem solving	Action planning	Review of behaviour goals	self monitoring of behaviour	Graded tasks	Credible source
0	0	Goal setting (behaviour)	Problem solving	Feedback on behaviour	social support	Instruction on how to perform the behaviour	Behavioural practice/rehearsal	Credible source
0	0	Goal setting (behaviour)	Problem solving	Feedback on behaviour	social support	Demonstration of the behaviour	Behavioural practice/rehearsal	Graded tasks
0	0	Goal setting (behaviour)	Problem solving	self monitoring of behaviour	social support	Instruction on how to perform the behaviour	Behavioural practice/rehearsal	Graded tasks
0	0	Goal setting (behaviour)	Problem solving	self monitoring of behaviour	social support	Demonstration of the behaviour	Behavioural practice/rehearsal	Graded tasks
0	0	Goal setting (behaviour)	Problem solving	social support	Instruction on how to perform the behaviour	Demonstration of the behaviour	Behavioural practice/rehearsal	Graded tasks
0	0	Goal setting (behaviour)	Feedback on behaviour	self monitoring of behaviour	social support	Behavioural practice/rehearsal	Graded tasks	Credible source
0	0	Goal setting (behaviour)	Feedback on behaviour	social support	Instruction on how to perform the behaviour	Behavioural practice/rehearsal	Graded tasks	Credible source
0	0	Goal setting (behaviour)	Feedback on behaviour	social support	Demonstration of the behaviour	Behavioural practice/rehearsal	Graded tasks	Credible source
2	2	Goal setting (behaviour)	Graded tasks	Self-talk				
2	2	Action planning	self monitoring of behaviour	Self-talk				
2	2	Action planning	social support	Self-talk				
2	2	Action planning	Graded tasks	Self-talk				
2	2	self monitoring of behaviour	social support	Self-talk				

3	1	0	1	0	0	0	0	1	1	0	0	0	0	0	1	1	0	0	0	0	0	3	2	1	2	1	1	1	Action planning	Review of behaviour goals	Discrepancy between current behaviour and goal	Instruction on how to perform the behaviour	Information about health consequences	Prompts/cues	50	0	100	0	0	0	0	6
3	1	0	1	0	0	0	0	1	1	0	0	0	0	0	1	1	0	0	0	0	0	3	2	1	2	1	1	1	Action planning	[illegible]	[illegible]	[illegible]	[illegible]	[illegible]	50	0	100	0	0	0	0	6
3	1	0	1	0	0	0	0	1	1	0	0	0	0	0	1	1	0	0	0	0	0	3	2	1	2	1	1	1	Action planning	[illegible]	[illegible]	[illegible]	[illegible]	[illegible]	50	0	100	0	0	0	0	6
3	1	0	1	0	0	0	0	1	1	0	0	0	0	0	1	1	0	0	0	0	0	3	2	1	2	1	1	1	Action planning	[illegible]	[illegible]	[illegible]	[illegible]	[illegible]	50	0	100	0	0	0	0	6
3	1	0	1	0	0	0	0	1	1	0	0	0	0	0	1	1	0	0	0	0	0	3	2	1	2	1	1	1	Action planning	[illegible]	[illegible]	[illegible]	[illegible]	[illegible]	50	0	100	0	0	0	0	6
3	1	0	1	0	0	0	0	1	1	0	0	0	0	0	1	1	0	0	0	0	0	3	2	1	2	1	1	1	Action planning	[illegible]	[illegible]	[illegible]	[illegible]	[illegible]	50	0	100	0	0	0	0	6
3	1	0	1	0	0	0	0	1	1	0	0	0	0	0	1	1	0	0	0	0	0	3	2	1	2	1	1	1	Action planning	[illegible]	[illegible]	[illegible]	[illegible]	[illegible]	50	0	100	0	0	0	0	6
3	1	0	1	0	0	0	0	1	1	0	0	0	0	0	1	1	0	0	0	0	0	3	2	1	2	1	1	1	Action planning	[illegible]	[illegible]	[illegible]	[illegible]	[illegible]	50	0	100	0	0	0	0	6
3	1	0	1	0	0	0	0	1	1	0	0	0	0	0	1	1	0	0	0	0	0	3	2	1	2	1	1	1	Action planning	[illegible]	[illegible]	[illegible]	[illegible]	[illegible]	50	0	100	0	0	0	0	6
3	1	0	1	0	0	0	0	1	1	0	0	0	0	0	1	1	0	0	0	0	0	3	2	1	2	1	1	1	Action planning	[illegible]	[illegible]	[illegible]	[illegible]	[illegible]	50	0	100	0	0	0	0	6
3	1	0	1	0	0	0	0	1	0	0	0	0	0	0	1	1	0	0	0	0	0	3	1	1	1	1	1	1	Action planning	[illegible]	[illegible]	[illegible]	[illegible]	[illegible]	50	0	100	0	0	0	0	6
3	1	0	1	0	0	0	0	1	1	0	0	0	0	0	1	1	0	0	0	0	0	3	2	1	2	1	1	1	Action planning	[illegible]	[illegible]	[illegible]	[illegible]	[illegible]	50	0	100	0	0	0	0	6
3	0	0	0	0	0	0	0	2	1	1	0	0	0	0	0	0	0	0	0	0	0	2	1	1	0	0	1	0	social support	[illegible]	[illegible]	[illegible]	[illegible]	[illegible]	0	0	0	0	0	0	0	4
3	2	0	2	0	0	0	0	0	0	0	0	0	0	0	1	2	0	0	0	0	0	3	2	2	2	2	2	2	Goal setting (outcome)	[illegible]	[illegible]	[illegible]	[illegible]	[illegible]	100	0	100	0	0	0	0	6
3	1	1	3	0	0	0	0	1	0	0	0	0	0	0	1	2	0	0	0	0	0	3	3	3	3	3	3	3	Goal setting (outcome)	[illegible]	[illegible]	[illegible]	[illegible]	[illegible]	50	100	100	0	0	0	0	6
3	2	0	2	0	0	0	0	0	0	0	0	0	0	0	1	2	0	0	0	0	0	3	2	2	2	2	2	2	Goal setting (outcome)	[illegible]	[illegible]	[illegible]	[illegible]	[illegible]	100	0	100	0	0	0	0	6
3	1	0	1	0	0	0	0	1	1	0	0	0	0	0	1	1	0	0	0	0	0	3	2	1	2	1	1	1	Action planning	[illegible]	[illegible]	[illegible]	[illegible]	[illegible]	50	0	100	0	0	0	0	6
3	1	0	1	0	0	0	0	1	1	0	0	0	0	0	1	1	0	0	0	0	0	3	2	1	2	1	1	1	Action planning	[illegible]	[illegible]	[illegible]	[illegible]	[illegible]	50	0	100	0	0	0	0	6
3	1	0	1	0	0	0	0	1	1	0	0	0	0	0	1	1	0	0	0	0	0	3	2	1	2	1	1	1	Action planning	[illegible]	[illegible]	[illegible]	[illegible]	[illegible]	50	0	100	0	0	0	0	6
3	0	0	2	0	0	0	0	1	0	0	0	0	0	0	2	2	0	0	0	0	0	3	2	2	2	2	2	2	Action planning	[illegible]	[illegible]	[illegible]	[illegible]	[illegible]	0	0	100	0	0	0	0	6
3	2	0	2	0	0	0	0	0	0	0	0	0	0	0	1	2	0	0	0	0	0	3	2	2	2	2	2	2	Action planning	[illegible]	[illegible]	[illegible]	[illegible]	[illegible]	100	0	100	0	0	0	0	6
3	1	0	1	0	0	0	0	1	0	0	0	0	0	0	1	1	0	0	0	0	0	3	1	1	1	1	1	1	Action planning	[illegible]	[illegible]	[illegible]	[illegible]	[illegible]	50	0	100	0	0	0	0	6
3	1	0	1	0	0	0	0	1	1	0	0	0	0	0	1	1	0	0	0	0	0	3	2	1	2	1	1	1	Action planning	[illegible]	[illegible]	[illegible]	[illegible]	[illegible]	50	0	100	0	0	0	0	6
3	2	0	2	0	0	0	0	0	0	0	0	0	0	0	1	2	0	0	0	0	0	3	2	2	2	2	2	2	Action planning	[illegible]	[illegible]	[illegible]	[illegible]	[illegible]	100	0	100	0	0	0	0	6
3	0	0	2	0	0	0	0	1	0	0	0	0	0	0	2	2	0	0	0	0	0	3	2	2	2	2	2	2	Action planning	[illegible]	[illegible]	[illegible]	[illegible]	[illegible]	0	0	100	0	0	0	0	6
3	0	0	2	0	0	0	0	1	0	0	0	0	0	0	2	2	0	0	0	0	0	3	2	2	2	2	2	2	Action planning	[illegible]	[illegible]	[illegible]	[illegible]	[illegible]	0	0	100	0	0	0	0	6
3	1	1	2	0	0	0	0	1	0	0	0	0	0	0	1	1	0	0	0	0	0	3	2	2	2	2	2	2	Problem solving	[illegible]	[illegible]	[illegible]	[illegible]	[illegible]	50	100	100	0	0	0	0	6
3	2	0	1	0	0	0	0	0	0	0	0	0	0	0	1	1	0	0	0	0	0	3	1	1	1	1	1	1	Problem solving	[illegible]	[illegible]	[illegible]	[illegible]	[illegible]	100	0	100	0	0	0	0	6
3	2	0	2	0	0	0	0	0	0	0	0	0	0	0	1	2	0	0	0	0	0	3	2	2	2	2	2	2	Problem solving	[illegible]	[illegible]	[illegible]	[illegible]	[illegible]	100	0	100	0	0	0	0	6
3	2	0	2	0	0	0	0	0	0	0	0	0	0	0	1	2	0	0	0	0	0	3	2	2	2	2	2	2	Problem solving	[illegible]	[illegible]	[illegible]	[illegible]	[illegible]	100	0	100	0	0	0	0	6
3	1	0	1	0	0	0	0	1	0	0	0	0	0	0	1	1	0	0	0	0	0	3	1	1	1	1	1	1	Problem solving	[illegible]	[illegible]	[illegible]	[illegible]	[illegible]	50	0	100	0	0	0	0	6
3	1	0	1	0	0	0	0	1	1	0	0	0	0	0	1	1	0	0	0	0	0	3	2	1	2	1	1	1	Problem solving	[illegible]	[illegible]	[illegible]	[illegible]	[illegible]	50	0	100	0	0	0	0	6

2	2	Goal setting (behaviour)	Action planning	Feedback on behaviour	self monitoring of behaviour	Instruction on how to perform the behaviour	Reduce negative emotions
2	2	Goal setting (behaviour)	Action planning	Feedback on behaviour	self monitoring of behaviour	Information about health consequences	Reduce negative emotions
1	1	Action planning	Review of behaviour goals	Feedback on behaviour	self monitoring of behaviour	social support	Behavioural practice/rehearsal
1	1	Action planning	Review of behaviour goals	Feedback on behaviour	social support	Instruction on how to perform the behaviour	Behavioural practice/rehearsal
1	1	Action planning	Review of behaviour goals	Feedback on behaviour	social support	Behavioural practice/rehearsal	Graded tasks
2	2	Goal setting (behaviour)	Action planning	Feedback on behaviour	Information about health consequences	Behavioural practice/rehearsal	Reduce negative emotions
2	2	Goal setting (behaviour)	Action planning	Feedback on behaviour	Information about health consequences	Graded tasks	Reduce negative emotions
2	2	Goal setting (behaviour)	Action planning	Discrepancy between current behaviour and goal	Feedback on behaviour	Graded tasks	Credible source
2	2	Goal setting (behaviour)	Action planning	Discrepancy between current behaviour and goal	self monitoring of behaviour	Instruction on how to perform the behaviour	Demonstration of the behaviour
2	2	Goal setting (behaviour)	Action planning	Discrepancy between current behaviour and goal	self monitoring of behaviour	Instruction on how to perform the behaviour	Graded tasks
1	1	Goal setting (behaviour)	Action planning	Discrepancy between current behaviour and goal	self monitoring of behaviour	Demonstration of the behaviour	Behavioural practice/rehearsal
2	2	Goal setting (behaviour)	Action planning	Discrepancy between current behaviour and goal	self monitoring of behaviour	Demonstration of the behaviour	Credible source
2	2	Goal setting (behaviour)	Action planning	Discrepancy between current behaviour and goal	self monitoring of behaviour	Graded tasks	Credible source
0	0	Problem solving	Feedback on behaviour	social support	Instruction on how to perform the behaviour	Information about health consequences	Graded tasks
2	2	Goal setting (behaviour)	Action planning	Feedback on behaviour	self monitoring of behaviour	Behavioural practice/rehearsal	Reduce negative emotions
2	2	Goal setting (behaviour)	Action planning	Feedback on behaviour	self monitoring of behaviour	Graded tasks	Reduce negative emotions
2	2	Goal setting (behaviour)	Action planning	Feedback on behaviour	Instruction on how to perform the behaviour	Information about health consequences	Reduce negative emotions
2	2	Goal setting (behaviour)	Action planning	Feedback on behaviour	Instruction on how to perform the behaviour	Behavioural practice/rehearsal	Reduce negative emotions
2	2	Goal setting (behaviour)	Action planning	Feedback on behaviour	Instruction on how to perform the behaviour	Graded tasks	Reduce negative emotions
1	1	Goal setting (behaviour)	Action planning	Feedback on behaviour	Information about health consequences	Prompts/cues	Graded tasks
2	2	Goal setting (behaviour)	Action planning	Feedback on behaviour	Information about health consequences	Behavioural practice/rehearsal	Graded tasks
2	2	Goal setting (behaviour)	Action planning	self monitoring of behaviour	Information about health consequences	Behavioural practice/rehearsal	Graded tasks
1	1	Goal setting (behaviour)	Action planning	self monitoring of behaviour	Information about health consequences	Behavioural practice/rehearsal	Credible source
2	2	Goal setting (behaviour)	Action planning	self monitoring of behaviour	Information about health consequences	Behavioural practice/rehearsal	Reduce negative emotions
0	0	Problem solving	Action planning	social support	Instruction on how to perform the behaviour	Information about health consequences	Graded tasks
0	0	Problem solving	Review of behaviour goals	Feedback on behaviour	social support	Information about health consequences	Graded tasks
0	0	Problem solving	Review of behaviour goals	self monitoring of behaviour	social support	Information about health consequences	Graded tasks
0	0	Problem solving	Review of behaviour goals	social support	Instruction on how to perform the behaviour	Information about health consequences	Graded tasks
0	0	Problem solving	Feedback on behaviour	self monitoring of behaviour	social support	Information about health consequences	Graded tasks
2	2	Goal setting (behaviour)	Action planning	Discrepancy between current behaviour and goal	Feedback on behaviour	Demonstration of the behaviour	Credible source
0	0	Action planning	Feedback on behaviour	social support	Instruction on how to perform the behaviour	Demonstration of the behaviour	Behavioural practice/rehearsal
1	1	Action planning	Feedback on behaviour	social support	Instruction on how to perform the behaviour	Behavioural practice/rehearsal	Graded tasks
1	1	Action planning	self monitoring of behaviour	social support	Instruction on how to perform the behaviour	Behavioural practice/rehearsal	Graded tasks
1	1	Review of behaviour goals	Feedback on behaviour	self monitoring of behaviour	social support	Instruction on how to perform the behaviour	Behavioural practice/rehearsal
1	1	Review of behaviour goals	Feedback on behaviour	self monitoring of behaviour	social support	Behavioural practice/rehearsal	Graded tasks
1	1	Review of behaviour goals	Feedback on behaviour	social support	Instruction on how to perform the behaviour	Behavioural practice/rehearsal	Graded tasks
1	1	Review of behaviour goals	self monitoring of behaviour	social support	Instruction on how to perform the behaviour	Behavioural practice/rehearsal	Graded tasks
2	2	Goal setting (behaviour)	Action planning	Review of behaviour goals	Information about health consequences	Behavioural practice/rehearsal	Reduce negative emotions
2	2	Goal setting (behaviour)	Action planning	Review of behaviour goals	Information about health consequences	Graded tasks	Reduce negative emotions
1	1	Goal setting (behaviour)	Action planning	Review of behaviour goals	Demonstration of the behaviour	Behavioural practice/rehearsal	Graded tasks
2	2	Goal setting (behaviour)	Action planning	Review of behaviour goals	Behavioural practice/rehearsal	Graded tasks	Reduce negative emotions
2	2	Goal setting (behaviour)	Action planning	Discrepancy between current behaviour and goal	Feedback on behaviour	self monitoring of behaviour	Demonstration of the behaviour
2	2	Goal setting (behaviour)	Action planning	Discrepancy between current behaviour and goal	Feedback on behaviour	self monitoring of behaviour	Graded tasks
2	2	Goal setting (behaviour)	Action planning	Discrepancy between current behaviour and goal	Feedback on behaviour	Instruction on how to perform the behaviour	Demonstration of the behaviour
2	2	Goal setting (behaviour)	Action planning	Discrepancy between current behaviour and goal	Feedback on behaviour	Instruction on how to perform the behaviour	Graded tasks
1	1	Goal setting (behaviour)	Action planning	Review of behaviour goals	Instruction on how to perform the behaviour	Prompts/cues	Graded tasks
1	1	Goal setting (behaviour)	Action planning	Review of behaviour goals	Instruction on how to perform the behaviour	Prompts/cues	Credible source
2	2	Goal setting (behaviour)	Action planning	Review of behaviour goals	Instruction on how to perform the behaviour	Behavioural practice/rehearsal	Reduce negative emotions
1	1	Goal setting (behaviour)	Action planning	Review of behaviour goals	Instruction on how to perform the behaviour	Graded tasks	Credible source
2	2	Goal setting (behaviour)	Action planning	Review of behaviour goals	Instruction on how to perform the behaviour	Graded tasks	Reduce negative emotions
1	1	Goal setting (behaviour)	Action planning	Review of behaviour goals	Information about health consequences	Prompts/cues	Graded tasks
1	1	Goal setting (behaviour)	Action planning	Review of behaviour goals	Information about health consequences	Prompts/cues	Credible source
2	2	Goal setting (behaviour)	Action planning	Review of behaviour goals	Information about health consequences	Behavioural practice/rehearsal	Graded tasks
1	1	Action planning	Review of behaviour goals	self monitoring of behaviour	social support	Instruction on how to perform the behaviour	Behavioural practice/rehearsal
1	1	Action planning	Review of behaviour goals	self monitoring of behaviour	social support	Behavioural practice/rehearsal	Graded tasks
1	1	Action planning	Review of behaviour goals	social support	Instruction on how to perform the behaviour	Behavioural practice/rehearsal	Graded tasks
1	1	Action planning	Feedback on behaviour	self monitoring of behaviour	social support	Behavioural practice/rehearsal	Graded tasks
2	2	Goal setting (behaviour)	Action planning	Review of behaviour goals	Instruction on how to perform the behaviour	Information about health consequences	Behavioural practice/rehearsal
1	1	Goal setting (behaviour)	Action planning	Review of behaviour goals	Instruction on how to perform the behaviour	Information about health consequences	Credible source
2	2	Goal setting (behaviour)	Action planning	Review of behaviour goals	Instruction on how to perform the behaviour	Information about health consequences	Reduce negative emotions
1	1	Goal setting (behaviour)	Action planning	Review of behaviour goals	Instruction on how to perform the behaviour	Demonstration of the behaviour	Graded tasks
1	1	Goal setting (behaviour)	Action planning	Review of behaviour goals	Discrepancy between current behaviour and goal	self monitoring of behaviour	Instruction on how to perform the behaviour
1	1	Goal setting (behaviour)	Action planning	Review of behaviour goals	Discrepancy between current behaviour and goal	self monitoring of behaviour	Information about health consequences
1	1	Goal setting (behaviour)	Action planning	Review of behaviour goals	Discrepancy between current behaviour and goal	self monitoring of behaviour	Prompts/cues
1	1	Goal setting (behaviour)	Action planning	Review of behaviour goals	Discrepancy between current behaviour and goal	self monitoring of behaviour	Credible source

1	1	Discrepancy between current behaviour and goal	Feedback on behaviour	Instruction on how to perform the behaviour	Prompts/cues	Graded tasks
1	1	Problem solving	self monitoring of behaviour	Behavioural practice/rehearsal	Framing/reframing	
1	1	Problem solving	Demonstration of the behaviour	Behavioural practice/rehearsal	Framing/reframing	
1	1	Action planning	self monitoring of behaviour	Demonstration of the behaviour	Framing/reframing	
1	1	Action planning	Demonstration of the behaviour	Behavioural practice/rehearsal	Framing/reframing	
1	1	self monitoring of behaviour	Demonstration of the behaviour	Behavioural practice/rehearsal	Framing/reframing	
1	1	Goal setting (behaviour)	Action planning	self monitoring of behaviour	Behavioural practice/rehearsal	Framing/reframing
1	1	Problem solving	Action planning	self monitoring of behaviour	Demonstration of the behaviour	Framing/reframing
2	2	Review of behaviour goals	Information about health consequences	Behavioural practice/rehearsal	Graded tasks	Reduce negative emotions
2	2	Review of behaviour goals	Demonstration of the behaviour	Behavioural practice/rehearsal	Graded tasks	Credible source
1	1	Discrepancy between current behaviour and goal	Feedback on behaviour	self monitoring of behaviour	Information about health consequences	Graded tasks
2	2	Discrepancy between current behaviour and goal	Feedback on behaviour	self monitoring of behaviour	Demonstration of the behaviour	Graded tasks
2	2	Discrepancy between current behaviour and goal	Feedback on behaviour	self monitoring of behaviour	Demonstration of the behaviour	Credible source
2	2	Discrepancy between current behaviour and goal	Feedback on behaviour	self monitoring of behaviour	Graded tasks	Credible source
2	2	Discrepancy between current behaviour and goal	Feedback on behaviour	Instruction on how to perform the behaviour	Demonstration of the behaviour	Graded tasks
2	2	Discrepancy between current behaviour and goal	Feedback on behaviour	Instruction on how to perform the behaviour	Demonstration of the behaviour	Credible source
1	1	Review of behaviour goals	Instruction on how to perform the behaviour	Information about health consequences	Prompts/cues	Graded tasks
1	1	Review of behaviour goals	Instruction on how to perform the behaviour	Information about health consequences	Prompts/cues	Credible source
2	2	Review of behaviour goals	Instruction on how to perform the behaviour	Information about health consequences	Behavioural practice/rehearsal	Graded tasks
2	2	Review of behaviour goals	Instruction on how to perform the behaviour	Information about health consequences	Behavioural practice/rehearsal	Reduce negative emotions
2	2	Review of behaviour goals	Instruction on how to perform the behaviour	Information about health consequences	Graded tasks	Reduce negative emotions
2	2	Review of behaviour goals	Instruction on how to perform the behaviour	Demonstration of the behaviour	Graded tasks	Credible source
2	2	Review of behaviour goals	Instruction on how to perform the behaviour	Behavioural practice/rehearsal	Graded tasks	Credible source
2	2	Review of behaviour goals	Instruction on how to perform the behaviour	Behavioural practice/rehearsal	Graded tasks	Reduce negative emotions
1	1	Goal setting (behaviour)	Action planning	Behavioural practice/rehearsal	Framing/reframing	
1	1	Goal setting (behaviour)	self monitoring of behaviour	Behavioural practice/rehearsal	Framing/reframing	
1	1	Problem solving	Action planning	self monitoring of behaviour	Framing/reframing	
1	1	Problem solving	Action planning	Demonstration of the behaviour	Framing/reframing	
1	1	Problem solving	Action planning	Behavioural practice/rehearsal	Framing/reframing	
1	1	Problem solving	self monitoring of behaviour	Demonstration of the behaviour	Framing/reframing	
2	2	Review of behaviour goals	self monitoring of behaviour	Behavioural practice/rehearsal	Graded tasks	Credible source
2	2	Review of behaviour goals	self monitoring of behaviour	Behavioural practice/rehearsal	Graded tasks	Reduce negative emotions
[illegible]	[illegible]	[illegible]	[illegible]	[illegible]	[illegible]	[illegible]

2	2	Problem solving	Review of behaviour goals	self monitoring of behaviour	Behavioural practice/rehearsal	Credible source			100	0	100	0	0	0	0	5
1	1	Problem solving	Review of behaviour goals	Instruction on how to perform the behaviour	Information about health consequences	Behavioural practice/rehearsal			0	100	100	0	0	0	0	5
2	2	Problem solving	Review of behaviour goals	Instruction on how to perform the behaviour	Demonstration of the behaviour	Credible source			100	0	100	0	0	0	0	5
2	2	Problem solving	Action planning	Review of behaviour goals	Behavioural practice/rehearsal	Credible source			100	0	100	0	0	0	0	5
2	2	Problem solving	Action planning	Discrepancy between current behaviour and goal	Feedback on behaviour	Graded tasks			50	100	100	0	0	0	0	5
2	2	Problem solving	Action planning	Discrepancy between current behaviour and goal	self monitoring of behaviour	Demonstration of the behaviour			100	100	100	0	0	0	0	5
2	2	Problem solving	Action planning	Discrepancy between current behaviour and goal	self monitoring of behaviour	Graded tasks			50	100	100	0	0	0	0	5
3	2	Problem solving	Action planning	Discrepancy between current behaviour and goal	Instruction on how to perform the behaviour	Demonstration of the behaviour			100	50	67	0	0	0	0	5
2	2	Problem solving	Action planning	Discrepancy between current behaviour and goal	Instruction on how to perform the behaviour	Graded tasks			50	100	100	0	0	0	0	5
3	2	Problem solving	Action planning	Discrepancy between current behaviour and goal	Demonstration of the behaviour	Credible source			100	50	67	0	0	0	0	5
2	2	Problem solving	Action planning	Discrepancy between current behaviour and goal	Graded tasks	Credible source			50	100	100	0	0	0	0	5
1	1	Problem solving	Action planning	Social support (emotional)	Demonstration of the behaviour	Behavioural practice/rehearsal			100	0	100	0	0	0	0	5
1	1	Problem solving	Action planning	Social support (emotional)	Prompts/cues	Behavioural practice/rehearsal			100	0	100	0	0	0	0	5
2	2	Problem solving	Action planning	Instruction on how to perform the behaviour	Demonstration of the behaviour	Prompts/cues			100	100	100	0	0	0	0	5
2	2	Problem solving	Action planning	Feedback on behaviour	Social support (emotional)	Demonstration of the behaviour			100	100	100	0	0	0	0	5
1	1	Problem solving	Action planning	self monitoring of behaviour	Social support (emotional)	Prompts/cues			100	0	100	0	0	0	0	5
1	1	Problem solving	Action planning	self monitoring of behaviour	Social support (emotional)	Behavioural practice/rehearsal			100	0	100	0	0	0	0	5
2	2	Problem solving	Action planning	Review of behaviour goals	Demonstration of the behaviour	Credible source			100	0	100	0	0	0	0	5
1	1	Problem solving	Action planning	Review of behaviour goals	Prompts/cues	Credible source			50	0	100	0	0	0	0	5
1	1	Goal setting (behaviour)	Discrepancy between current behaviour and goal	Feedback on behaviour	social support	Instruction on how to perform the behaviour	Demonstration of the behaviour		67	100	100	0	0	0	0	6
1	1	Goal setting (behaviour)	Discrepancy between current behaviour and goal	self monitoring of behaviour	social support	Instruction on how to perform the behaviour	Demonstration of the behaviour		67	100	100	0	0	0	0	6
0	0	Goal setting (behaviour)	Feedback on behaviour	self monitoring of behaviour	social support	Social comparison	Credible source		67	0	0	0	0	0	0	6
0	0	Goal setting (behaviour)	Feedback on behaviour	social support	Instruction on how to perform the behaviour	Social comparison	Credible source		67	0	0	0	0	0	0	6
0	0	Goal setting (behaviour)	self monitoring of behaviour	social support	Instruction on how to perform the behaviour	Social comparison	Credible source		67	0	0	0	0	0	0	6
1	1	Problem solving	Action planning	self monitoring of behaviour	Information about health consequences	Demonstration of the behaviour			50	0	100	0	0	0	0	5
1	1	Problem solving	Action planning	self monitoring of behaviour	Prompts/cues	Behavioural practice/rehearsal			100	0	100	0	0	0	0	5
1	1	Problem solving	Action planning	Social support (emotional)	Demonstration of the behaviour	Prompts/cues			100	0	100	0	0	0	0	5
1	1	Problem solving	Review of behaviour goals	Discrepancy between current behaviour and goal	Feedback on behaviour	Credible source			50	0	100	0	0	0	0	5
1	1	Problem solving	Review of behaviour goals	Discrepancy between current behaviour and goal	self monitoring of behaviour	Instruction on how to perform the behaviour			50	0	100	0	0	0	0	5
1	1	Problem solving	Review of behaviour goals	Discrepancy between current behaviour and goal	self monitoring of behaviour	Credible source			50	0	100	0	0	0	0	5
1	1	Problem solving	Review of behaviour goals	Discrepancy between current behaviour and goal	Instruction on how to perform the behaviour	Information about health consequences			50	0	100	0	0	0	0	5
1	1	Problem solving	Review of behaviour goals	Discrepancy between current behaviour and goal	Instruction on how to perform the behaviour	Prompts/cues			50	0	100	0	0	0	0	5

2	2	self monitoring of behaviour	Information about health consequences	Behavioural practice/rehearsal	Reduce negative emotions	0	0	100	0	0	0	0	4
1	1	self monitoring of behaviour	Information about health consequences	Graded tasks	Credible source	50	100	100	0	0	0	0	4
2	2	self monitoring of behaviour	Information about health consequences	Graded tasks	Reduce negative emotions	0	0	100	0	0	0	0	4
1	1	self monitoring of behaviour	Demonstration of the behaviour	Prompts/cues	Behavioural practice/rehearsal	100	0	100	0	0	0	0	4
2	2	Feedback on behaviour	self monitoring of behaviour	Behavioural practice/rehearsal	Reduce negative emotions	0	0	100	0	0	0	0	4
2	2	Feedback on behaviour	self monitoring of behaviour	Graded tasks	Reduce negative emotions	0	0	100	0	0	0	0	4
2	2	Review of behaviour goals	Feedback on behaviour	Instruction on how to perform the behaviour	Reduce negative emotions	0	0	100	0	0	0	0	4
2	2	Review of behaviour goals	Feedback on behaviour	Information about health consequences	Behavioural practice/rehearsal	0	0	100	0	0	0	0	4
1	1	Review of behaviour goals	Feedback on behaviour	Information about health consequences	Credible source	50	0	100	0	0	0	0	4
2	2	Review of behaviour goals	Feedback on behaviour	Information about health consequences	Reduce negative emotions	0	0	100	0	0	0	0	4
2	2	Review of behaviour goals	Feedback on behaviour	Demonstration of the behaviour	Credible source	100	0	100	0	0	0	0	4
1	1	Review of behaviour goals	Feedback on behaviour	Prompts/cues	Credible source	50	0	100	0	0	0	0	4
2	2	Review of behaviour goals	Feedback on behaviour	Behavioural practice/rehearsal	Credible source	100	0	100	0	0	0	0	4
2	2	Review of behaviour goals	Feedback on behaviour	Behavioural practice/rehearsal	Reduce negative emotions	0	0	100	0	0	0	0	4
2	2	Review of behaviour goals	self monitoring of behaviour	Behavioural practice/rehearsal	Reduce negative emotions	0	0	100	0	0	0	0	4
2	2	Review of behaviour goals	self monitoring of behaviour	Graded tasks	Reduce negative emotions	0	0	100	0	0	0	0	4
2	2	Review of behaviour goals	Instruction on how to perform the behaviour	Information about health consequences	Reduce negative emotions	0	0	100	0	0	0	0	4
1	1	Review of behaviour goals	Instruction on how to perform the behaviour	Prompts/cues	Graded tasks	50	0	100	0	0	0	0	4
1	1	Review of behaviour goals	Instruction on how to perform the behaviour	Prompts/cues	Credible source	50	0	100	0	0	0	0	4
2	2	Review of behaviour goals	Instruction on how to perform the behaviour	Behavioural practice/rehearsal	Reduce negative emotions	0	0	100	0	0	0	0	4
1	1	Review of behaviour goals	Discrepancy between current behaviour and goal	Prompts/cues	Credible source	50	0	100	0	0	0	0	4
2	2	Review of behaviour goals	Feedback on behaviour	self monitoring of behaviour	Reduce negative emotions	0	0	100	0	0	0	0	4
2	2	Discrepancy between current behaviour and goal	Feedback on behaviour	Graded tasks	Credible source	50	100	100	0	0	0	0	4
2	2	Review of behaviour goals	self monitoring of behaviour	Instruction on how to perform the behaviour	Reduce negative emotions	0	0	100	0	0	0	0	4
2	2	Review of behaviour goals	self monitoring of behaviour	Information about health consequences	Behavioural practice/rehearsal	0	0	100	0	0	0	0	4
1	1	Review of behaviour goals	self monitoring of behaviour	Information about health consequences	Credible source	50	0	100	0	0	0	0	4
2	2	Review of behaviour goals	self monitoring of behaviour	Information about health consequences	Reduce negative emotions	0	0	100	0	0	0	0	4
2	2	Review of behaviour goals	self monitoring of behaviour	Demonstration of the behaviour	Credible source	100	0	100	0	0	0	0	4
1	1	Review of behaviour goals	self monitoring of behaviour	Prompts/cues	Credible source	50	0	100	0	0	0	0	4
2	2	Review of behaviour goals	self monitoring of behaviour	Behavioural practice/rehearsal	Credible source	100	0	100	0	0	0	0	4
2	2	Review of behaviour goals	Information about health consequences	Behavioural practice/rehearsal	Reduce negative emotions	0	0	100	0	0	0	0	4
2	2	Review of behaviour goals	Information about health consequences	Graded tasks	Reduce negative emotions	0	0	100	0	0	0	0	4

2	2	Action planning	Review of behaviour goals	Reduce negative emotions				0	0	100	0	0	0	0	3
1	0	social support	Instruction on how to perform the behaviour	Information about health consequences	Social comparison	Credible source		100	0	0	0	0	0	0	5
1	0	Problem solving	Action planning	Discrepancy between current behaviour and goal	social support	Instruction on how to perform the behaviour	Information about health consequences	50	0	0	0	0	0	0	6
1	0	Problem solving	Action planning	Discrepancy between current behaviour and goal	social support	Information about health consequences	Credible source	50	0	0	0	0	0	0	6
1	0	Problem solving	Action planning	social support	Instruction on how to perform the behaviour	Social comparison	Credible source	100	0	0	0	0	0	0	6
1	0	Problem solving	Action planning	social support	Information about health consequences	Social comparison	Credible source	100	0	0	0	0	0	0	6
1	0	Problem solving	Action planning	Instruction on how to perform the behaviour	Information about health consequences	Social comparison	Credible source	100	0	0	0	0	0	0	6
1	0	Problem solving	Discrepancy between current behaviour and goal	social support	Instruction on how to perform the behaviour	Information about health consequences	Credible source	50	0	0	0	0	0	0	6
1	0	Problem solving	social support	Instruction on how to perform the behaviour	Information about health consequences	Social comparison	Credible source	100	0	0	0	0	0	0	6
0	0	Action planning	Feedback on behaviour	Prompts/cues	Reduce prompts/cues			33	0	0	0	0	0	0	4
0	0	Action planning	social support	Instruction on how to perform the behaviour	Reduce prompts/cues			33	0	0	0	0	0	0	4
0	0	Action planning	social support	Prompts/cues	Reduce prompts/cues			33	0	0	0	0	0	0	4
0	0	Action planning	Instruction on how to perform the behaviour	Prompts/cues	Reduce prompts/cues			33	0	0	0	0	0	0	4
0	0	Feedback on behaviour	social support	Prompts/cues	Reduce prompts/cues			33	0	0	0	0	0	0	4
0	0	Feedback on behaviour	Instruction on how to perform the behaviour	Prompts/cues	Reduce prompts/cues			33	0	0	0	0	0	0	4
0	0	social support	Instruction on how to perform the behaviour	Prompts/cues	Reduce prompts/cues			33	0	0	0	0	0	0	4
0	0	Goal setting (behaviour)	Action planning	Feedback on behaviour	social support	Reduce prompts/cues		33	0	0	0	0	0	0	5
2	2	Goal setting (behaviour)	Behavioural practice/rehearsal	Reduce negative emotions				0	0	100	0	0	0	0	3
2	2	Goal setting (behaviour)	Graded tasks	Reduce negative emotions				0	0	100	0	0	0	0	3
1	1	Problem solving	Action planning	Generalisation of target behaviour				50	0	100	0	0	0	0	3
0	0	Goal setting (behaviour)	Feedback on behaviour	Prompts/cues	Reduce prompts/cues			33	0	0	0	0	0	0	4
0	0	Goal setting (behaviour)	social support	Prompts/cues	Reduce prompts/cues			33	0	0	0	0	0	0	4
0	0	Goal setting (behaviour)	Instruction on how to perform the behaviour	Prompts/cues	Reduce prompts/cues			33	0	0	0	0	0	0	4
0	0	Action planning	Feedback on behaviour	social support	Reduce prompts/cues			33	0	0	0	0	0	0	4
0	0	Action planning	Feedback on behaviour	Instruction on how to perform the behaviour	Reduce prompts/cues			33	0	0	0	0	0	0	4
0	0	Instruction on how to perform the behaviour	Prompts/cues	Reduce prompts/cues				33	0	0	0	0	0	0	3
0	0	Goal setting (behaviour)	Action planning	Feedback on behaviour	Reduce prompts/cues			33	0	0	0	0	0	0	4
0	0	Goal setting (behaviour)	Action planning	social support	Reduce prompts/cues			33	0	0	0	0	0	0	4
0	0	Goal setting (behaviour)	Action planning	Instruction on how to perform the behaviour	Reduce prompts/cues			33	0	0	0	0	0	0	4
0	0	Goal setting (behaviour)	Action planning	Prompts/cues	Reduce prompts/cues			33	0	0	0	0	0	0	4
2	2	Goal setting (behaviour)	Instruction on how to perform the behaviour	Reduce negative emotions				0	0	100	0	0	0	0	3
1	1	Goal setting (behaviour)	Information about health consequences	Generalisation of target behaviour				100	0	100	0	0	0	0	3
[illegible]															

0	0	Goal setting (behaviour)	Review of behaviour goals	social support	Instruction on how to perform the behaviour	Prompts/cues		
0	0	Goal setting (behaviour)	Review of behaviour goals	social support	Information about health consequences	Prompts/cues		
0	0	Goal setting (behaviour)	Discrepancy between current behaviour and goal	Feedback on behaviour	social support	Prompts/cues		
1	1	Goal setting (behaviour)	Discrepancy between current behaviour and goal	Feedback on behaviour	social support	Graded tasks		
0	0	Discrepancy between current behaviour and goal	self monitoring of behaviour	social support	Prompts/cues			
1	1	Discrepancy between current behaviour and goal	self monitoring of behaviour	social support	Graded tasks			
0	0	Discrepancy between current behaviour and goal	social support	Instruction on how to perform the behaviour	Prompts/cues			
1	1	Discrepancy between current behaviour and goal	social support	Instruction on how to perform the behaviour	Graded tasks			
0	0	social support	Instruction on how to perform the behaviour	Information about health consequences	Prompts/cues			
1	1	Goal setting (behaviour)	Problem solving	Action planning	Discrepancy between current behaviour and goal	social support		
0	0	Goal setting (behaviour)	Problem solving	Discrepancy between current behaviour and goal	social support	Prompts/cues		
1	1	Goal setting (behaviour)	Problem solving	Discrepancy between current behaviour and goal	social support	Graded tasks		
3	3	Problem solving	Feedback on behaviour	Social support (emotional)	Instruction on how to perform the behaviour	Demonstration of the behaviour	Graded tasks	
3	3	Problem solving	Feedback on behaviour	Social support (emotional)	Instruction on how to perform the behaviour	Demonstration of the behaviour	Credible source	
3	3	Problem solving	Feedback on behaviour	Social support (emotional)	Instruction on how to perform the behaviour	Graded tasks	Credible source	
3	3	Problem solving	Feedback on behaviour	Social support (emotional)	Demonstration of the behaviour	Graded tasks	Credible source	
2	2	Problem solving	self monitoring of behaviour	Feedback on outcomes of behaviour	Instruction on how to perform the behaviour	Demonstration of the behaviour	Graded tasks	
3	3	Problem solving	self monitoring of behaviour	Social support (emotional)	Instruction on how to perform the behaviour	Demonstration of the behaviour	Graded tasks	
3	3	Problem solving	self monitoring of behaviour	Social support (emotional)	Instruction on how to perform the behaviour	Demonstration of the behaviour	Credible source	
3	3	Problem solving	self monitoring of behaviour	Social support (emotional)	Instruction on how to perform the behaviour	Graded tasks	Credible source	
0	0	Action planning	Review of behaviour goals	social support	Information about health consequences	Prompts/cues		
0	0	Action planning	Feedback on behaviour	social support	Information about health consequences	Credible source		
1	1	Action planning	self monitoring of behaviour	social support	Prompts/cues	Credible source		
0	0	Action planning	social support	Instruction on how to perform the behaviour	Information about health consequences	Prompts/cues		
0	0	Review of behaviour goals	Feedback on behaviour	self monitoring of behaviour	social support	Prompts/cues		
0	0	Review of behaviour goals	Feedback on behaviour	social support	Instruction on how to perform the behaviour	Prompts/cues		
0	0	Review of behaviour goals	Feedback on behaviour	social support	Information about health consequences	Prompts/cues		
0	0	Review of behaviour goals	self monitoring of behaviour	social support	Instruction on how to perform the behaviour	Prompts/cues		
0	0	Problem solving	self monitoring of behaviour	social support	Information about health consequences	Prompts/cues		
1	1	Problem solving	self monitoring of behaviour	social support	Prompts/cues	Credible source		
0	0	Problem solving	social support	Instruction on how to perform the behaviour	Information about health consequences	Prompts/cues		
1	1	Problem solving	social support	Instruction on how to perform the behaviour	Prompts/cues	Credible source		
0	0	Action planning	Review of behaviour goals	Feedback on behaviour	social support	Prompts/cues		
0	0	Action planning	Review of behaviour goals	Feedback on behaviour	social support	Credible source		
0	0	Action planning	Review of behaviour goals	self monitoring of behaviour	social support	Credible source		
0	0	Action planning	Review of behaviour goals	social support	Instruction on how to perform the behaviour	Prompts/cues		
0	0	Problem solving	Discrepancy between current behaviour and goal	self monitoring of behaviour	social support	Prompts/cues		
1	1	Problem solving	Discrepancy between current behaviour and goal	self monitoring of behaviour	social support	Graded tasks		
1	1	Problem solving	Discrepancy between current behaviour and goal	self monitoring of behaviour	social support	Credible source		
0	0	Problem solving	Discrepancy between current behaviour and goal	social support	Instruction on how to perform the behaviour	Prompts/cues		
1	1	Problem solving	Discrepancy between current behaviour and goal	social support	Instruction on how to perform the behaviour	Graded tasks		
0	0	Problem solving	Feedback on behaviour	social support	Information about health consequences	Prompts/cues		
0	0	Problem solving	Feedback on behaviour	social support	Information about health consequences	Credible source		
1	1	Problem solving	Feedback on behaviour	social support	Prompts/cues	Credible source		
1	1	Discrepancy between current behaviour and goal	self monitoring of behaviour	social support	Instruction on how to perform the behaviour	Graded tasks		
0	0	Feedback on behaviour	social support	Instruction on how to perform the behaviour	Information about health consequences	Prompts/cues		
0	0	Feedback on behaviour	social support	Instruction on how to perform the behaviour	Information about health consequences	Credible source		
0	0	self monitoring of behaviour	social support	Instruction on how to perform the behaviour	Information about health consequences	Prompts/cues		
1	1	self monitoring of behaviour	social support	Instruction on how to perform the behaviour	Prompts/cues	Credible source		
0	0	Goal setting (behaviour)	Problem solving	Action planning	Review of behaviour goals	social support	Credible source	
1	1	Goal setting (behaviour)	Problem solving	Action planning	Discrepancy between current behaviour and goal	Feedback on behaviour	social support	
1	1	Goal setting (behaviour)	Problem solving	Action planning	Discrepancy between current behaviour and goal	self monitoring of behaviour	social support	
3	3	Goal setting (outcome)	self monitoring of behaviour	Social support (emotional)	Instruction on how to perform the behaviour	Demonstration of the behaviour	Credible source	
0	0	Review of behaviour goals	self monitoring of behaviour	social support	Information about health consequences	Prompts/cues		
0	0	Review of behaviour goals	social support	Instruction on how to perform the behaviour	Information about health consequences	Prompts/cues		
0	0	Discrepancy between current behaviour and goal	Feedback on behaviour	self monitoring of behaviour	social support	Prompts/cues		
1	1	Discrepancy between current behaviour and goal	Feedback on behaviour	self monitoring of behaviour	social support	Graded tasks		
0	0	Discrepancy between current behaviour and goal	Feedback on behaviour	social support	Instruction on how to perform the behaviour	Prompts/cues		
1	1	Discrepancy between current behaviour and goal	Feedback on behaviour	social support	Instruction on how to perform the behaviour	Graded tasks		
0	0	Discrepancy between current behaviour and goal	self monitoring of behaviour	social support	Instruction on how to perform the behaviour	Prompts/cues		
3	3	Goal setting (outcome)	Feedback on behaviour	self monitoring of behaviour	Social support (emotional)	Demonstration of the behaviour	Credible source	
3	3	Goal setting (outcome)	Feedback on behaviour	self monitoring of behaviour	Social support (emotional)	Graded tasks	Credible source	
3	3	Goal setting (outcome)	Feedback on behaviour	self monitoring of behaviour	Instruction on how to perform the behaviour	Credible source	Avoidance/reducing exposure to the behaviour	
3	3	Goal setting (outcome)	Feedback on behaviour	Social support (emotional)	Instruction on how to perform the behaviour	Demonstration of the behaviour	Graded tasks	
3	3	Goal setting (outcome)	Feedback on behaviour	Social support (emotional)	Instruction on how to perform the behaviour	Demonstration of the behaviour	Credible source	
3	3	Goal setting (outcome)	Feedback on behaviour	Social support (emotional)	Instruction on how to perform the behaviour	Graded tasks	Credible source	
3	3	Goal setting (outcome)	Feedback on behaviour	Social support (emotional)	Demonstration of the behaviour	Graded tasks	Credible source	
3	3	Goal setting (outcome)	self monitoring of behaviour	Social support (emotional)	Instruction on how to perform the behaviour	Demonstration of the behaviour	Graded tasks	
3	3	Goal setting (outcome)	self monitoring of behaviour	Social support (emotional)	Demonstration of the behaviour	Graded tasks	Credible source	
2	2	Problem solving	self monitoring of behaviour	Instruction on how to perform the behaviour	Demonstration of the behaviour	Generalisation of target behaviour	Credible source	

0	0	Goal setting (behaviour)	Action planning	Review of behaviour goals	self monitoring of behaviour	social support	Instruction on how to perform the behaviour	Information about health consequences	Prompts/cues
0	0	Goal setting (behaviour)	Action planning	Feedback on behaviour	self monitoring of behaviour	social support	Instruction on how to perform the behaviour	Information about health consequences	Prompts/cues
0	0	Goal setting (behaviour)	Action planning	Feedback on behaviour	self monitoring of behaviour	social support	Instruction on how to perform the behaviour	Information about health consequences	Credible source
1	1	Goal setting (behaviour)	Action planning	Feedback on behaviour	self monitoring of behaviour	social support	Instruction on how to perform the behaviour	Prompts/cues	Credible source
0	0	Goal setting (behaviour)	Review of behaviour goals	Feedback on behaviour	self monitoring of behaviour	social support	Instruction on how to perform the behaviour	Information about health consequences	Prompts/cues
0	0	Problem solving	Action planning	Review of behaviour goals	Feedback on behaviour	self monitoring of behaviour	social support	Instruction on how to perform the behaviour	Prompts/cues
0	0	Problem solving	Action planning	Review of behaviour goals	Feedback on behaviour	self monitoring of behaviour	social support	Instruction on how to perform the behaviour	Credible source
1	1	Goal setting (behaviour)	Problem solving	Discrepancy between current behaviour and goal	Feedback on behaviour	self monitoring of behaviour	social support	Instruction on how to perform the behaviour	Graded tasks
1	1	Goal setting (behaviour)	Problem solving	Discrepancy between current behaviour and goal	Feedback on behaviour	self monitoring of behaviour	social support	Instruction on how to perform the behaviour	Credible source
0	0	Goal setting (behaviour)	Problem solving	Feedback on behaviour	self monitoring of behaviour	social support	Instruction on how to perform the behaviour	Information about health consequences	Prompts/cues
0	0	Goal setting (behaviour)	Problem solving	Feedback on behaviour	self monitoring of behaviour	social support	Instruction on how to perform the behaviour	Information about health consequences	Credible source
1	1	Goal setting (behaviour)	Problem solving	Feedback on behaviour	self monitoring of behaviour	social support	Instruction on how to perform the behaviour	Prompts/cues	Credible source
0	0	Goal setting (behaviour)	Action planning	Review of behaviour goals	Feedback on behaviour	self monitoring of behaviour	social support	Instruction on how to perform the behaviour	Prompts/cues
0	0	Goal setting (behaviour)	Action planning	Review of behaviour goals	Feedback on behaviour	self monitoring of behaviour	social support	Instruction on how to perform the behaviour	Credible source
0	0	Goal setting (behaviour)	Action planning	Review of behaviour goals	Feedback on behaviour	self monitoring of behaviour	social support	Information about health consequences	Prompts/cues
3	3	Goal setting (behaviour)	self monitoring of behaviour	Social support (emotional)	Instruction on how to perform the behaviour	Demonstration of the behaviour	Credible source		
3	3	Goal setting (behaviour)	self monitoring of behaviour	Social support (emotional)	Instruction on how to perform the behaviour	Graded tasks	Credible source		
3	3	Goal setting (behaviour)	self monitoring of behaviour	Social support (emotional)	Demonstration of the behaviour	Graded tasks	Credible source		
2	2	Goal setting (behaviour)	self monitoring of behaviour	Instruction on how to perform the behaviour	Demonstration of the behaviour	Generalisation of target behaviour	Credible source		
3	3	Goal setting (behaviour)	Social support (emotional)	Instruction on how to perform the behaviour	Demonstration of the behaviour	Graded tasks	Credible source		
3	2	Problem solving	Goal setting (outcome)	Feedback on behaviour	self monitoring of behaviour	Feedback on outcomes of behaviour	Instruction on how to perform the behaviour		
2	2	Goal setting (behaviour)	Problem solving	Feedback on behaviour	Motivational Interviewing				
2	2	Goal setting (behaviour)	Problem solving	self monitoring of behaviour	Motivational Interviewing				
1	1	Goal setting (behaviour)	Feedback on behaviour	self monitoring of behaviour	Instruction on how to perform the behaviour	Demonstration of the behaviour	Social comparison		
2	2	Goal setting (behaviour)	Feedback on behaviour	Feedback on outcomes of behaviour	Instruction on how to perform the behaviour	Demonstration of the behaviour	Graded tasks		
3	3	Goal setting (behaviour)	Feedback on behaviour	Social support (emotional)	Instruction on how to perform the behaviour	Demonstration of the behaviour	Graded tasks		
3	3	Goal setting (behaviour)	Feedback on behaviour	Social support (emotional)	Instruction on how to perform the behaviour	Demonstration of the behaviour	Credible source		
3	3	Goal setting (behaviour)	Feedback on behaviour	Social support (emotional)	Instruction on how to perform the behaviour	Graded tasks	Credible source		
3	3	Goal setting (behaviour)	Feedback on behaviour	Social support (emotional)	Demonstration of the behaviour	Graded tasks	Credible source		
2	2	Goal setting (behaviour)	self monitoring of behaviour	Feedback on outcomes of behaviour	Instruction on how to perform the behaviour	Demonstration of the behaviour	Graded tasks		
3	3	Goal setting (behaviour)	self monitoring of behaviour	Social support (emotional)	Instruction on how to perform the behaviour	Demonstration of the behaviour	Graded tasks		
2	2	Goal setting (behaviour)	Feedback on behaviour	self monitoring of behaviour	Feedback on outcomes of behaviour	Instruction on how to perform the behaviour	Graded tasks		
2	2	Goal setting (behaviour)	Feedback on behaviour	self monitoring of behaviour	Feedback on outcomes of behaviour	Demonstration of the behaviour	Graded tasks		
3	3	Goal setting (behaviour)	Feedback on behaviour	self monitoring of behaviour	Social support (emotional)	Instruction on how to perform the behaviour	Demonstration of the behaviour		
3	3	Goal setting (behaviour)	Feedback on behaviour	self monitoring of behaviour	Social support (emotional)	Instruction on how to perform the behaviour	Graded tasks		
3	3	Goal setting (behaviour)	Feedback on behaviour	self monitoring of behaviour	Social support (emotional)	Instruction on how to perform the behaviour	Credible source		
3	3	Goal setting (behaviour)	Feedback on behaviour	self monitoring of behaviour	Social support (emotional)	Demonstration of the behaviour	Graded tasks		
3	3	Goal setting (behaviour)	Feedback on behaviour	self monitoring of behaviour	Social support (emotional)	Demonstration of the behaviour	Credible source		
3	3	Goal setting (behaviour)	Feedback on behaviour	self monitoring of behaviour	Social support (emotional)	Graded tasks	Credible source		
0	0	Goal setting (behaviour)	Problem solving	Action planning	Feedback on behaviour	self monitoring of behaviour	social support	Instruction on how to perform the behaviour	Information about health consequences
1	1	Goal setting (behaviour)	Problem solving	Action planning	Feedback on behaviour	self monitoring of behaviour	social support	Instruction on how to perform the behaviour	Prompts/cues
0	0	Goal setting (behaviour)	Problem solving	Review of behaviour goals	Feedback on behaviour	self monitoring of behaviour	social support	Instruction on how to perform the behaviour	Information about health consequences
0	0	Goal setting (behaviour)	Action planning	Review of behaviour goals	Feedback on behaviour	self monitoring of behaviour	social support	Instruction on how to perform the behaviour	Information about health consequences
0	0	Problem solving	Action planning	Review of behaviour goals	Feedback on behaviour	self monitoring of behaviour	social support	Instruction on how to perform the behaviour	Information about health consequences
0	0	Goal setting (behaviour)	Problem solving	Action planning	Review of behaviour goals	Feedback on behaviour	self monitoring of behaviour	social support	Instruction on how to perform the behaviour
2	2	Goal setting (behaviour)	Goal setting (outcome)	Instruction on how to perform the behaviour	Demonstration of the behaviour	Generalisation of target behaviour	Credible source		
2	2	Goal setting (behaviour)	Feedback on behaviour	self monitoring of behaviour	Feedback on outcomes of behaviour	Instruction on how to perform the behaviour	Demonstration of the behaviour		
0	0	Problem solving	Review of behaviour goals	Feedback on behaviour	Information about health consequences	Adding object to the environment			
0	0	Problem solving	Review of behaviour goals	self monitoring of behaviour	Information about health consequences	Adding object to the environment			
0	0	Review of behaviour goals	Feedback on behaviour	self monitoring of behaviour	Information about health consequences	Adding object to the environment			
0	0	Goal setting (behaviour)	Problem solving	Review of behaviour goals	Feedback on behaviour	Information about health consequences	Adding object to the environment		
0	0	Goal setting (behaviour)	Problem solving	Review of behaviour goals	self monitoring of behaviour	Information about health consequences	Adding object to the environment		
0	0	Goal setting (behaviour)	Review of behaviour goals	Feedback on behaviour	self monitoring of behaviour	Information about health consequences	Adding object to the environment		
0	0	Problem solving	Review of behaviour goals	Feedback on behaviour	self monitoring of behaviour	Information about health consequences	Adding object to the environment		
0	0	Goal setting (behaviour)	Problem solving	Review of behaviour goals	Feedback on behaviour	self monitoring of behaviour	Information about health consequences	Adding object to the environment	
3	3	Goal setting (behaviour)	Goal setting (outcome)	self monitoring of behaviour	Social support (emotional)	Graded tasks	Credible source		
2	2	Goal setting (behaviour)	Goal setting (outcome)	self monitoring of behaviour	Instruction on how to perform the behaviour	Demonstration of the behaviour	Generalisation of target behaviour		
2	2	Goal setting (behaviour)	Goal setting (outcome)	self monitoring of behaviour	Instruction on how to perform the behaviour	Generalisation of target behaviour	Credible source		
2	2	Goal setting (behaviour)	Goal setting (outcome)	self monitoring of behaviour	Demonstration of the behaviour	Generalisation of target behaviour	Credible source		
3	3	Goal setting (behaviour)	Goal setting (outcome)	Social support (emotional)	Instruction on how to perform the behaviour	Demonstration of the behaviour	Graded tasks		
3	3	Goal setting (behaviour)	Goal setting (outcome)	Social support (emotional)	Instruction on how to perform the behaviour	Demonstration of the behaviour	Credible source		
3	3	Goal setting (behaviour)	Goal setting (outcome)	Social support (emotional)	Instruction on how to perform the behaviour	Graded tasks	Credible source		
3	3	Goal setting (behaviour)	Goal setting (outcome)	Social support (emotional)	Demonstration of the behaviour	Graded tasks	Credible source		
3	3	Goal setting (behaviour)	Goal setting (outcome)	Feedback on behaviour	Social support (emotional)	Demonstration of the behaviour	Graded tasks		
3	3	Goal setting (behaviour)	Goal setting (outcome)	Feedback on behaviour	Social support (emotional)	Demonstration of the behaviour	Credible source		

1	1	Goal setting (behaviour)	Action planning	self monitoring of behaviour	social support	Instruction on how to perform the behaviour	Social reward
1	1	Goal setting (behaviour)	Action planning	self monitoring of behaviour	social support	Graded tasks	Social reward
2	2	Goal setting (behaviour)	self monitoring of behaviour	Demonstration of the behaviour	Generalisation of target behaviour	Credible source	
2	2	Goal setting (behaviour)	Feedback on outcomes of behaviour	Instruction on how to perform the behaviour	Demonstration of the behaviour	Graded tasks	
3	3	Goal setting (behaviour)	Social support (emotional)	Instruction on how to perform the behaviour	Demonstration of the behaviour	Graded tasks	
3	3	Goal setting (behaviour)	Social support (emotional)	Instruction on how to perform the behaviour	Demonstration of the behaviour	Credible source	
3	3	Goal setting (behaviour)	Social support (emotional)	Instruction on how to perform the behaviour	Graded tasks	Credible source	
3	3	Goal setting (behaviour)	Social support (emotional)	Demonstration of the behaviour	Graded tasks	Credible source	
2	2	Goal setting (behaviour)	Instruction on how to perform the behaviour	Demonstration of the behaviour	Generalisation of target behaviour	Credible source	
2	2	Problem solving	Goal setting (outcome)	Feedback on behaviour	self monitoring of behaviour	Self monitoring of outcome(s) of behaviour	
3	3	Goal setting (behaviour)	self monitoring of behaviour	Social support (emotional)	Instruction on how to perform the behaviour	Graded tasks	
3	3	Goal setting (behaviour)	self monitoring of behaviour	Social support (emotional)	Instruction on how to perform the behaviour	Credible source	
3	3	Goal setting (behaviour)	self monitoring of behaviour	Social support (emotional)	Demonstration of the behaviour	Graded tasks	
3	3	Goal setting (behaviour)	self monitoring of behaviour	Social support (emotional)	Demonstration of the behaviour	Credible source	
3	3	Goal setting (behaviour)	self monitoring of behaviour	Social support (emotional)	Graded tasks	Credible source	
1	1	Goal setting (behaviour)	self monitoring of behaviour	Instruction on how to perform the behaviour	Demonstration of the behaviour	Social comparison	
2	2	Goal setting (behaviour)	self monitoring of behaviour	Instruction on how to perform the behaviour	Demonstration of the behaviour	Generalisation of target behaviour	
2	2	Goal setting (behaviour)	self monitoring of behaviour	Instruction on how to perform the behaviour	Generalisation of target behaviour	Credible source	
1	1	Goal setting (behaviour)	Feedback on behaviour	Instruction on how to perform the behaviour	Demonstration of the behaviour	Social comparison	
2	2	Goal setting (behaviour)	self monitoring of behaviour	Feedback on outcomes of behaviour	Instruction on how to perform the behaviour	Demonstration of the behaviour	
2	2	Goal setting (behaviour)	self monitoring of behaviour	Feedback on outcomes of behaviour	Instruction on how to perform the behaviour	Graded tasks	
2	2	Goal setting (behaviour)	self monitoring of behaviour	Feedback on outcomes of behaviour	Demonstration of the behaviour	Graded tasks	
3	3	Goal setting (behaviour)	self monitoring of behaviour	Social support (emotional)	Instruction on how to perform the behaviour	Demonstration of the behaviour	
1	1	Problem solving	Action planning	self monitoring of behaviour	social support	Instruction on how to perform the behaviour	Social reward
0	0	Problem solving	Action planning	self monitoring of behaviour	social support	Instruction on how to perform the behaviour	Verbal persuasion about capability
1	1	Problem solving	Action planning	self monitoring of behaviour	social support	Prompts/cues	Adding object to the environment
2	2	Goal setting (behaviour)	Feedback on behaviour	Feedback on outcomes of behaviour	Instruction on how to perform the behaviour	Graded tasks	
2	2	Goal setting (behaviour)	Feedback on behaviour	Feedback on outcomes of behaviour	Demonstration of the behaviour	Graded tasks	
3	3	Goal setting (behaviour)	Feedback on behaviour	Social support (emotional)	Instruction on how to perform the behaviour	Demonstration of the behaviour	
3	3	Goal setting (behaviour)	Feedback on behaviour	Social support (emotional)	Instruction on how to perform the behaviour	Graded tasks	
3	3	Goal setting (behaviour)	Feedback on behaviour	Social support (emotional)	Instruction on how to perform the behaviour	Credible source	
3	3	Goal setting (behaviour)	Feedback on behaviour	Social support (emotional)	Demonstration of the behaviour	Graded tasks	

1	1	Goal setting (behaviour)	Feedback on behaviour	Instruction on how to perform the behaviour	Social reward	Adding object to the environment
0	0	Problem solving	Action planning	Feedback on behaviour	Information about health consequences	Adding object to the environment
0	0	Problem solving	Review of behaviour goals	Feedback on behaviour	self monitoring of behaviour	Social reward
0	0	Problem solving	Review of behaviour goals	Feedback on behaviour	Graded tasks	Adding object to the environment
0	0	Problem solving	Review of behaviour goals	self monitoring of behaviour	Graded tasks	Adding object to the environment
0	0	Goal setting (behaviour)	Review of behaviour goals	Feedback on behaviour	social support	Verbal persuasion about capability
0	0	Goal setting (behaviour)	Review of behaviour goals	Feedback on behaviour	Instruction on how to perform the behaviour	Verbal persuasion about capability
0	0	Goal setting (behaviour)	Review of behaviour goals	Feedback on behaviour	Graded tasks	Adding object to the environment
0	0	Goal setting (behaviour)	Review of behaviour goals	self monitoring of behaviour	social support	Verbal persuasion about capability
0	0	Goal setting (behaviour)	Review of behaviour goals	self monitoring of behaviour	Instruction on how to perform the behaviour	Verbal persuasion about capability
0	0	Goal setting (behaviour)	Review of behaviour goals	social support	Instruction on how to perform the behaviour	Verbal persuasion about capability
0	0	Goal setting (behaviour)	Feedback on behaviour	self monitoring of behaviour	Instruction on how to perform the behaviour	Verbal persuasion about capability
1	1	Goal setting (behaviour)	Feedback on behaviour	social support	Instruction on how to perform the behaviour	Social reward
0	0	Goal setting (behaviour)	Action planning	Review of behaviour goals	Feedback on behaviour	Verbal persuasion about capability
0	0	Goal setting (behaviour)	Action planning	Review of behaviour goals	self monitoring of behaviour	Verbal persuasion about capability
0	0	Goal setting (behaviour)	Action planning	Review of behaviour goals	social support	Verbal persuasion about capability
0	0	Goal setting (behaviour)	Action planning	Review of behaviour goals	Instruction on how to perform the behaviour	Verbal persuasion about capability
0	0	Goal setting (behaviour)	Action planning	Feedback on behaviour	Instruction on how to perform the behaviour	Verbal persuasion about capability
0	0	Goal setting (behaviour)	Action planning	Feedback on behaviour	Information about health consequences	Adding object to the environment
0	0	Goal setting (behaviour)	Review of behaviour goals	Feedback on behaviour	self monitoring of behaviour	Social reward
0	0	Goal setting (behaviour)	Review of behaviour goals	Feedback on behaviour	self monitoring of behaviour	Verbal persuasion about capability
0	0	Action planning	Review of behaviour goals	self monitoring of behaviour	social support	Verbal persuasion about capability
0	0	Action planning	Review of behaviour goals	self monitoring of behaviour	Instruction on how to perform the behaviour	Verbal persuasion about capability
0	0	Action planning	Review of behaviour goals	social support	Instruction on how to perform the behaviour	Verbal persuasion about capability
0	0	Action planning	Feedback on behaviour	self monitoring of behaviour	Instruction on how to perform the behaviour	Verbal persuasion about capability
0	0	Action planning	Feedback on behaviour	self monitoring of behaviour	Information about health consequences	Adding object to the environment
0	0	Action planning	Feedback on behaviour	social support	Instruction on how to perform the behaviour	Verbal persuasion about capability
0	0	Action planning	Feedback on behaviour	social support	Information about health consequences	Adding object to the environment
0	0	Action planning	Feedback on behaviour	Instruction on how to perform the behaviour	Information about health consequences	Adding object to the environment
1	1	Problem solving	Feedback on behaviour	social support	Social reward	Adding object to the environment
1	1	Problem solving	Feedback on behaviour	Instruction on how to perform the behaviour	Prompts/cues	Social reward
1	1	Problem solving	Feedback on behaviour	Instruction on how to perform the behaviour	Prompts/cues	Adding object to the environment

3	3	2	1	0	0	0	0	0	0	0	0	0	0	0	0	0	0	0	0	0	0	3	2	1	2	1	1	1	Problem solving	Feedback on behaviour	self monitoring of behaviour	social support	Instruction on how to perform the behaviour	Prompts/cues	Graded tasks	Social reward	100	100	100	0	0	0	0	8
3	3	2	1	0	0	0	0	0	0	0	0	0	0	0	0	0	0	0	0	0	0	3	2	1	2	1	1	1	Problem solving	Feedback on behaviour	self monitoring of behaviour	social support	Instruction on how to perform the behaviour	Prompts/cues	Graded tasks	Adding object to the environment	100	100	100	0	0	0	0	8
3	3	2	1	0	0	0	0	0	0	0	0	0	0	0	0	0	0	0	0	0	0	3	2	1	2	1	1	1	Problem solving	Feedback on behaviour	self monitoring of behaviour	social support	Instruction on how to perform the behaviour	Prompts/cues	Social reward	Adding object to the environment	100	100	100	0	0	0	0	8
3	3	2	1	0	0	0	0	0	0	0	0	0	0	0	0	0	0	0	0	0	0	3	2	1	2	1	1	1	Problem solving	Feedback on behaviour	self monitoring of behaviour	social support	Instruction on how to perform the behaviour	Graded tasks	Social reward	Adding object to the environment	100	100	100	0	0	0	0	8
3	3	2	1	0	0	0	0	0	0	0	0	0	0	0	0	0	0	0	0	0	0	3	2	1	2	1	1	1	Problem solving	Feedback on behaviour	self monitoring of behaviour	social support	Prompts/cues	Graded tasks	Social reward	Adding object to the environment	100	100	100	0	0	0	0	8
3	3	2	1	0	0	0	0	0	0	0	0	0	0	0	0	0	0	0	0	0	0	3	2	1	2	1	1	1	Problem solving	Feedback on behaviour	self monitoring of behaviour	Instruction on how to perform the behaviour	Prompts/cues	Graded tasks	Social reward	Adding object to the environment	100	100	100	0	0	0	0	8
3	3	2	1	0	0	0	0	0	0	0	0	0	0	0	0	0	0	0	0	0	0	3	2	1	2	1	1	1	Problem solving	Feedback on behaviour	social support	Instruction on how to perform the behaviour	Prompts/cues	Graded tasks	Social reward	Adding object to the environment	100	100	100	0	0	0	0	8
3	3	2	1	0	0	0	0	0	0	0	0	0	0	0	0	0	0	0	0	0	0	3	2	1	2	1	1	1	Feedback on behaviour	self monitoring of behaviour	social support	Instruction on how to perform the behaviour	Prompts/cues	Graded tasks	Social reward	Adding object to the environment	100	100	100	0	0	0	0	8
3	3	2	1	0	0	0	0	0	0	0	0	0	0	0	0	0	0	0	0	0	0	3	2	1	2	1	1	1	Goal setting (behaviour)	Feedback on behaviour	self monitoring of behaviour	social support	Instruction on how to perform the behaviour	Prompts/cues	Graded tasks	Social reward	100	100	100	0	0	0	0	8
3	3	2	1	0	0	0	0	0	0	0	0	0	0	0	0	0	0	0	0	0	0	3	2	1	2	1	1	1	Goal setting (behaviour)	Feedback on behaviour	self monitoring of behaviour	social support	Instruction on how to perform the behaviour	Prompts/cues	Graded tasks	Adding object to the environment	100	100	100	0	0	0	0	8
3	3	2	1	0	0	0	0	0	0	0	0	0	0	0	0	0	0	0	0	0	0	3	2	1	2	1	1	1	Goal setting (behaviour)	Feedback on behaviour	self monitoring of behaviour	social support	Instruction on how to perform the behaviour	Prompts/cues	Social reward	Adding object to the environment	100	100	100	0	0	0	0	8
3	3	2	1	0	0	0	0	0	0	0	0	0	0	0	0	0	0	0	0	0	0	3	2	1	2	1	1	1	Goal setting (behaviour)	Feedback on behaviour	self monitoring of behaviour	social support	Instruction on how to perform the behaviour	Graded tasks	Social reward	Adding object to the environment	100	100	100	0	0	0	0	8
3	3	2	1	0	0	0	0	0	0	0	0	0	0	0	0	0	0	0	0	0	0	3	2	1	2	1	1	1	Goal setting (behaviour)	Feedback on behaviour	self monitoring of behaviour	social support	Prompts/cues	Graded tasks	Social reward	Adding object to the environment	100	100	100	0	0	0	0	8
3	3	2	1	0	0	0	0	0	0	0	0	0	0	0	0	0	0	0	0	0	0	3	2	1	2	1	1	1	Goal setting (behaviour)	Feedback on behaviour	self monitoring of behaviour	Instruction on how to perform the behaviour	Prompts/cues	Graded tasks	Social reward	Adding object to the environment	100	100	100	0	0	0	0	8
3	3	2	1	0	0	0	0	0	0	0	0	0	0	0	0	0	0	0	0	0	0	3	2	1	2	1	1	1	Goal setting (behaviour)	Feedback on behaviour	social support	Instruction on how to perform the behaviour	Prompts/cues	Graded tasks	Social reward	Adding object to the environment	100	100	100	0	0	0	0	8
3	2	0	0	0	0	0	0	1	0	0	0	0	0	0	0	0	0	0	0	0	0	3	0	0	0	0	0	0	Problem solving	Action planning	Feedback on behaviour	self monitoring of behaviour	social support	Instruction on how to perform the behaviour	Information about health consequences	Adding object to the environment	67	0	0	0	0	0	0	8
3	3	2	1	0	0	0	0	0	0	0	0	0	0	0	0	0	0	0	0	0	0	3	2	1	2	1	1	1	Goal setting (behaviour)	Problem solving	Feedback on behaviour	social support	Instruction on how to perform the behaviour	Prompts/cues	Graded tasks	Social reward	100	100	100	0	0	0	0	8
3	3	2	1	0	0	0	0	0	0	0	0	0	0	0	0	0	0	0	0	0	0	3	2	1	2	1	1	1	Goal setting (behaviour)	Problem solving	Feedback on behaviour	social support	Instruction on how to perform the behaviour	Prompts/cues	Graded tasks	Adding object to the environment	100	100	100	0	0	0	0	8
3	3	2	1	0	0	0	0	0	0	0	0	0	0	0	0	0	0	0	0	0	0	3	2	1	2	1	1	1	Goal setting (behaviour)	Problem solving	Feedback on behaviour	social support	Instruction on how to perform the behaviour	Prompts/cues	Social reward	Adding object to the environment	100	100	100	0	0	0	0	8
3	3	2	1	0	0	0	0	0	0	0	0	0	0	0	0	0	0	0	0	0	0	3	2	1	2	1	1	1	Goal setting (behaviour)	Problem solving	Feedback on behaviour	social support	Instruction on how to perform the behaviour	Graded tasks	Social reward	Adding object to the environment	100	100	100	0	0	0	0	8
3	3	2	1	0	0	0	0	0	0	0	0	0	0	0	0	0	0	0	0	0	0	3	2	1	2	1	1	1	Goal setting (behaviour)	Problem solving	Feedback on behaviour	social support	Prompts/cues	Graded tasks	Social reward	Adding object to the environment	100	100	100	0	0	0	0	8
3	3	2	1	0	0	0	0	0	0	0	0	0	0	0	0	0	0	0	0	0	0	3	2	1	2	1	1	1	Goal setting (behaviour)	Problem solving	Feedback on behaviour	Instruction on how to perform the behaviour	Prompts/cues	Graded tasks	Social reward	Adding object to the environment	100	100	100	0	0	0	0	8
3	3	0	0	0	0	0	0	0	1	0	0	0	0	0	0	0	0	0	0	0	0	3	1	0	1	0	0	0	Goal setting (behaviour)	Action planning	Review of behaviour goals	Feedback on behaviour	self monitoring of behaviour	social support	Instruction on how to perform the behaviour	Verbal persuasion about capability	100	0	0	0	0	0	0	8
3	2	0	0	0	0	0	0	1	0	0	0	0	0	0	0	0	0	0	0	0	0	3	0	0	0	0	0	0	Goal setting (behaviour)	Action planning	Feedback on behaviour	self monitoring of behaviour	social support	Instruction on how to perform the behaviour	Information about health consequences	Adding object to the environment	67	0	0	0	0	0	0	8
3	2	0	0	0	0	0	0	1	0	0	0	0	0	0	0	0	0	0	0	0	0	3	0	0	0	0	0	0	Goal setting (behaviour)	Feedback on behaviour	Information about emotinal consequences	Social comparison					67	0	0	0	0	0	0	4
3	2	0	0	0	0	0	0	1	0	0	0	0	0	0	0	0	0	0	0	0	0	3	0	0	0	0	0	0	Goal setting (behaviour)	Feedback on behaviour	Information about emotinal consequences	Behavioural practice/rehearsal					67	0	0	0	0	0	0	4
3	2	0	0	0	0	0	0	1	0	0	0	0	0	0	0	0	0	0	0	0	0	3	0	0	0	0	0	0	Goal setting (behaviour)	self monitoring of behaviour	social support	Information about emotinal consequences					67	0	0	0	0	0	0	4
3	2	0	0	0	0	0	0	1	0	0	0	0	0	0	0	0	0	0	0	0	0	3	0	0	0	0	0	0	Goal setting (behaviour)	self monitoring of behaviour	Information abour social & environmental consequences	Information about emotinal consequences					67	0	0	0	0	0	0	4
3	2	0	0	0	0	0	0	1	0	0	0	0	0	0	0	0	0	0	0	0	0	3	0	0	0	0	0	0	Goal setting (behaviour)	self monitoring of behaviour	Information about emotinal consequences	Social comparison					67	0	0	0	0	0	0	4
3	2	0	0	0	0	0	0	1	0	0	0	0	0	0	0	0	0	0	0	0	0	3	0	0	0	0	0	0	Goal setting (behaviour)	self monitoring of behaviour	Information about emotinal consequences	Behavioural practice/rehearsal					67	0	0	0	0	0	0	4
3	2	0	0	0	0	0	0	1	0	0	0	0	0	0	0	0	0	0	0	0	0	3	0	0	0	0	0	0	Problem solving	Action planning	Feedback on behaviour	Information abour social & environmental consequences					67	0	0	0	0	0	0	4
3	2	0	0	0	0	0	0	1	0	0	0	0	0	0	0	0	0	0	0	0	0	3	0	0	0	0	0	0	Problem solving	Action planning	Feedback on behaviour	Information about emotinal consequences					67	0	0	0	0	0	0	4

2	2	self monitoring of behaviour	Demonstration of the behaviour	Generalisation of target behaviour		
1	1	self monitoring of behaviour	Social comparison	Graded tasks		
2	2	self monitoring of behaviour	Generalisation of target behaviour	Credible source		
0	0	Feedback on behaviour	Instruction on how to perform the behaviour	Information abour social & environmental consequences	Information about emotinal consequences	Behavioural practice/rehearsal
0	0	Feedback on behaviour	Instruction on how to perform the behaviour	Information about emotinal consequences	Social comparison	Behavioural practice/rehearsal
0	0	Feedback on behaviour	Information about health consequences	Information abour social & environmental consequences	Information about emotinal consequences	Social comparison
0	0	Feedback on behaviour	Information about health consequences	Information abour social & environmental consequences	Information about emotinal consequences	Behavioural practice/rehearsal
0	0	Feedback on behaviour	Information about health consequences	Information abour social & environmental consequences	Social comparison	Behavioural practice/rehearsal
0	0	Feedback on behaviour	Information about health consequences	Information about emotinal consequences	Social comparison	Behavioural practice/rehearsal
0	0	Feedback on behaviour	Information abour social & environmental consequences	Information about emotinal consequences	Social comparison	Behavioural practice/rehearsal
0	0	self monitoring of behaviour	social support	Instruction on how to perform the behaviour	Information abour social & environmental consequences	Information about emotinal consequences
0	0	Feedback on behaviour	social support	Information about emotinal consequences	Social comparison	Behavioural practice/rehearsal
0	0	Feedback on behaviour	Instruction on how to perform the behaviour	Information about health consequences	Information abour social & environmental consequences	Information about emotinal consequences
0	0	Feedback on behaviour	Instruction on how to perform the behaviour	Information about health consequences	Information abour social & environmental consequences	Social comparison
0	0	Feedback on behaviour	Instruction on how to perform the behaviour	Information about health consequences	Information abour social & environmental consequences	Behavioural practice/rehearsal
0	0	Feedback on behaviour	Instruction on how to perform the behaviour	Information about health consequences	Information abour social & environmental consequences	Adding object to the environment
0	0	Feedback on behaviour	Instruction on how to perform the behaviour	Information about health consequences	Information about emotinal consequences	Social comparison
0	0	Feedback on behaviour	Instruction on how to perform the behaviour	Information about health consequences	Information about emotinal consequences	Behavioural practice/rehearsal
0	0	Feedback on behaviour	Instruction on how to perform the behaviour	Information abour social & environmental consequences	Information about emotinal consequences	Social comparison
0	0	Feedback on behaviour	social support	Information about health consequences	Information abour social & environmental consequences	Information about emotinal consequences
0	0	Feedback on behaviour	social support	Information about health consequences	Information abour social & environmental consequences	Social comparison
0	0	Feedback on behaviour	social support	Information about health consequences	Information abour social & environmental consequences	Behavioural practice/rehearsal
0	0	Feedback on behaviour	social support	Information about health consequences	Information abour social & environmental consequences	Adding object to the environment
0	0	Feedback on behaviour	social support	Information about health consequences	Information about emotinal consequences	Social comparison
0	0	Feedback on behaviour	social support	Information about health consequences	Information about emotinal consequences	Behavioural practice/rehearsal
0	0	Feedback on behaviour	social support	Information abour social & environmental consequences	Information about emotinal consequences	Social comparison
0	0	Feedback on behaviour	social support	Information abour social & environmental consequences	Information about emotinal consequences	Behavioural practice/rehearsal
0	0	self monitoring of behaviour	Information about health consequences	Information abour social & environmental consequences	Information about emotinal consequences	Behavioural practice/rehearsal
0	0	self monitoring of behaviour	Information about health consequences	Information abour social & environmental consequences	Social comparison	Behavioural practice/rehearsal
0	0	self monitoring of behaviour	Information about health consequences	Information about emotinal consequences	Social comparison	Behavioural practice/rehearsal
0	0	self monitoring of behaviour	Information abour social & environmental consequences	Information about emotinal consequences	Social comparison	Behavioural practice/rehearsal
2	2	Goal setting (behaviour)	Problem solving	Goal setting (outcome)	Action planning	Feedback on behaviour

0	0	Problem solving	self monitoring of behaviour	Instruction on how to perform the behaviour	Information about health consequences	Information about social & environmental consequences	Information about emotinal consequences
0	0	Problem solving	self monitoring of behaviour	Instruction on how to perform the behaviour	Information about health consequences	Information about social & environmental consequences	Social comparison
0	0	Problem solving	self monitoring of behaviour	Instruction on how to perform the behaviour	Information about health consequences	Information about emotinal consequences	Social comparison
0	0	Problem solving	self monitoring of behaviour	Instruction on how to perform the behaviour	Information about health consequences	Information about emotinal consequences	Behavioural practice/rehearsal
0	0	Problem solving	self monitoring of behaviour	Instruction on how to perform the behaviour	Information about social & environmental consequences	Information about emotinal consequences	Social comparison
0	0	Problem solving	self monitoring of behaviour	Instruction on how to perform the behaviour	Information about social & environmental consequences	Information about emotinal consequences	Behavioural practice/rehearsal
0	0	Problem solving	self monitoring of behaviour	social support	Instruction on how to perform the behaviour	Information about emotinal consequences	Social comparison
0	0	Problem solving	self monitoring of behaviour	social support	Instruction on how to perform the behaviour	Information about emotinal consequences	Behavioural practice/rehearsal
0	0	Problem solving	self monitoring of behaviour	social support	Information about health consequences	Information about social & environmental consequences	Information about emotinal consequences
0	0	Problem solving	self monitoring of behaviour	social support	Information about health consequences	Information about social & environmental consequences	Social comparison
0	0	Problem solving	self monitoring of behaviour	social support	Information about health consequences	Information about emotinal consequences	Social comparison
0	0	Problem solving	self monitoring of behaviour	social support	Information about health consequences	Information about emotinal consequences	Behavioural practice/rehearsal
0	0	Problem solving	self monitoring of behaviour	social support	Information about social & environmental consequences	Information about emotinal consequences	Social comparison
0	0	Problem solving	self monitoring of behaviour	social support	Information about social & environmental consequences	Information about emotinal consequences	Behavioural practice/rehearsal
0	0	Problem solving	Feedback on behaviour	Information about health consequences	Information about social & environmental consequences	Information about emotinal consequences	Social comparison
0	0	Problem solving	Feedback on behaviour	Information about health consequences	Information about social & environmental consequences	Information about emotinal consequences	Behavioural practice/rehearsal
0	0	Problem solving	Feedback on behaviour	Information about health consequences	Information about social & environmental consequences	Social comparison	Behavioural practice/rehearsal
0	0	Problem solving	Feedback on behaviour	Information about health consequences	Information about emotinal consequences	Social comparison	Behavioural practice/rehearsal
0	0	Problem solving	Feedback on behaviour	Information about social & environmental consequences	Information about emotinal consequences	Social comparison	Behavioural practice/rehearsal
0	0	Problem solving	self monitoring of behaviour	social support	Instruction on how to perform the behaviour	Information about health consequences	Information about emotinal consequences
0	0	Problem solving	self monitoring of behaviour	social support	Instruction on how to perform the behaviour	Information about social & environmental consequences	Information about emotinal consequences
0	0	Problem solving	self monitoring of behaviour	social support	Instruction on how to perform the behaviour	Information about social & environmental consequences	Social comparison
0	0	Problem solving	Feedback on behaviour	Instruction on how to perform the behaviour	Information about health consequences	Information about social & environmental consequences	Behavioural practice/rehearsal
0	0	Problem solving	Feedback on behaviour	Instruction on how to perform the behaviour	Information about health consequences	Information about social & environmental consequences	Adding object to the environment
0	0	Problem solving	Feedback on behaviour	Instruction on how to perform the behaviour	Information about health consequences	Information about emotinal consequences	Social comparison
0	0	Problem solving	Feedback on behaviour	Instruction on how to perform the behaviour	Information about health consequences	Information about emotinal consequences	Behavioural practice/rehearsal
0	0	Problem solving	Feedback on behaviour	Instruction on how to perform the behaviour	Information about social & environmental consequences	Information about emotinal consequences	Social comparison
0	0	Problem solving	Feedback on behaviour	Instruction on how to perform the behaviour	Information about social & environmental consequences	Information about emotinal consequences	Behavioural practice/rehearsal
0	0	Problem solving	Feedback on behaviour	Instruction on how to perform the behaviour	Information about social & environmental consequences	Social comparison	Behavioural practice/rehearsal
0	0	Problem solving	Feedback on behaviour	Instruction on how to perform the behaviour	Information about emotinal consequences	Social comparison	Behavioural practice/rehearsal
0	0	Action planning	Feedback on behaviour	self monitoring of behaviour	Instruction on how to perform the behaviour	Behavioural practice/rehearsal	Adding object to the environment
0	0	Action planning	Feedback on behaviour	self monitoring of behaviour	Information about health consequences	Information about social & environmental consequences	Information about emotinal consequences
[illegible]	[illegible]	[illegible]	[illegible]	[illegible]	[illegible]	[illegible]	[illegible]

0	0	Goal setting (behaviour)	Action planning	Feedback on behaviour	social support	Instruction on how to perform the behaviour	Behavioural practice/rehearsal	Adding object to the environment	
0	0	Goal setting (behaviour)	Feedback on behaviour	self monitoring of behaviour	social support	Instruction on how to perform the behaviour	Information about emotinal consequences	Behavioural practice/rehearsal	
0	0	Goal setting (behaviour)	Feedback on behaviour	self monitoring of behaviour	social support	Instruction on how to perform the behaviour	Behavioural practice/rehearsal	Adding object to the environment	
0	0	Goal setting (behaviour)	Feedback on behaviour	self monitoring of behaviour	social support	Information about health consequences	Information abour social & environmental consequences	Information about emotinal consequences	
0	0	Goal setting (behaviour)	Feedback on behaviour	self monitoring of behaviour	social support	Information about health consequences	Information abour social & environmental consequences	Social comparison	
0	0	Goal setting (behaviour)	Feedback on behaviour	self monitoring of behaviour	social support	Information about health consequences	Information abour social & environmental consequences	Behavioural practice/rehearsal	
0	0	Goal setting (behaviour)	Feedback on behaviour	self monitoring of behaviour	social support	Information about health consequences	Information abour social & environmental consequences	Adding object to the environment	
0	0	Goal setting (behaviour)	Feedback on behaviour	self monitoring of behaviour	social support	Information about health consequences	Information about emotinal consequences	Social comparison	
0	0	Goal setting (behaviour)	Feedback on behaviour	self monitoring of behaviour	social support	Information about health consequences	Information about emotinal consequences	Behavioural practice/rehearsal	
0	0	Goal setting (behaviour)	Action planning	self monitoring of behaviour	Information about health consequences	Information abour social & environmental consequences	Information about emotinal consequences	Behavioural practice/rehearsal	
0	0	Goal setting (behaviour)	Action planning	self monitoring of behaviour	Information about health consequences	Information abour social & environmental consequences	Social comparison	Behavioural practice/rehearsal	
0	0	Goal setting (behaviour)	Action planning	self monitoring of behaviour	Information about health consequences	Information about emotinal consequences	Social comparison	Behavioural practice/rehearsal	
0	0	Goal setting (behaviour)	Action planning	self monitoring of behaviour	Information abour social & environmental consequences	Information about emotinal consequences	Social comparison	Behavioural practice/rehearsal	
0	0	Goal setting (behaviour)	Feedback on behaviour	self monitoring of behaviour	social support	Instruction on how to perform the behaviour	Information about health consequences	Information about emotinal consequences	
0	0	Goal setting (behaviour)	Feedback on behaviour	self monitoring of behaviour	social support	Instruction on how to perform the behaviour	Information abour social & environmental consequences	Information about emotinal consequences	
0	0	Goal setting (behaviour)	Feedback on behaviour	self monitoring of behaviour	social support	Instruction on how to perform the behaviour	Information abour social & environmental consequences	Adding object to the environment	
0	0	Goal setting (behaviour)	Feedback on behaviour	self monitoring of behaviour	social support	Instruction on how to perform the behaviour	Information about emotinal consequences	Social comparison	
0	0	Goal setting (behaviour)	Action planning	self monitoring of behaviour	Instruction on how to perform the behaviour	Information about health consequences	Information abour social & environmental consequences	Information about emotinal consequences	
0	0	Goal setting (behaviour)	Action planning	self monitoring of behaviour	Instruction on how to perform the behaviour	Information about health consequences	Information abour social & environmental consequences	Social comparison	
0	0	Goal setting (behaviour)	Action planning	self monitoring of behaviour	Instruction on how to perform the behaviour	Information about health consequences	Information about emotinal consequences	Social comparison	
0	0	Goal setting (behaviour)	Action planning	self monitoring of behaviour	Instruction on how to perform the behaviour	Information about health consequences	Information about emotinal consequences	Behavioural practice/rehearsal	
0	0	Goal setting (behaviour)	Action planning	self monitoring of behaviour	Instruction on how to perform the behaviour	Information abour social & environmental consequences	Information about emotinal consequences	Social comparison	
0	0	Goal setting (behaviour)	Action planning	self monitoring of behaviour	Instruction on how to perform the behaviour	Information abour social & environmental consequences	Information about emotinal consequences	Behavioural practice/rehearsal	
0	0	Goal setting (behaviour)	Action planning	self monitoring of behaviour	Instruction on how to perform the behaviour	Information about emotinal consequences	Social comparison	Behavioural practice/rehearsal	
0	0	Goal setting (behaviour)	Action planning	self monitoring of behaviour	Information about health consequences	Information abour social & environmental consequences	Information about emotinal consequences	Social comparison	
0	0	Goal setting (behaviour)	Action planning	self monitoring of behaviour	social support	Instruction on how to perform the behaviour	Information about emotinal consequences	Behavioural practice/rehearsal	
0	0	Goal setting (behaviour)	Action planning	self monitoring of behaviour	social support	Information about health consequences	Information abour social & environmental consequences	Information about emotinal consequences	
0	0	Goal setting (behaviour)	Action planning	self monitoring of behaviour	social support	Information about health consequences	Information abour social & environmental consequences	Social comparison	
0	0	Goal setting (behaviour)	Action planning	self monitoring of behaviour	social support	Information about health consequences	Information about emotinal consequences	Social comparison	
0	0	Goal setting (behaviour)	Action planning	self monitoring of behaviour	social support	Information about health consequences	Information about emotinal consequences	Behavioural practice/rehearsal	
0	0	Goal setting (behaviour)	Action planning	self monitoring of behaviour	social support	Information abour social & environmental consequences	Information about emotinal consequences	Social comparison	
0	0	Goal setting (behaviour)	Action planning	self monitoring of behaviour	social support	Information abour social & environmental consequences	Information about emotinal consequences	Behavioural practice/rehearsal	
0	0	Goal setting (behaviour)	Action planning	self monitoring of behaviour	social support	Information about emotinal consequences	Social comparison	Behavioural practice/rehearsal	
0	0	Goal setting (behaviour)	Feedback on behaviour	social support	Instruction on how to perform the behaviour	Information abour social & environmental consequences	Information about emotinal consequences	Behavioural practice/rehearsal	
0	0	Goal setting (behaviour)	Feedback on behaviour	social support	Instruction on how to perform the behaviour	Information about emotinal consequences	Social comparison	Behavioural practice/rehearsal	
0	0	Goal setting (behaviour)	Feedback on behaviour	social support	Information about health consequences	Information abour social & environmental consequences	Information about emotinal consequences	Social comparison	
0	0	Goal setting (behaviour)	Feedback on behaviour	social support	Information about health consequences	Information abour social & environmental consequences	Information about emotinal consequences	Behavioural practice/rehearsal	
0	0	Goal setting (behaviour)	Feedback on behaviour	social support	Information about health consequences	Information abour social & environmental consequences	Social comparison	Behavioural practice/rehearsal	
0	0	Goal setting (behaviour)	Feedback on behaviour	social support	Information about health consequences	Information about emotinal consequences	Social comparison	Behavioural practice/rehearsal	
0	0	Goal setting (behaviour)	Feedback on behaviour	social support	Information abour social & environmental consequences	Information about emotinal consequences	Social comparison	Behavioural practice/rehearsal	
0	0	Goal setting (behaviour)	Feedback on behaviour	Instruction on how to perform the behaviour	Information about health consequences	Information abour social & environmental consequences	Information about emotinal consequences	Social comparison	
0	0	Goal setting (behaviour)	Feedback on behaviour	self monitoring of behaviour	Information abour social & environmental consequences	Information about emotinal consequences	Social comparison	Behavioural practice/rehearsal	
0	0	Goal setting (behaviour)	Feedback on behaviour	social support	Instruction on how to perform the behaviour	Information about health consequences	Information abour social & environmental consequences	Information about emotinal consequences	
0	0	Goal setting (behaviour)	Feedback on behaviour	social support	Instruction on how to perform the behaviour	Information about health consequences	Information abour social & environmental consequences	Social comparison	
0	0	Goal setting (behaviour)	Feedback on behaviour	social support	Instruction on how to perform the behaviour	Information about health consequences	Information abour social & environmental consequences	Behavioural practice/rehearsal	
0	0	Goal setting (behaviour)	Feedback on behaviour	social support	Instruction on how to perform the behaviour	Information about health consequences	Information abour social & environmental consequences	Adding object to the environment	
0	0	Goal setting (behaviour)	Feedback on behaviour	social support	Instruction on how to perform the behaviour	Information about health consequences	Information about emotinal consequences	Social comparison	
0	0	Goal setting (behaviour)	Feedback on behaviour	social support	Instruction on how to perform the behaviour	Information about health consequences	Information about emotinal consequences	Behavioural practice/rehearsal	
0	0	Goal setting (behaviour)	Feedback on behaviour	social support	Instruction on how to perform the behaviour	Information abour social & environmental consequences	Information about emotinal consequences	Social comparison	
0	0	Goal setting (behaviour)	Feedback on behaviour	self monitoring of behaviour	Instruction on how to perform the behaviour	Information about health consequences	Information about emotinal consequences	Behavioural practice/rehearsal	
0	0	Goal setting (behaviour)	Feedback on behaviour	self monitoring of behaviour	Instruction on how to perform the behaviour	Information abour social & environmental consequences	Information about emotinal consequences	Social comparison	
0	0	Goal setting (behaviour)	Feedback on behaviour	self monitoring of behaviour	Instruction on how to perform the behaviour	Information abour social & environmental consequences	Information about emotinal consequences	Behavioural practice/rehearsal	
0	0	Goal setting (behaviour)	Feedback on behaviour	self monitoring of behaviour	Instruction on how to perform the behaviour	Information about emotinal consequences	Social comparison	Behavioural practice/rehearsal	
0	0	Goal setting (behaviour)	Feedback on behaviour	self monitoring of behaviour	Information about health consequences	Information abour social & environmental consequences	Information about emotinal consequences	Social comparison	
0	0	Goal setting (behaviour)	Feedback on behaviour	self monitoring of behaviour	Information about health consequences	Information abour social & environmental consequences	Information about emotinal consequences	Behavioural practice/rehearsal	
0	0	Goal setting (behaviour)	Feedback on behaviour	self monitoring of behaviour	Information about health consequences	Information abour social & environmental consequences	Social comparison	Behavioural practice/rehearsal	
0	0	Goal setting (behaviour)	Feedback on behaviour	self monitoring of behaviour	Information about health consequences	Information about emotinal consequences	Social comparison	Behavioural practice/rehearsal	
0	0	Goal setting (behaviour)	Feedback on behaviour	self monitoring of behaviour	social support	Information abour social & environmental consequences	Information about emotinal consequences	Social comparison	
0	0	Goal setting (behaviour)	Feedback on behaviour	self monitoring of behaviour	social support	Information abour social & environmental consequences	Information about emotinal consequences	Behavioural practice/rehearsal	
0	0	Goal setting (behaviour)	Feedback on behaviour	self monitoring of behaviour	social support	Information about emotinal consequences	Social comparison	Behavioural practice/rehearsal	
0	0	Goal setting (behaviour)	Feedback on behaviour	self monitoring of behaviour	Instruction on how to perform the behaviour	Information about health consequences	Information abour social & environmental consequences	Information about emotinal consequences	
0	0	Goal setting (behaviour)	Feedback on behaviour	self monitoring of behaviour	Instruction on how to perform the behaviour	Information about health consequences	Information abour social & environmental consequences	Social comparison	
0	0	Goal setting (behaviour)	Feedback on behaviour	self monitoring of behaviour	Instruction on how to perform the behaviour	Information about health consequences	Information abour social & environmental consequences	Behavioural practice/rehearsal	
0	0	Goal setting (behaviour)	Feedback on behaviour	self monitoring of behaviour	Instruction on how to perform the behaviour	Information about health consequences	Information abour social & environmental consequences	Adding object to the environment	
0	0	Goal setting (behaviour)	Feedback on behaviour	self monitoring of behaviour	Instruction on how to perform the behaviour	Information about health consequences	Information about emotinal consequences	Social comparison	
2	2	Problem solving	Goal setting (outcome)	Action planning	Feedback on behaviour	self monitoring of behaviour	social support	Adding object to the environment	
2	2	Problem solving	Goal setting (outcome)	Action planning	Feedback on behaviour	self monitoring of behaviour	Instruction on how to perform the behaviour	Adding object to the environment	
2	2	Problem solving	Goal setting (outcome)	Action planning	Feedback on behaviour	social support	Instruction on how to perform the behaviour	Adding object to the environment	
2	2	Problem solving	Goal setting (outcome)	Feedback on behaviour	self monitoring of behaviour	social support	Instruction on how to perform the behaviour	Adding object to the environment	
0	0	Problem solving	Action planning	Feedback on behaviour	self monitoring of behaviour	social support	Instruction on how to perform the behaviour	Information abour social & environmental consequences	
0	0	Problem solving	Action planning	Feedback on behaviour	self monitoring of behaviour	social support	Instruction on how to perform the behaviour	Information about emotinal consequences	
0	0	Problem solving	Action planning	Feedback on behaviour	self monitoring of behaviour	social support	Information about health consequences	Information abour social & environmental consequences	
0	0	Problem solving	Action planning	Feedback on behaviour	self monitoring of behaviour	social support	Information about health consequences	Information about emotinal consequences	
0	0	Goal setting (behaviour)	self monitoring of behaviour	social support	Information abour social & environmental consequences	Information about emotinal consequences	Social comparison	Behavioural practice/rehearsal	
0	0	Goal setting (behaviour)	self monitoring of behaviour	Instruction on how to perform the behaviour	Information about health consequences	Information abour social & environmental consequences	Information about emotinal consequences	Social comparison	
0	0	Goal setting (behaviour)	self monitoring of behaviour	Instruction on how to perform the behaviour	Information about health consequences	Information abour social & environmental consequences	Information about emotinal consequences	Behavioural practice/rehearsal	
0	0	Goal setting (behaviour)	self monitoring of behaviour	Instruction on how to perform the behaviour	Information about health consequences	Information abour social & environmental consequences	Social comparison	Behavioural practice/rehearsal	
0	0	Goal setting (behaviour)	self monitoring of behaviour	Instruction on how to perform the behaviour	Information about health consequences	Information about emotinal consequences	Social comparison	Behavioural practice/rehearsal	
0	0	Goal setting (behaviour)	self monitoring of behaviour	Instruction on how to perform the behaviour	Information abour social & environmental consequences	Information about emotinal consequences	Social comparison	Behavioural practice/rehearsal	
0	0	Goal setting (behaviour)	self monitoring of behaviour	Information about health consequences	Information abour social & environmental consequences	Information about emotinal consequences	Social comparison	Behavioural practice/rehearsal	
2	2	Problem solving	Goal setting (outcome)	Action planning	Feedback on behaviour	self monitoring of behaviour	social support	Instruction on how to perform the behaviour	
0	0	Goal setting (behaviour)	self monitoring of behaviour	social support	Instruction on how to perform the behaviour	Information about health consequences	Information about emotinal consequences	Behavioural practice/rehearsal	
0	0	Goal setting (behaviour)	self monitoring of behaviour	social support	Instruction on how to perform the behaviour	Information abour social & environmental consequences	Information about emotinal consequences	Social comparison	
0	0	Goal setting (behaviour)	self monitoring of behaviour	social support	Instruction on how to perform the behaviour	Information abour social & environmental consequences	Information about emotinal consequences	Behavioural practice/rehearsal	
0	0	Goal setting (behaviour)	self monitoring of behaviour	social support	Instruction on how to perform the behaviour	Information about emotinal consequences	Social comparison	Behavioural practice/rehearsal	
0	0	Goal setting (behaviour)	self monitoring of behaviour	social support	Information about health consequences	Information abour social & environmental consequences	Information about emotinal consequences	Social comparison	
0	0	Goal setting (behaviour)	self monitoring of behaviour	social support	Information about health consequences	Information abour social & environmental consequences	Information about emotinal consequences	Behavioural practice/rehearsal	
0	0	Goal setting (behaviour)	self monitoring of behaviour	social support	Information about health consequences	Information abour social & environmental consequences	Social comparison	Behavioural practice/rehearsal	
0	0	Goal setting (behaviour)	self monitoring of behaviour	social support	Information about health consequences	Information about emotinal consequences	Social comparison	Behavioural practice/rehearsal	
0	0	Goal setting (behaviour)	Feedback on behaviour	Instruction on how to perform the behaviour	Information about health consequences	Information abour social & environmental consequences	Information about emotinal consequences	Behavioural practice/rehearsal	
0	0	Goal setting (behaviour)	Feedback on behaviour	Instruction on how to perform the behaviour	Information about health consequences	Information abour social & environmental consequences	Social comparison	Behavioural practice/rehearsal	
0	0	Goal setting (behaviour)	Feedback on behaviour	Instruction on how to perform the behaviour	Information about health consequences	Information about emotinal consequences	Social comparison	Behavioural practice/rehearsal	
0	0	Goal setting (behaviour)	Feedback on behaviour	Instruction on how to perform the behaviour	Information abour social & environmental consequences	Information about emotinal consequences	Social comparison	Behavioural practice/rehearsal	
0	0	Goal setting (behaviour)	Feedback on behaviour	Information about health consequences	Information abour social & environmental consequences	Information about emotinal consequences	Social comparison	Behavioural practice/rehearsal	
0	0	Goal setting (behaviour)	self monitoring of behaviour	social support	Instruction on how to perform the behaviour	Information about health consequences	Information abour social & environmental consequences	Information about emotinal consequences	
0	0	Goal setting (behaviour)	self monitoring of behaviour	social support	Instruction on how to perform the behaviour	Information about health consequences	Information abour social & environmental consequences	Social comparison	
0	0	Goal setting (behaviour)	self monitoring of behaviour	social support	Instruction on how to perform the behaviour	Information about health consequences	Information about emotinal consequences	Social comparison	
0	0	Problem solving	Action planning	Feedback on behaviour	social support	Instruction on how to perform the behaviour	Information about health consequences	Information about emotinal consequences	
0	0	Problem solving	Action planning	Feedback on behaviour	social support	Instruction on how to perform the behaviour	Information abour social & environmental consequences	Information about emotinal consequences	
0	0	Problem solving	Action planning	Feedback on behaviour	social support	Instruction on how to perform the behaviour	Information abour social & environmental consequences	Social comparison	
0	0	Problem solving	Action planning	Feedback on behaviour	social support	Instruction on how to perform the behaviour	Information abour social & environmental consequences	Behavioural practice/rehearsal	
0	0	Problem solving	Action planning	Feedback on behaviour	social support	Instruction on how to perform the behaviour	Information about emotinal consequences	Social comparison	
0	0	Problem solving	Action planning	Feedback on behaviour	social support	Instruction on how to perform the behaviour	Information about emotinal consequences	Behavioural practice/rehearsal	
0	0	Problem solving	Action planning	Feedback on behaviour	social support	Instruction on how to perform the behaviour	Behavioural practice/rehearsal	Adding object to the environment	
0	0	Problem solving	Action planning	Feedback on behaviour	social support	Information about health consequences	Information abour social & environmental consequences	Information about emotinal consequences	
0	0	Problem solving	Action planning	Feedback on behaviour	self monitoring of behaviour	Information about health consequences	Information abour social & environmental consequences	Behavioural practice/rehearsal	
0	0	Problem solving	Action planning	Feedback on behaviour	self monitoring of behaviour	Information about health consequences	Information about emotinal consequences	Social comparison	
0	0	Problem solving	Action planning	Feedback on behaviour	self monitoring of behaviour	Information about health consequences	Information about emotinal consequences	Behavioural practice/rehearsal	
0	0	Problem solving	Action planning	Feedback on behaviour	self monitoring of behaviour	Information abour social & environmental consequences	Information about emotinal consequences	Social comparison	
0	0	Problem solving	Action planning	Feedback on behaviour	self monitoring of behaviour	Information abour social & environmental consequences	Information about emotinal consequences	Behavioural practice/rehearsal	
0	0	Problem solving	Action planning	Feedback on behaviour	self monitoring of behaviour	Information abour social & environmental consequences	Social comparison	Behavioural practice/rehearsal	
0	0	Problem solving	Action planning	Feedback on behaviour	self monitoring of behaviour	Information about emotinal consequences	Social comparison	Behavioural practice/rehearsal	
0	0	Problem solving	Action planning	Feedback on behaviour	social support	Instruction on how to perform the behaviour	Information about health consequences	Information abour social & environmental consequences	
0	0	Problem solving	Action planning	Feedback on behaviour	self monitoring of behaviour	Instruction on how to perform the behaviour	Information abour social & environmental consequences	Information about emotinal consequences	
0	0	Problem solving	Action planning	Feedback on behaviour	self monitoring of behaviour	Instruction on how to perform the behaviour	Information abour social & environmental consequences	Social comparison	
0	0	Problem solving	Action planning	Feedback on behaviour	self monitoring of behaviour	Instruction on how to perform the behaviour	Information abour social & environmental consequences	Behavioural practice/rehearsal	
0	0	Problem solving	Action planning	Feedback on behaviour	self monitoring of behaviour	Instruction on how to perform the behaviour	Information about emotinal consequences	Social comparison	
0	0	Problem solving	Action planning	Feedback on behaviour	self monitoring of behaviour	Instruction on how to perform the behaviour	Information about emotinal consequences	Behavioural practice/rehearsal	
0	0	Problem solving	Action planning	Feedback on behaviour	self monitoring of behaviour	Instruction on how to perform the behaviour	Behavioural practice/rehearsal	Adding object to the environment	
0	0	Problem solving	Action planning	Feedback on behaviour	self monitoring of behaviour	Information about health consequences	Information abour social & environmental consequences	Information about emotinal consequences	
0	0	Problem solving	Action planning	Feedback on behaviour	self monitoring of behaviour	Information about health consequences	Information abour social & environmental consequences	Social comparison	
0	0	Problem solving	Action planning	Feedback on behaviour	self monitoring of behaviour	social support	Information abour social & environmental consequences	Information about emotinal consequences	
0	0	Problem solving	Action planning	Feedback on behaviour	self monitoring of behaviour	social support	Information abour social & environmental consequences	Social comparison	
0	0	Problem solving	Action planning	Feedback on behaviour	self monitoring of behaviour	social support	Information abour social & environmental consequences	Behavioural practice/rehearsal	
0	0	Problem solving	Action planning	Feedback on behaviour	self monitoring of behaviour	social support	Information about emotinal consequences	Social comparison	
0	0	Problem solving	Action planning	Feedback on behaviour	self monitoring of behaviour	social support	Information about emotinal consequences	Behavioural practice/rehearsal	
0	0	Problem solving	Action planning	Feedback on behaviour	self monitoring of behaviour	social support	Behavioural practice/rehearsal	Adding object to the environment	
0	0	Problem solving	Action planning	Feedback on behaviour	self monitoring of behaviour	Instruction on how to perform the behaviour	Information about health consequences	Information abour social & environmental consequences	
0	0	Problem solving	Action planning	Feedback on behaviour	self monitoring of behaviour	Instruction on how to perform the behaviour	Information about health consequences	Information about emotinal consequences	
0	0	Problem solving	Action planning	self monitoring of behaviour	social support	Instruction on how to perform the behaviour	Information abour social & environmental consequences	Social comparison	
0	0	Problem solving	Action planning	self monitoring of behaviour	social support	Instruction on how to perform the behaviour	Information about emotinal consequences	Social comparison	
0	0	Problem solving	Action planning	self monitoring of behaviour	social support	Instruction on how to perform the behaviour	Information about emotinal consequences	Behavioural practice/rehearsal	
0	0	Problem solving	Action planning	self monitoring of behaviour	social support	Information about health consequences	Information abour social & environmental consequences	Information about emotinal consequences	
0	0	Problem solving	Action planning	self monitoring of behaviour	social support	Information about health consequences	Information abour social & environmental consequences	Social comparison	
0	0	Problem solving	Action planning	self monitoring of behaviour	social support	Information about health consequences	Information about emotinal consequences	Social comparison	
0	0	Problem solving	Action planning	self monitoring of behaviour	social support	Information about health consequences	Information about emotinal consequences	Behavioural practice/rehearsal	
0	0	Problem solving	Action planning	self monitoring of behaviour	social support	Information abour social & environmental consequences	Information about emotinal consequences	Social comparison	
0	0	Problem solving	Action planning	Feedback on behaviour	Instruction on how to perform the behaviour	Information about emotinal consequences	Social comparison	Behavioural practice/rehearsal	
0	0	Problem solving	Action planning	Feedback on behaviour	Information about health consequences	Information abour social & environmental consequences	Information about emotinal consequences	Social comparison	
0	0	Problem solving	Action planning	Feedback on behaviour	Information about health consequences	Information abour social & environmental consequences	Information about emotinal consequences	Behavioural practice/rehearsal	
0	0	Problem solving	Action planning	Feedback on behaviour	Information about health consequences	Information abour social & environmental consequences	Social comparison	Behavioural practice/rehearsal	
0	0	Problem solving	Action planning	Feedback on behaviour	Information about health consequences	Information about emotinal consequences	Social comparison	Behavioural practice/rehearsal	
0	0	Problem solving	Action planning	Feedback on behaviour	Information abour social & environmental consequences	Information about emotinal consequences	Social comparison	Behavioural practice/rehearsal	
0	0	Problem solving	Action planning	self monitoring of behaviour	social support	Instruction on how to perform the behaviour	Information about health consequences	Information about emotinal consequences	
0	0	Problem solving	Action planning	self monitoring of behaviour	social support	Instruction on how to perform the behaviour	Information abour social & environmental consequences	Information about emotinal consequences	
0	0	Problem solving	Action planning	Feedback on behaviour	Instruction on how to perform the behaviour	Information about health consequences	Information abour social & environmental consequences	Information about emotinal consequences	
0	0	Problem solving	Action planning	Feedback on behaviour	Instruction on how to perform the behaviour	Information about health consequences	Information abour social & environmental consequences	Social comparison	
0	0	Problem solving	Action planning	Feedback on behaviour	Instruction on how to perform the behaviour	Information about health consequences	Information abour social & environmental consequences	Behavioural practice/rehearsal	
0	0	Problem solving	Action planning	Feedback on behaviour	Instruction on how to perform the behaviour	Information about health consequences	Information about emotinal consequences	Social comparison	
0	0	Problem solving	Action planning	Feedback on behaviour	Instruction on how to perform the behaviour	Information about health consequences	Information about emotinal consequences	Behavioural practice/rehearsal	
0	0	Problem solving	Action planning	Feedback on behaviour	Instruction on how to perform the behaviour	Information abour social & environmental consequences	Information about emotinal consequences	Social comparison	
0	0	Problem solving	Action planning	Feedback on behaviour	Instruction on how to perform the behaviour	Information abour social & environmental consequences	Information about emotinal consequences	Behavioural practice/rehearsal	
0	0	Problem solving	Action planning	Feedback on behaviour	Instruction on how to perform the behaviour	Information abour social & environmental consequences	Social comparison	Behavioural practice/rehearsal	
0	0	Problem solving	Action planning	Feedback on behaviour	social support	Information about health consequences	Information abour social & environmental consequences	Social comparison	
0	0	Problem solving	Action planning	Feedback on behaviour	social support	Information about health consequences	Information abour social & environmental consequences	Behavioural practice/rehearsal	
0	0	Problem solving	Action planning	Feedback on behaviour	social support	Information about health consequences	Information about emotinal consequences	Social comparison	
0	0	Problem solving	Action planning	Feedback on behaviour	social support	Information about health consequences	Information about emotinal consequences	Behavioural practice/rehearsal	
0	0	Problem solving	Action planning	Feedback on behaviour	social support	Information abour social & environmental consequences	Information about emotinal consequences	Social comparison	
0	0	Problem solving	Action planning	Feedback on behaviour	social support	Information abour social & environmental consequences	Information about emotinal consequences	Behavioural practice/rehearsal	
0	0	Problem solving	Action planning	Feedback on behaviour	social support	Information abour social & environmental consequences	Social comparison	Behavioural practice/rehearsal	
0	0	Problem solving	Action planning	Feedback on behaviour	social support	Information about emotinal consequences	Social comparison	Behavioural practice/rehearsal	
0	0	Problem solving	Feedback on behaviour	self monitoring of behaviour	social support	Information about health consequences	Information abour social & environmental consequences	Information about emotinal consequences	
0	0	Problem solving	Feedback on behaviour	self monitoring of behaviour	social support	Information about health consequences	Information abour social & environmental consequences	Social comparison	
0	0	Problem solving	Feedback on behaviour	self monitoring of behaviour	social support	Information about health consequences	Information abour social & environmental consequences	Behavioural practice/rehearsal	
0	0	Problem solving	Feedback on behaviour	self monitoring of behaviour	social support	Information about health consequences	Information abour social & environmental consequences	Adding object to the environment	
0	0	Problem solving	Feedback on behaviour	self monitoring of behaviour	social support	Information about health consequences	Information about emotinal consequences	Social comparison	
0	0	Problem solving	Feedback on behaviour	self monitoring of behaviour	social support	Information about health consequences	Information about emotinal consequences	Behavioural practice/rehearsal	
0	0	Problem solving	Feedback on behaviour	self monitoring of behaviour	social support	Information abour social & environmental consequences	Information about emotinal consequences	Social comparison	
0	0	Problem solving	Feedback on behaviour	self monitoring of behaviour	social support	Information abour social & environmental consequences	Information about emotinal consequences	Behavioural practice/rehearsal	
0	0	Problem solving	Feedback on behaviour	self monitoring of behaviour	social support	Instruction on how to perform the behaviour	Information about health consequences	Information about emotinal consequences	
0	0	Problem solving	Feedback on behaviour	self monitoring of behaviour	social support	Instruction on how to perform the behaviour	Information abour social & environmental consequences	Information about emotinal consequences	
0	0	Problem solving	Feedback on behaviour	self monitoring of behaviour	social support	Instruction on how to perform the behaviour	Information abour social & environmental consequences	Social comparison	
0	0	Problem solving	Feedback on behaviour	self monitoring of behaviour	social support	Instruction on how to perform the behaviour	Information abour social & environmental consequences	Behavioural practice/rehearsal	
0	0	Problem solving	Feedback on behaviour	self monitoring of behaviour	social support	Instruction on how to perform the behaviour	Information abour social & environmental consequences	Adding object to the environment	
0	0	Problem solving	Feedback on behaviour	self monitoring of behaviour	social support	Instruction on how to perform the behaviour	Information about emotinal consequences	Social comparison	
0	0	Problem solving	Feedback on behaviour	self monitoring of behaviour	social support	Instruction on how to perform the behaviour	Information about emotinal consequences	Behavioural practice/rehearsal	
0	0	Problem solving	Feedback on behaviour	self monitoring of behaviour	social support	Instruction on how to perform the behaviour	Behavioural practice/rehearsal	Adding object to the environment	
0	0	Problem solving	Action planning	self monitoring of behaviour	Instruction on how to perform the behaviour	Information abour social & environmental consequences	Information about emotinal consequences	Behavioural practice/rehearsal	
0	0	Problem solving	Action planning	self monitoring of behaviour	Instruction on how to perform the behaviour	Information abour social & environmental consequences	Social comparison	Behavioural practice/rehearsal	
0	0	Problem solving	Action planning	self monitoring of behaviour	Instruction on how to perform the behaviour	Information about emotinal consequences	Social comparison	Behavioural practice/rehearsal	
0	0	Problem solving	Action planning	self monitoring of behaviour	Information about health consequences	Information abour social & environmental consequences	Information about emotinal consequences	Social comparison	
0	0	Problem solving	Action planning	self monitoring of behaviour	Information about health consequences	Information abour social & environmental consequences	Information about emotinal consequences	Behavioural practice/rehearsal	
0	0	Problem solving	Action planning	self monitoring of behaviour	Information about health consequences	Information abour social & environmental consequences	Social comparison	Behavioural practice/rehearsal	
0	0	Problem solving	Action planning	self monitoring of behaviour	Information about health consequences	Information about emotinal consequences	Social comparison	Behavioural practice/rehearsal	
0	0	Problem solving	Action planning	self monitoring of behaviour	Information abour social & environmental consequences	Information about emotinal consequences	Social comparison	Behavioural practice/rehearsal	
0	0	Problem solving	Action planning	self monitoring of behaviour	social support	Information abour social & environmental consequences	Information about emotinal consequences	Behavioural practice/rehearsal	
0	0	Problem solving	Action planning	self monitoring of behaviour	social support	Information abour social & environmental consequences	Social comparison	Behavioural practice/rehearsal	
0	0	Problem solving	Action planning	self monitoring of behaviour	social support	Information about emotinal consequences	Social comparison	Behavioural practice/rehearsal	
0	0	Problem solving	Action planning	self monitoring of behaviour	Instruction on how to perform the behaviour	Information about health consequences	Information abour social & environmental consequences	Information about emotinal consequences	
0	0	Problem solving	Action planning	self monitoring of behaviour	Instruction on how to perform the behaviour	Information about health consequences	Information abour social & environmental consequences	Social comparison	
0	0	Problem solving	Action planning	self monitoring of behaviour	Instruction on how to perform the behaviour	Information about health consequences	Information about emotinal consequences	Social comparison	
0	0	Problem solving	Action planning	self monitoring of behaviour	Instruction on how to perform the behaviour	Information about health consequences	Information about emotinal consequences	Behavioural practice/rehearsal	
0	0	Problem solving	Action planning	self monitoring of behaviour	Instruction on how to perform the behaviour	Information abour social & environmental consequences	Information about emotinal consequences	Social comparison	
0	0	Problem solving	Feedback on behaviour	social support	Instruction on how to perform the behaviour	Information abour social & environmental consequences	Information about emotinal consequences	Behavioural practice/rehearsal	
0	0	Problem solving	Feedback on behaviour	social support	Instruction on how to perform the behaviour	Information abour social & environmental consequences	Social comparison	Behavioural practice/rehearsal	
0	0	Problem solving	Feedback on behaviour	social support	Instruction on how to perform the behaviour	Information about emotinal consequences	Social comparison	Behavioural practice/rehearsal	
0	0	Problem solving	Feedback on behaviour	social support	Information about health consequences	Information abour social & environmental consequences	Information about emotinal consequences	Social comparison	
0	0	Problem solving	Feedback on behaviour	social support	Information about health consequences	Information abour social & environmental consequences	Information about emotinal consequences	Behavioural practice/rehearsal	
0	0	Problem solving	Feedback on behaviour	social support	Information about health consequences	Information abour social & environmental consequences	Social comparison	Behavioural practice/rehearsal	
0	0	Problem solving	Feedback on behaviour	social support	Information about health consequences	Information about emotinal consequences	Social comparison	Behavioural practice/rehearsal	
0	0	Problem solving	Feedback on behaviour	social support	Information abour social & environmental consequences	Information about emotinal consequences	Social comparison	Behavioural practice/rehearsal	
0	0	Problem solving	Feedback on behaviour	self monitoring of behaviour	Information abour social & environmental consequences	Information about emotinal consequences	Social comparison	Behavioural practice/rehearsal	
0	0	Problem solving	Feedback on behaviour	social support	Instruction on how to perform the behaviour	Information about health consequences	Information abour social & environmental consequences	Information about emotinal consequences	
0	0	Problem solving	Feedback on behaviour	social support	Instruction on how to perform the behaviour	Information about health consequences	Information abour social & environmental consequences	Social comparison	
0	0	Problem solving	Feedback on behaviour	social support	Instruction on how to perform the behaviour	Information about health consequences	Information abour social & environmental consequences	Behavioural practice/rehearsal	
0	0	Problem solving	Feedback on behaviour	social support	Instruction on how to perform the behaviour	Information about health consequences	Information abour social & environmental consequences	Adding object to the environment	
0	0	Problem solving	Feedback on behaviour	social support	Instruction on how to perform the behaviour	Information about health consequences	Information about emotinal consequences	Social comparison	
0	0	Problem solving	Feedback on behaviour	social support	Instruction on how to perform the behaviour	Information about health consequences	Information about emotinal consequences	Behavioural practice/rehearsal	
0	0	Problem solving	Feedback on behaviour	social support	Instruction on how to perform the behaviour	Information abour social & environmental consequences	Information about emotinal consequences	Social comparison	
0	0	Problem solving	Feedback on behaviour	self monitoring of behaviour	Instruction on how to perform the behaviour	Information abour social & environmental consequences	Information about emotinal consequences	Social comparison	
0	0	Problem solving	Feedback on behaviour	self monitoring of behaviour	Instruction on how to perform the behaviour	Information abour social & environmental consequences	Information about emotinal consequences	Behavioural practice/rehearsal	
0	0	Problem solving	Feedback on behaviour	self monitoring of behaviour	Instruction on how to perform the behaviour	Information abour social & environmental consequences	Social comparison	Behavioural practice/rehearsal	
0	0	Problem solving	Feedback on behaviour	self monitoring of behaviour	Instruction on how to perform the behaviour	Information about emotinal consequences	Social comparison	Behavioural practice/rehearsal	
0	0	Problem solving	Feedback on behaviour	self monitoring of behaviour	Information about health consequences	Information abour social & environmental consequences	Information about emotinal consequences	Social comparison	
0	0	Problem solving	Feedback on behaviour	self monitoring of behaviour	Information about health consequences	Information abour social & environmental consequences	Information about emotinal consequences	Behavioural practice/rehearsal	
0	0	Problem solving	Feedback on behaviour	self monitoring of behaviour	Information about health consequences	Information abour social & environmental consequences	Social comparison	Behavioural practice/rehearsal	
0	0	Problem solving	Feedback on behaviour	self monitoring of behaviour	Information about health consequences	Information about emotinal consequences	Social comparison	Behavioural practice/rehearsal	
0	0	Problem solving	Feedback on behaviour	self monitoring of behaviour	social support	Information abour social & environmental consequences	Social comparison	Behavioural practice/rehearsal	
0	0	Problem solving	Feedback on behaviour	self monitoring of behaviour	social support	Information about emotinal consequences	Social comparison	Behavioural practice/rehearsal	
0	0	Problem solving	Feedback on behaviour	self monitoring of behaviour	Instruction on how to perform the behaviour	Information about health consequences	Information abour social & environmental consequences	Information about emotinal consequences	
0	0	Problem solving	Feedback on behaviour	self monitoring of behaviour	Instruction on how to perform the behaviour	Information about health consequences	Information abour social & environmental consequences	Social comparison	
0	0	Problem solving	Feedback on behaviour	self monitoring of behaviour	Instruction on how to perform the behaviour	Information about health consequences	Information abour social & environmental consequences	Behavioural practice/rehearsal	
0	0	Problem solving	Feedback on behaviour	self monitoring of behaviour	Instruction on how to perform the behaviour	Information about health consequences	Information abour social & environmental consequences	Adding object to the environment	
0	0	Problem solving	Feedback on behaviour	self monitoring of behaviour	Instruction on how to perform the behaviour	Information about health consequences	Information about emotinal consequences	Social comparison	
0	0	Problem solving	Feedback on behaviour	self monitoring of behaviour	Instruction on how to perform the behaviour	Information about health consequences	Information about emotinal consequences	Behavioural practice/rehearsal	
0	0	Problem solving	self monitoring of behaviour	Information about health consequences	Information abour social & environmental consequences	Information about emotinal consequences	Social comparison	Behavioural practice/rehearsal	
2	2	Goal setting (outcome)	Action planning	Feedback on behaviour	self monitoring of behaviour	social support	Instruction on how to perform the behaviour	Adding object to the environment	
0	0	Action planning	Feedback on behaviour	self monitoring of behaviour	social support	Instruction on how to perform the behaviour	Information about health consequences	Information abour social & environmental consequences	
0	0	Action planning	Feedback on behaviour	self monitoring of behaviour	social support	Instruction on how to perform the behaviour	Information about health consequences	Information about emotinal consequences	
0	0	Action planning	Feedback on behaviour	self monitoring of behaviour	social support	Instruction on how to perform the behaviour	Information abour social & environmental consequences	Information about emotinal consequences	
0	0	Action planning	Feedback on behaviour	self monitoring of behaviour	social support	Instruction on how to perform the behaviour	Information about emotinal consequences	Social comparison	
0	0	Action planning	Feedback on behaviour	self monitoring of behaviour	social support	Instruction on how to perform the behaviour	Information about emotinal consequences	Behavioural practice/rehearsal	
0	0	Action planning	Feedback on behaviour	self monitoring of behaviour	social support	Instruction on how to perform the behaviour	Behavioural practice/rehearsal	Adding object to the environment	
0	0	Problem solving	self monitoring of behaviour	social support	Information about health consequences	Information abour social & environmental consequences	Social comparison	Behavioural practice/rehearsal	
0	0	Problem solving	self monitoring of behaviour	social support	Information about health consequences	Information about emotinal consequences	Social comparison	Behavioural practice/rehearsal	
0	0	Problem solving	self monitoring of behaviour	social support	Information abour social & environmental consequences	Information about emotinal consequences	Social comparison	Behavioural practice/rehearsal	
0	0	Problem solving	self monitoring of behaviour	Instruction on how to perform the behaviour	Information about health consequences	Information abour social & environmental consequences	Information about emotinal consequences	Social comparison	
0	0	Problem solving	self monitoring of behaviour	Instruction on how to perform the behaviour	Information about health consequences	Information abour social & environmental consequences	Information about emotinal consequences	Behavioural practice/rehearsal	
0	0	Problem solving	self monitoring of behaviour	Instruction on how to perform the behaviour	Information about health consequences	Information abour social & environmental consequences	Social comparison	Behavioural practice/rehearsal	
0	0	Problem solving	self monitoring of behaviour	Instruction on how to perform the behaviour	Information about health consequences	Information about emotinal consequences	Social comparison	Behavioural practice/rehearsal	
0	0	Problem solving	self monitoring of behaviour	Instruction on how to perform the behaviour	Information abour social & environmental consequences	Information about emotinal consequences	Social comparison	Behavioural practice/rehearsal	
0	0	Problem solving	self monitoring of behaviour	social support	Instruction on how to perform the behaviour	Information about health consequences	Information about emotinal consequences	Social comparison	
0	0	Problem solving	self monitoring of behaviour	social support	Instruction on how to perform the behaviour	Information about health consequences	Information about emotinal consequences	Behavioural practice/rehearsal	
0	0	Problem solving	self monitoring of behaviour	social support	Instruction on how to perform the behaviour	Information abour social & environmental consequences	Information about emotinal consequences	Social comparison	
0	0	Problem solving	self monitoring of behaviour	social support	Instruction on how to perform the behaviour	Information abour social & environmental consequences	Information about emotinal consequences	Behavioural practice/rehearsal	
0	0	Problem solving	self monitoring of behaviour	social support	Instruction on how to perform the behaviour	Information abour social & environmental consequences	Social comparison	Behavioural practice/rehearsal	
0	0	Problem solving	self monitoring of behaviour	social support	Instruction on how to perform the behaviour	Information about emotinal consequences	Social comparison	Behavioural practice/rehearsal	
0	0	Problem solving	self monitoring of behaviour	social support	Information about health consequences	Information abour social & environmental consequences	Information about emotinal consequences	Social comparison	
0	0	Problem solving	self monitoring of behaviour	social support	Information about health consequences	Information abour social & environmental consequences	Information about emotinal consequences	Behavioural practice/rehearsal	
0	0	Problem solving	Feedback on behaviour	Instruction on how to perform the behaviour	Information about health consequences	Information abour social & environmental consequences	Information about emotinal consequences	Social comparison	
0	0	Problem solving	Feedback on behaviour	Instruction on how to perform the behaviour	Information about health consequences	Information abour social & environmental consequences	Information about emotinal consequences	Behavioural practice/rehearsal	
0	0	Problem solving	Feedback on behaviour	Instruction on how to perform the behaviour	Information about health consequences	Information abour social & environmental consequences	Social comparison	Behavioural practice/rehearsal	
0	0	Problem solving	Feedback on behaviour	Instruction on how to perform the behaviour	Information about health consequences	Information about emotinal consequences	Social comparison	Behavioural practice/rehearsal	
0	0	Problem solving	Feedback on behaviour	Instruction on how to perform the behaviour	Information abour social & environmental consequences	Information about emotinal consequences	Social comparison	Behavioural practice/rehearsal	
0	0	Problem solving	Feedback on behaviour	Information about health consequences	Information abour social & environmental consequences	Information about emotinal consequences	Social comparison	Behavioural practice/rehearsal	
0	0	Problem solving	self monitoring of behaviour	social support	Instruction on how to perform the behaviour	Information about health consequences	Information abour social & environmental consequences	Information about emotinal consequences	
0	0	Problem solving	self monitoring of behaviour	social support	Instruction on how to perform the behaviour	Information about health consequences	Information abour social & environmental consequences	Social comparison	
0	0	Action planning	Feedback on behaviour	social support	Instruction on how to perform the behaviour	Information about health consequences	Information about emotinal consequences	Social comparison	
0	0	Action planning	Feedback on behaviour	social support	Instruction on how to perform the behaviour	Information about health consequences	Information about emotinal consequences	Behavioural practice/rehearsal	
0	0	Action planning	Feedback on behaviour	social support	Instruction on how to perform the behaviour	Information abour social & environmental consequences	Information about emotinal consequences	Social comparison	
0	0	Action planning	Feedback on behaviour	social support	Instruction on how to perform the behaviour	Information abour social & environmental consequences	Information about emotinal consequences	Behavioural practice/rehearsal	
0	0	Action planning	Feedback on behaviour	social support	Instruction on how to perform the behaviour	Information about emotinal consequences	Social comparison	Behavioural practice/rehearsal	
0	0	Action planning	Feedback on behaviour	social support	Information about health consequences	Information abour social & environmental consequences	Information about emotinal consequences	Social comparison	
0	0	Action planning	Feedback on behaviour	social support	Information about health consequences	Information abour social & environmental consequences	Information about emotinal consequences	Behavioural practice/rehearsal	
0	0	Action planning	Feedback on behaviour	social support	Information about health consequences	Information abour social & environmental consequences	Social comparison	Behavioural practice/rehearsal	
0	0	Action planning	Feedback on behaviour	self monitoring of behaviour	Information about health consequences	Information abour social & environmental consequences	Information about emotinal consequences	Social comparison	
0	0	Action planning	Feedback on behaviour	self monitoring of behaviour	Information about health consequences	Information abour social & environmental consequences	Information about emotinal consequences	Behavioural practice/rehearsal	
0	0	Action planning	Feedback on behaviour	self monitoring of behaviour	Information about health consequences	Information abour social & environmental consequences	Social comparison	Behavioural practice/rehearsal	
0	0	Action planning	Feedback on behaviour	self monitoring of behaviour	Information about health consequences	Information about emotinal consequences	Social comparison	Behavioural practice/rehearsal	
0	0	Action planning	Feedback on behaviour	self monitoring of behaviour	Information abour social & environmental consequences	Information about emotinal consequences	Social comparison	Behavioural practice/rehearsal	
0	0	Action planning	Feedback on behaviour	social support	Instruction on how to perform the behaviour	Information about health consequences	Information abour social & environmental consequences	Information about emotinal consequences	
0	0	Action planning	Feedback on behaviour	social support	Instruction on how to perform the behaviour	Information about health consequences	Information abour social & environmental consequences	Social comparison	
0	0	Action planning	Feedback on behaviour	social support	Instruction on how to perform the behaviour	Information about health consequences	Information abour social & environmental consequences	Behavioural practice/rehearsal	
0	0	Action planning	Feedback on behaviour	self monitoring of behaviour	Instruction on how to perform the behaviour	Information about health consequences	Information abour social & environmental consequences	Information about emotinal consequences	
0	0	Action planning	Feedback on behaviour	self monitoring of behaviour	Instruction on how to perform the behaviour	Information about health consequences	Information abour social & environmental consequences	Social comparison	
0	0	Action planning	Feedback on behaviour	self monitoring of behaviour	Instruction on how to perform the behaviour	Information about health consequences	Information abour social & environmental consequences	Behavioural practice/rehearsal	
0	0	Action planning	Feedback on behaviour	self monitoring of behaviour	Instruction on how to perform the behaviour	Information about health consequences	Information about emotinal consequences	Social comparison	
0	0	Action planning	Feedback on behaviour	self monitoring of behaviour	Instruction on how to perform the behaviour	Information about health consequences	Information about emotinal consequences	Behavioural practice/rehearsal	
0	0	Action planning	Feedback on behaviour	self monitoring of behaviour	Instruction on how to perform the behaviour	Information abour social & environmental consequences	Information about emotinal consequences	Social comparison	
0	0	Action planning	Feedback on behaviour	self monitoring of behaviour	Instruction on how to perform the behaviour	Information abour social & environmental consequences	Information about emotinal consequences	Behavioural practice/rehearsal	
0	0	Action planning	Feedback on behaviour	self monitoring of behaviour	Instruction on how to perform the behaviour	Information about emotinal consequences	Social comparison	Behavioural practice/rehearsal	
0	0	Action planning	Feedback on behaviour	self monitoring of behaviour	social support	Information about health consequences	Information abour social & environmental consequences	Information about emotinal consequences	
0	0	Action planning	Feedback on behaviour	self monitoring of behaviour	social support	Information about health consequences	Information abour social & environmental consequences	Social comparison	
0	0	Action planning	Feedback on behaviour	self monitoring of behaviour	social support	Information about health consequences	Information abour social & environmental consequences	Behavioural practice/rehearsal	
0	0	Action planning	Feedback on behaviour	self monitoring of behaviour	social support	Information about health consequences	Information about emotinal consequences	Social comparison	
0	0	Action planning	Feedback on behaviour	self monitoring of behaviour	social support	Information about health consequences	Information about emotinal consequences	Behavioural practice/rehearsal	
0	0	Action planning	Feedback on behaviour	self monitoring of behaviour	social support	Information abour social & environmental consequences	Information about emotinal consequences	Social comparison	
0	0	Action planning	Feedback on behaviour	self monitoring of behaviour	social support	Information abour social & environmental consequences	Information about emotinal consequences	Behavioural practice/rehearsal	
0	0	Action planning	Feedback on behaviour	self monitoring of behaviour	social support	Information about emotinal consequences	Social comparison	Behavioural practice/rehearsal	
0	0	Action planning	self monitoring of behaviour	Information about health consequences	Information abour social & environmental consequences	Information about emotinal consequences	Social comparison	Behavioural practice/rehearsal	
0	0	Feedback on behaviour	self monitoring of behaviour	social support	Instruction on how to perform the behaviour	Information about health consequences	Information abour social & environmental consequences	Information about emotinal consequences	
0	0	Feedback on behaviour	self monitoring of behaviour	social support	Instruction on how to perform the behaviour	Information about health consequences	Information abour social & environmental consequences	Social comparison	
0	0	Feedback on behaviour	self monitoring of behaviour	social support	Instruction on how to perform the behaviour	Information about health consequences	Information abour social & environmental consequences	Behavioural practice/rehearsal	
0	0	Feedback on behaviour	self monitoring of behaviour	social support	Instruction on how to perform the behaviour	Information about health consequences	Information abour social & environmental consequences	Adding object to the environment	
0	0	Feedback on behaviour	self monitoring of behaviour	social support	Instruction on how to perform the behaviour	Information about health consequences	Information about emotinal consequences	Social comparison	
0	0	Feedback on behaviour	self monitoring of behaviour	social support	Instruction on how to perform the behaviour	Information about health consequences	Information about emotinal consequences	Behavioural practice/rehearsal	
0	0	Feedback on behaviour	self monitoring of behaviour	social support	Instruction on how to perform the behaviour	Information abour social & environmental consequences	Information about emotinal consequences	Social comparison	
0	0	Action planning	self monitoring of behaviour	social support	Information about health consequences	Information abour social & environmental consequences	Social comparison	Behavioural practice/rehearsal	
0	0	Action planning	self monitoring of behaviour	social support	Information about health consequences	Information about emotinal consequences	Social comparison	Behavioural practice/rehearsal	
0	0	Action planning	self monitoring of behaviour	social support	Information abour social & environmental consequences	Information about emotinal consequences	Social comparison	Behavioural practice/rehearsal	
0	0	Action planning	self monitoring of behaviour	Instruction on how to perform the behaviour	Information about health consequences	Information abour social & environmental consequences	Information about emotinal consequences	Social comparison	
0	0	Action planning	self monitoring of behaviour	Instruction on how to perform the behaviour	Information about health consequences	Information abour social & environmental consequences	Information about emotinal consequences	Behavioural practice/rehearsal	
0	0	Action planning	self monitoring of behaviour	Instruction on how to perform the behaviour	Information about health consequences	Information abour social & environmental consequences	Social comparison	Behavioural practice/rehearsal	
0	0	Action planning	self monitoring of behaviour	Instruction on how to perform the behaviour	Information about health consequences	Information about emotinal consequences	Social comparison	Behavioural practice/rehearsal	
0	0	Action planning	self monitoring of behaviour	Instruction on how to perform the behaviour	Information abour social & environmental consequences	Information about emotinal consequences	Social comparison	Behavioural practice/rehearsal	
0	0	Action planning	self monitoring of behaviour	social support	Instruction on how to perform the behaviour	Information about health consequences	Information abour social & environmental consequences	Information about emotinal consequences	
0	0	Action planning	self monitoring of behaviour	social support	Instruction on how to perform the behaviour	Information about health consequences	Information abour social & environmental consequences	Social comparison	
0	0	Action planning	self monitoring of behaviour	social support	Instruction on how to perform the behaviour	Information about health consequences	Information about emotinal consequences	Behavioural practice/rehearsal	
0	0	Action planning	self monitoring of behaviour	social support	Instruction on how to perform the behaviour	Information abour social & environmental consequences	Information about emotinal consequences	Social comparison	
0	0	Action planning	self monitoring of behaviour	social support	Instruction on how to perform the behaviour	Information abour social & environmental consequences	Information about emotinal consequences	Behavioural practice/rehearsal	
0	0	Action planning	self monitoring of behaviour	social support	Instruction on how to perform the behaviour	Information about emotinal consequences	Social comparison	Behavioural practice/rehearsal	
0	0	Action planning	self monitoring of behaviour	social support	Information about health consequences	Information abour social & environmental consequences	Information about emotinal consequences	Social comparison	
0	0	Action planning	self monitoring of behaviour	social support	Information about health consequences	Information abour social & environmental consequences	Information about emotinal consequences	Behavioural practice/rehearsal	
0	0	Action planning	Feedback on behaviour	social support	Information about health consequences	Information about emotinal consequences	Social comparison	Behavioural practice/rehearsal	
0	0	Action planning	Feedback on behaviour	social support	Information abour social & environmental consequences	Information about emotinal consequences	Social comparison	Behavioural practice/rehearsal	
0	0	Action planning	Feedback on behaviour	Instruction on how to perform the behaviour	Information about health consequences	Information abour social & environmental consequences	Information about emotinal consequences	Social comparison	
0	0	Action planning	Feedback on behaviour	Instruction on how to perform the behaviour	Information about health consequences	Information abour social & environmental consequences	Information about emotinal consequences	Behavioural practice/rehearsal	
0	0	Action planning	Feedback on behaviour	Instruction on how to perform the behaviour	Information about health consequences	Information abour social & environmental consequences	Social comparison	Behavioural practice/rehearsal	
0	0	Action planning	Feedback on behaviour	Instruction on how to perform the behaviour	Information about health consequences	Information about emotinal consequences	Social comparison	Behavioural practice/rehearsal	
0	0	Action planning	Feedback on behaviour	Instruction on how to perform the behaviour	Information abour social & environmental consequences	Information about emotinal consequences	Social comparison	Behavioural practice/rehearsal	
0	0	Action planning	Feedback on behaviour	Information about health consequences	Information abour social & environmental consequences	Information about emotinal consequences	Social comparison	Behavioural practice/rehearsal	
0	0	self monitoring of behaviour	social support	Instruction on how to perform the behaviour	Information abour social & environmental consequences	Information about emotinal consequences	Social comparison	Behavioural practice/rehearsal	
0	0	self monitoring of behaviour	social support	Information about health consequences	Information abour social & environmental consequences	Information about emotinal consequences	Social comparison	Behavioural practice/rehearsal	
0	0	self monitoring of behaviour	Instruction on how to perform the behaviour	Information about health consequences	Information abour social & environmental consequences	Information about emotinal consequences	Social comparison	Behavioural practice/rehearsal	
2	2	Goal setting (behaviour)	Problem solving	Goal setting (outcome)	Action planning	Feedback on behaviour	self monitoring of behaviour	social support	Instruction on how to perform the behaviour
2	2	Goal setting (behaviour)	Problem solving	Goal setting (outcome)	Action planning	Feedback on behaviour	self monitoring of behaviour	social support	Adding object to the environment
2	2	Goal setting (behaviour)	Problem solving	Goal setting (outcome)	Action planning	Feedback on behaviour	self monitoring of behaviour	Instruction on how to perform the behaviour	Adding object to the environment
2	2	Goal setting (behaviour)	Problem solving	Goal setting (outcome)	Action planning	Feedback on behaviour	social support	Instruction on how to perform the behaviour	Adding object to the environment
2	2	Goal setting (behaviour)	Problem solving	Goal setting (outcome)	Feedback on behaviour	self monitoring of behaviour	social support	Instruction on how to perform the behaviour	Adding object to the environment
0	0	Feedback on behaviour	social support	Instruction on how to perform the behaviour	Information about health consequences	Information about emotinal consequences	Social comparison	Behavioural practice/rehearsal	
0	0	Feedback on behaviour	social support	Instruction on how to perform the behaviour	Information abour social & environmental consequences	Information about emotinal consequences	Social comparison	Behavioural practice/rehearsal	
0	0	Feedback on behaviour	social support	Information about health consequences	Information abour social & environmental consequences	Information about emotinal consequences	Social comparison	Behavioural practice/rehearsal	
0	0	Feedback on behaviour	Instruction on how to perform the behaviour	Information about health consequences	Information abour social & environmental consequences	Information about emotinal consequences	Social comparison	Behavioural practice/rehearsal	
0	0	self monitoring of behaviour	social support	Instruction on how to perform the behaviour	Information about health consequences	Information abour social & environmental consequences	Information about emotinal consequences	Social comparison	
0	0	self monitoring of behaviour	social support	Instruction on how to perform the behaviour	Information about health consequences	Information abour social & environmental consequences	Information about emotinal consequences	Behavioural practice/rehearsal	
0	0	self monitoring of behaviour	social support	Instruction on how to perform the behaviour	Information about health consequences	Information abour social & environmental consequences	Social comparison	Behavioural practice/rehearsal	
0	0	self monitoring of behaviour	social support	Instruction on how to perform the behaviour	Information about health consequences	Information about emotinal consequences	Social comparison	Behavioural practice/rehearsal	
0	0	Feedback on behaviour	self monitoring of behaviour	Instruction on how to perform the behaviour	Information about health consequences	Information abour social & environmental consequences	Information about emotinal consequences	Behavioural practice/rehearsal	
0	0	Feedback on behaviour	self monitoring of behaviour	Instruction on how to perform the behaviour	Information about health consequences	Information abour social & environmental consequences	Social comparison	Behavioural practice/rehearsal	
0	0	Feedback on behaviour	self monitoring of behaviour	Instruction on how to perform the behaviour	Information about health consequences	Information about emotinal consequences	Social comparison	Behavioural practice/rehearsal	
0	0	Feedback on behaviour	self monitoring of behaviour	Instruction on how to perform the behaviour	Information abour social & environmental consequences	Information about emotinal consequences	Social comparison	Behavioural practice/rehearsal	
0	0	Feedback on behaviour	self monitoring of behaviour	Information about health consequences	Information abour social & environmental consequences	Information about emotinal consequences	Social comparison	Behavioural practice/rehearsal	
0	0	Feedback on behaviour	social support	Instruction on how to perform the behaviour	Information about health consequences	Information abour social & environmental consequences	Information about emotinal consequences	Social comparison	
0	0	Feedback on behaviour	social support	Instruction on how to perform the behaviour	Information about health consequences	Information abour social & environmental consequences	Information about emotinal consequences	Behavioural practice/rehearsal	
0	0	Feedback on behaviour	social support	Instruction on how to perform the behaviour	Information about health consequences	Information abour social & environmental consequences	Social comparison	Behavioural practice/rehearsal	
0	0	Feedback on behaviour	self monitoring of behaviour	social support	Instruction on how to perform the behaviour	Information abour social & environmental consequences	Information about emotinal consequences	Behavioural practice/rehearsal	
0	0	Feedback on behaviour	self monitoring of behaviour	social support	Instruction on how to perform the behaviour	Information about emotinal consequences	Social comparison	Behavioural practice/rehearsal	
0	0	Feedback on behaviour	self monitoring of behaviour	social support	Information about health consequences	Information abour social & environmental consequences	Information about emotinal consequences	Social comparison	
0	0	Feedback on behaviour	self monitoring of behaviour	social support	Information about health consequences	Information abour social & environmental consequences	Information about emotinal consequences	Behavioural practice/rehearsal	
0	0	Feedback on behaviour	self monitoring of behaviour	social support	Information about health consequences	Information abour social & environmental consequences	Social comparison	Behavioural practice/rehearsal	
0	0	Feedback on behaviour	self monitoring of behaviour	social support	Information about health consequences	Information about emotinal consequences	Social comparison	Behavioural practice/rehearsal	
0	0	Feedback on behaviour	self monitoring of behaviour	social support	Information abour social & environmental consequences	Information about emotinal consequences	Social comparison	Behavioural practice/rehearsal	
0	0	Feedback on behaviour	self monitoring of behaviour	Instruction on how to perform the behaviour	Information about health consequences	Information abour social & environmental consequences	Information about emotinal consequences	Social comparison	
0	0	Goal setting (behaviour)	Problem solving	Action planning	Feedback on behaviour	self monitoring of behaviour	Information abour social & environmental consequences	Information about emotinal consequences	Behavioural practice/rehearsal
0	0	Goal setting (behaviour)	Problem solving	Action planning	Feedback on behaviour	self monitoring of behaviour	Information abour social & environmental consequences	Social comparison	Behavioural practice/rehearsal
0	0	Goal setting (behaviour)	Problem solving	Action planning	Feedback on behaviour	self monitoring of behaviour	Information about emotinal consequences	Social comparison	Behavioural practice/rehearsal
0	0	Goal setting (behaviour)	Problem solving	Action planning	Feedback on behaviour	social support	Instruction on how to perform the behaviour	Information about health consequences	Information abour social & environmental consequences
0	0	Goal setting (behaviour)	Problem solving	Action planning	Feedback on behaviour	social support	Instruction on how to perform the behaviour	Information about health consequences	Information about emotinal consequences
0	0	Goal setting (behaviour)	Problem solving	Action planning	Feedback on behaviour	social support	Instruction on how to perform the behaviour	Information abour social & environmental consequences	Information about emotinal consequences
0	0	Goal setting (behaviour)	Problem solving	Action planning	Feedback on behaviour	social support	Instruction on how to perform the behaviour	Information abour social & environmental consequences	Social comparison
0	0	Goal setting (behaviour)	Problem solving	Action planning	Feedback on behaviour	social support	Instruction on how to perform the behaviour	Information abour social & environmental consequences	Behavioural practice/rehearsal
0	0	Goal setting (behaviour)	Problem solving	Action planning	Feedback on behaviour	self monitoring of behaviour	Instruction on how to perform the behaviour	Information about emotinal consequences	Behavioural practice/rehearsal
0	0	Goal setting (behaviour)	Problem solving	Action planning	Feedback on behaviour	self monitoring of behaviour	Instruction on how to perform the behaviour	Behavioural practice/rehearsal	Adding object to the environment
0	0	Goal setting (behaviour)	Problem solving	Action planning	Feedback on behaviour	self monitoring of behaviour	Information about health consequences	Information abour social & environmental consequences	Information about emotinal consequences
0	0	Goal setting (behaviour)	Problem solving	Action planning	Feedback on behaviour	self monitoring of behaviour	Information about health consequences	Information abour social & environmental consequences	Social comparison
0	0	Goal setting (behaviour)	Problem solving	Action planning	Feedback on behaviour	self monitoring of behaviour	Information about health consequences	Information abour social & environmental consequences	Behavioural practice/rehearsal
0	0	Goal setting (behaviour)	Problem solving	Action planning	Feedback on behaviour	self monitoring of behaviour	Information about health consequences	Information about emotinal consequences	Social comparison
0	0	Goal setting (behaviour)	Problem solving	Action planning	Feedback on behaviour	self monitoring of behaviour	Information about health consequences	Information about emotinal consequences	Behavioural practice/rehearsal
0	0	Goal setting (behaviour)	Problem solving	Action planning	Feedback on behaviour	self monitoring of behaviour	Information abour social & environmental consequences	Information about emotinal consequences	Social comparison
0	0	Goal setting (behaviour)	Problem solving	Action planning	Feedback on behaviour	self monitoring of behaviour	social support	Information about emotinal consequences	Behavioural practice/rehearsal
0	0	Goal setting (behaviour)	Problem solving	Action planning	Feedback on behaviour	self monitoring of behaviour	social support	Behavioural practice/rehearsal	Adding object to the environment
0	0	Goal setting (behaviour)	Problem solving	Action planning	Feedback on behaviour	self monitoring of behaviour	Instruction on how to perform the behaviour	Information about health consequences	Information abour social & environmental consequences
0	0	Goal setting (behaviour)	Problem solving	Action planning	Feedback on behaviour	self monitoring of behaviour	Instruction on how to perform the behaviour	Information about health consequences	Information about emotinal consequences
0	0	Goal setting (behaviour)	Problem solving	Action planning	Feedback on behaviour	self monitoring of behaviour	Instruction on how to perform the behaviour	Information abour social & environmental consequences	Information about emotinal consequences
0	0	Goal setting (behaviour)	Problem solving	Action planning	Feedback on behaviour	self monitoring of behaviour	Instruction on how to perform the behaviour	Information abour social & environmental consequences	Social comparison
0	0	Goal setting (behaviour)	Problem solving	Action planning	Feedback on behaviour	self monitoring of behaviour	Instruction on how to perform the behaviour	Information abour social & environmental consequences	Behavioural practice/rehearsal
0	0	Goal setting (behaviour)	Problem solving	Action planning	Feedback on behaviour	self monitoring of behaviour	Instruction on how to perform the behaviour	Information about emotinal consequences	Social comparison
0	0	Goal setting (behaviour)	Problem solving	Action planning	Feedback on behaviour	self monitoring of behaviour	social support	Instruction on how to perform the behaviour	Information abour social & environmental consequences
0	0	Goal setting (behaviour)	Problem solving	Action planning	Feedback on behaviour	self monitoring of behaviour	social support	Instruction on how to perform the behaviour	Information about emotinal consequences
0	0	Goal setting (behaviour)	Problem solving	Action planning	Feedback on behaviour	self monitoring of behaviour	social support	Information about health consequences	Information abour social & environmental consequences
0	0	Goal setting (behaviour)	Problem solving	Action planning	Feedback on behaviour	self monitoring of behaviour	social support	Information about health consequences	Information about emotinal consequences

3	2	0	0	0	0	0	0	1	0	0	0	0	0	0	0	0	0	0	0	0	0	3	0	0	0	0	0	0	Goal setting (behaviour)	Problem solving	Action planning	Feedback on behaviour	self monitoring of behaviour	social support	Information about social & environmental consequences	Information about emotional consequences	67	0	0	0	0	0	0	8
3	2	0	0	0	0	0	0	1	0	0	0	0	0	0	0	0	0	0	0	0	0	3	0	0	0	0	0	0	Goal setting (behaviour)	Problem solving	Action planning	Feedback on behaviour	self monitoring of behaviour	social support	Information about social & environmental consequences	Social comparison	67	0	0	0	0	0	0	8
3	2	0	0	0	0	0	0	1	0	0	0	0	0	0	0	0	0	0	0	0	0	3	0	0	0	0	0	0	Goal setting (behaviour)	Problem solving	Action planning	Feedback on behaviour	self monitoring of behaviour	social support	Information about social & environmental consequences	Behavioural practice/rehearsal	67	0	0	0	0	0	0	8
3	2	0	0	0	0	0	0	1	0	0	0	0	0	0	0	0	0	0	0	0	0	3	0	0	0	0	0	0	Goal setting (behaviour)	Problem solving	Action planning	Feedback on behaviour	self monitoring of behaviour	social support	Information about emotional consequences	Social comparison	67	0	0	0	0	0	0	8
3	2	0	0	0	0	0	0	1	0	0	0	0	0	0	0	0	0	0	0	0	0	3	0	0	0	0	0	0	Goal setting (behaviour)	Problem solving	Action planning	Feedback on behaviour	Information about health consequences	Information about emotional consequences	Social comparison	Behavioural practice/rehearsal	67	0	0	0	0	0	0	8
3	2	0	0	0	0	0	0	1	0	0	0	0	0	0	0	0	0	0	0	0	0	3	0	0	0	0	0	0	Goal setting (behaviour)	Problem solving	Action planning	Feedback on behaviour	Information about social & environmental consequences	Information about emotional consequences	Social comparison	Behavioural practice/rehearsal	67	0	0	0	0	0	0	8
3	2	0	0	0	0	0	0	1	0	0	0	0	0	0	0	0	0	0	0	0	0	3	0	0	0	0	0	0	Goal setting (behaviour)	Problem solving	Action planning	self monitoring of behaviour	social support	Instruction on how to perform the behaviour	Information about health consequences	Information about emotional consequences	67	0	0	0	0	0	0	8
3	2	0	0	0	0	0	0	1	0	0	0	0	0	0	0	0	0	0	0	0	0	3	0	0	0	0	0	0	Goal setting (behaviour)	Problem solving	Action planning	self monitoring of behaviour	social support	Instruction on how to perform the behaviour	Information about social & environmental consequences	Information about emotional consequences	67	0	0	0	0	0	0	8
3	2	0	0	0	0	0	0	1	0	0	0	0	0	0	0	0	0	0	0	0	0	3	0	0	0	0	0	0	Goal setting (behaviour)	Problem solving	Action planning	self monitoring of behaviour	social support	Instruction on how to perform the behaviour	Information about social & environmental consequences	Social comparison	67	0	0	0	0	0	0	8
3	2	0	0	0	0	0	0	1	0	0	0	0	0	0	0	0	0	0	0	0	0	3	0	0	0	0	0	0	Goal setting (behaviour)	Problem solving	Action planning	self monitoring of behaviour	social support	Instruction on how to perform the behaviour	Information about emotional consequences	Social comparison	67	0	0	0	0	0	0	8
3	2	0	0	0	0	0	0	1	0	0	0	0	0	0	0	0	0	0	0	0	0	3	0	0	0	0	0	0	Goal setting (behaviour)	Problem solving	Action planning	self monitoring of behaviour	social support	Instruction on how to perform the behaviour	Information about emotional consequences	Behavioural practice/rehearsal	67	0	0	0	0	0	0	8
3	2	0	0	0	0	0	0	1	0	0	0	0	0	0	0	0	0	0	0	0	0	3	0	0	0	0	0	0	Goal setting (behaviour)	Problem solving	Action planning	self monitoring of behaviour	social support	Information about health consequences	Information about social & environmental consequences	Information about emotional consequences	67	0	0	0	0	0	0	8
3	2	0	0	0	0	0	0	1	0	0	0	0	0	0	0	0	0	0	0	0	0	3	0	0	0	0	0	0	Goal setting (behaviour)	Problem solving	Action planning	Feedback on behaviour	Instruction on how to perform the behaviour	Information about health consequences	Information about emotional consequences	Behavioural practice/rehearsal	67	0	0	0	0	0	0	8
3	2	0	0	0	0	0	0	1	0	0	0	0	0	0	0	0	0	0	0	0	0	3	0	0	0	0	0	0	Goal setting (behaviour)	Problem solving	Action planning	Feedback on behaviour	Instruction on how to perform the behaviour	Information about social & environmental consequences	Information about emotional consequences	Social comparison	67	0	0	0	0	0	0	8
3	2	0	0	0	0	0	0	1	0	0	0	0	0	0	0	0	0	0	0	0	0	3	0	0	0	0	0	0	Goal setting (behaviour)	Problem solving	Action planning	Feedback on behaviour	Instruction on how to perform the behaviour	Information about social & environmental consequences	Information about emotional consequences	Behavioural practice/rehearsal	67	0	0	0	0	0	0	8
3	2	0	0	0	0	0	0	1	0	0	0	0	0	0	0	0	0	0	0	0	0	3	0	0	0	0	0	0	Goal setting (behaviour)	Problem solving	Action planning	Feedback on behaviour	Instruction on how to perform the behaviour	Information about social & environmental consequences	Social comparison	Behavioural practice/rehearsal	67	0	0	0	0	0	0	8
3	2	0	0	0	0	0	0	1	0	0	0	0	0	0	0	0	0	0	0	0	0	3	0	0	0	0	0	0	Goal setting (behaviour)	Problem solving	Action planning	Feedback on behaviour	Instruction on how to perform the behaviour	Information about emotional consequences	Social comparison	Behavioural practice/rehearsal	67	0	0	0	0	0	0	8
3	2	0	0	0	0	0	0	1	0	0	0	0	0	0	0	0	0	0	0	0	0	3	0	0	0	0	0	0	Goal setting (behaviour)	Problem solving	Action planning	Feedback on behaviour	Information about health consequences	Information about social & environmental consequences	Information about emotional consequences	Social comparison	67	0	0	0	0	0	0	8
3	2	0	0	0	0	0	0	1	0	0	0	0	0	0	0	0	0	0	0	0	0	3	0	0	0	0	0	0	Goal setting (behaviour)	Problem solving	Action planning	Feedback on behaviour	Information about health consequences	Information about social & environmental consequences	Information about emotional consequences	Behavioural practice/rehearsal	67	0	0	0	0	0	0	8
3	2	0	0	0	0	0	0	1	0	0	0	0	0	0	0	0	0	0	0	0	0	3	0	0	0	0	0	0	Goal setting (behaviour)	Problem solving	Action planning	Feedback on behaviour	Information about health consequences	Information about social & environmental consequences	Social comparison	Behavioural practice/rehearsal	67	0	0	0	0	0	0	8
3	2	0	0	0	0	0	0	1	0	0	0	0	0	0	0	0	0	0	0	0	0	3	0	0	0	0	0	0	Goal setting (behaviour)	Problem solving	Action planning	Feedback on behaviour	social support	Information about social & environmental consequences	Information about emotional consequences	Social comparison	67	0	0	0	0	0	0	8
3	2	0	0	0	0	0	0	1	0	0	0	0	0	0	0	0	0	0	0	0	0	3	0	0	0	0	0	0	Goal setting (behaviour)	Problem solving	Action planning	Feedback on behaviour	social support	Information about social & environmental consequences	Information about emotional consequences	Behavioural practice/rehearsal	67	0	0	0	0	0	0	8
3	2	0	0	0	0	0	0	1	0	0	0	0	0	0	0	0	0	0	0	0	0	3	0	0	0	0	0	0	Goal setting (behaviour)	Problem solving	Action planning	Feedback on behaviour	social support	Information about social & environmental consequences	Social comparison	Behavioural practice/rehearsal	67	0	0	0	0	0	0	8
3	2	0	0	0	0	0	0	1	0	0	0	0	0	0	0	0	0	0	0	0	0	3	0	0	0	0	0	0	Goal setting (behaviour)	Problem solving	Action planning	Feedback on behaviour	social support	Information about emotional consequences	Social comparison	Behavioural practice/rehearsal	67	0	0	0	0	0	0	8
3	2	0	0	0	0	0	0	1	0	0	0	0	0	0	0	0	0	0	0	0	0	3	0	0	0	0	0	0	Goal setting (behaviour)	Problem solving	Action planning	Feedback on behaviour	Instruction on how to perform the behaviour	Information about health consequences	Information about social & environmental consequences	Information about emotional consequences	67	0	0	0	0	0	0	8
3	2	0	0	0	0	0	0	1	0	0	0	0	0	0	0	0	0	0	0	0	0	3	0	0	0	0	0	0	Goal setting (behaviour)	Problem solving	Action planning	Feedback on behaviour	Instruction on how to perform the behaviour	Information about health consequences	Information about social & environmental consequences	Social comparison	67	0	0	0	0	0	0	8
3	2	0	0	0	0	0	0	1	0	0	0	0	0	0	0	0	0	0	0	0	0	3	0	0	0	0	0	0	Goal setting (behaviour)	Problem solving	Action planning	Feedback on behaviour	Instruction on how to perform the behaviour	Information about health consequences	Information about social & environmental consequences	Behavioural practice/rehearsal	67	0	0	0	0	0	0	8
3	2	0	0	0	0	0	0	1	0	0	0	0	0	0	0	0	0	0	0	0	0	3	0	0	0	0	0	0	Goal setting (behaviour)	Problem solving	Action planning	Feedback on behaviour	Instruction on how to perform the behaviour	Information about health consequences	Information about emotional consequences	Social comparison	67	0	0	0	0	0	0	8
3	2	0	0	0	0	0	0	1	0	0	0	0	0	0	0	0	0	0	0	0	0	3	0	0	0	0	0	0	Goal setting (behaviour)	Problem solving	Action planning	Feedback on behaviour	social support	Instruction on how to perform the behaviour	Information about emotional consequences	Social comparison	67	0	0	0	0	0	0	8
3	2	0	0	0	0	0	0	1	0	0	0	0	0	0	0	0	0	0	0	0	0	3	0	0	0	0	0	0	Goal setting (behaviour)	Problem solving	Action planning	Feedback on behaviour	social support	Instruction on how to perform the behaviour	Information about emotional consequences	Behavioural practice/rehearsal	67	0	0	0	0	0	0	8
3	2	0	0	0	0	0	0	1	0	0	0	0	0	0	0	0	0	0	0	0	0	3	0	0	0	0	0	0	Goal setting (behaviour)	Problem solving	Action planning	Feedback on behaviour	social support	Instruction on how to perform the behaviour	Behavioural practice/rehearsal	Adding object to the environment	67	0	0	0	0	0	0	8
3	2	0	0	0	0	0	0	1	0	0	0	0	0	0	0	0	0	0	0	0	0	3	0	0	0	0	0	0	Goal setting (behaviour)	Problem solving	Action planning	Feedback on behaviour	social support	Information about health consequences	Information about social & environmental consequences	Information about emotional consequences	67	0	0	0	0	0	0	8
3	2	0	0	0	0	0	0	1	0	0	0	0	0	0	0	0	0	0	0	0	0	3	0	0	0	0	0	0	Goal setting (behaviour)	Problem solving	Action planning	Feedback on behaviour	social support	Information about health consequences	Information about social & environmental consequences	Social comparison	67	0	0	0	0	0	0	8
3	2	0	0	0	0	0	0	1	0	0	0	0	0	0	0	0	0	0	0	0	0	3	0	0	0	0	0	0	Goal setting (behaviour)	Problem solving	Action planning	Feedback on behaviour	social support	Information about health consequences	Information about social & environmental consequences	Behavioural practice/rehearsal	67	0	0	0	0	0	0	8
3	2	0	0	0	0	0	0	1	0	0	0	0	0	0	0	0	0	0	0	0	0	3	0	0	0	0	0	0	Goal setting (behaviour)	Problem solving	Action planning	Feedback on behaviour	social support	Information about health consequences	Information about emotional consequences	Social comparison	67	0	0	0	0	0	0	8
3	2	0	0	0	0	0	0	1	0	0	0	0	0	0	0	0	0	0	0	0	0	3	0	0	0	0	0	0	Goal setting (behaviour)	Problem solving	Action planning	Feedback on behaviour	social support	Information about health consequences	Information about emotional consequences	Behavioural practice/rehearsal	67	0	0	0	0	0	0	8
3	2	0	0	0	0	0	0	1	0	0	0	0	0	0	0	0	0	0	0	0	0	3	0	0	0	0	0	0	Goal setting (behaviour)	Problem solving	Feedback on behaviour	self monitoring of behaviour	social support	Instruction on how to perform the behaviour	Information about social & environmental consequences	Adding object to the environment	67	0	0	0	0	0	0	8
3	2	0	0	0	0	0	0	1	0	0	0	0	0	0	0	0	0	0	0	0	0	3	0	0	0	0	0	0	Goal setting (behaviour)	Problem solving	Feedback on behaviour	self monitoring of behaviour	social support	Instruction on how to perform the behaviour	Information about emotional consequences	Social comparison	67	100	0	0	0	0	0	8
3	2	0	0	0	0	0	0	1	0	0	0	0	0	0	0	0	0	0	0	0	0	3	0	0	0	0	0	0	Goal setting (behaviour)	Problem solving	Feedback on behaviour	self monitoring of behaviour	social support	Instruction on how to perform the behaviour	Information about emotional consequences	Behavioural practice/rehearsal	67	0	0	0	0	0	0	8
3	2	0	0	0	0	0	0	1	0	0	0	0	0	0	0	0	0	0	0	0	0	3	0	0	0	0	0	0	Goal setting (behaviour)	Problem solving	Feedback on behaviour	self monitoring of behaviour	social support	Instruction on how to perform the behaviour	Behavioural practice/rehearsal	Adding object to the environment	67	0	0	0	0	0	0	8
3	2	0	0	0	0	0	0	1	0	0	0	0	0	0	0	0	0	0	0	0	0	3	0	0	0	0	0	0	Goal setting (behaviour)	Problem solving	Feedback on behaviour	self monitoring of behaviour	social support	Information about health consequences	Information about social & environmental consequences	Information about emotional consequences	67	0	0	0	0	0	0	8
3	2	0	0	0	0	0	0	1	0	0	0	0	0	0	0	0	0	0	0	0	0	3	0	0	0	0	0	0	Goal setting (behaviour)	Problem solving	Feedback on behaviour	self monitoring of behaviour	social support	Information about health consequences	Information about social & environmental consequences	Social comparison	67	0	0	0	0	0	0	8
3	2	0	0	0	0	0	0	1	0	0	0	0	0	0	0	0	0	0	0	0	0	3	0	0	0	0	0	0	Goal setting (behaviour)	Problem solving	Feedback on behaviour	self monitoring of behaviour	social support	Information about health consequences	Information about social & environmental consequences	Behavioural practice/rehearsal	67	0	0	0	0	0	0	8
3	2	0	0	0	0	0	0	1	0	0	0	0	0	0	0	0	0	0	0	0	0	3	0	0	0	0	0	0	Goal setting (behaviour)	Problem solving	Feedback on behaviour	self monitoring of behaviour	social support	Information about health consequences	Information about social & environmental consequences	Adding object to the environment	67	0	0	0	0	0	0	8

0	0	Action planning	Feedback on behaviour	social support	Instruction on how to perform the behaviour	Information abour social & environmental consequences	Information about emotinal consequences	Social comparison	Behavioural practice/rehearsal
0	0	Action planning	Feedback on behaviour	social support	Information about health consequences	Information abour social & environmental consequences	Information about emotinal consequences	Social comparison	Behavioural practice/rehearsal
0	0	Action planning	Feedback on behaviour	Instruction on how to perform the behaviour	Information about health consequences	Information abour social & environmental consequences	Information about emotinal consequences	Social comparison	Behavioural practice/rehearsal
0	0	Action planning	self monitoring of behaviour	social support	Instruction on how to perform the behaviour	Information about health consequences	Information abour social & environmental consequences	Information about emotinal consequences	Social comparison
0	0	Action planning	self monitoring of behaviour	social support	Instruction on how to perform the behaviour	Information about health consequences	Information abour social & environmental consequences	Information about emotinal consequences	Behavioural practice/rehearsal
0	0	Action planning	self monitoring of behaviour	social support	Instruction on how to perform the behaviour	Information about health consequences	Information abour social & environmental consequences	Social comparison	Behavioural practice/rehearsal
0	0	Action planning	self monitoring of behaviour	social support	Instruction on how to perform the behaviour	Information about health consequences	Information about emotinal consequences	Social comparison	Behavioural practice/rehearsal
0	0	Goal setting (behaviour)	Problem solving	Action planning	Feedback on behaviour	social support	Instruction on how to perform the behaviour	Information about health consequences	Information about emotinal consequences
0	0	Goal setting (behaviour)	Problem solving	Action planning	Feedback on behaviour	social support	Instruction on how to perform the behaviour	Information abour social & environmental consequences	Information about emotinal consequences
0	0	Goal setting (behaviour)	Problem solving	Action planning	Feedback on behaviour	social support	Instruction on how to perform the behaviour	Information abour social & environmental consequences	Information about emotinal consequences
0	0	Goal setting (behaviour)	Problem solving	Action planning	Feedback on behaviour	social support	Instruction on how to perform the behaviour	Information abour social & environmental consequences	Social comparison
0	0	Goal setting (behaviour)	Problem solving	Action planning	Feedback on behaviour	social support	Instruction on how to perform the behaviour	Information about emotinal consequences	Social comparison
0	0	Goal setting (behaviour)	Problem solving	Action planning	Feedback on behaviour	social support	Information about health consequences	Information abour social & environmental consequences	Information about emotinal consequences
0	0	Goal setting (behaviour)	Problem solving	Action planning	Feedback on behaviour	social support	Information about health consequences	Information abour social & environmental consequences	Information about emotinal consequences
0	0	Goal setting (behaviour)	Problem solving	Action planning	Feedback on behaviour	social support	Information about health consequences	Information abour social & environmental consequences	Social comparison
0	0	Goal setting (behaviour)	Problem solving	Action planning	Feedback on behaviour	self monitoring of behaviour	Information about health consequences	Information abour social & environmental consequences	Information about emotinal consequences
0	0	Goal setting (behaviour)	Problem solving	Action planning	Feedback on behaviour	self monitoring of behaviour	Information about health consequences	Information abour social & environmental consequences	Social comparison
0	0	Goal setting (behaviour)	Problem solving	Action planning	Feedback on behaviour	self monitoring of behaviour	Information about health consequences	Information about emotinal consequences	Social comparison
0	0	Goal setting (behaviour)	Problem solving	Action planning	Feedback on behaviour	self monitoring of behaviour	Information abour social & environmental consequences	Information about emotinal consequences	Social comparison
0	0	Goal setting (behaviour)	Problem solving	Action planning	Feedback on behaviour	social support	Instruction on how to perform the behaviour	Information about health consequences	Information abour social & environmental consequences
0	0	Goal setting (behaviour)	Problem solving	Action planning	Feedback on behaviour	social support	Instruction on how to perform the behaviour	Information about health consequences	Information abour social & environmental consequences
0	0	Goal setting (behaviour)	Problem solving	Action planning	Feedback on behaviour	social support	Instruction on how to perform the behaviour	Information about health consequences	Information abour social & environmental consequences
0	0	Goal setting (behaviour)	Problem solving	Action planning	Feedback on behaviour	social support	Instruction on how to perform the behaviour	Information about health consequences	Information about emotinal consequences
0	0	Goal setting (behaviour)	Problem solving	Action planning	Feedback on behaviour	self monitoring of behaviour	Instruction on how to perform the behaviour	Information about health consequences	Information abour social & environmental consequences
0	0	Goal setting (behaviour)	Problem solving	Action planning	Feedback on behaviour	self monitoring of behaviour	Instruction on how to perform the behaviour	Information about health consequences	Information about emotinal consequences
0	0	Goal setting (behaviour)	Problem solving	Action planning	Feedback on behaviour	self monitoring of behaviour	Instruction on how to perform the behaviour	Information about health consequences	Information about emotinal consequences
0	0	Goal setting (behaviour)	Problem solving	Action planning	Feedback on behaviour	self monitoring of behaviour	Instruction on how to perform the behaviour	Information abour social & environmental consequences	Information about emotinal consequences
0	0	Goal setting (behaviour)	Problem solving	Action planning	Feedback on behaviour	self monitoring of behaviour	Instruction on how to perform the behaviour	Information abour social & environmental consequences	Information about emotinal consequences
0	0	Goal setting (behaviour)	Problem solving	Action planning	Feedback on behaviour	self monitoring of behaviour	Instruction on how to perform the behaviour	Information abour social & environmental consequences	Social comparison
0	0	Goal setting (behaviour)	Problem solving	Action planning	Feedback on behaviour	self monitoring of behaviour	Instruction on how to perform the behaviour	Information about emotinal consequences	Social comparison
0	0	Goal setting (behaviour)	Problem solving	Action planning	Feedback on behaviour	self monitoring of behaviour	Information about health consequences	Information abour social & environmental consequences	Information about emotinal consequences
0	0	Goal setting (behaviour)	Problem solving	Action planning	Feedback on behaviour	self monitoring of behaviour	social support	Information about health consequences	Information about emotinal consequences

2	2	Action planning	Review of behaviour goals	Feedback on behaviour	self monitoring of behaviour	Behavioural practice/rehearsal	Framing/reframing		
2	2	Action planning	Review of behaviour goals	Feedback on behaviour	self monitoring of behaviour	Graded tasks	Framing/reframing		
3	3	Goal setting (outcome)	Social support (emotional)	Graded tasks					
3	3	Goal setting (outcome)	Social support (emotional)	Credible source					
2	2	Goal setting (outcome)	Instruction on how to perform the behaviour	Generalisation of target behaviour					
3	3	Goal setting (outcome)	Instruction on how to perform the behaviour	Avoidance/reducing e1posure to the behaviour					
2	2	Goal setting (outcome)	Demonstration of the behaviour	Generalisation of target behaviour					
2	2	Goal setting (outcome)	Generalisation of target behaviour	Credible source					
3	3	Goal setting (outcome)	Credible source	Avoidance/reducing e1posure to the behaviour					
2	2	Feedback on behaviour	Self monitoring of outcome(s) of behaviour	Instruction on how to perform the behaviour					
2	2	Action planning	Feedback on behaviour	Credible source	Adding object to the environment				
2	2	Action planning	Demonstration of the behaviour	Graded tasks	Adding object to the environment				
2	2	Action planning	Graded tasks	Credible source	Adding object to the environment				
2	2	Feedback on behaviour	Instruction on how to perform the behaviour	Credible source	Adding object to the environment				
2	2	Feedback on behaviour	Graded tasks	Credible source	Adding object to the environment				
2	2	self monitoring of behaviour	Graded tasks	Credible source	Adding object to the environment				
2	2	social support	Graded tasks	Credible source	Adding object to the environment				
2	2	Instruction on how to perform the behaviour	Graded tasks	Credible source	Adding object to the environment				
3	3	Goal setting (outcome)	Feedback on behaviour	Social support (emotional)					
3	3	Goal setting (outcome)	Feedback on behaviour	Avoidance/reducing e1posure to the behaviour					
3	3	Goal setting (outcome)	self monitoring of behaviour	Social support (emotional)					
2	2	Goal setting (outcome)	self monitoring of behaviour	Generalisation of target behaviour					
3	3	Goal setting (outcome)	self monitoring of behaviour	Avoidance/reducing e1posure to the behaviour					
2	2	Goal setting (outcome)	Self monitoring of outcome(s) of behaviour	Credible source					
2	2	Problem solving	Feedback on behaviour	Credible source	Adding object to the environment				
2	2	Problem solving	Graded tasks	Credible source	Adding object to the environment				
2	2	Review of behaviour goals	Feedback on behaviour	self monitoring of behaviour	Instruction on how to perform the behaviour	Behavioural practice/rehearsal	Graded tasks	Framing/reframing	
2	2	Action planning	Review of behaviour goals	Feedback on behaviour	self monitoring of behaviour	Instruction on how to perform the behaviour	Behavioural practice/rehearsal	Graded tasks	Framing/reframing
2	2	Graded tasks	Credible source	Adding object to the environment					
2	2	Goal setting (behaviour)	Graded tasks	Credible source	Adding object to the environment				
2	1	Problem solving	Demonstration of the behaviour	Social comparison					
2	2	Problem solving	Demonstration of the behaviour	Generalisation of target behaviour					
3	3	Problem solving	Credible source	Avoidance/reducing e1posure to the behaviour					
3	2	Goal setting (outcome)	Feedback on behaviour	Feedback on outcomes of behaviour					
2	2	Review of behaviour goals	self monitoring of behaviour	Instruction on how to perform the behaviour	Behavioural practice/rehearsal	Graded tasks	Framing/reframing		
2	2	Feedback on behaviour	self monitoring of behaviour	Instruction on how to perform the behaviour	Behavioural practice/rehearsal	Graded tasks	Framing/reframing		
2	2	Action planning	Review of behaviour goals	Feedback on behaviour	self monitoring of behaviour	Instruction on how to perform the behaviour	Behavioural practice/rehearsal	Framing/reframing	
2	2	Action planning	Review of behaviour goals	Feedback on behaviour	self monitoring of behaviour	Instruction on how to perform the behaviour	Graded tasks	Framing/reframing	
2	2	Action planning	Review of behaviour goals	Feedback on behaviour	self monitoring of behaviour	Behavioural practice/rehearsal	Graded tasks	Framing/reframing	
2	2	Action planning	Review of behaviour goals	Feedback on behaviour	Instruction on how to perform the behaviour	Behavioural practice/rehearsal	Graded tasks	Framing/reframing	
2	2	Action planning	Review of behaviour goals	self monitoring of behaviour	Instruction on how to perform the behaviour	Behavioural practice/rehearsal	Graded tasks	Framing/reframing	
2	2	Action planning	Feedback on behaviour	self monitoring of behaviour	Instruction on how to perform the behaviour	Behavioural practice/rehearsal	Graded tasks	Framing/reframing	
2	2	Problem solving	Feedback on behaviour	self monitoring of behaviour	Credible source	Adding object to the environment			
2	2	Problem solving	Feedback on behaviour	social support	Credible source	Adding object to the environment			
2	2	Problem solving	Feedback on behaviour	Instruction on how to perform the behaviour	Credible source	Adding object to the environment			
2	2	Problem solving	Feedback on behaviour	Demonstration of the behaviour	Credible source	Adding object to the environment			
2	2	Problem solving	Feedback on behaviour	Graded tasks	Credible source	Adding object to the environment			
2	2	Problem solving	self monitoring of behaviour	Graded tasks	Credible source	Adding object to the environment			
2	2	Problem solving	social support	Graded tasks	Credible source	Adding object to the environment			
2	2	Problem solving	Instruction on how to perform the behaviour	Graded tasks	Credible source	Adding object to the environment			
3	3	Problem solving	Social support (emotional)	Graded tasks					
3	3	Problem solving	Social support (emotional)	Credible source					
2	2	Goal setting (behaviour)	Instruction on how to perform the behaviour	Graded tasks	Credible source	Adding object to the environment			
2	2	Goal setting (behaviour)	Demonstration of the behaviour	Graded tasks	Credible source	Adding object to the environment			
2	2	Problem solving	Action planning	Feedback on behaviour	Demonstration of the behaviour	Adding object to the environment			
2	2	Problem solving	Action planning	Feedback on behaviour	Credible source	Adding object to the environment			
2	2	Problem solving	Action planning	Demonstration of the behaviour	Graded tasks	Adding object to the environment			
2	2	Problem solving	Action planning	Graded tasks	Credible source	Adding object to the environment			
2	2	Goal setting (behaviour)	self monitoring of behaviour	Graded tasks	Credible source	Adding object to the environment			
2	2	Goal setting (behaviour)	social support	Graded tasks	Credible source	Adding object to the environment			
2	2	Problem solving	self monitoring of behaviour	Generalisation of target behaviour					
2	2	Problem solving	Self monitoring of outcome(s) of behaviour	Instruction on how to perform the behaviour					
2	2	Problem solving	Self monitoring of outcome(s) of behaviour	Avoidance/reducing e1posure to the behaviour					
2	2	Problem solving	Feedback on outcomes of behaviour	Demonstration of the behaviour					
2	2	Problem solving	Feedback on outcomes of behaviour	Graded tasks					
3	3	Problem solving	Social support (emotional)	Instruction on how to perform the behaviour					
2	2	Demonstration of the behaviour	Graded tasks	Credible source	Adding object to the environment				
2	2	Goal setting (behaviour)	Problem solving	Feedback on behaviour	Credible source	Adding object to the environment			
2	2	Goal setting (behaviour)	Problem solving	Graded tasks	Credible source	Adding object to the environment			
2	2	Goal setting (behaviour)	Action planning	Feedback on behaviour	Credible source	Adding object to the environment			

2	2	Problem solving	Feedback on behaviour	social support	Instruction on how to perform the behaviour	Demonstration of the behaviour	Credible source	Adding object to the environment	
2	2	Problem solving	Feedback on behaviour	social support	Instruction on how to perform the behaviour	Graded tasks	Credible source	Adding object to the environment	
2	2	Problem solving	Feedback on behaviour	social support	Demonstration of the behaviour	Graded tasks	Credible source	Adding object to the environment	
2	2	Problem solving	Feedback on behaviour	Instruction on how to perform the behaviour	Demonstration of the behaviour	Graded tasks	Credible source	Adding object to the environment	
2	2	Problem solving	self monitoring of behaviour	social support	Instruction on how to perform the behaviour	Graded tasks	Credible source	Adding object to the environment	
2	2	Problem solving	Action planning	social support	Instruction on how to perform the behaviour	Demonstration of the behaviour	Graded tasks	Credible source	
2	2	Problem solving	Action planning	social support	Instruction on how to perform the behaviour	Demonstration of the behaviour	Graded tasks	Adding object to the environment	
2	2	Problem solving	Action planning	social support	Instruction on how to perform the behaviour	Graded tasks	Credible source	Adding object to the environment	
2	2	Problem solving	Action planning	social support	Demonstration of the behaviour	Graded tasks	Credible source	Adding object to the environment	
2	2	Problem solving	Action planning	Instruction on how to perform the behaviour	Demonstration of the behaviour	Graded tasks	Credible source	Adding object to the environment	
2	2	Problem solving	Feedback on behaviour	self monitoring of behaviour	social support	Instruction on how to perform the behaviour	Credible source	Adding object to the environment	
2	2	Problem solving	Feedback on behaviour	self monitoring of behaviour	social support	Demonstration of the behaviour	Credible source	Adding object to the environment	
2	2	Problem solving	Feedback on behaviour	self monitoring of behaviour	social support	Graded tasks	Credible source	Adding object to the environment	
2	2	Goal setting (behaviour)	Problem solving	Action planning	Feedback on behaviour	social support	Instruction on how to perform the behaviour	Credible source	Adding object to the environment
2	2	Goal setting (behaviour)	Problem solving	Action planning	Feedback on behaviour	social support	Demonstration of the behaviour	Graded tasks	Credible source
2	2	Goal setting (behaviour)	Problem solving	Action planning	Feedback on behaviour	social support	Demonstration of the behaviour	Graded tasks	Adding object to the environment
2	2	Goal setting (behaviour)	Problem solving	Action planning	Feedback on behaviour	social support	Demonstration of the behaviour	Credible source	Adding object to the environment
2	2	Goal setting (behaviour)	Problem solving	Action planning	Feedback on behaviour	social support	Graded tasks	Credible source	Adding object to the environment
2	2	Goal setting (behaviour)	Problem solving	Action planning	Feedback on behaviour	Instruction on how to perform the behaviour	Demonstration of the behaviour	Graded tasks	Adding object to the environment
2	2	Goal setting (behaviour)	Problem solving	Action planning	Feedback on behaviour	Instruction on how to perform the behaviour	Demonstration of the behaviour	Credible source	Adding object to the environment
2	2	Goal setting (behaviour)	Problem solving	Action planning	Feedback on behaviour	Instruction on how to perform the behaviour	Graded tasks	Credible source	Adding object to the environment
2	2	Goal setting (behaviour)	Problem solving	Action planning	Feedback on behaviour	self monitoring of behaviour	social support	Credible source	Adding object to the environment
2	2	Goal setting (behaviour)	Problem solving	Action planning	Feedback on behaviour	self monitoring of behaviour	Instruction on how to perform the behaviour	Demonstration of the behaviour	Adding object to the environment
2	2	Goal setting (behaviour)	Problem solving	Action planning	Feedback on behaviour	self monitoring of behaviour	Instruction on how to perform the behaviour	Credible source	Adding object to the environment
2	2	Goal setting (behaviour)	Problem solving	Action planning	Feedback on behaviour	self monitoring of behaviour	Demonstration of the behaviour	Graded tasks	Adding object to the environment
2	2	Goal setting (behaviour)	Problem solving	Action planning	Feedback on behaviour	self monitoring of behaviour	Demonstration of the behaviour	Credible source	Adding object to the environment
2	2	Goal setting (behaviour)	Problem solving	Action planning	Feedback on behaviour	self monitoring of behaviour	Graded tasks	Credible source	Adding object to the environment
2	2	Goal setting (behaviour)	Problem solving	Action planning	Feedback on behaviour	social support	Instruction on how to perform the behaviour	Demonstration of the behaviour	Credible source
2	2	Goal setting (behaviour)	Problem solving	Action planning	Feedback on behaviour	social support	Instruction on how to perform the behaviour	Demonstration of the behaviour	Adding object to the environment
2	2	Feedback on behaviour	self monitoring of behaviour	social support	Instruction on how to perform the behaviour	Demonstration of the behaviour	Credible source	Adding object to the environment	
2	2	Feedback on behaviour	self monitoring of behaviour	social support	Instruction on how to perform the behaviour	Graded tasks	Credible source	Adding object to the environment	
2	2	Feedback on behaviour	self monitoring of behaviour	social support	Demonstration of the behaviour	Graded tasks	Credible source	Adding object to the environment	
2	2	Feedback on behaviour	self monitoring of behaviour	Instruction on how to perform the behaviour	Demonstration of the behaviour	Graded tasks	Credible source	Adding object to the environment	
2	2	Feedback on behaviour	social support	Instruction on how to perform the behaviour	Demonstration of the behaviour	Graded tasks	Credible source	Adding object to the environment	
2	2	self monitoring of behaviour	social support	Instruction on how to perform the behaviour	Demonstration of the behaviour	Graded tasks	Credible source	Adding object to the environment	
2	2	Goal setting (behaviour)	Problem solving	Action planning	Feedback on behaviour	self monitoring of behaviour	social support	Demonstration of the behaviour	Credible source
2	2	Goal setting (behaviour)	Problem solving	Action planning	Feedback on behaviour	self monitoring of behaviour	social support	Demonstration of the behaviour	Adding object to the environment
2	2	Action planning	Feedback on behaviour	social support	Demonstration of the behaviour	Graded tasks	Credible source	Adding object to the environment	
2	2	Action planning	Feedback on behaviour	Instruction on how to perform the behaviour	Demonstration of the behaviour	Graded tasks	Credible source	Adding object to the environment	
2	2	Action planning	self monitoring of behaviour	social support	Instruction on how to perform the behaviour	Demonstration of the behaviour	Graded tasks	Credible source	
2	2	Action planning	self monitoring of behaviour	social support	Instruction on how to perform the behaviour	Demonstration of the behaviour	Graded tasks	Adding object to the environment	
2	2	Action planning	self monitoring of behaviour	social support	Instruction on how to perform the behaviour	Graded tasks	Credible source	Adding object to the environment	
2	2	Action planning	self monitoring of behaviour	social support	Demonstration of the behaviour	Graded tasks	Credible source	Adding object to the environment	
2	2	Action planning	self monitoring of behaviour	Instruction on how to perform the behaviour	Demonstration of the behaviour	Graded tasks	Credible source	Adding object to the environment	
2	2	Action planning	social support	Instruction on how to perform the behaviour	Demonstration of the behaviour	Graded tasks	Credible source	Adding object to the environment	
2	2	Goal setting (behaviour)	Problem solving	Feedback on behaviour	social support	Instruction on how to perform the behaviour	Graded tasks	Credible source	Adding object to the environment
2	2	Goal setting (behaviour)	Problem solving	Feedback on behaviour	social support	Demonstration of the behaviour	Graded tasks	Credible source	Adding object to the environment
2	2	Goal setting (behaviour)	Problem solving	Feedback on behaviour	Instruction on how to perform the behaviour	Demonstration of the behaviour	Graded tasks	Credible source	Adding object to the environment
2	2	Goal setting (behaviour)	Problem solving	self monitoring of behaviour	social support	Instruction on how to perform the behaviour	Graded tasks	Credible source	Adding object to the environment
2	2	Goal setting (behaviour)	Problem solving	self monitoring of behaviour	social support	Demonstration of the behaviour	Graded tasks	Credible source	Adding object to the environment
2	2	Goal setting (behaviour)	Problem solving	self monitoring of behaviour	Instruction on how to perform the behaviour	Demonstration of the behaviour	Graded tasks	Credible source	Adding object to the environment
2	2	Goal setting (behaviour)	Problem solving	social support	Instruction on how to perform the behaviour	Demonstration of the behaviour	Graded tasks	Credible source	Adding object to the environment
2	2	Goal setting (behaviour)	Action planning	Feedback on behaviour	self monitoring of behaviour	social support	Instruction on how to perform the behaviour	Credible source	Adding object to the environment
2	2	Goal setting (behaviour)	Problem solving	Action planning	Instruction on how to perform the behaviour	Demonstration of the behaviour	Graded tasks	Credible source	Adding object to the environment
2	2	Goal setting (behaviour)	Problem solving	Feedback on behaviour	self monitoring of behaviour	social support	Instruction on how to perform the behaviour	Credible source	Adding object to the environment
2	2	Goal setting (behaviour)	Problem solving	Feedback on behaviour	self monitoring of behaviour	social support	Demonstration of the behaviour	Credible source	Adding object to the environment
2	2	Goal setting (behaviour)	Problem solving	Feedback on behaviour	self monitoring of behaviour	social support	Graded tasks	Credible source	Adding object to the environment
2	2	Goal setting (behaviour)	Problem solving	Feedback on behaviour	self monitoring of behaviour	Instruction on how to perform the behaviour	Demonstration of the behaviour	Credible source	Adding object to the environment
2	2	Goal setting (behaviour)	Problem solving	Feedback on behaviour	self monitoring of behaviour	Instruction on how to perform the behaviour	Graded tasks	Credible source	Adding object to the environment

[illegible]
