# Supplementary material for: Which combinations of behaviour change techniques work best? Evidence from meta-review of interventions addressing healthy diet and exercise after breast cancer
Source: Int J Behav Nutr Phys Act. 2026 Aug 4;23:104. doi: 10.1186/s12966-026-01961-6 (PMC13725973; doi:10.1186/s12966-026-01961-6)
Supplement: Supplementary file 2 [file 12966_2026_1961_MOESM1_ESM.docx]

**Which Combinations of Behaviour Change Techniques Work Best?**

**Evidence from Meta-review of Interventions Addressing Healthy Diet and Exercise after Breast Cancer**

**Additional file**

**Table S1**

*Keywords used in Searches of Databases*

| **Category** | **Key words** |
| --- | --- |
| Diagnosis | "breast cancer" OR "breast neoplasm*" OR "breast tumor*" OR "breast cancer patient*" OR "breast cancer survivor*" |
| Type of behavior | "physical activ*" OR "physical exercise*" OR "physical intensity" OR "physical exertion" OR "sport" OR "metabolic equivalent" OR "sedentary" OR "inactiv*" OR "diet" OR "unhealthy diet" OR "nutrition" OR "dietary behavio*" OR "eating behavio*" OR "weight loss" OR "weight control" OR "obes*" OR "overweight" OR "calorie restrict*" OR "alcohol consumption" OR "alcohol intake" OR "body mass reduction" OR "Body weight reduction" OR "smoking" OR "nicotine" OR "vaping" |
| Kind of intervention | "behavio* change technique*" OR "behavio* change strateg*" OR "behavio* change theor*" OR "motivational interview*" OR "motivational dialogue" OR "BCT taxonomy" OR "Michie" |
| Type of intervention | "mhealth" OR "ehealth" OR "application*" OR "digital intervention*" OR "lifestyle intervention*" OR "intervention*" OR "training*" OR "therap*" OR "treatment*" OR "program*" |
| Design of the study | "systematic review*" OR "meta-synthesis" OR "meta-review" OR "synthesis of data" OR "critical analysis of the literature" OR "metaanaly*" OR "meta-analy*" OR "meta analy*" OR "systematic overview*" OR "systematic review*" OR "literature search" OR "review of literature" OR "search of literature" OR "search of the literature" OR "Qualitative analysis" OR "Quantitative analysis" OR systematic search" OR "comprehensive search" OR "manual search" OR "scoping study" OR "meta-study" OR "literature-based study" OR "compilation of the literature" OR "compilation of studies" OR "meta-regression" OR "meta-evidence” |

**S1. List of texts entered in AS Review as a relevant and irrelevant**

After entering the database to AS Review and the deletion of duplicates, 1,939 articles were retained for which 8 texts were entered as examples of relevant and irrelevant publications.

**Relevant:**

Cooper KB, Lapierre S, Carrera Seoane M, Lindstrom K, Pritschmann R, Donahue M, et al. Behavior change techniques in digital physical activity interventions for breast cancer survivors: a systematic review. Transl Behav Med. 2023;13(4):268-280. doi:10.1093/tbm/ibac111.

Hailey V, Rojas-Garcia A, Kassianos AP. A systematic review of behaviour change techniques used in interventions to increase physical activity among breast cancer survivors. Breast Cancer. 2022;29(2):193-208. doi:10.1007/s12282-021-01323-z.

Liu MG, Davis GM, Kilbreath SL, Yee J. Physical activity interventions using behaviour change theories for women with breast cancer: a systematic review and meta-analysis. J Cancer Surviv. 2022;16:1127–1148. doi:10.1007/s11764-021-01104-9.

Perperidi M, Saliari D, Christakis C, Huybrechts I, Saloustros E, Theodorakis Y, et al. Identifying the effective behaviour change techniques in nutrition and physical activity interventions for the treatment of overweight/obesity in post-treatment breast cancer survivors: a systematic review. Cancer Causes Control. 2023;34(8):683-703. doi:10.1007/s10552-023-01707-w.

Pudkasam S, Feehan J, Talevski J, Vingrys K, Polman R, Chinlumprasert N, et al. Motivational strategies to improve adherence to physical activity in breast cancer survivors: a systematic review and meta-analysis. Maturitas. 2021;152:32-47. doi:10.1016/j.maturitas.2021.06.008.

Short CE, James EL, Stacey F, Plotnikoff RC. A qualitative synthesis of trials promoting physical activity behaviour change among post-treatment breast cancer survivors. J Cancer Surviv. 2013;7:570–581. doi:10.1007/s11764-013-0296-4.

Sheeran P, Abraham C, Jones K, Villegas ME, Avishai A, Symes YR, Ellinger H, Miles E, Gates KM, Wright CE, Ribisi KM, Mayer DK. Promoting physical activity among cancer survivors: meta-analysis and meta-CART analysis of randomized controlled trials. Health Psychol. 2019;38(6):467–480. doi:10.1037/hea0000712.

Sheeran P, Wright CE, Listrom O, Klein WM, Rothman AJ. Which intervention strategies promote the adoption and maintenance of physical activity? Evidence from behavioral trials with cancer survivors. Ann Behav Med. 2023;57(9):708–721. doi:10.1093/abm/kaad002.

**Irrelevant:**

2017 Oncology Nursing Society Annual Congress Podium and Poster Abstracts. [https://search.ebscohost.com/login.aspx?direct=true&db=asn&AN=140411869&site=ehost-live](https://search.ebscohost.com/login.aspx?direct=true&db=asn&AN=140411869&site=ehost-live)

23rd Family Medicine Scientific Conference. [https://search.ebscohost.com/login.aspx?direct=true&db=asn&AN=153571563&site=ehost-live](https://search.ebscohost.com/login.aspx?direct=true&db=asn&AN=153571563&site=ehost-live)

26th Annual Conference of the International Society for Quality of Life Research. [https://search.ebscohost.com/login.aspx?direct=true&db=asn&AN=138666758&site=ehost-live](https://search.ebscohost.com/login.aspx?direct=true&db=asn&AN=138666758&site=ehost-live)

Culture IJ of B, Mind and. A comparison of individual and group motivational interviewing in the improvement of medication adherence in prostate cancer. Int J Body Mind Cult. 2024;10(4):542–553. Available from: <https://ijbmc.org/index.php/ijbmc/article/view/609>

Kunstler B, O’Halloran P, Cook J, Kemp J, Finch C. “…like you’re pushing the snowball back up hill”—the experiences of Australian physiotherapists promoting non-treatment physical activity: a qualitative study. AIMS Med Sci. 2018;5(3):224–237. doi:10.3934/medsci.2018.3.224

Lara J, Hobbs N, Moynihan PJ, Meyer TD, Adamson AJ, Errington L, et al. Effectiveness of dietary interventions among adults of retirement age: a systematic review and meta-analysis of randomized controlled trials. BMC Med. 2014;12:60. doi:10.1186/1741-7015-12-60

Samdal GB, Eide GE, Barth T, Williams G, Meland E. Effective behaviour change techniques for physical activity and healthy eating in overweight and obese adults: systematic review and meta-regression analyses. Int J Behav Nutr Phys Act. 2017;14:42. doi:10.1186/s12966-017-0494-y

Wyse R, Wolfenden L, Campbell E, Campbell KJ, Wiggers J, Brennan L, et al. A cluster randomized controlled trial of a telephone-based parent intervention to increase preschoolers’ fruit and vegetable consumption. Am J Clin Nutr. 2012;96(1):102–110. doi:10.3945/ajcn.111.030585

**S2. Python Code for Data Processing** (Python 3.13.5)

import pandas as pd

from itertools import combinations

\# load data from csv file CSV

input_csv_path_new = 'in.csv'

data_df_new = pd.read_csv(input_csv_path_new, delimiter=';')

\# Fill in the missing values with empty strings (NaN -> '')

data_df_new = data_df_new.fillna('')

\# Download efficiency information (rows 0–2)

effectiveness_info = data_df_new.iloc[0:3].copy()

effectiveness_info.index = ['PA Effective', 'Dieta Effective', 'Weight Loss Effective']

\# We do not remove the header with techniques, we only reset the indexes

data_df_new = data_df_new.iloc[3:].copy().reset_index(drop=True)

\# Change the name of the first column to 'Technika'

data_df_new.rename(columns={data_df_new.columns[0]: 'Technika'}, inplace=True)

\# Set column 'Technika' as index

data_df_new.set_index('Technika', inplace=True)

\# Dictionaries for storing results

combination_counts = {}

effectiveness_counts = {}

\# Iterate through each column (badanie) to find combinations of techniques

for column in data_df_new.columns:

present_techniques = data_df_new.index[data_df_new[column].astype(str).str.strip().isin(['1', '1*'])].tolist()

pa_value = effectiveness_info.loc['PA Effective', column].strip().lower()

dieta_value = effectiveness_info.loc['Dieta Effective', column].strip().lower()

weight_loss_value = effectiveness_info.loc['Weight Loss Effective', column].strip().lower()

pa_valid = pa_value not in ['-']

dieta_valid = dieta_value not in ['-']

weight_loss_valid = weight_loss_value not in ['-']

pa_effective = pa_value in ['effective', 'ef']

dieta_effective = dieta_value in ['effective', 'ef']

weight_loss_effective = weight_loss_value in ['effective', 'ef']

pa_ineffective = pa_value in ['ineffective', 'i-ef']

dieta_ineffective = dieta_value in ['ineffective', 'i-ef']

weight_loss_ineffective = weight_loss_value in ['ineffective', 'i-ef']

pa_no_data = pa_value in ['no data', 'no dta']

dieta_no_data = dieta_value in ['no data', 'no dta']

weight_loss_no_data = weight_loss_value in ['no data', 'no dta']

pa_dieta_valid = pa_valid and dieta_valid

pa_weight_loss_valid = pa_valid and weight_loss_valid

dieta_weight_loss_valid = dieta_valid and weight_loss_valid

all_three_valid = pa_valid and dieta_valid and weight_loss_valid

for r in range(2, len(present_techniques) + 1):

for combo in combinations(present_techniques, r):

combo_key = ', '.join(combo)

if combo_key in combination_counts:

combination_counts[combo_key] += 1

else:

combination_counts[combo_key] = 1

if combo_key not in effectiveness_counts:

effectiveness_counts[combo_key] = {

'PA Effective': 0, 'Dieta Effective': 0, 'Weight Loss Effective': 0,

'PA & Dieta Effective': 0, 'Dieta & Weight Loss Effective': 0, 'PA & Weight Loss Effective': 0,

'PA & Dieta & Weight Loss Effective': 0,

'PA Ineffective': 0, 'Dieta Ineffective': 0, 'Weight Loss Ineffective': 0,

'PA & Dieta Ineffective': 0, 'Dieta & Weight Loss Ineffective': 0, 'PA & Weight Loss Ineffective': 0,

'PA & Dieta & Weight Loss Ineffective': 0,

'PA No Data': 0, 'Dieta No Data': 0, 'Weight Loss No Data': 0,

'PA & Dieta No Data': 0, 'Dieta & Weight Loss No Data': 0, 'PA & Weight Loss No Data': 0,

'PA & Dieta & Weight Loss No Data': 0,

'Total PA': 0, 'Total Dieta': 0, 'Total Weight Loss': 0,

'Total PA & Dieta': 0, 'Total PA & Weight Loss': 0, 'Total Dieta & Weight Loss': 0,

'Total PA & Dieta & Weight Loss': 0

}

if pa_effective:

effectiveness_counts[combo_key]['PA Effective'] += 1

if dieta_effective:

effectiveness_counts[combo_key]['Dieta Effective'] += 1

if weight_loss_effective:

effectiveness_counts[combo_key]['Weight Loss Effective'] += 1

if pa_ineffective:

effectiveness_counts[combo_key]['PA Ineffective'] += 1

if dieta_ineffective:

effectiveness_counts[combo_key]['Dieta Ineffective'] += 1

if weight_loss_ineffective:

effectiveness_counts[combo_key]['Weight Loss Ineffective'] += 1

if pa_no_data:

effectiveness_counts[combo_key]['PA No Data'] += 1

if dieta_no_data:

effectiveness_counts[combo_key]['Dieta No Data'] += 1

if weight_loss_no_data:

effectiveness_counts[combo_key]['Weight Loss No Data'] += 1

if pa_valid:

effectiveness_counts[combo_key]['Total PA'] += 1

if dieta_valid:

effectiveness_counts[combo_key]['Total Dieta'] += 1

if weight_loss_valid:

effectiveness_counts[combo_key]['Total Weight Loss'] += 1

if pa_dieta_valid:

effectiveness_counts[combo_key]['Total PA & Dieta'] += 1

if pa_weight_loss_valid:

effectiveness_counts[combo_key]['Total PA & Weight Loss'] += 1

if dieta_weight_loss_valid:

effectiveness_counts[combo_key]['Total Dieta & Weight Loss'] += 1

if all_three_valid:

effectiveness_counts[combo_key]['Total PA & Dieta & Weight Loss'] += 1

\# Preparing data for recording

output_data_new = []

for combo, count in combination_counts.items():

techniques_list = combo.split(', ')

row = [count] + [effectiveness_counts[combo][key] for key in effectiveness_counts[combo].keys()] + techniques_list

if len(techniques_list) > 2 and count > 2:

output_data_new.append(row)

max_techniques = max(len(row) - len(effectiveness_counts[combo].keys()) - 1 for row in output_data_new)

headers_new = ['Liczba badań'] + list(effectiveness_counts[combo].keys()) + [f'Technika {i+1}' for i in range(max_techniques)]

output_df_new = pd.DataFrame(output_data_new, columns=headers_new)

output_df_new_sorted = output_df_new.sort_values(by='Liczba badań', ascending=False)

output_df_new_sorted.to_csv('pary_trojka.csv', index=False)

**Metadata:**

**the names and content of the variables in the data file (used in Python code) ***

**Liczba badań** – the total number of studies for a given combination

**PA Effective** – the number of effective studies supporting a given combination for the Physical Activity outcome

**Dieta Effective** – the number of effective studies supporting a given combination for the Healthy Nutrition outcome

**Weight Loss Effective** – the number of effective studies supporting a given combination for the Healthy Body weight outcome

**PA & Dieta Effective** – the number of effective studies supporting a given combination for the Physical Activity and Healthy Nutrition outcomes

**Dieta & Weight Loss Effective** – the number of effective studies supporting a given combination for the Healthy Nutrition and Healthy Body weight outcomes

**PA & Weight Loss Effective** – the number of effective studies supporting a given combination for the Physical Activity and Healthy Body weight outcomes

**PA & Dieta & Weight Loss Effective** – the number of effective studies supporting a given combination for the Physical Activity, Healthy Nutrition and Healthy Body weight outcomes

**PA Ineffective** – the number of ineffective studies (not supporting a given combination) for the Physical Activity outcome

**Dieta Ineffective** – the number of ineffective studies (not supporting a given combination) for the Healthy Nutrition outcome

**Weight Loss Ineffective** – the number of ineffective studies (not supporting a given combination) for the Healthy Body weight outcome

**PA & Dieta Ineffective** – the number of ineffective studies (not supporting a given combination) for the Physical Activity and Healthy Nutrition outcomes

**Dieta & Weight Loss Ineffective** – the number of ineffective studies (not supporting a given combination) for the Healthy Nutrition and Healthy Body weight outcomes

**PA & Weight Loss Ineffective** – the number of ineffective studies (not supporting a given combination) for the Physical Activity and Healthy Body weight outcomes

**PA & Dieta & Weight Loss Ineffective** – the number of ineffective studies (not supporting a given combination) for the Physical Activity, Healthy Nutrition and Healthy Body weight outcomes

**PA No Data** – the number of studies lacking information on effectiveness (not suitable for coding) for the Physical Activity outcome

**Dieta No Data** – the number of studies lacking information on effectiveness (not suitable for coding) for the Healthy Nutrition outcome

**Weight Loss No Data** – the number of studies lacking information on effectiveness (not suitable for coding) for the Healthy Body weight outcome

**PA & Dieta No Data** – the number of studies lacking information on effectiveness (not suitable for coding) for the Physical Activity and Healthy Nutrition outcomes

**Dieta & Weight Loss No Data** – the number of studies lacking information on effectiveness (not suitable for coding) for the Healthy Nutrition and Healthy Body weight outcome

**PA & Weight Loss No Data** – the number of studies lacking information on effectiveness (not suitable for coding) for the Physical Activity and Healthy Body weight outcome

**PA & Dieta & Weight Loss No Data** – the number of studies lacking information on effectiveness (not suitable for coding) for the Physical Activity, Healthy Nutrition and Healthy Body weight outcome

**Total PA** – the total number of studies for the Physical Activity outcome

**Total Dieta** – the total number of studies for the Healthy Nutrition outcome

**Total Weight Loss** – the total number of studies for the Healthy Body weight outcome

**Total PA & Dieta** – the total number of studies for the Physical Activity and Healthy Nutrition outcomes

**Total PA & Weight Loss** – the total number of studies for the Physical Activity and Healthy Body weight outcomes

**Total Dieta & Weight Loss** – the total number of studies for the Healthy Nutrition and Healthy Body weight outcomes

**Total PA & Dieta & Weight Loss** – the total number of studies for the Physical Activity, Healthy Nutrition and Healthy Body weight outcomes

**Technika 1, 2, ….12** – techniques included in a given combination

**pa** – % effectiveness of studies for the Physical Activity outcome (calculated as the proportion of effective to ineffective studies)

**dieta** – % effectiveness of studies for the Healthy Nutrition outcome (calculated as the proportion of effective to ineffective studies)

**wl** – % effectiveness of studies for the Healthy Body weight outcome (calculated as the proportion of effective to ineffective studies)

**pa&d** – % effectiveness of studies for the physical activity and healthy nutrition outcome (calculated as the proportion of effective to ineffective studies)

**d&wl** – % effectiveness of studies for the healthy nutrition and Healthy Body weight outcome (calculated as the proportion of effective to ineffective studies)

**pa&wl** – % effectiveness of studies for the physical activity and Healthy Body weight outcome (calculated as the proportion of effective to ineffective studies)

**ilość Technik** – number of techniques in a given combination

**PA tak/nie** – combinations meeting the criteria of at least 3 studies testing a given combination for physical activity and achieving a minimum of 60% effectiveness (yes – meets the criteria)

**Dieta tak/nie** – combinations meeting the criteria of at least 3 studies testing a given combination for Healthy Nutrition and achieving a minimum of 60% effectiveness (yes – meets the criteria)

**WL tak/ nie** – combinations meeting the criteria of at least 3 studies testing a given combination for Healthy Body weight and achieving a minimum of 60% effectiveness (yes – meets the criteria)

**Table S2**
*Risk of Bias Across the ROBIS Domains for Each of the Reviews Included in the Meta-review*

|  | 1. Study eligibility criteria | 2. Identification and selection of studies | 3. Data collection and study appraisal | 4. Synthesis and findings | Risk of Bias in the criteria |
| --- | --- | --- | --- | --- | --- |
| Amireault, 2016 (28) | HIGH | HIGH | LOW | LOW | HIGH |
| Brunet, 2020 (29) | LOW | LOW | LOW | HIGH | HIGH |
| Cooper, 2023 (8) | LOW | HIGH | LOW | HIGH | HIGH |
| Finne, 2018 (30) | LOW | HIGH | LOW | LOW | HIGH |
| Gan, 2022 (31) | HIGH | LOW | LOW | HIGH | HIGH |
| Gan, Cheng, 2022 (32) | LOW | LOW | LOW | HIGH | HIGH |
| Goode, 2015 (33) | HIGH | LOW | LOW | HIGH | HIGH |
| Grimmett, 2019 (34) | LOW | LOW | LOW | LOW | LOW |
| Haberlin, 2018 (35) | LOW | LOW | LOW | LOW | LOW |
| Hailey, 2022 (10) | LOW | HIGH | HIGH | HIGH | HIGH |
| Hoedjes, 2017 (37) | HIGH | LOW | HIGH | HIGH | HIGH |
| Ibeggazene, 2021 (37) | LOW | HIGH | LOW | LOW | HIGH |
| Ijsbrandy, 2018 (38) | LOW | LOW | LOW | LOW | LOW |
| Kiss, 2019 (39) | LOW | LOW | LOW | LOW | LOW |
| Knols, 2010 (40) | HIGH | HIGH | LOW | HIGH | HIGH |
| Ning, 2022 (41) | LOW | LOW | LOW | LOW | LOW |
| Perperidi, 2023 (9) | LOW | LOW | LOW | LOW | LOW |
| Playdon, 2013 (42) | HIGH | HIGH | HIGH | HIGH | HIGH |
| Pudkasam, 2021 (20) | LOW | HIGH | LOW | HIGH | HIGH |
| Quadflieg, 2023 (43) | LOW | LOW | LOW | LOW | LOW |
| Roberts, 2017 (44) | LOW | LOW | LOW | LOW | LOW |
| Rodrigues, 2023 (45) | LOW | HIGH | LOW | LOW | HIGH |
| Salisbury, 2023 (46) | LOW | LOW | LOW | HIGH | HIGH |
| Seven, 2023 (47) | LOW | LOW | HIGH | HIGH | HIGH |
| Short, 2013 (48) | LOW | HIGH | LOW | HIGH | HIGH |
| Singh, 2022 (49) | LOW | LOW | LOW | LOW | LOW |
| Sremanakowa, 2021 (12) | LOW | LOW | LOW | LOW | LOW |
| Stacey, 2015 (50) | HIGH | HIGH | LOW | HIGH | HIGH |
| Swartz, 2017 (51) | LOW | LOW | LOW | LOW | LOW |
| Takemura, 2023 (52) | LOW | LOW | LOW | LOW | LOW |
| Teo, 2023 (53) | LOW | LOW | LOW | LOW | LOW |
| Turner, 2018 (54) | LOW | LOW | LOW | LOW | LOW |

**Table S3**
*Most Effective BCT Combinationsfor Physical Activity, Nutrition, and Healthy Body Weight in Relation to the Number of Techniques Used*

| Total number of studies | PA Effective | Healthy Nutrition behaviour Effective | Healthy Body weight Effective | PA Ineffective | Healthy Nutrition Behaviour Ineffective | Healthy Body weight Ineffective | BCT #1 | BCT #2 | BCT #3 | BCT #4 | BCT #5 | BCT #6 | PA Effectiveness [%] | Healthy Nutrition Behaviour Effectiveness [%] | Healthy body weight Effectiveness [%] | Total number of BCTs |
| --- | --- | --- | --- | --- | --- | --- | --- | --- | --- | --- | --- | --- | --- | --- | --- | --- |
| 16 | 13 | 5 | 4 | 1 | 0 | 0 | Goal setting (behaviour) | Problem solving | Demonstration of the behaviour |  |  |  | 93 | 100 | 100 | 3 |
| 15 | 12 | 4 | 5 | 1 | 0 | 0 | Problem solving | Self-monitoring of behaviour | Demonstration of the behaviour |  |  |  | 92 | 100 | 100 | 3 |
| 14 | 11 | 4 | 4 | 1 | 0 | 0 | Goal setting (behaviour) | Problem solving | Self-monitoring of behaviour | Demonstration of the behaviour |  |  | 92 | 100 | 100 | 4 |
| 12 | 9 | 4 | 5 | 1 | 0 | 0 | Problem solving | Self-monitoring of behaviour | Instruction on how to perform the behaviour | Demonstration of the behaviour |  |  | 90 | 100 | 100 | 4 |
| 12 | 9 | 4 | 5 | 1 | 0 | 0 | Problem solving | Feedback on behaviour | Demonstration of the behaviour |  |  |  | 90 | 100 | 100 | 3 |
| 12 | 9 | 4 | 5 | 1 | 0 | 0 | Self-monitoring of behaviour | Demonstration of the behaviour | Credible source |  |  |  | 90 | 100 | 100 | 3 |
| 12 | 9 | 5 | 4 | 1 | 0 | 0 | Goal setting (behaviour) | Problem solving | Instruction on how to perform the behaviour | Demonstration of the behaviour |  |  | 90 | 100 | 100 | 4 |
| 11 | 8 | 3 | 5 | 1 | 0 | 0 | Feedback on behaviour | Demonstration of the behaviour | Credible source |  |  |  | 89 | 100 | 100 | 3 |
| 11 | 8 | 3 | 5 | 1 | 0 | 0 | Problem solving | Feedback on behaviour | Self-monitoring of behaviour | Demonstration of the behaviour |  |  | 89 | 100 | 100 | 4 |
| 11 | 8 | 4 | 4 | 1 | 0 | 0 | Goal setting (behaviour) | Problem solving | Feedback on behaviour | Demonstration of the behaviour |  |  | 89 | 100 | 100 | 4 |
| 11 | 8 | 4 | 4 | 1 | 0 | 0 | Goal setting (behaviour) | Problem solving | Self-monitoring of behaviour | Instruction on how to perform the behaviour | Demonstration of the behaviour |  | 89 | 100 | 100 | 5 |
| 11 | 8 | 4 | 5 | 1 | 0 | 0 | Self-monitoring of behaviour | Instruction on how to perform the behaviour | Demonstration of the behaviour | Credible source |  |  | 89 | 100 | 100 | 4 |
| 11 | 8 | 4 | 5 | 1 | 0 | 0 | Problem solving | Self-monitoring of behaviour | Instruction on how to perform the behaviour | Demonstration of the behaviour | Graded tasks |  | 89 | 100 | 100 | 5 |
| 11 | 8 | 4 | 4 | 1 | 0 | 0 | Goal setting (behaviour) | Self-monitoring of behaviour | Demonstration of the behaviour | Credible source |  |  | 89 | 100 | 100 | 4 |
| 11 | 8 | 4 | 5 | 1 | 0 | 0 | Problem solving | Demonstration of the behaviour | Graded tasks |  |  |  | 89 | 100 | 100 | 3 |
| 11 | 8 | 4 | 5 | 1 | 0 | 0 | Problem solving | Instruction on how to perform the behaviour | Demonstration of the behaviour | Graded tasks |  |  | 89 | 100 | 100 | 4 |
| 11 | 8 | 4 | 5 | 1 | 0 | 0 | Goal setting (behaviour) | Problem solving | Goal setting (outcome) |  |  |  | 89 | 100 | 100 | 3 |
| 11 | 8 | 4 | 5 | 1 | 0 | 0 | Problem solving | Feedback on behaviour | Instruction on how to perform the behaviour | Demonstration of the behaviour |  |  | 89 | 100 | 100 | 4 |
| 11 | 8 | 4 | 5 | 1 | 0 | 0 | Problem solving | Self-monitoring of behaviour | Demonstration of the behaviour | Graded tasks |  |  | 89 | 100 | 100 | 4 |
| 10 | 8 | 4 | 3 | 1 | 0 | 0 | Goal setting (behaviour) | Social support | Demonstration of the behaviour | Credible source |  |  | 89 | 100 | 100 | 4 |
| 18 | 14 | 7 | 5 | 2 | 0 | 0 | Problem solving | Self-monitoring of behaviour | Graded tasks |  |  |  | 88 | 100 | 100 | 3 |
| 17 | 14 | 5 | 3 | 2 | 0 | 0 | Self-monitoring of behaviour | Social support | Graded tasks |  |  |  | 88 | 100 | 100 | 3 |
| 10 | 7 | 3 | 5 | 1 | 0 | 0 | Problem solving | Feedback on behaviour | Instruction on how to perform the behaviour | Demonstration of the behaviour | Graded tasks |  | 88 | 100 | 100 | 5 |
| 10 | 7 | 4 | 4 | 1 | 0 | 0 | Goal setting (behaviour) | Problem solving | Instruction on how to perform the behaviour | Demonstration of the behaviour | Graded tasks |  | 88 | 100 | 100 | 5 |
| 10 | 7 | 4 | 4 | 1 | 0 | 0 | Goal setting (behaviour) | Problem solving | Feedback on behaviour | Instruction on how to perform the behaviour | Demonstration of the behaviour |  | 88 | 100 | 100 | 5 |
| 10 | 7 | 4 | 4 | 1 | 0 | 0 | Goal setting (behaviour) | Problem solving | Self monitoring of behaviour | Demonstration of the behaviour | Graded tasks |  | 88 | 100 | 100 | 5 |
| 10 | 7 | 4 | 5 | 1 | 0 | 0 | Instruction on how to perform the behaviour | Graded tasks | Credible source |  |  |  | 88 | 100 | 100 | 3 |
| 10 | 7 | 4 | 5 | 1 | 0 | 0 | Self-monitoring of behaviour | Graded tasks | Credible source |  |  |  | 88 | 100 | 100 | 3 |
| 10 | 7 | 3 | 4 | 1 | 0 | 0 | Goal setting (behaviour) | Problem solving | Feedback on behaviour | Self-monitoring of behaviour | Demonstration of the behaviour |  | 88 | 100 | 100 | 5 |
| 10 | 7 | 4 | 5 | 1 | 0 | 0 | Goal setting (behaviour) | Problem solving | Goal setting (outcome) | Feedback on behaviour |  |  | 88 | 100 | 100 | 4 |
| 10 | 7 | 3 | 5 | 1 | 0 | 0 | Goal setting (behaviour) | Problem solving | Goal setting (outcome) | Self-monitoring of behaviour |  |  | 88 | 100 | 100 | 4 |
| 10 | 7 | 4 | 4 | 1 | 0 | 0 | Goal setting (behaviour) | Problem solving | Demonstration of the behaviour | Graded tasks |  |  | 88 | 100 | 100 | 4 |
| 10 | 7 | 4 | 4 | 1 | 0 | 0 | Goal setting (behaviour) | Problem solving | Self monitoring of behaviour | Instruction on how to perform the behaviour | Demonstration of the behaviour | Graded tasks | 88 | 100 | 100 | 6 |
| 10 | 7 | 3 | 5 | 1 | 0 | 0 | Feedback on behaviour | Self-monitoring of behaviour | Demonstration of the behaviour | Credible source |  |  | 88 | 100 | 100 | 4 |
| 10 | 7 | 4 | 5 | 1 | 0 | 0 | Self-monitoring of behaviour | Instruction on how to perform the behaviour | Graded tasks | Credible source |  |  | 88 | 100 | 100 | 4 |
| 10 | 7 | 3 | 5 | 1 | 0 | 0 | Problem solving | Feedback on behaviour | Self-monitoring of behaviour | Instruction on how to perform the behaviour | Demonstration of the behaviour | Graded tasks | 88 | 100 | 100 | 6 |
| 10 | 7 | 3 | 5 | 1 | 0 | 0 | Feedback on behaviour | Instruction on how to perform the behaviour | Demonstration of the behaviour | Credible source |  |  | 88 | 100 | 100 | 4 |
| 10 | 7 | 3 | 5 | 1 | 0 | 0 | Problem solving | Feedback on behaviour | Self-monitoring of behaviour | Instruction on how to perform the behaviour | Demonstration of the behaviour |  | 88 | 100 | 100 | 5 |
| 10 | 7 | 3 | 5 | 1 | 0 | 0 | Problem solving | Feedback on behaviour | Self-monitoring of behaviour | Demonstration of the behaviour | Graded tasks |  | 88 | 100 | 100 | 5 |
| 10 | 7 | 3 | 5 | 1 | 0 | 0 | Problem solving | Feedback on behaviour | Demonstration of the behaviour | Graded tasks |  |  | 88 | 100 | 100 | 4 |
| 10 | 7 | 4 | 4 | 1 | 0 | 0 | Goal setting (behaviour) | Self-monitoring of behaviour | Instruction on how to perform the behaviour | Demonstration of the behaviour | Credible source |  | 88 | 100 | 100 | 5 |
| 17 | 13 | 7 | 4 | 2 | 0 | 0 | Goal setting (behaviour) | Problem solving | Graded tasks |  |  |  | 87 | 100 | 100 | 3 |
| 17 | 13 | 7 | 4 | 2 | 0 | 0 | Goal setting (behaviour) | Problem solving | Self-monitoring of behaviour | Graded tasks |  |  | 87 | 100 | 100 | 4 |
| 16 | 13 | 5 | 3 | 2 | 0 | 0 | Goal setting (behaviour) | Social support | Graded tasks |  |  |  | 87 | 100 | 100 | 3 |
| 16 | 13 | 5 | 3 | 2 | 0 | 0 | Goal setting (behaviour) | self monitoring of behaviour | social support | Graded tasks |  |  | 87 | 100 | 100 | 4 |
| 38 | 31 | 12 | 6 | 5 | 1 | 0 | Goal setting (behaviour) | Problem solving | Self-monitoring of behaviour |  |  |  | 86 | 92 | 100 | 3 |
|  |  |  |  |  |  |  |  |  |  |  |  |  |  |  |  |  |

*Note:* BCT – behavior change technique*;* PA – Physical Activity; PA Effective – the number of effective studies supporting a given combination for the Physical Activity outcome; Healthy Nutrition Behaviour Effective – the number of effective studies supporting a given combination for the Nutrition behavior outcome; Healthy Body weight Effective - the number of effective studies supporting a given combination for Healthy Body weight outcome; PA Ineffective – the number of ineffective studies (not supporting a given combination) for the Physical Activity outcome; Healthy Nutrition Behaviour Ineffective – the number of ineffective studies (not supporting a given combination) for the Healthy Nutrition behavior outcome; Healthy Body weight Ineffective – the number of ineffective studies (not supporting a given combination) for the Healthy Body weight outcome; BCT 1,2...-6 – BCTs included in a given combination; PA Effectiveness [%] – % effectiveness of studies for the Physical Activity outcome (calculated as the proportion of effective to ineffective studies); Healthy Nutrition Behaviour Effectiveness [%] – % effectiveness of studies for the Nutrition behavior outcome (calculated as the proportion of effective to ineffective studies); Healthy body weight Effectiveness [%] – % effectiveness of studies for the Healthy body weight outcome (calculated as the proportion of effective to ineffective studies); Total number of techniques – the number of techniques in a given combination
